# A Comprehensive Survey of Statistical Approaches for Differential Expression Analysis in Single-Cell RNA Sequencing Studies

**DOI:** 10.3390/genes12121947

**Published:** 2021-12-02

**Authors:** Samarendra Das, Anil Rai, Michael L. Merchant, Matthew C. Cave, Shesh N. Rai

**Affiliations:** 1 Division of Statistical Genetics, ICAR-Indian Agricultural Statistics Research Institute, PUSA, New Delhi 110012, India; samarendra.das@louisville.edu; 2 Biostatistics and Bioinformatics Facility, JG Brown Cancer Center, University of Louisville, Louisville, KY 40202, USA; 3 School of Interdisciplinary and Graduate Studies, University of Louisville, Louisville, KY 40292, USA; 4 Centre for Agricultural Bioinformatics, ICAR-Indian Agricultural Statistics Research Institute, PUSA, New Delhi 110012, India; anil.rai@icar.gov.in; 5 Department of Medicine, School of Medicine, University of Louisville, Louisville, KY 40202, USA; michael.merchant@louisville.edu; 6 Hepatobiology and Toxicology Center, University of Louisville, Louisville, KY 40202, USA; 7 Biostatistics and Informatics Facility, Center for Integrative Environmental Health Sciences, University of Louisville, Louisville, KY 40202, USA; matt.cave@louisville.edu; 8 Christina Lee Brown Envirome Institute, University of Louisville, Louisville, KY 40202, USA; 9 Department of Bioinformatics and Biostatistics, School of Public Health and Information Science, University of Louisville, Louisville, KY 40202, USA

**Keywords:** scRNA-seq, differential expression, statistical models, multiple criteria decision making, combined data settings, TOPSIS

## Abstract

Single-cell RNA-sequencing (scRNA-seq) is a recent high-throughput sequencing technique for studying gene expressions at the cell level. Differential Expression (DE) analysis is a major downstream analysis of scRNA-seq data. DE analysis the in presence of noises from different sources remains a key challenge in scRNA-seq. Earlier practices for addressing this involved borrowing methods from bulk RNA-seq, which are based on non-zero differences in average expressions of genes across cell populations. Later, several methods specifically designed for scRNA-seq were developed. To provide guidance on choosing an appropriate tool or developing a new one, it is necessary to comprehensively study the performance of DE analysis methods. Here, we provide a review and classification of different DE approaches adapted from bulk RNA-seq practice as well as those specifically designed for scRNA-seq. We also evaluate the performance of 19 widely used methods in terms of 13 performance metrics on 11 real scRNA-seq datasets. Our findings suggest that some bulk RNA-seq methods are quite competitive with the single-cell methods and their performance depends on the underlying models, DE test statistic(s), and data characteristics. Further, it is difficult to obtain the method which will be best-performing globally through individual performance criterion. However, the multi-criteria and combined-data analysis indicates that DECENT and EBSeq are the best options for DE analysis. The results also reveal the similarities among the tested methods in terms of detecting common DE genes. Our evaluation provides proper guidelines for selecting the proper tool which performs best under particular experimental settings in the context of the scRNA-seq.

## 1. Introduction

The bulk-cell RNA-sequencing (RNA-seq) technique measures the aggregated expression levels of thousand(s) of genes from tissue samples, i.e., a collection of thousand(s) of cells. This technology cannot capture cell-cell heterogeneity since there is no cell-specific information available [1,2]. Hence, the single-cell RNA-sequencing (scRNA-seq) technique was developed for studying the expression dynamics of genes at the single-cell level [3]. Through RNA-seq, the expression is quantified by mapping reads to a reference genome, followed by counting the number of reads mapped to each gene [4]. In scRNA-seq, individual transcript molecules are attached with Unique Molecular Identifier (UMI) tags and, subsequently, counting the UMIs yields the number of transcripts for each gene in a cell [5]. Moreover, the scRNA-seq data have unique features, such as low library sizes of cells, stochasticity of gene expression, high-level noises, low capturing of mRNA molecules, high dropouts, amplification bias, multi-modality, zero-inflation, etc. The addition of UMIs during library preparation step reduces the inherent amplification bias [6] but has no effect on the noises. The noises in the scRNA-seq data are mainly due to biological and technical factors. These biological and technical factors contribute higher proportions of zeros in the data, characterized as true and false/dropout zeros, respectively [7,8,9].

The most commonly performed downstream analysis on scRNA-seq data is Differential Expression (DE) analysis. Such analysis is necessary for the identification of gene markers for different cell types, which establishes the molecular basis for phenotypic variation [10]. Further, the detected genes can be used as input for other secondary analyses, such as gene network modeling, pathways, or gene set analysis [11]. Although DE analysis methods for bulk RNA-seq are well reported, these approaches may not be suitable for single-cell data, given the special features [12]. For instance, bulk RNA-seq methods, such as edgeR [13] and DESeq2 [14,15] (based on a negative binomial (NB) model), are used for the analysis of scRNA-seq data. The utility of such tools may raise serious concerns about their validity due to higher dropouts [12], transcriptional bursting [16], lower molecular capturing in cells [17,18], higher dispersion [19], etc.

Single-cell specific DE methods have been developed in recent years, which use different sets of strategies to cope with the above concerns [11,12,17,18,20,21,22]. For instance, SCDE uses a mixture model (i.e., Poisson for dropout and NB for amplification) to capture the observed abundance of a given transcript in each cell [23]. Many DE methods and tools are available in the literature, which greatly vary from each other with respect to distributional assumptions of the data, DE test statistic(s), etc. [1,2,10,24,25,26]. Hence, it is pertinent to review and comprehensively study the available approaches and tools in order to understand their background statistical theory, unique features, and their limitations. Without sufficient understanding of the underlying statistical principles of these approaches, we may risk drawing erroneous biological interpretations and statistical conclusions.

We, therefore, aim to present a comprehensive survey of the up-to-date statistical methods for DE analysis of scRNA-seq data. There are many methodologies that have been developed for bulk RNA-seq, collectively named bulk RNA-seq DE methods, which have been extended to single-cell data analysis. Overall, the purpose of these methods is to analyze the data to provide an expansive view of the underlying biological processes, which lead to phenotypic differences. The paper is organized as follows. In the first part, we provide an overview of the DE analysis approaches that can be adapted from bulk RNA-seq practices to fit scRNA-seq data, as well as those specifically designed for scRNA-seq. While there are plenty of DE approaches, they can be distinguished based on the type of distributional model they use to fit the data. Subsequently, we also classify the available approaches into different classes, along with their special features and limitations.

In the second part of the paper, we attempt to provide a meaningful comparison of several statistical approaches; these are intrinsically different in terms of the model they fit. This includes 19 methods, such as DEGseq [27], edgeRLRT [13], edgeRQLF [13], DESeqLRT [15], DESeqNB [14], LIMMA [28], NBPSeq [29], EBSeq [30], BPSC [31], MAST [11], Monocle [22], scDD [32], NODES [33], DEsingle [20], DECENT [18], T-test [34], Wilcoxon rank sum test (Wilcox) [35], ROTS [36], and EMDomics [37] (Supplementary Figure S1). Among those, the first eight methods were designed for bulk RNA-seq, the next seven methods were developed for single-cell, and the remaining methods are general purpose. We compare these methods based on different criteria, such as Area under Receiver Operating Characteristics (AUROC) curve, False Discovery Rate (FDR), 10 other performance metrics, and runtime, on multiple real single-cell datasets. Not surprisingly, the performance of various methods depends on the data characteristics, the statistical models they fit, and the DE test statistic they use. The findings indicate that some bulk RNA-seq DE methods are quite competitive with some of the single-cell specific methods. Additionally, we also assess the performance of the methods under Multiple Criteria Decision Making (MCDM) and combined-data setups, which indicated that DECENT, followed by EBSeq are the best options for the DE analysis of scRNA-seq data. The similarity analysis of the methods revealed that there exists similarities among the tested methods in terms of detecting common DE genes. These findings were unknown before. Hence, our evaluation provides a guideline for selecting the proper DE tool which performs best under particular experimental settings in the context of scRNA-seq.

## 2. Overview and Classification of scRNA-seq DE Methods

**Figure 1:**
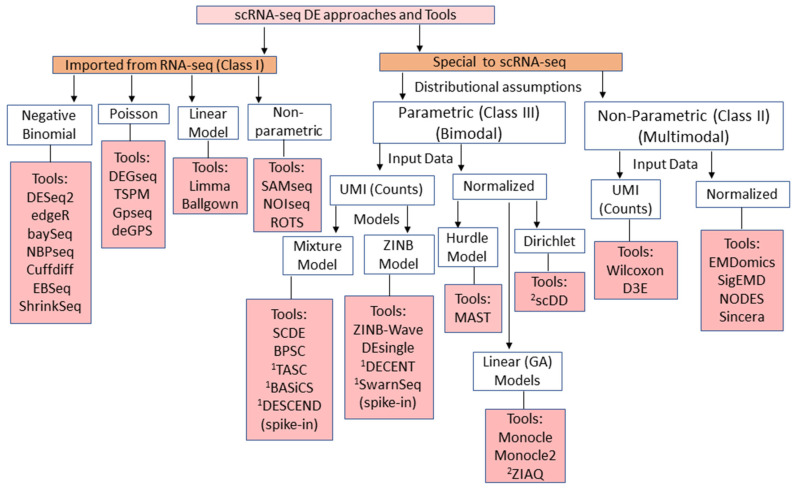
Schematic Representation of Classification of scRNA-seq DE Methods and Tools. Schematic overview illustrating the breakup of the methods that can be adapted from the RNA-seq practice to fit scRNA-seq data (Class I) as well as those specifically designed for single-cell (Classes II, III) based on the different distribution models that they fit. Different example tools belonging to each category are listed in pink color boxes.^1^ Methods use the external RNA spike-ins and ^2^ Parametric approaches but can handle multi-modality of the data.

The available DE analysis approaches used in single-cell data analytics are listed in Table 1. Table 1 also presents a comparative overview of the methods in terms of distributional assumptions, original data motivation (utility), input data type, test statistic(s), runtime, and their availability platform. Instead of reviewing them individually, we classified these methods based on different factors, as shown in Figure 1. The available methods can be classified based on origin, i.e., methods originally developed for bulk RNA-seq but later extended to scRNA-seq and methods exclusively designed for single-cell (Figure 1). Further, the bulk RNA-seq methods can be classified into parametric and non-parametric (NP) classes (Figure 1). The former class assumes that the data follow certain count data models, while the latter is distribution-free. For instance, the parametric class methods mostly assume the read counts are obtained from Poisson or NB distribution and based on software packages, such as edgeR [13], DESeq2 [14,15], BaySeq [38], DEGseq [27], and TSPM [39], have been developed. However, the NP methods estimate the parameters that can quantify the distribution of expression profiles and make comparisons between case vs. control groups. The tools for this category include SAMseq [40], TOISeq, and ROTS [36,41], to name a few, which were developed for bulk RNA-seq, but later extended to scRNA-seq (Figure 1). The bulk RNA-seq DE methods suffer from serious limitations, as listed in Table 2, when they are extended to the scRNA-seq data.

Similarly, the single-cell methods can also be grouped into parametric and NP classes based on the distributional assumptions of the UMI counts. The parametric methods assume that the UMI data follow count models, such as Zero Inflated Models (ZIM) (Zero Inflated NB (ZINB), Zero Inflated Poisson (ZIP)), and Mixture Models (Beta-Poisson, Poisson-NB, NB-Logistic, and Hurdle models). The R packages, such as SwarnSeq, DEsingle, DECENT, ZINB-wave, BPSC, SCDE, and MAST, belong to this category. Furthermore, the NP methods are implemented in software packages, such as D3E [47], sigEMD [51], Sincera [49], NODES [33], and EMDomics [37]. These approaches can handle the multi-modality of scRNA-seq data but are limited to only two group comparisons. The special features, pros, and limitations for the various classes of methods are listed in Table 2.

Classification of the DE methods can be made based on the nature of the input data to the concerned tools, such as discrete/counts (UMI) or continuous (fragments per kilo-base per million reads (FPKM) or, counts-per-million reads (CPM) or, normalized data). The methods which are specialized to handle the UMI counts include DEsingle, DECENT, SwarnSeq, DESCEND, and ZINB-wave, to name a few. Other methods, based on continuous data or that transform the original UMI counts include scDD, MAST, Monocle2, EMDomics, and ROTS. (Table 1). However, such methods ignore the counts nature of UMI data and, subsequently, there is a chance of losing information due to data transformation. Another classification of methods may be possible through the type of test statistic(s) used for DE testing. This includes methods, such as DESeq2, edgeR, DECENT, BPSC, MAST, and Monocle2, that based on the Likelihood Ratio Test (LRT) statistic computed in a Generalized Linear Model (GLM) framework. The other class of methods, including D3E, NODES, EMDomics, Sincera, and sigEMD, computes the test statistic(s) from the NP testing procedure. In addition, there is an important class of method found in the literature which integrates (cell) molecular capture process in the UMI counts modeling. This includes TASC [65], BASiCs [56], DECENT [18], SwarnSeq [54,55] and DESCEND [17]. This class of methods explicitly considers technical variation and molecular capturing processes based on external spike-ins data, while fitting the models [17,18,54,56,65].

## 3. Real scRNA-seq Datasets

To assess the performance of the DE methods, we used real scRNA-seq datasets publicly available in Gene Expression Omnibus (GEO) database of NCBI (https://www.ncbi.nlm.nih.gov/geo) (assessed on 10 October 2020). In our comparative analysis, we included 11 UMI count datasets derived from nine independent single-cell studies. The main rationale behind selecting these count expression datasets is that they are quality checked and preprocessed by the authors of these original publications. The selected datasets include scRNA-seq data from lung cancer cells, pluripotent stem cells, liver cells, adipose stem/stromal cells, kidney cells, breast cancer cells from humans, embryonic stem (ES) cells, blood cells, mouse embryonic fibroblasts (MEF) cells, and cells from mice. The brief and detail description of the selected datasets are given in Table 3 and Supplementary Document S1, respectively. For instance, Islam data have UMI counts of 22,928 genes over 92 cells (48: ES cells; 44: MEF cells), available at the GEO database with accession GSE29087. In order to reduce the dimensions of data, we then filtered out the genes with a lower expression, i.e., genes which do not have non-zero expressions in at least five cells (Supplementary Table S1). Further, the reference genes for the same cell lines were collected from the Microarray study, available at http://carlosibanezlab.se//Data/Moliner_CELfiles.zip [66], to assess the performance of the methods. The technique of performance evaluation of methods through obtaining reference genes from parallel studies, including Microarrays [2] and RNA-seq [18,54], is well reported in the literature. Similar descriptions about other real datasets, including Tung data [6], Soumillon1 data [67], Soumillon2 data [67], Soumillon3 data [67], Klein data [68], Gierahn data [69], Chen data [57], Savas data [70], Grun data [71], and Ziegenhain data [72], are given in the Supplementary Document S1. Moreover, data processing and selection of reference genes for each of the datasets is also described in Supplementary Document S2.

## 4. Methods for scRNA-seq DE Analysis

The notations are as follows: $Y_{ij}$: random variable (*rv*) represents observed UMI counts of *i^th^* (*i* = 1, 2, …, *N*) gene in *j^th^* (*j* = 1, 2, …, *M*) cell; *N*: total number of genes; *M*: total number of cells; $\mu_{ij}$: mean of *i^th^* gene in *j^th^* cell for NB distribution (count part); $\varphi_{ij}$ and $\theta_{ij}$ $(=\varphi_{ij}^{-1}$): dispersion and size parameters respectively for *i^th^* gene in *j^th^* cell for NB distribution; $\pi_{ij}\left(\in \left[0,\;1\right]\right)$: mixture probability (zero inflation probability) of *i^th^* gene in *j^th^* cell; $s_{j}$: library size of *j^th^* cell; $Z_{ij}$: *rv* represents the true (unknown) counts of UMI of *i^th^* gene in *j^th^* cell; X: design matrix for cell group information, whose *j^th^* row: $X_{j}=\left[X_{j1},\;X_{j2},\ldots ,\;X_{jN}\right]$; and $W_{ij}$: indicator *rv* representing rate of expression of *i^th^* gene in *j^th^* cell, i.e., $W_{ij}=0:\;Y_{ij}=0;\;W_{ij}=1:\;Y_{ij}>0$.

### 4.1. Negative Binomial Model Based Methods

The Probability Mass Function (PMF) of the NB distribution is expressed as:

(1) $$f_{NB}\left(y\right)=P\left[Y_{ij}=y\right]=\frac{G\left(y+\theta_{ij}\right)}{G\left(y+1\right)G\left(\theta_{ij}\right)}{\left(\frac{\theta_{ij}}{\theta_{ij}+\mu_{ij}}\right)}^{\theta_{ij}}{\left(\frac{\mu_{ij}}{\theta_{ij}+\mu_{ij}}\right)}^{y}\;\forall \;y=0,\;1,\;2,\;\ldots$$

where, $\mu_{ij}\geq 0;\;\theta_{ij}>0$ are the parameters of NB distribution, *G*(.): Gamma function. Following on from this, the expected value and variance of $Y_{ij}$ is shown as:

(2) $$E\left(Y_{ij}\right)=\mu_{ij}$$

(3) $$V\left(Y_{ij}\right)=\mu_{ij}+\frac{\mu_{ij}{}^{2}}{\theta_{ij}}=\mu_{ij}+\varphi_{ij}\mu_{ij}{}^{2}$$

$$\mathrm{If}\;\varphi_{ij}\to 0\;\left(No\;dispersion\right)\;\dot{\Rightarrow }NB\left(\mu_{ij},\theta_{ij}\right)\;\to Poisson\left(\mu_{ij}\right).$$

#### 4.1.1. DESeq

DESeq [14] assumes that $Y_{ij}$ follows the NB model (Equation (1)). In other words, the read counts are modeled by the NB distribution with $\mu_{ij}$ and $V\left(Y_{ij}\right)$ estimated from the scRNA-seq data. For each gene, the $\mu_{ij}$ (Equation (2)) is expressed as the product of $E\left(Z_{ij}\right)$ and $s_{j}$. Further, $Y_{ij}$ can be modeled within the NB based GLM framework through the following expressions:

(4) $$\;Y_{ij}\sim NB\left(\mu_{ij},\theta_{ij}\right)$$

(5) $$\mu_{ij}=s_{j}E\left(Z_{ij}\right)$$

(6) $$\log_{2}E\left(Z_{ij}\right)=\beta_{0i}+\beta_{1i}X_{j}$$

where, $X_{j}$: binary indicator for cellular group, $\beta_{0i}$: logarithm of mean parameter for *i^th^* gene in the reference cell group, $\beta_{1i}$: log fold-change parameter for *i^th^* gene. DESeq first estimates the size factors that account for the differences in the library size, then estimates the dispersion, and lastly, fits a GLM for each gene. The DESeq uses various test statistic(s) to compute the *p*-value and size effect estimate for the log2 fold change. Here, we used DESeq method based on LRT and NB test statistic(s) through executing *nbinomTest*, and *DESeq* functions respectively implemented in DESeq2 R package [15].

#### 4.1.2. edgeR

Like DESeq, edgeR [13] also models $Y_{ij}$ using NB distribution (Equation (1)). For each gene, $\mu_{ij}$ is assumed to be product of $s_{j}$ and $Z_{ij}$ in the current experimental condition. Here, $V\left(Y_{ij}\right)$ is a function of $\mu_{ij}$ (Equation (3)) and its computation requires the estimation of $\varphi_{ij}$. As such, edgeR estimates $\varphi_{ij}$ using a Maximum Likelihood Estimation (MLE) procedure conditioned on the total read count of each gene and an empirical Bayes procedure to shrink the dispersions toward a consensus value [73]. For each gene, the DE test statistic(s) are computed through the GLM based LRT [74] or Quasi-Likelihood F-test (QLF). Here, we used the edgeR method based on LRT, and QLF test statistic(s), i.e., edgeRLRT and edgeRQLF through executing *glmLRT* and *glmQLFTest*, implemented in the edgeR R package [13].

#### 4.1.3. NBPSeq

The NBPSeq [29] (or NBSeq) method was originally developed for the DE analysis of RNA-seq data, which assumes that the read counts follow an NB distribution. The DE testing procedure is based on parameterization of the NB distribution and uses the extended version of the exact test proposed by Robinson and Smyth (2007) [73]. Through this test, the constant $\varphi_{ij}$ is used to model the count variability between biological replicates and introduces an additional parameter to allow the $\varphi_{ij}$ to depend on $\mu_{ij}$. To implement the NBSeq method, we executed the *nbp.test* function implemented in the NBPSeq R package [29].

#### 4.1.4. EBSeq

EBSeq [30] assumes that the true (unknown) read counts follow the NB model and uses a Beta prior distribution to model the fluctuations in technical and biological variations. For RNA-seq data with two biological conditions, EBSeq tests the hypothesis, $H_{0}:\;\mu_{i1}=\mu_{i2}$, using Bayesian approaches through incorporating prior probability of the DE of counts (modeled by mixture distribution). Here, the means and variances of genes are obtained through the method-of-moments, and the global hyperparameters are computed using the Expected Maximization (EM) algorithm. With these parameter estimates, the posterior probability of the DE of genes are obtained using Bayes’ rule and, subsequently, DE genes are detected. To execute this method, the *EBTest* function implemented in EBSeq R package [30] was used.

### 4.2. Poisson Model

#### DEGseq

DEGSeq [27] assumes that read counts follow a Poisson Distribution (PD) model (Supplementary Document S3) [75]. The model parameters were estimated using the MLE method by maximizing concave joint likelihood function [75]. Further, with the estimates of PD parameters, the DE genes are identified through Fisher’s exact and LRT test statistic(s) [75]. Here, we used the LRT statistic to detect DE genes in the data through executing the *DEGexp* function implemented in the DEGSeq R package [27].

### 4.3. Zero Inflated Negative Binomial Model

The PMF of the ZINB distribution used to model the UMI counts is given in Equation (7).

(7) $$P\left[Y_{ij}=y\right]=\{\begin{array}{c} \pi_{ij}+\left(1-\pi_{ij}\right){\left(\frac{\theta_{ij}}{\theta_{ij}+\mu_{ij}}\right)}^{\theta_{ijk}}\;when\;y=0\\ \left(1-\pi_{ijk}\right)\frac{G\left(y+\theta_{ij}\right)}{G\left(y+1\right)G\left(\theta_{ij}\right)}{\left(\frac{\theta_{ij}}{\theta_{ij}+\mu_{ij}}\right)}^{\theta_{ijk}}{\left(\frac{\mu_{ij}}{\theta_{ij}+\mu_{ij}}\right)}^{y};\;y>0 \end{array}$$

For, $Y_{ij}\sim ZINB\left(\pi_{ij},\mu_{ij},\theta_{ij}\right),$ the expected value and variance of $Y_{ij}$ can be obtained as (Supplementary Document S3):

(8) $$E\left(Y_{ij}\right)=\left(1-\pi_{ij}\right)\mu_{ij}$$

(9) $$V\left(Y_{ij}\right)=\left(1-\pi_{ij}\right)\mu_{ij}\left(1+\pi_{ij}\mu_{ij}+\frac{\mu_{ij}}{\theta_{ij}}\right)$$

$$\mathrm{If}\;\pi_{ij}=0\dot{\Rightarrow }ZINB\left(\pi_{ij},\mu_{ij},\theta_{ij}\right)\to NB\left(\mu_{ij},\theta_{ij}\right).$$

$$\mathrm{If}\;\varphi_{ij}\to 0\;\left(No\;dispersion\right)\dot{\Rightarrow }\;ZINB\left(\pi_{ij},\mu_{ij},\theta_{ij}\right)\;\to ZIP\left(\pi_{ij},\mu_{ij}\right).$$

#### 4.3.1. DEsingle

DEsingle [20] is a ZIM based approach that employs the ZINB distribution (Equation (7)) to discriminate the observed zeros into two parts: dropout and true zeros. Under this model formulation, DEsingle is designed to overcome the issues of excessive zeros that are observed in the scRNA-seq data. To detect DE genes between two cell groups, DEsingle first estimates the parameters of two ZINB populations (Equation (7)) (for two cell groups). It then detects the DE genes using the LRT statistic through the constrained MLE of the two models’ parameters under null hypothesis. Here, the *p*-values for genes were computed through executing the *DEsingle* function that is implemented in the DEsingle R package [20].

#### 4.3.2. DECENT

DECENT [18] is based on ZIM, and precisely uses the ZINB model (Equation (7)) for fitting scRNA-seq data. It also explicitly and accurately models the molecular capture process using a Beta-Binomial model. Here, $Z_{ij}$ and the cellular capture process are assumed to follow the ZINB and Binomial models, respectively. Mathematically, DECENT assumes the following models for different processes.

(10) $$Z_{ij};\pi_{ij},s_{j},\mu_{ij},\theta_{ij}\;\sim \;ZINB\left(\pi_{ij},s_{j}\mu_{ij},\theta_{ij}\right)$$

(11) $$Y_{ij}|Z_{ij}=k;p_{ij\;}\sim \;B\left(k,\;p_{ij}\right)$$

(12) $$p_{ij}\;\sim \;Beta\left(a_{ij},\;b_{ij}\right)$$

where, $p_{ij}$ is transcriptional capture rate for *i^th^* gene in *j^th^* cell, *B*(.): Binomial distribution, $a_{ij},\;and\;b_{ij}$ in Equation (12) are the parameters of Beta distribution. DECENT uses the Expected Conditional Maximization (ECM) algorithm to calculate the MLE of the ZINB model parameters (Equation (10)) using the observed data through integrating the molecular capturing procedure in the presence of external RNA-spike ins. To detect DE genes, DECENT uses the GLM framework (Equation (13)) to model the $\mu_{ij}$.

(13) $$\log\mu_{ij}=\beta_{0i}+\beta_{1i}X_{j}+\tau_{i}^{\prime }U_{j}$$

where, $\beta_{0i},\;\;\beta_{1i},\;X_{j}$ has the usual meaning as in Equation (6) and $\tau_{i}$: regression coefficient of *i^th^* gene for *j^th^* cell-level auxiliary is $U_{j}.$ The *p*-values for each gene are computed through LRT statistic under the GLM (Equation (13)), which is executed through *decent* function implemented in DECENT R package [18].

### 4.4. Mixed Model Based Methods

#### 4.4.1. BPSC

BPSC [31] is an analytical method based on the Beta-Poisson (BP) mixture model, designed to capture the distributional features of the scRNA-seq data, i.e., non-integer expression or low expression values. In BPSC, normalized data (Supplementary Document S4), such as FPKM or CPM, are fitted by using the four parameters BP model given in Equation (14).

(14) $$BP_{4}\left(Y_{ij}|\alpha ,\beta ,\vartheta_{1},\vartheta_{2}\right)=\vartheta_{2}P\left(Y_{ij}|\vartheta_{1}Beta\left(\alpha ,\beta \right)\right)$$

where, $Y_{ij}$: normalized value of the read counts; *P*(.): Poisson PMF; $\alpha ,\beta ,\vartheta_{1},\vartheta_{2}$ are the parameters of the BP model (Equation (14)). The expected value and variance of $Y_{ij}$ are expressed as:

(15) $$E\left(Y_{ij}\right)=\mu_{ij}=\vartheta_{1}\vartheta_{2}\frac{\alpha }{\alpha +\beta }$$

(16) $$V\left(Y_{ij}\right)=\mu_{ij}\vartheta_{2}+\mu_{ij}{}^{2}\frac{\beta }{\alpha \left(\alpha +\beta +1\right)}$$

The MLEs of the parameters in Equation (14) are estimated using the iterative weighted least-squares algorithm [31]. The DE analysis of the genes was carried out under the GLM framework given in Equation (6). Further, *p*-values for the genes are computed through the LRT statistic by executing the *BPglm* function implemented in the BPSC R package [31].

#### 4.4.2. scDD

The scDD [32] method, based on the Logistic-Dirichlet mixture model, is designed to model the scRNA-seq data under a Bayesian modeling framework. It models the excess zeros in scRNA-seq data using logistic regression and models the non-zero counts using conjugate Dirichlet model of normal distributions. Here, the UMI counts are transformed to CPM measures through the *cpm* function implemented in the edgeR R package [13] followed by log-transformation. scDD uses a Bayesian modeling approach to detect DE genes between the two cellular groups. For this purpose, it computes an approximate Bayes factor score that compares the probability of DE with the probability of non-DE for each gene. The empirical gene *p*-values for the DE tests are computed using a permutation method. To execute this method, we used the *scDD* function implemented in the scDD R package [32].

### 4.5. Normal/Gaussian Based Methods

#### 4.5.1. LIMMA

LIMMA [28,42], based on a linear model, was originally designed for Microarrays but was recently extended to bulk RNA-seq data. For expression counts, LIMMA uses Voom transformations [42] and then models the transformed expression values ($Y_{ij}^{v}$) using linear models. Alternatively, it considers gene-specific linear models to model $Y_{ij}^{v}$, and is given as:

(17) $$E\left(Y_{ij}^{v}\right)=X\omega_{i}$$

(18) $$Var\left(Y_{ij}^{v}\right)=L_{i}\sigma_{i}^{2}I$$

where, $\omega_{i}$: regression coefficient vector for *i^th^* gene, $L_{i}$: known weight matrix for *i^th^* gene, and $\sigma_{i}^{2}$: variance of *i^th^* gene. For performing DE analysis, the empirical Bayes approach was used by incorporating the expected value–variance relationship [28]. In this study, the *voom*, *lmFit*, and *eBayes* functions implemented in the limma R package were executed for data transformations, model fitting, and DE analysis, respectively.

#### 4.5.2. MAST

MAST [11] uses a hurdle model for DE analysis and assumes conditional independence between the expression rate ($W_{ij}$) and expression levels $\left(Y_{ij}\right)$ for the *i^th^* gene. MAST fits a logistic regression for $W_{ij}$ and a Gaussian linear model for the continuous variable ($Y_{ij}$|$W_{ij}$ = 1), which can be summarized as:

(19) $$logit\left[\mathrm{Pr}\left(W_{ij}=1\right)\right]=X_{j}\beta_{i}$$

(20) $$Pr(Y_{ij}=y|W_{ij}=1)=N\left(X_{j}\beta_{i},\;\sigma_{i}^{2}\right)$$

In order to improve the inference for genes with sparse expression, the model parameters are fitted using an empirical Bayesian framework [11]. Finally, DE testing for genes is performed across the two cellular groups through the LRT statistic(s). Here, we executed the *zlm*, and *summary* functions for hurdle model fitting and DE analysis, respectively, as implemented in the MAST R package [11].

#### 4.5.3. Monocle

Monocle [21,22] (updated as Monocle2 [22]), is a specially designed method for DE analysis, i.e., identifying DE genes that vary across different cell groups or pseudo-times in scRNA-seq data. This uses a generalized additive model (GAMs) to model $\mu_{ij}$ under the GLM framework, i.e., relating $\mu_{ij}$ to one or more predictors through GAMs for the *i^th^* gene and is expressed as:

(21) $$log\mu_{ij}=\beta_{0i}+f_{1}\left(x_{1}\right)+f_{2}\left(x_{2}\right)+\ldots +f_{M}\left(x_{M}\right)$$

where, $\beta_{0i}$: regression co-efficient; $x_{j}$: predictor variable that represents group memberships of *j^th^* cell; and $f_{j}\left(.\right)$: smoothing functions, e.g., cubic splines. Specifically, the expression values across the cells are modeled using a Tobit model (approximately); thus, Monocle’s GAM becomes:

(22) $$\mu_{ij}=s\left(\delta_{t}\left(b_{x},f_{j}\right)\right)+\varepsilon$$

where, $\delta_{t}\left(b_{x},f_{j}\right)$: pseudo-time or cell group of a cell; $f_{j}:$ cubic smoothing function (with three effective degrees of freedom), and ε: error term (follow standard normal distribution). Further, Monocle performs DE testing of genes across cell groups through LRT statistic(s) by comparing full GLM with additional effects to a reduced GLM based on the NB model. To implement this method, the *differentialGeneTest* function of the monocle R package [22] was executed.

#### 4.5.4. T-Test

T-test [34] is a general-purpose method, used to compare the mean expressions of genes across two cellular groups. Traditionally, scRNA-seq UMI data violates the T-test’s normality assumptions, and as such, we used the TMM method to normalize the data (Supplementary Document S4). The test statistic for the T-test is expressed as:

(23) $$t_{i}=\frac{{\bar{y}}_{i1}-{\bar{y}}_{i2}}{\sigma_{i}}$$

(24) $$\sigma_{i}=\sqrt{\frac{S_{i1}^{2}}{M_{1}}+\frac{S_{i2}^{2}}{M_{2}}}$$

where, ${\bar{y}}_{ik},S_{ik}^{2}$: mean and variance of the normalized expressions of *i^th^* gene for *k^th^* (*k* = 1,2) cell group, *M_k_*: number of cells in *k^th^* cell group. Empirically, scRNA-seq data are highly (positively) skewed, but the T-test is known to have a certain robustness against skewness. Therefore, it is worth comparing its performance against sophisticated bulk and single-cell methods. This method is executed through the *t.test* function implemented in the stats R package.

### 4.6. Non-Parametric Methods

#### 4.6.1. EMDomics

EMDomics [37] is a general-purpose NP method based on Earth Mover’s Distance (EMD), developed for the DE analysis of genomics data (testing difference in mean expressions of genes across two cell groups that are significantly different from zero).

Let, $P_{i}=\left\{\left(p_{i1},w_{p1}\right),\;\left(p_{i2},w_{p2}\right)\ldots ,\left(p_{iM_{1}},w_{pM_{1}}\right)\right\}$ and $Q_{i}=\left\{\left(q_{i1},w_{q1}\right),\;\left(q_{i2},w_{q2}\right)\ldots ,\left(q_{iM_{2}},w_{qM_{2}}\right)\right\}$ be the signatures of *i^th^* gene across two cell groups; $p_{ij}$ (*j* = 1, 2, …, *M*_1_) and $q_{ij^{\prime }}$ ($j^{\prime }$ = 1, 2, …, *M*_2_) are the centers of *j^th^* and $j^{\prime }$*^th^* histogram in two cell groups; and $w_{pj}$ and $w_{qj^{\prime }}$ are the weights for *j^th^* and $j^{\prime }$*^th^* cells in two groups. The EMD statistic for the *i^th^* gene is computed through Equation (25).

(25) $$EMD_{i}=\frac{\sum_{j=1}^{M_{1}}\sum_{j^{\prime }=1}^{M_{2}}f_{jj^{\prime }}^{i}d_{jj^{\prime }}^{i}}{\sum_{j=1}^{M_{1}}\sum_{j^{\prime }=1}^{M_{2}}f_{jj^{\prime }}^{i}}$$

where, $d_{jj^{\prime }}^{i}$: Euclidean distance between *j^th^* and $j^{\prime }$*^th^* cells across two groups for *i^th^* gene and $f_{jj^{\prime }}^{i}$: coefficient of flow from *j^th^* to $j^{\prime }$*^th^* cell for *i^th^* gene and obtained through minimizing the cost function in Equation (26).

(26) $$Cost^{i}\left(P,Q,\;F\right)=\sum_{j=1}^{M_{1}}\sum_{j^{\prime }=1}^{M_{2}}f_{jj^{\prime }}^{i}d_{jj^{\prime }}^{i}$$

The EMD test statistic reflects the overall difference between the two normalized distributions (for two cell groups), which is usually assessed through statistical significance using the permutation test. To implement this method, *calculate*_*emd* function of EMDomics R package [37] was executed.

#### 4.6.2. NODES

NODES [33] is an NP method used for detecting DE genes across two cell groups using normalized scRNA-seq data. NODES uses the Pseudo-Counted Quantile Normalization method to normalize the counts data [33]. It then performs DE analysis on the normalized data using the test statistic given in Equation (27).

(27) $$d_{i}=\frac{\left|{\bar{y}}_{i1}-{\bar{y}}_{i2}\right|}{a_{0}+\sigma_{i}}$$

where, $d_{i}:$ test statistic for *i^th^* gene, $a_{0}$: fixed percentile (e.g., 50^th^) of the standard errors $\left\{\sigma_{i};i=1,2,\ldots ,\;N\right\}$, and ${\bar{y}}_{i1},{\bar{y}}_{i2},\;\mathrm{and}\;\sigma_{i}$ are defined in Equation (23). NODES computes *p*-values for the genes through permutation test, which is implemented through executing the *NODES* function of the NODES R package [33].

#### 4.6.3. Wilcoxon Signed Rank Test (Wilcox)

The Wilcox method [35] (Mann –Whitney test) is an NP method used to test whether a genes’ mean expressions across the two cell groups is significantly different or not. The test’s main idea is to compare the ranks of the expression values that come from the two cell groups. This rank-based test mostly ignores the magnitude of the expression of deviations of genes between the two cell groups, but may be a potential method in comparison to others. To execute this method, we used the *wilcox*.*test* function implemented in the stats R package.

#### 4.6.4. ROTS

Like the T-test, Wilcox, and EMDomics, ROTS [36] does not have any single-cell or sequencing-specific functions. It optimizes the parameters among a family of modified t-statistics (Equation (27)) by maximizing the detections’ reproducibility across bootstrap samples. In other words, ROTS maximizes the scaled reproducibility (Equation (28)) over the parameters, $\alpha =\left(\alpha_{1},\alpha_{2}\right);\;\alpha_{1}\epsilon \left[0,\infty \right),\;\alpha_{2}\epsilon \left\{0,1\right\}$, and *k* (>0).

(28) $$\frac{R_{k}\left(d_{i\alpha }\right)-R_{k}^{0}\left(d_{i\alpha }\right)}{S_{k}\left(d_{\alpha }\right)}$$

where, $S_{k}\left(d_{i\alpha }\right)$: estimated the standard deviation of bootstrap distribution of observed reproducibility for the *i^th^* gene, $R_{k}\left(d_{i\alpha }\right)$ and $R_{k}^{0}\left(d_{i\alpha }\right)$: reproducibility for observed and random data respectively. The observed reproducibility is calculated as the average of the reproducibilities over randomized data sets, which are permuted from the real samples. The observed average reproducibility is defined in Equation (29).

(29) $$R_{k}\left(d_{i\alpha }\right)=\frac{1}{B}\overset{B}{\underset{b=1}{\sum }}R_{k}^{\left(b\right)}\left(d_{i\alpha }\right)$$

where, *B*: number of bootstrap samples, and $d_{i\alpha }$: test statistic defined in Equation (27). This method was executed through *ROTS* function implemented in the ROTS R package [36].

## 5. Comparative Performance Evaluation

### 5.1. Performance Metrics

We evaluated the performance of the 19 tested methods (Supplementary Document S5) for identifying genuine DE genes through individual performance metrics, such as True Positives (TP), False Positives (FP), True Negatives (TN), False Negatives (FN), True Positive Rate (TPR), False Positive Rate (FPR), FDR, Positive Prediction Rate (PPV), Negative Prediction value (NPV), Accuracy (ACC), F1 score (F1), and AUROC (Equations (30)–(37)), and runtime criteria on 11 real datasets (Table 3). The availability of the 19 methods and their parameter settings, used in this study, are provided in Supplementary Tables S2 and S3 respectively. The layout of this comparative study is shown in Supplementary Figure S1. Further, the performance metrics (Equations (30)–(37)) were computed by comparing the DE genes obtained through each method with the reference genes (Supplementary Document S2) for each dataset. For instance, we defined TP in Equation (30) as the selected DE genes which are matched with the reference genes and FP in Equation (31) as the genes those were found to be significant but were not reference DE genes. Similarly, TN in Equation (31) were defined as genes that were not reference DE genes and were not found to be significant, and FN in Equation (30) were defined as genes that were reference DE genes but were not found to be significant.

(30) $$TPR=Sensitivity=\frac{TP}{TP+FN}$$

(31) $$FPR=1-Specificity=\frac{FP}{FP+TN}\;$$

(32) $$PPR=\frac{TP}{TP+FP}$$

(33) $$FDR=\frac{FP}{FP+TP}$$

(34) $$NPV=\frac{TN}{TN+FN}$$

(35) $$ACC=\frac{TP+TN}{TP+TN+FP+FN}$$

(36) $$F1=\frac{2TP}{2TP+FP+FN}$$

*AUROC* = Area under *Sensitivity* vs. (1-*Specificity*) curve (37)

The impact of the above-defined criteria on the performance of the methods is shown in Supplementary Table S4. For instance, a higher value of TP or TPR (“+” sign in Supplementary Table S4) indicates that the method performs better and *vice-versa*. Similar interpretations can be made for other criteria (Supplementary Table S4).

### 5.2. Performance Evaluation under Multiple Criteria Decision-Making setup

We emphasized the analysis of the 19 methods (Supplementary Table S2) under the simultaneous consideration of all 13 criteria (Supplementary Table S4). In operational research, such a performance evaluation setting is called a MCDM setup [76], where the main idea is to consider a set of criteria and choose the best performing method from a list of methods [77]. Under this MCDM set up, the Technique for Order Performance by Similarity to Ideal Solution (TOPSIS) [78] has been used extensively [79]. However, we used the TOPSIS technique for the first time in single-cell data analytics to choose the best method under simultaneous consideration of the 13 decision criteria (Supplementary Table S4). The major analytical steps of the MCDM-TOPSIS analysis are given in Supplementary Document S6. Further, the TOPSIS technique was also used to analyze the methods under the combined-data setup. Through this, the methods with a higher *R_r_* (*R_r_*: TOPSIS score and 0 ≤ *R_r_* ≤ 1) (Supplementary Document S6) are preferred and are considered to be better over the multiple criteria (Supplementary Table S4) and *vice-versa*.

## 6. Results and Discussion

### 6.1. Count Models for Fitting of scRNA-seq Data

The scRNA-seq UMI data have overdispersion and an excess amount of zeros as the expression of genes due to higher dropout events, which is well established in the literature [11,18,19]. Here, we also showed that the statistical tests for zero-inflation and overdispersion are significant for most of the genes for experimental single-cell data (Supplementary Documents S7 and S8). In DE analysis, the existing methods assume certain count models for fitting the underlying data. To test the fitness of such models to scRNA-seq count data, we considered five count models, such as NB, ZINB, PD, Hermite Distribution (HD), and ZIP [80,81] (Supplementary Document S3). The descriptions of the considered datasets and obtained results are given in Supplementary Document S9. Our preliminary analytical results indicated that the expected frequencies computed from the ZINB model were much closer to their observed counterparts, followed by the NB model compared to others (Supplementary Tables S5 and S6, and Figure S2). At this stage, we inferred that the ZINB and NB models are best suited for fitting the scRNA-seq count data.

To be more specific, we also tested the NB and ZINB models’ ability to estimate the mean and dispersion parameters of genes through simulation. The detailed procedure of simulation and corresponding analysis is described in Supplementary Document S10. Our analytical results indicate that the NB model underestimated the mean and overestimated the dispersion (Supplementary Table S7). Further, the ZINB model provided better estimates of mean and dispersion parameters with lower bias and standard error as compared to NB model. The findings indicated better suitability of the ZINB for modeling the zero-inflated and overdispersed (scRNA-seq) count data (Supplementary Tables S5–S7).

### 6.2. Comparative Performance Analysis of scRNA-seq DE Methods

We compared the performance of the 19 methods for detecting true DE genes on 11 real scRNA-seq datasets under the condition of comparing two groups of cells. However, real studies involve more complex experimental designs, which some of the tested methods do not accommodate. Specifically, the T-test, Wilcox, ROTS, DEsingle, scDD, and NODES are limited to two-group comparisons, whereas the remaining methods can be generalized for multi-group comparisons. To make the comparisons fully reproducible, we provide R-codes, processed scRNA-seq datasets, and reference genes in https://github.com/sam-uofl/RoopSeq.

#### 6.2.1. Comparative Assessment Based on Performance Metrics

**Figure 2:**
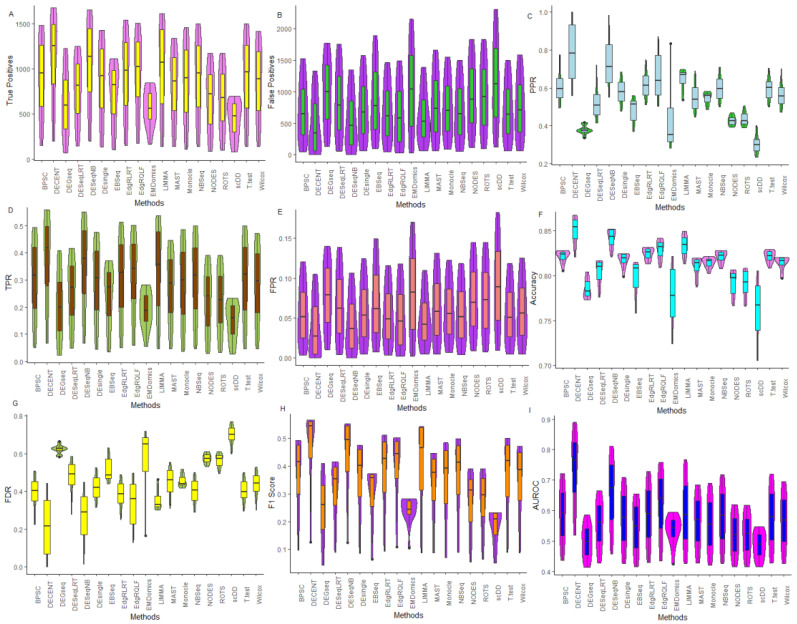
Comparative evaluation of DE methods through performance metrics for Soumillion2 data. The tested DE methods are evaluated on Soumillion2 data through the performance evaluation metrics, such as TP, FP, TPR, FPR, PPR, FDR, Accuracy, F1-score, and AUROC. The 19 tested methods are shown in X-axis. The violin plots are shown for the comparative evaluation of tested methods through (**A**) TP; (**B**) FP; (**C**) PPR; (**D**) TPR; (**E**) FPR; (**F**) Accuracy; (**G**) FDR; (**H**) F1-score; and (**I**) AUROC. The violin plot shows full distribution of performance metrics computed for each tested method. The box represents the inter-quartile range; the horizontal line represents the median, and the bars on the boxes show 1.5x inter-quartile range.

The single-cell datasets and their respective comparison designs were used to detect the DE genes through each of the 19 tested methods (Supplementary Table S2). For instance, in Islam data [5], the experimental design involves DE analysis of genes between 48 mouse ES cells and 44 MEF cells (Supplementary Document S1). In other words, we selected the DE gene sets of sizes 200, 400, …, 3000 through the tested methods from the Islam data (Table 3). The performance metrics, such as TP, FP, PPR, TPR, FPR, ACC, and F1, were then computed by comparing the detected DE genes with the reference genes for each dataset (Supplementary Document S2), and the results are shown in Figure 2, Figures S3–S12, Table 4, and Tables S8–S18.

In this comparison setting, for Soumillon2 data, the DECENT provided the highest TP values, followed by DESeqNB, LIMMA, and edgeRQLF (Figure 2A). Similar findings were observed when assessed through TPR (Figure 2D). Further, we found the lowest values of FP and FPR for these methods compared to others (Figure 2B). For instance, for the DE gene set of size 3000, the DECENT detected 1674 genes as truly DE, followed by DESeqNB (1653) and LIMMA (1612) (Table 4). In other words, DECENT detected fewer FP genes with higher probabilities along with DESeqNB, LIMMA, and edgeRQLF as compared to others. The accuracy-based performance analysis of the tested methods indicated that the DECENT was found to detect true (both positive and negative) genes more accurately, followed by DESeqNB and edgeRQLF compared to others (Figure 2C). Among the tested methods, EMDomics and scDD were found to have lowest rates of sensitivities and specificities for detecting the true DE genes, therefore they performed poorly for the Soumillon2 data (Figure 2). Similar interpretations can be made about the tested methods for this data through the PPR and F1-score (Figure 2, Supplementary Table S8).

For the Islam data, DECENT’s performance was found to be superior, followed by DEsingle and BPSC among the single-cell methods (Supplementary Figure S3, Table S9). Overall, the median TPR values for DESeqNB and edgeRQLF was found to be the highest, followed by edgeRLRT and DECENT (Supplementary Figure S3, Table S9). This observation indicated that these tested methods identified genes that are truly DE at higher rates (with lower FPR) compared to others. Further, the methods, including LIMMA, NODES, scDD, and DEGseq, were found to have poor performance in terms of higher FPR and lower TPR (Supplementary Figure S3). Similar interpretations can be made for all of the tested methods through other performance metrics, such as PPR, ACC, NPV, and F1 measures (Supplementary Figure S3, Table S9).

For other datasets, such as Tung, Chen, Savas, Soumillon1, Grun, Ziegenhain, Soumillon3, Gierahn, and Klein, similar interpretations can be made for the tested methods (Supplementary Figures S4–S12, Tables S10–S18). It can be observed that the performance of the tested methods varies differently across the datasets when assessed through individual performance metrics (Supplementary Table S19). For instance, EBSeq, edgeRQLF performed better for Tung data, while DECENT, EMDomics, provided better results for the Soumillon3 data. In other words, the tested methods’ performance was mostly data specific (no method was the best fit for all) when assessed through individual performance metrics. However, we found that the bulk RNA-seq methods are significantly more competitive (for some cases) than the single-cell methods under comparison of the two cellular groups (Supplementary Table S19). Similar findings were also obtained from Soneson and Robinson’s study [25].

#### 6.2.2. Performance Assessment Based on ROC

Under this comparison setting, performance of the DE methods was tested on 11 real datasets through AUROC, and the results are shown in Figure 2I, Supplementary Figures S3I–S12I, and Tables S8–S18. For Soumillon2 data, DECENT provided highest AUROC values, followed by DESeqNB, LIMMA, and edgeRQLF (Figure 2I), which indicated its superior performance over other methods. For instance, for the gene set size 3000, an AUROC value of 0.857 was observed for DECENT followed by DESeqNB (0.811), LIMMA (0.768), and edgeRQLF (0.758) (Table 4). In other words, the DECENT has higher sensitivity and specificity rates to detect true DE genes for Soumillon2 data compared to others. The single-cell specific tools scDD and MAST performed worst in this comparison, while DEGseq followed by EBSeq and DESeqLRT showed an overall poor performance among the bulk RNA-seq methods along with EMDomics (Figure 2I, Table 4 and Table S8).

For the Islam data, the DESeqNB method produced the highest AUROC value, followed by edgeRQLF, edgeRLRT, DECENT, and EMDomics (Supplementary Figure S3I, Table S9). The lowest AUROC values were observed for NODES, LIMMA, scDD, and DEGseq, with higher probabilities than others (Supplementary Figure S3I). The simple methods, such as the T-test, and Wilcox, performed relatively well with moderate sensitivities and specificities for detecting DE genes (Supplementary Figure S3I). Similar interpretations can be made for other nine datasets based on the AUROC (Supplementary Figures S4I–S12I, Tables S10–S18). Through sensitivity-specificity analysis, it was observed that the performance of the tested methods varies considerably across the real datasets (Supplementary Table S19). For instance, EMDomics and MAST performed very well for the Klein data, while LIMMA and EBSeq were better suited to the Chen data. However, scDD, NODES, and ROTS consistently performed worst across all of the datasets, while methods, such as DEGseq, DESeqLRT, and MAST, performed poorly for some of the datasets (Supplementary Table S20). Similar inferences can be made for the other datasets (Supplementary Tables S19 and S20). 

#### 6.2.3. Performance Assessment Based on FDR Rates

The results from the tested methods’ performance assessed through FDR across the 11 datasets are shown in Figure 2G, Figures S3G–12G, and Tables S8–S18. For Soumillon2 data, DECENT’s median FDR value was found to be the lowest, followed by DESeqNB and edgeRQLF (Figure 2G, Table 4 and Table S8). For instance, with a gene set of size 3000, the FDR value was observed to be 0.442 for DECENT and 0.448 for DESeqNB, whereas methods, including scDD, and EMDomics, provided the highest FDR values (Table 4). This indicates that the UMI-specialized DECENT tool’s performance was robust compared to other count data-based tools (Figure 2G). Further, normalized data-based tools, i.e., scDD, NODES, EMDomics, and ROTS, did not perform well in terms of robustness for detecting true DE genes.

For Islam data, the findings indicated that the performance of DESeqNB, edgeRQLF, edgeRLRT, and DECENT was observed to be robust among other competitive methods (Supplementary Figure S3G). Specifically, DECENT’s performance was better and more robust among the single-cell methods, followed by Monocle and MAST (Supplementary Table S9). However, bulk RNA-seq methods, such as DESeqNB, edgeRQLF, and edgeRLRT, performed better and were robust, even compared to single-cell methods for Islam data (Supplementary Figure S3G, Table S9). Further, among all methods, DEGseq, LIMMA, and NODES performed the worst in terms of robustness for this data. Similar interpretations can be made for other datasets through the computed FDR metric (Supplementary Figures S4G–S12G, and Tables S10–S18). Through such analysis, we observed that the tested methods’ performance varied across the real datasets for detecting robust DE genes (Supplementary Table S21). Hence, we can infer that there was not one single method that was found to be globally robust for DE analysis of scRNA-seq UMI data.

#### 6.2.4. Performance Assessment Based on Runtime

In single-cell data analytics, the computational processing speed for large-scale scRNA-seq data is an important factor. Hence, we evaluated the tested methods’ performance based on runtime criterion, where the runtime refers to the computational time required to analyze the data. Through this, the method which requires less runtime was considered to be better and *vice-versa*. To measure this, we ran the code written in R (*v* 4.0.2) [82] for each tested method by following instructions and recommendations of their respective R software packages. The required average CPU time (over 50 runs for each program) was observed for each of the methods for analyzing a UMI dataset with 10,000 genes and 500 cells. All of these analyses were performed on a 16 GB RAM computer with Windows 10 OS and Intel(R) Core (TM) i3-6100U CPU clock rate as 2.93 GHz. The details of the runtime-based performance analysis of the methods are given in Supplementary Document S11. Under this setting, it was found that DECENT is the slowest and most computationally intensive method, followed by DEsingle, due to the implementation of an iterative algorithm to estimate the model parameters (Supplementary Document S11). For instance, the UMI data (10,000 genes over 500 cells) in DECENT took ~20 h, followed by DEsingle (~12 h) to detect the DE genes (Supplementary Document S11). Among the methods, T-test, Wilcox, and LIMMA are the fastest to run; MAST, edgeR, and DESeq are also relatively fast (Supplementary Document S11). Further, various methods, such as EBSeq, ROTS, EMDomics, and NODES, are relatively time-consuming due to the implementation of resampling, i.e., permutation and bootstrap, procedures. The remaining methods do not include any heavily time-consuming steps, and therefore are considered as computationally efficient (Supplementary Document S11).

#### 6.2.5. Performance Assessment Based on MCDM-TOPSIS Analysis

**Figure 3:**
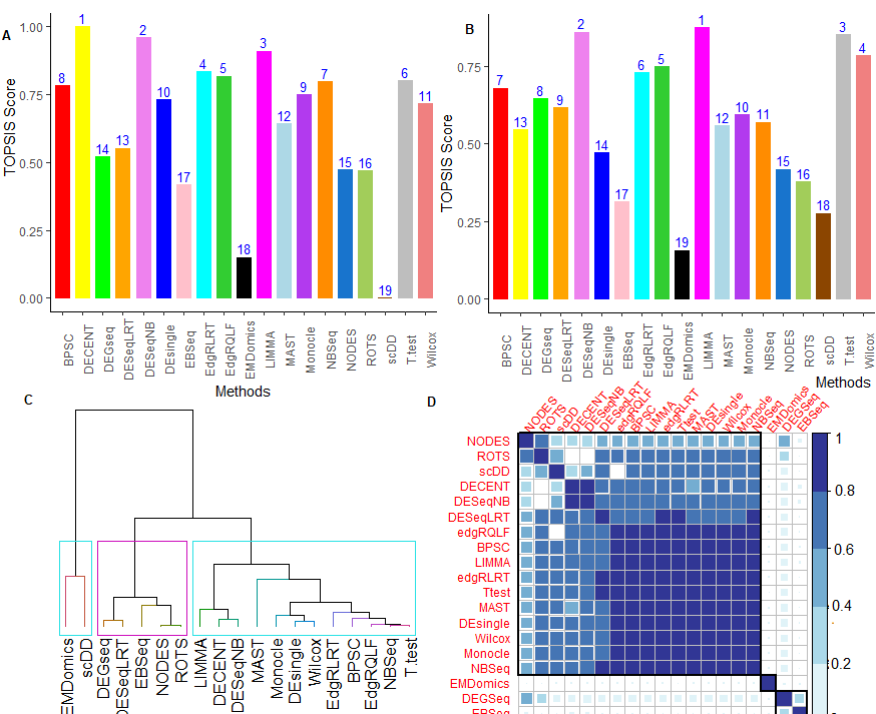
Performance evaluation of methods under MCDM setup for Soumillion2 data. Comparative performance analysis of 19 tested methods was carried out through the TOPSIS approach under MCDM setup on the Soumillion2 dataset. MCDM-TOPSIS analysis was carried out under (*i*) multi-criteria including runtime criterion; (*ii*) multi-criteria excluding runtime criterion. X-axis represents tested methods and Y-axis represents TOPSIS scores. The results from the (**A**) MCDM-TOPSIS analysis of the DE methods are shown for 12 performance metrics excluding runtime criterion; (**B**) MCDM-TOPSIS analysis of the methods based on 13 performance metrics including runtime criterion; (**C**) Average similarities between evaluated DE methods based on 13-performance metrics. The dendrogram was obtained by average-linkage hierarchical clustering based on matrix of average values of performance metrics over all gene sets; (**D**) Similarity analysis among the methods based on their ability to detect common DE genes. The *p*-values for each comparison were computed through the Binomial test (Supplementary Document S12). Significant proportions (at 1% level of significance) of common genes shared among the methods are shown in various colors and white empty cells represent non-significant values.

We observed conflicts among the 13 criteria (Supplementary Table S4) through which the tested methods’ performance was assessed (Supplementary Tables S22–S30). For instance, DECENT performed better among the methods based on most of the criteria but performed worst under runtime criterion (Supplementary Tables S29 and S30). Due to such conflicts in terms of performance evaluation, the TOPSIS approach was used to choose the best method over the available methods (Supplementary Table S2) under the MCDM setting (Supplementary Table S4). The results from the MCDM-TOPSIS analyses are shown in Figure 3 and Supplementary Figures S13–S22. For Soumillon2 data, DECENT provided the highest TOPSIS score, followed by DESeqNB and LIMMA, compared to others (Figure 3A). In contrast, we found that the methods, including scDD, EMDomics, and EBSeq, performed worst among others under the MCDM setup. Further, when runtime criterion was included in MCDM-TOPSIS analysis, the tested methods’ rankings were found to be significantly changed (Figure 3A,B). For instance, the rank of DECENT slipped to 13 (Figure 3B) under the runtime included MCDM analysis from rank 1 (without runtime-MCDM analysis) (Figure 3A). This indicated that the performance of the best methods (when assessed under MCDM analysis) was compromised when their runtime was integrated into the analysis. Here, it is interesting to note that the bulk RNA-seq methods, such as LIMMA, DESeqNB, and edgeRQLF, performed better under the MCDM settings (Figure 3A). However, the univariate methods, including the T-test and Wilcox methods, performed well due to their lesser runtime (Supplementary Figure S13). Under the MCDM-TOPSIS (without runtime) settings, the DECENT was found to perform better, followed by Monocle, MAST, DEsingle, and BPSC, in the single-cell categories for Soumillon2 and Islam datasets (Figure 3A, Figure S13). Similar interpretations can be made from the MCDM-TOPSIS analysis for other datasets (Supplementary Figures S14–S22).

Through the MCDM analysis, it was found that the performance of the tested methods varies across the datasets and mostly depends on data characteristics, such as number of cells in the data (Supplementary Table S31). For instance, read count-based NB tools, including edgeRQLF, DESeqNB, and edgeRLRT, performed better when the total number of cells in the data is relatively small and performed poor for datasets with large number of cells (Supplementary Table S31). The specially designed UMI-based DECENT performed better, particularly when there was a sufficient number of cells present in the data (e.g., >1000) (Supplementary Table S31). However, the normalized data-based LIMMA performed exceptionally well for scRNA-seq data, which had larger sample sizes, but performed poorly under small sample situations.

#### 6.2.6. Between-Methods Similarity Analysis

The similarity analysis of the tested methods, based on the computed performance metrics, revealed similarities in their performances. We also compared the overlaps in terms of the detection of common DE genes between any pair of methods (i.e., the degree of similarity) through the Binomial test, as discussed in Supplementary Document S12. For Soumillon2 data, the results are shown in Figure 3C,D. Here, we observed that bulk RNA-seq methods, i.e., DESeqNB, edgeRQLF, NBSeq, and LIMMA, are grouped together with single-cell methods, such as DECENT, DEsingle, BPSC, MAST, and Monocle, along with general T-test and Wilcox methods (Figure 3C). Further, these methods shared a greater degree of similarity in terms of detecting common DE genes compared to other methods (Figure 3D, Supplementary Document S12). This finding was well supported with higher correlations among themselves (Supplementary Figure S23). In contrast, the methods which performed moderately well were clustered together, which included methods, such as DEGseq, ROTS, EBSeq, etc. Finally, the poorly performing methods (scDD and EMDomics), capable of dealing with the data’s multi-modal nature, were grouped together and shared fewer common DE genes with other methods (Figure 3C,D).

For Islam and other real datasets, similar interpretations can be made about the similarity of the tested methods (Supplementary Figures S13–S22). It is worth noting that the degree of similarity between DESeqNB and DESeqLRT was found to be low (Supplementary Figures S13 and S24), indicating that the DE test statistic has a significant effect on the performance of the methods. The degree of similarity in terms of sharing common genes between any given pair of methods varied widely (Figure 3D, Supplementary Figures S13–S22) within and across datasets and mostly depends on the real data characteristics (i.e., total number of cells and cells per group) (Supplementary Figures S13–S22). These findings were also partly supported by the previous studies [10,24,25].

#### 6.2.7. Combined-Data Methods Analysis

**Figure 4:**
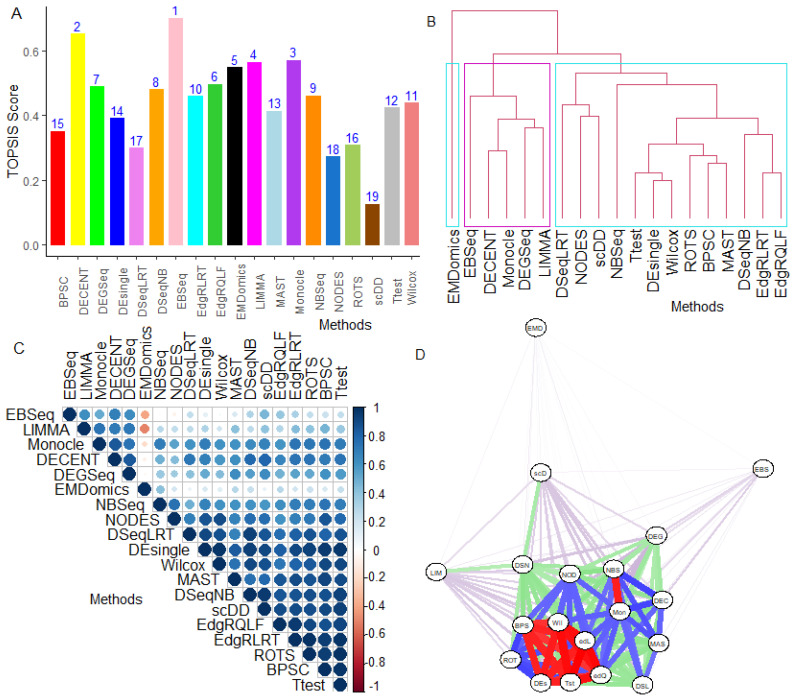
Combined data analysis of methods based on F1-score through the TOPSIS technique. Comparative performance evaluation of DE methods was performed based on F1score through the TOPSIS approach under multi-data setup. This analysis was performed on data matrix having F1-scores of tested methods across 11 considered datasets. (**A**) Shows results from the TOPSIS analysis of tested methods; (**B**) Similarity analysis of tested methods through clustering. Dendrogram was obtained by average-linkage hierarchical clustering based on the matrix of average F1-scores (over DE gene sets). (**C**) Correlation analysis of methods through rank correlation. Correlation plot was obtained by Spearman’s rank correlation method using the matrix of average (over DE gene sets) F1-scores across all datasets. (**D**) Weighted similarity analysis of tested methods (Supplementary Document S12) based on their ability to detect common genes. Nodes represent tested methods and edges represent shared degree of similarity between pairs of methods. Red color edges (with scores > 0.7) among methods indicated highest similarity, blue color edges indicate higher similarity ([0.5, 0.7]), green color edges represent with low similarity ([0.2, 0.5]), and magenta color edges represent lowest degree of similarity ([0, 0.2]) among the methods. Nodes in the network are abbreviated as EMD: EMDomics; LIM: LIMMA; EBS: EBSeq; scD: scDD; DEG: DEGseq; DSN: DESeqNB; NOD: NODES; BPS: BPSC; NBS: NBSeq; Wil: Wilcox; Mon: Monocle; DEC: DECENT; MAS: MAST; Tst: T-test; DEs: DEsingle; ROT: ROTS; DSL: DESeqLRT; edQ: edgeRQLF; and edL: edgeRLRT.

**Figure 5:**
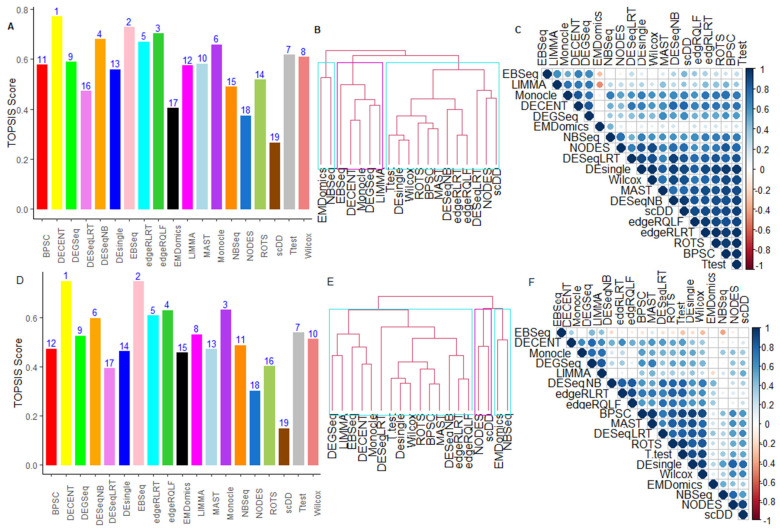
Combined data analysis of methods based on FDR and Accuracy metrics through TOPSIS technique. Comparative performance evaluation of DE methods was performed based on FDR and Accuracy metrics through TOPSIS technique under multi-data setup. This analysis was performed on the matrix having FDR and Accuracy scores of tested methods across 11 considered datasets. (**A**) TOPSIS analysis of tested methods based on FDR across real datasets. Bars show values of TOPSIS scores and number on each bar represents ranks; (**B**) Similarity analysis of methods based on FDR through clustering. Dendrogram was obtained by average-linkage hierarchical clustering based on the matrix of average FDR scores across all datasets. (**C**) Similarity analysis of methods based on FDR through correlation. Correlation plot was obtained by Spearman’s rank correlation method. White color boxes show non-significant correlation; (**D**) TOPSIS analysis of methods based on Accuracy across the real datasets. Bars show values of TOPSIS scores and number on each bar represent methods’ ranks; (**E**) Similarity analysis of methods based on Accuracy through clustering. (**F**) Similarity analysis of methods based on Accuracy metrics through correlation. Correlation plot was obtained by Spearman’s rank correlation for Accuracy scores across all datasets. Strength of correlation is shown through color intensity.

The performance of the tested methods was found to be highly inconsistent across the datasets (Supplementary Tables S19–S21). Therefore, we performed a combined-data analysis of the methods through the TOPSIS technique. For instance, edgeRQLF method performed better in Islam data but not in Ziegenhain data when assessed through the ACC metric (Supplementary Table S19). However, their performance was positively associated with the total number of cells and the number of cells per group (Supplementary Tables S31 and S32). To be more precise, with the selection of the best method across multiple real datasets, we performed TOPSIS analysis of the methods based on the criteria, such as F1, FDR, TPR, FPR, and AUROC, and the results are shown in Figure 4 and Figure 5, Figures S25 and S26, respectively. Through F1-based TOPSIS analysis, it was found that the score of EBSeq and DECENT was highest, followed by edgeRQLF, compared to others (Figure 4A). This indicates that both are better options for DE analysis over others across all the datasets but they are highly computationally intensive (Supplementary Document S11). Further, using F1-based similarity analysis, the parametric methods, such as DECENT, EBSeq, Monocle DEGseq, are grouped together (Figure 4B) and have similar performances to LIMMA (Figure 4C). However, EMDomics is the only NP method clustered separately, as it is a general-purpose method (does not consider single-cell data features) and its performance was negatively correlated with others (Figure 4B,C). The count-based bulk RNA-seq methods, such as DESeqNB, DESeqLRT, edgeRQLF, edgeRLRT, and NBSeq, are clustered together and were found to be similar with single-cell methods (i.e., BPSC, scDD, NODES, MAST and DEsingle) and general-purpose methods (i.e., T-test, Wilcox, and ROTS) (Figure 4B,C). Similar groupings of the methods and interpretations can be observed from the FDR, ACC, TPR, FPR, and AUROC-based similarity analysis of tested methods under combined-data setting (Figure 5, Figures S25 and S26). 

The combined-data similarity analysis of the tested methods, based on their ability to detect common DE genes (Supplementary Document S12), is shown in Figure 4D. The two group comparison methods (connected with red color edges), such as Wilcox, T-test, scDD, BPSC, DEsingle, shared a higher degree of similarity in terms of detecting more common genes compared to others (Figure 4D). Whereas the group of methods (connected with blue color edges) including single-cell methods (i.e., DECENT, Monocle, MAST, NODES, and BPSC) shared relatively lesser common DE genes (Figure 4D). Similar interpretations can be made for other tested methods. 

FDR and ACC-based TOPSIS analysis of the 19 methods over the 11 real datasets was also performed, and results are shown in Figure 5. It was found that the TOPSIS score (for both FDR and ACC) of DECENT was highest, followed by EBSeq compared to others (Figure 5A,D). Further, averaging the ACC measure across the DE gene sets and ranking the methods revealed that DECENT’s performance is the most consistent, followed by edgeRQLF and EBSeq (Supplementary Table S19). In comparison, performance of the NP methods, i.e., NODES, EMDomics, ROTS, and scDD, was found to be consistently poor across the datasets. Similar interpretations can be made for the other performance metrics, such as TPR, FPR, FDR, AUROC (Supplementary Tables S19–S21).

Among all of the tested methods, the single-cell methods, such as scDD, NODES, and DEsingle, performed poorly along with the general methods (EMDomics ad ROTS), and the bulk RNA-seq methods (DESeqLRT, NBSeq, and LIMMA) in terms of accuracy and robustness in detecting true DE genes (Figure 5A,D). The simple methods, such as the T-test and Wilcox, performed reasonably well with the least computational time required (Figure 5D, Document S11) to get better and more robust results. These methods, along with DEsingle, NODES, and ROTS, are limited to only two cell groups’ comparisons and cannot accommodate single-cell data features, whereas EMDomics can perform a limited number of analyses. The remaining methods implement the statistical frameworks that can accommodate more complex (fixed effect) designs, including comparisons across multiple groups and adjustments for batch effects and cell-level covariates. Further, there are specific methods, including Monocle, and LIMMA, which accurately detected the true DE genes but are prone to higher error rates (Figure 5A,D).

Sensitivity-specificity-based TOPSIS analysis of methods across the datasets indicated that DECENT and EBSeq performed well with higher sensitivities and specificities for detecting true DE genes (Supplementary Figure S25). While the methods, including Monocle, LIMMA, and EMDomics, were observed to have higher sensitivities, they compromised the specificities for detecting DE genes and had similar performance to NBSeq and DEGseq (Supplementary Figures S25 and S26). Further, the count-based RNA-seq methods (i.e., edgeRQLF, edgeRLRT, and DESeqNB) had higher specificities along with general methods (i.e., T-test and Wilcox), but compromised the sensitivities (Supplementary Figures S25 and S26). Surprisingly, the single-cell methods, such as BPSC, MAST, DEsingle, and NODES, were found to have lower sensitivities and specificities for DE analysis and have similar performance as ROTS. These findings were also supplemented by AUROC-based TOPSIS analysis (Supplementary Figure S26). 

The combined-data analysis through the TOPSIS technique allowed us to select the best option for DE analysis in single-cell studies. Through this, DECENT, consistently performed better, followed by EBSeq, and edgeRQLF, whereas the group of methods, such as scDD, NODES, ROTS, EMDomics, and DEsingle, always performed extremely poor. The DECENT’s superior performance may be attributed to it considering the cell capture rates, cell auxiliaries, and employing an efficient ECM algorithm for parameter estimation. Further, it is also well equipped to handle the molecular capture process, cell sizes, extra zero-inflation and biological dropout events present in the single-cell studies. It uses the ZINB model to fit the UMI data, which accurately estimates the mean and dispersion parameters, unlike other tools; thus, it has better accuracy (Supplementary Tables S5–S7). The remaining tested methods’ performances varied across the datasets under different performance metrics (Supplementary Tables S19–S21). Interestingly, the performances of the popular count-bulk RNA-seq methods, such as edgeRQLF and DESeqNB, were found to be consistently better than that of edgeRLRT and DESeqLRT, respectively (Figure 4 and Figure 5, Figures S25 and S26). This finding may be due to the good quality of UMI data and sufficient samples (i.e., cells) in single-cell datasets to estimate the dispersions. 

## 7. Conclusions and Future Work

The scRNA-seq is a rapidly growing field in gene expression genomics, and DE analysis is a popular downstream analysis that is performed on such data. Newer and better methods have been introduced over the years in the literature, that vary greatly on their utility, basic statistical concepts, models fitted, the test statistic(s) used, etc. Hence, it is pertinent for users to be updated on recent developments, the current status of the available methods, and, further, to choose the best method for their real data applications. Under these considerations, we presented a comprehensive survey of the available DE methods for scRNA-seq data analysis. Instead of individually reviewing them, we introduced a classification of the available methods, along with their unique features and limitations. In addition, we have performed a systematic comparison of 19 different methods that are extensively used for the DE analysis of scRNA-seq data, which broadly covers all of the classes of the methods. In this study, we focused on the most straightforward experimental design (i.e., comparing two cell groups). This design was also used in several computational studies, including the Soneson and Robinson study [25], which was conducted to assess the performances of the DE methods [2,18,54]. Still, our comprehensive study is unique with major strengths, including: (*i*) classification of existing methods; (*ii*) use of multiple real UMI datasets with varying sample sizes to capture true distributional nature and diversity of single-cell studies; (*iii*) assessment of methods based on individual-centric performance metrics; (*iv*) performance analysis of methods under MCDM setup; (*v*) combined-data analysis through TOPSIS technique; and (*vi*) similarity analysis of tested methods. Under the individual performance metric centric evaluations, it is not possible to find the globally best performing methods for DE analysis of scRNA-seq data, as their performances are data and performance metric(s) dependent. To search for the best method for DE analysis in scRNA-seq, we performed MCDM and combined-data analysis through the TOPSIS technique. Our findings revealed the practices, i.e., DECENT followed by EBSeq, that achieved better and more robust DE analysis of scRNA-seq UMI data. The DECENT is a single-cell method, specialized to handle UMI counts through ZINB model and consider dropout events, cell capture rates, and cell auxiliaries in DE analysis. The crucial conclusions from our work can be summarized as: (*i*) structured classification of existing methods; (*ii*) performance of the methods depends on real data characteristics; (*iii*) some bulk RNA-seq methods are competitive with single-cell methods; (*iv*) it is possible to find the globally best method through MCDM and combined-data analysis; and (*v*) similarities in the performance of the methods. This study will serve as a catalog and provide guidelines to genome researchers and experimental biologists to choose the best option objectively. 

In future, researchers may consider more complex experimental designs and gold-standard UMI datasets (with experimentally proven reference genes) to conduct comparative studies of single-cell methods. In addition, the effects of single-cell sequencing protocols (e.g., SMART-seq, Cel-seqs, and droplet) and the UMI or non-UMI nature of the data on the performance of the methods needs to be studied. The reported limitations and challenges will be addressed by statisticians and biologists collectively to develop innovative and efficient approaches. These approaches will analyze the data more efficiently in order to increase specificity, sensitivity, utility, and relevance of the single-cell studies.

## Figures and Tables

**Table 1:** Description of scRNA-seq DE methods.

SN.	Methods	Distribution	Utility	Input	DE Test Stat.	Runtime	Availability	Ref.
01	DESeq2	NB	Bulk-cell	Counts	Wald	Low	Bioconductor	[15]
02	edgeR	NB	Bulk-cell	Counts	QLF, LRT	Low	Bioconductor	[13]
03	LIMMA	Linear Model	Bulk-cell	Norm.	Bayesian Wald	Low	Bioconductor	[28,42]
04	DEGseq	Poisson	Bulk-cell	Counts	Z-score	Low	Bioconductor	[27]
05	T-test	T-test	General	Norm.	t stat.	Low	CRAN	[34,43]
06	Wilcoxon	Wilcoxon test	General	Counts	Wilcox	Low	CRAN	[35,43]
07	baySeq	NB	Bulk-cell	Counts	Posterior prob.	Low	Bioconductor	[38]
08	NBseq	NB	Bulk-cell	Counts	Fisher’s stat.	Low	CRAN	[29]
09	EBSeq	NB	Bulk-cell	Counts	Bayesian	High	Bioconductor	[30]
10	Cuffdiff	Beta-NB	Bulk-cell	sam file		Low	Linux	[44]
11	SAMseq	NP	Bulk-cell	Counts	Wilcox	Low	CRAN	[40]
12	Ballgown	Linear Model	Bulk-cell	Counts	Lin. Mod. test stat.	Medium	Bioconductor	[45]
13	TSPM	Poisson	Bulk-cell	Counts		Low	R code	[39]
14	ROTS	NP	Bulk-cell	Norm.	*Z*-stat. (bootstrap)	Medium	Bioconductor	[36,41]
15	metagenomeSeq		Bulk-cell			Medium		[46]
16	SCDE	Mixture Model	Single-cell	UMI	Bayesian Stat.	High	Bioconductor	[23]
17	scDD	Multi-Modal Bayesian	Single-cell	Norm.	Bayesian Stat.	High	Bioconductor	[32]
18	D3E	NP	Single-cell	UMI	Cramér-von Mises test/KS test	High	GitHub, Python	[47]
19	BPSC	Beta-Poisson	Single-cell	UMI	LRT	Medium	GitHub	[31]
20	MAST	Hurdle Model	Single-cell	Norm.	LRT	Medium	Bioconductor	[11]
21	Monocle2	GAM	Single-cell	Norm.	LRT	Medium	Bioconductor	[21,48]
22	DEsingle	ZINB	Single-cell	UMI	LRT	High	Bioconductor, GitHub	[20]
23	DECENT	ZINB, Beta-Binomial	Single-cell	UMI	LRT	High	GitHub	[18]
24	DESCEND	Poisson	Single-cell	UMI		High	GitHub	[17]
25	EMDomics	NP	Single-cell	Norm.	Euclidean distance	High	Bioconductor	[37]
26	Sincera	NP	Single-cell	Norm.	Welch’s t-stat. (LS) Wilcox (SS)	High	GitHub	[49]
27	ZIAQ	Logistic Regression	Single-cell	Norm.	Fisher’s stat.	Medium	GitHub	[50]
28	sigEMD	NP	Single-cell	Norm.	Distance measure	High	GitHub	[51]
29	TASC	Logistic, Poisson	Single-cell	UMI	LRT	High	GitHub	[52]
30	ZINB-Wave	ZINB	Single-cell	UMI	LRT	High	Bioconductor, GitHub	[12,53]
31	SwarnSeq	ZINB	Single-cell	UMI	LRT	High	GitHub	[54,55]
32	NODES	Wilcoxon test	Single-cell	Norm.	Wilcox	Medium	*Dropbox	[33]
33	BASiCS	Poisson-Gamma	Single-cell	Norm.	Posterior prob.	High	Bioconductor	[56]
34	NBID	NB	Single-cell	UMI	LRT	Medium	R code	[57]
35	tradeSeq	GAM	Single-cell	UMI	Wald	Medium	GitHub	[58]
36	SC2P	ZIP	Single-cell	UMI	Posterior prob.	High	GitHub	[59]

Bulk-cell: bulk RNA-seq; NB: Negative Binomial; ZINB: Zero Inflated Negative Binomial; ZIP: Zero Inflated Poisson; UMI: Unique Molecular Identification counts; single-cell: Single-cell RNA-seq; Norm.: Normalized (Continuous); Ref.: Reference; GAM: Generalized Additive Model; LRT: Likelihood Ratio Test; LS: Large Samples; SS: Small Samples; KS: Kolmogorov-Smirnov’s test; QLF: Quasi-Likelihood F-test; Wilcox: Wilcoxon signed rank/Mann–Whitney.

**Table 2:** Classification of methods used for detection of DE genes in scRNA-seq data.

SN.	Classes	Descriptions
01	Class I	Underlying Models: Negative Binomial Model; Linear Model; Poisson Model; Bayesian Model
		Features: Computationally simple; Requires less runtime; Applicable to both counts and normalized data
		Limitations: Does not consider multi-modality of data; Ignores dropout events; Fails to consider zero-inflation; Overestimates dispersion parameter; Underestimates mean (difference in mean across cellular condition); Lesser statistical power; Does not consider higher technical and biological variations; Cannot handle long-tailed (skewed) distributions; Ignores high sparsity in data
		Tools: DEseq2 [14,15], edgeR [13], Limma [28], SAMseq [40], DEGseq [27], baySeq [38], NBseq [29], Cuffdiff [44], Ballgown [45], TSPM [39], metagenomeSeq [46], ROTS [36,41], NOISeq [60] EBSeq [30], ShrinkSeq [61], GPseq [62], DeGPS [63]
02	Class II	Methods: NP methods
		Features: Distribution-free approach; Considers multi-modality of data distribution; Computationally not cumbersome; Estimates parameters without fitting distributions; Computes test statistic through distance-like metrics across two conditions/cell groups; Performs well when lesser proportions of zeros in data
		Limitations: Focuses on two cellular groups comparisons; Computationally complex for multi-groups; Performance severely affected due to high dropouts (some methods exclude dropouts); Cannot separate between true/biological and false/dropout zeros; Sensitive to sparsity; Methods like D3E and scDD fail to consider UMI count nature of data; Cannot separate technical from biological sources of variation; Cannot use cell-level auxiliary data
		Tools: D3E [47], scDD [32], sigEMD [51], NODES [33], EMDomics [37], Sincera [49], ZIAQ [50], Wilcox [35,43]
03	Class III	Models: Zero inflated Models; Hurdle Models; Mixture Models; GLM; GAM
		Features: Parametric approach; Captures only bi-modality distribution of data; Easily generalized to multi-cellular groups; Considers zero-inflations, dropout events; Methods like TASC, DECENT, SwarnSeq, etc. make use of external spike-ins to adjust distribution of observed data; Mostly uses GLM framework to compute DE statistics; Can accommodate cell-level auxiliary data while model building
		Limitations: Cannot capture multi-modality (>2) of data distribution; Methods like MAST failed to consider UMI nature of data and exclude dropout events; Methods like SCDE and MAST do not differentiate between true and dropout zeros during the model building; Computationally complex; Most of methods do not distinguish biological from technical factors that are causing dropouts; Assumes dropout events to be linear (ignores non-linear dropouts, especially for genes with low to moderate expression)
		Tools: SCDE [23], NBID [57], MAST [11], Monocle [21], Monocle2 [48], BPSC [31], ZINB-Wave [12], DEsingle [20], DECENT [18], DESCEND [17], TASC [52], BASiCS [56], Random Hurdle Model [64], SC2P [59], SwarnSeq [54,55]

SN.: Serial Number; DE: Differentially Expressed; GLM: Generalized Linear Model; GAM: Generalized Additive Model.

**Table 3:** List of the scRNA-seq datasets used in this study.

SN.	Data	Description	Accession	#Genes	#Cells	Ref.
01	Tung	Human induced Pluripotent stem cell lines	GSE77288	18938	576	[6]
02	Islam	single-cell (ES and MEF) transcriptional landscape by highly multiplex RNA-Seq	GSE29087	22928	92	[5]
03	Soumillon1	Differentiating adipose cells by scRNA-Seq (Day 1 vs. 2)	GSE53638	23895	1835	[67]
04	Soumillon2	Differentiating adipose cells by scRNA-Seq (Days 1 vs. 3)	GSE53638	23895	2268	[67]
05	Soumillon3	Differentiating adipose cells by scRNA-Seq (Days 2 vs. 3)	GSE53638	23895	1613	[67]
06	Klein	Mouse ES cells	GSE65525	24174	1481	[68]
07	Gierahn	Single-cell RNA sequencing experiments of HEK cells	GSE92495	24176	1453	[69]
08	Chen	ScRNA-seq of Rh41 using 10x Genomics	GSE113660	33694	7261	[57]
09	Savas	Breast cancer cells using 10x Genomics	GSE110686	33694	6311	[70]
10	Grun	Mouse ES single cells using CEL-seq technique	GSE54695	12467	320	[71]
11	Ziegenhain	Sc-RNA-seq of Mouse ES cells	GSE75790	39016	583	[72]

SN: Serial Number; #Genes: number of genes/transcripts, #Cells: number of cells; ES: Embryonic Stem; MEF: Mouse Embryonic Fibroblast; Ref.: Reference.

**Table 4:** Evaluation of DE methods based on performance evaluation metrics for Soumillon2 scRNA-seq data.

	TP	FP	TN	FN	TPR	FPR	FDR	PPR	NPV	ACC	F1	AUROC
BPSC	1478	1522	11113	1522	0.493	0.120	0.507	0.493	0.880	0.805	0.493	0.722
DECENT	1674	1326	11309	1326	0.558	0.105	0.442	0.558	0.895	0.830	0.558	0.857
DEGseq	1228	1772	10863	1772	0.409	0.140	0.591	0.409	0.860	0.773	0.409	0.585
DESeqNB	1653	1347	11288	1347	0.551	0.107	0.449	0.551	0.893	0.828	0.551	0.811
DESeqLRT	1247	1753	10882	1753	0.416	0.139	0.584	0.416	0.861	0.776	0.416	0.666
DEsingle	1428	1572	11063	1572	0.476	0.124	0.524	0.476	0.876	0.799	0.476	0.709
EBSeq	1110	1890	10745	1890	0.370	0.150	0.630	0.370	0.850	0.758	0.370	0.654
edgeRLRT	1537	1463	11172	1463	0.512	0.116	0.488	0.512	0.884	0.813	0.512	0.729
edgeRQLF	1506	1494	11141	1494	0.502	0.118	0.498	0.502	0.882	0.809	0.502	0.758
EMDomics	844	2156	10479	2156	0.281	0.171	0.719	0.281	0.829	0.724	0.281	0.594
LIMMA	1612	1388	11247	1388	0.537	0.110	0.463	0.537	0.890	0.822	0.537	0.768
MAST	1337	1663	10972	1663	0.446	0.132	0.554	0.446	0.868	0.787	0.446	0.685
Monocle	1454	1546	11089	1546	0.485	0.122	0.515	0.485	0.878	0.802	0.485	0.691
NBSeq	1497	1503	11132	1503	0.499	0.119	0.501	0.499	0.881	0.808	0.499	0.718
NODES	1173	1827	10808	1827	0.391	0.145	0.609	0.391	0.855	0.766	0.391	0.620
ROTS	1170	1830	10805	1830	0.390	0.145	0.610	0.390	0.855	0.766	0.390	0.618
scDD	697	2303	10332	2303	0.232	0.182	0.768	0.232	0.818	0.705	0.232	0.547
T-test	1501	1499	11136	1499	0.500	0.119	0.500	0.500	0.881	0.808	0.500	0.719
Wilcox	1413	1587	11048	1587	0.471	0.126	0.529	0.471	0.874	0.797	0.471	0.695

TP: True Positives; FP: False Positives; TN: True Negatives; FN: False Negatives; TPR: True Positive Rate; FPR: False Positive Rate; FDR: False Discovery Rate; PPR: Positive Prediction Rate; NPV: Negative Prediction Value; ACC: Accuracy; F1: F1 score; AUROC: Area Under Receiver Operating Curve; Values are computed for DE gene set of size 3000.

## Data Availability

All the statistical analyses are performed through R software. The R codes and the processed scRNA-seq datasets are publicly available at the RoopSeq GitHub directory (https://github.com/sam-uofl/RoopSeq). All the datasets used in this study are publicly available at GEO database of NCBI.

## Supplementary Materials

The following are available online at https://www.mdpi.com/article/10.3390/genes12121947/s1, Documentary S1: Descriptions of the Single-cell datasets used in this study; Document S2: Selection of reference genes for single-cell studies; Document S3: Count data models; Document S4: Normalization methods in scRNA-seq data analysis; Document S5: List of the methods and tools used in the comparative study; Document S6. Comparative performance analysis of the tested methods using TOPSIS technique under Multiple Criteria Decision-Making setup; Document S7: Testing for zero-inflation parameters for genes in scRNA-seq data; Document S8: Statistical testing for overdispersion parameters in scRNA-seq data; Document S9: Application of Count Data Models to Zero-Inflated and Overdispersed Real Datasets; Document S10: Comparative analysis of NB and ZINB models; Document S11: Performance evaluation of DE methods based on runtime criterion; Document S12: Similarity analysis of the scRNA-seq DE methods based on their ability to detect common DE genes; Table S1: Filtered scRNA-seq datasets along with used in this study; Table S2: ScRNA-seq DE Tools details used in this study and their availability; Table S3: List of the scRNA-seq DE methods along with the parameters used in this comparative study; Table S4: List of the criteria used for comparative performance analysis of the scRNA-seq DE methods; Table S5: Fitting of well-known count models to over-dispersed and zero-inflated cyst count data; Table S6: Fitting of well-known discrete models to over-dispersed and zero-inflated scRNA-seq read count data; Table S7: Comparative analysis of NB and ZINB models for estimation of parameters from scRNA-seq data; Table S8: Evaluation of DE methods based on performance evaluation metrics for Soumillon2 scRNA-seq data; Table S9: Evaluation of DE methods based on performance evaluation metrics for Islam scRNA-seq data; Table S10: Evaluation of DE methods based on performance evaluation metrics for Tung scRNA-seq data; Table S11: Evaluation of DE methods based on performance evaluation metrics for Ziegenhain scRNA-seq data; Table S12: Evaluation of DE methods based on performance evaluation metrics for Grun scRNA-seq data; Table S13: Evaluation of DE methods based on performance evaluation metrics for Savas scRNA-seq data; Table S14: Evaluation of DE methods based on performance evaluation metrics for Soumillon1 scRNA-seq data; Table S15: Evaluation of DE methods based on performance evaluation metrics for Soumillon3 scRNA-seq data; Table S16: Evaluation of DE methods based on performance evaluation metrics for Klein scRNA-seq data; Table S17: Evaluation of DE methods based on performance evaluation metrics for Gierahn scRNA-seq data; Table S18: Evaluation of DE methods based on performance evaluation metrics for Chen scRNA-seq data; Table S19: Ranking of DE methods across all datasets based on the performance metrics; Table S20: Ranking of DE methods across all datasets through AUROC metric; Table S21: Ranking of DE methods across all datasets based on the FDR metric; Table S22: Conflicts among the criteria for ranking of the DE methods for Geinhein data; Table S23: Conflicts among the criteria for ranking of the DE methods for Islam data; Table S24: Conflicts among the criteria for ranking of the DE methods for Tung data; Table S25: Conflicts among the criteria for ranking of the DE methods for Islam Ziegenhain data; Table S26: Conflicts among the criteria for ranking of the DE methods for Grun data; Table S27: Conflicts among the criteria for ranking of the DE methods for Soumillon1 data; Table S28: Conflicts among the criteria for ranking of the DE methods for Klein data; Table S29: Conflicts among the criteria for ranking of the DE methods for Soumillon3 data; Table S30: Conflicts among the criteria for ranking of the DE methods for Soumillon2 data; Table S31: Effect of the number of cells on the performance (ranking) of the DE methods assessed through AUROC; Table S32: ANOVA table for studying the effects of number of cells and cells/group on performance of DE methods; Figure S1: Schematic overview and layout of scRNA-seq DE analysis; Figure S2: Data Characteristics, Distributions and Fitting of Various Discrete Data Models; Figure S3. Comparative performance evaluation of the Differential Expression (DE) methods through the performance metrics for Islam data; Figure S4: Comparative performance evaluation of the DE methods through the performance metrics for Tung data; Figure S5: Comparative performance evaluation of the DE methods through the performance metrics for Soumillon1 data; Figure S6: Comparative performance evaluation of the DE methods through the performance metrics for Soumillon 3 data; Figure S7: Comparative performance evaluation of the DE methods through the performance metrics for Klein data; Figure S8: Comparative performance evaluation of the DE methods through the performance metrics for Gierahn data; Figure S9: Comparative performance evaluation of the DE methods through the performance metrics for Chen data; Figure S10: Comparative performance evaluation of the DE methods through the performance metrics for Savas data; Figure S11: Comparative performance evaluation of the DE methods through the performance metrics for Grun data; Figure S12: Comparative performance evaluation of the DE methods through the performance metrics for Ziegenhain data; Figure S13: Performance evaluation of DE methods under Multiple Criteria Decision Making (MCDM) setup for Islam data; Figure S14: Performance evaluation of DE methods under the MCDM setup for Tung data; Figure S15: Performance evaluation of DE methods under the MCDM setup for Soumillion1 data; Figure S16: Performance evaluation of DE methods under the MCDM setup for Soumillion3 data; Figure S17: Performance evaluation of DE methods under the MCDM setup for Klein data; Figure S18: Performance evaluation of DE methods under the MCDM setup for Gierahn data; Figure S19: Performance evaluation of DE methods under the MCDM setup for Chen data; Figure S20: Performance evaluation of DE methods under the MCDM setup for Savas data; Figure S21: Performance evaluation of DE methods under the MCDM setup for Grun data; Figure S22: Performance evaluation of DE methods under the MCDM for Ziegenhain data; Figure S23: Correlation plot for the tested methods over the computed performance metrics for Soumillon2 data; Figure S24: Correlation plot for the tested methods over the computed performance metrics for the other ten real scRNA-seq datasets; Figure S25: Combined data analysis of the DE methods based on TPR and FPR metrics through TOPSIS Approach; Figure S26: Combined data analysis of the DE methods based on AUROC metrics through TOPSIS Approach.
