# Supplementary material for: A Comprehensive Survey of Statistical Approaches for Differential Expression Analysis in Single-Cell RNA Sequencing Studies
Source: Genes (Basel). 2021 Dec 2;12(12):1947. doi: 10.3390/genes12121947 (PMC8701051; doi:10.3390/genes12121947)
Supplement: Supplementary file 1 [file genes-12-01947-s001.zip › genes-1462517-supplementary.pdf]

Supplementary Materials

of

A Comprehensive Survey of Statistical Approaches for Differential Expression Analysis in Single-cell RNA Sequencing Studies

Samarendra Das[1-3], Anil Rai[4], Michael L. Merchant[5,6], Matthew C. Cave[7] and Shesh N. Rai[2,3,6-9 *]

[1]Division of Statistical Genetics, ICAR-Indian Agricultural Statistics Research Institute, PUSA, New Delhi 110012, India

[2]Biostatistics and Bioinformatics Facility, JG Brown Cancer Center, University of Louisville, Louisville, KY 40202, USA

[3]School of Interdisciplinary and Graduate Studies, University of Louisville, Louisville, KY 40292, USA

[4]Centre for Agricultural Bioinformatics, ICAR-Indian Agricultural Statistics Research Institute, PUSA, New Delhi 110012, India

[5]Department of Medicine, School of Medicine, University of Louisville, Louisville, KY 40202, USA

[6]Hepatobiology and Toxicology Center, University of Louisville, Louisville, KY 40202, USA

[7]Biostatisitcs and Informatics Facility, Center for Integrative Environmental Health Sciences, University of Louisville, Louisville, KY 40202, USA

[8]Christina Lee Brown Envirome Institute, University of Louisville, Louisville, KY 40202, USA

[9]Department of Bioinformatics and Biostatistics, School of Public Health and Information Science, University of Louisville, Louisville, KY 40202, USA

Authors' email addresses:
SD: samarendra.das@louisville.edu
AR: anil.rai@icar.gov.in
MLM: michael.merchant@louisville.edu
MCC: matt.cave@louisville.edu
SNR: shesh.rai@louisville.edu

***To whom the correspondence should be addressed-** email: shesh.rai@louisville.edu.

Contents

Sl. No.	Topics	Page
1	Descriptions of the single-cell datasets	1 – 7
2	Reference genes for single-cell studies	17 – 10
3	Count data models in scRNA-seq	10 – 14
3.1	Negative Binomial Model	
3.2	Zero Inflated Negative Binomial Model	
3.3	Poisson Model	
3.4	Zero Inflated Poisson Model	
3.5	Hermite distribution model	
4	Normalization methods in scRNA-seq data analysis	14 – 15
4.1	TMM	
4.2	DESeq-norm	
5	List of the methods used in this comparative study	15 – 18
6	List of the criteria used in this study	18 – 19
7	MCDM-TOPSIS Analysis	19 – 21
8	Testing for zero inflation in scRNA-seq data	22 – 23
9	Testing for overdispersion parameters in scRNA-seq data	23 – 24
10	Application of count models	24 – 29
10.1	Application to embryonic mouse cysts data	
10.2	Application to real scRNA-seq data	
11	Comparative analysis of NB and ZINB models	30 – 31
12	Performance evaluation of methods based on runtime criterion	32 – 33
13	Similarity analysis of methods	34 – 36
13.1	Similarity analysis based on detection of common DE genes	
13.2	Combined analysis of methods based on common DE genes	
14	Supplementary Figures	37 – 60
15	Computed performance metrics (Tables)	61 – 93
16	Ranking of methods across all datasets	94 – 97
17	Conflicts among criteria in real datasets	98 – 101
18	Effects of data characteristics of methods' performance	102
19	References	103 – 105

Documentary S1: Descriptions of the Single-cell datasets used in this study

A. ***Islam Data*** (https://www.ncbi.nlm.nih.gov/geo/query/acc.cgi?acc=GSE29087): This dataset is publicly available in NCBI Gene Expression Omnibus (GEO) database with accession GSE29087 [1] and widely used for benchmarking of scRNA-seq DE methods. Single-cell expression profiles were available for 96 cells (48 mouse Embryonic Stem (ES) cells, 44 Mouse Embryonic Fibroblasts (MEF) and 4 negative controls). Here, the negative control cells were removed from further analysis. For DE analysis, ES and MEF cell lines are considered as two cellular groups. Here, we have not removed any cells from analysis, and further removed the genes which have non-zero expressions in ≤ 5 cells. Through this process, we selected expression counts of 11436 genes over 92 cells. A brief description about this data is given in Table S1.

B. ***Tung Data*** (www.ncbi.nlm.nih.gov/geo/query/acc.cgi?acc=GSE77288): This dataset is publicly available in NCBI GEO database with accession id GSE77288 [2] and the UMI count data is also available at GitHub. We downloaded the filtered UMI count matrix along with the spike-ins and molecular concentration data from their GitHub repository (https://github.com/jdblischak/singleCellSeq). The full dataset contains three Yoruba (YRI) induced pluripotent stem cell (iPSC) lines. Here, we only used data of two individual cell lines NA19101 (288 cells) and NA19239 (288 cells) for further analyses. Here, we have not removed any cells from analysis, but removed the genes which have non-zero expressions in ≤ 5 cells to reduce the size of the data. Through this, we selected expression counts of 15,955 genes over 576 cells for further analysis. Further, for DE analysis, we took two cell lines (*i.e.,* NA19101 and NA19239) as two cellular groups. A brief description about this data is given in Table S1.

C. ***Soumillon Datasets*** (www.ncbi.nlm.nih.gov/geo/query/acc.cgi?acc=GSE53638): This dataset is publicly available from NCBI GEO database with Accession GSE53638 [3]. In this

study, cells were extracted during directed differentiation of human adipose-derived stem/stromal cells and further, 11, 116 cells are profiled by the authors of the original publication. Here, cells were collected at different stages and different time points (day 0, day 3 and day 7) of differentiation of human adipose-derived stem/stromal cells. FACS sorted cells were sequenced using the SCRB-seq protocol with UMI. To study the performance of scRNA-seq DE tools, we used two group comparison settings based on different time points, *i*) Data 1 (Day 0 (1245 cells, baseline) vs. Day 3 (590 cells), (*ii*) Data 2 (Day 0 (1245 cells, baseline) vs. Day 7 (1023 cells), and (*iii*) Data 3 (Day 7 (1023 cells, baseline) vs. Day 3 (590 cells). Here, we have not removed any cells from the analysis, and further removed the genes which have non-zero expressions in ≤ 5 cells. Through this process, we selected expression counts of 14863, 15637, and 15015 genes over 1835, 2268, 1613 cells for Soumillon Data 1, Data 2, and Data 3 respectively.

D. ***Klein Data*** (www.ncbi.nlm.nih.gov/geo/query/acc.cgi?acc=GSE65525): Klein data, mouse scRNA-seq count datasets, freely accessible from NCBI GEO database with accession id GSE65525 [4]. In this experiment, the mouse embryonic stem cells expressions were profiled through high throughput sequencing using a droplet-microfluidic approach. Here, the study includes 8 single cell datasets: 3 for mouse ES cells (1 biological replicate, 2 technical replicates); 3 samples following LIF withdrawal (days 2,4, 7); one pure RNA data set (from human lymphoblast K562 cells); and one sample of single K562 cells. We took the counts data, which are already undergone through several quality control steps by the authors of the original publication. Further, at the preliminary stage, we removed the cells whose library size is less than 1500 and also removed the genes which have non-zero expressions in ≤ 5 cells. Through this process, we selected expression counts of 23971 genes over 1481 cells for further analysis. Further, for DE

analysis, we took day 4 (cells 683) as group 1 and day 7 (798 cells) as group 2. A brief description about this data is given in Table S1.

E. ***Ziegenhain Data*** (www.ncbi.nlm.nih.gov/geo/query/acc.cgi?acc=GSE75790): This dataset is a publicly available scRNA-seq count data in NCBI GEO database with accession id GSE75790. In this experimental study, the single-cell datasets were generated for 583 mouse ES cells through six prominent scRNA-seq protocols, *viz.* CEL-seq2 (71), Drop-seq (76), MARS-seq (65), SCRB-seq (84), Smart-seq (130), and Smart-seq2 (157). Here, we considered the single-cell data generated for 287 cells through Smart-seq protocol as one cell group and the remaining cells (296) for which data is generated using other protocols is considered as another cellular group. This dataset contains expression counts of 39,016 transcripts over 583 cells. At the preliminary stage, we kept all those transcripts whose expression are non-zero counts in at least 30 cells. Through this process, we selected the single-cell expression count data of 15579 transcripts over 583 cells for further analysis. A brief description about this data is given in Table S1.

F. ***Grun data*** (https://www.ncbi.nlm.nih.gov/geo/query/acc.cgi?acc=GSE54695): This dataset is publicly available in NCBI GEO database with accession GSE54695. In this experimental study, the J1 mouse ES cells were cultured in 2i or in serum medium. Single cells were processed using the CEL-seq technique protocol. In this experiment, a 4bp random barcode as unique molecular identifier (UMI) was added to the primer in between the cell specific barcode and the poly T stretch. Libraries were sequenced on an Illumina HighSeq 2500 using 50bp paired end sequencing. For benchmarking scRNA-seq DE tools, we considered count dataset for J1 cells, as it has expressions of thousands of genes over hundreds of cells. Further, at the preliminary stage, we removed the cells whose library size is less than 1000 and also removed the genes which have non-zero expressions in ≤ 20 cells. Through this process, we selected expression counts of 11570

genes over 274 cells for further analysis. For performing DE analysis on this dataset, the cell group information is necessary, which is known here. Here, we considered those (152) cells, cultured in 2i medium, as one cellular group, and other (122) cells, cultured in serum medium, as another cellular group.

G. ***Gierahn Data*** (https://www.ncbi.nlm.nih.gov/geo/query/acc.cgi?acc=GSE92495): This dataset is publicly available in NCBI GEO database with accession id GSE92495 [5]. This experiment contains scRNA-seq dataset of Mouse and Human HEK/3T3 mixing, PBMCs, and TB-exposed cell Macrophages generated through Seq-Well, a portable, low-cost platform for single-cells. For benchmarking scRNA-seq DE tools, we considered count dataset for HEK cells, as it has expressions of thousands of genes over a large number of cells. Further, at the preliminary stage, we removed the cells whose library size is less than 1500 and also removed the genes which have non-zero expressions in ≤ 5 cells. Through this process, we selected expression counts of 15524 genes over 1453 cells for further analysis. For performing DE analysis on this dataset, the cell group information is necessary, which is unknown. Therefore, we used the clustering technique and methodology developed by Das et al. (2021) [6,7] to determine the optimum number of cell clusters for this data. For this purpose, we used the *OptimCluster* function implemented in SwarnSeq R package [6] to decide the number of optimum cell clusters. The results are shown in following Figure. Here, we set the seed value at 208 and, found that the 1453 cells are clustered into 8 optimum cell clusters. Further, for DE analysis, we took cell cluster 8 (cells 537) as group 1 and remaining cell clusters (916 cells) as group 2, as below shown Table. A brief description about this data is given in Table S1.

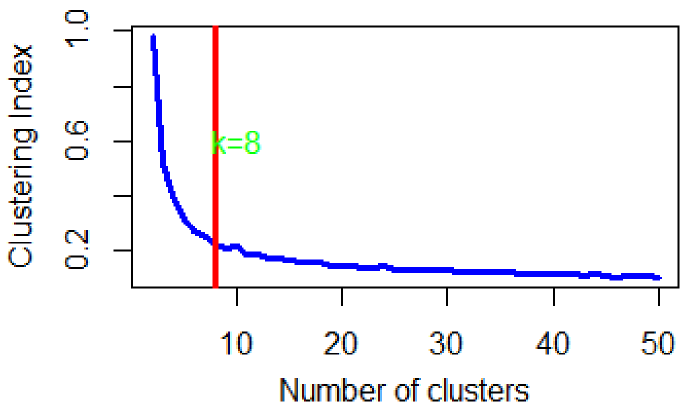

Choosing optimum number of cell clusters for Gierahn data.

The cell cluster and cellular group membership of the cells present in Gierahn dataset is shown in below mentioned Table.

Table. Cell cluster and cell group information for Gierahn data.

Cell Clusters	1	2	3	4	5	6	7	8
Number of cells	268	194	126	16	172	38	102	537
	Group 1 (916)							Group 2 (537)

H. ***Chen data*** (https://www.ncbi.nlm.nih.gov/geo/query/acc.cgi?acc= GSE113660): This data is available in GEO database with accession GSE113660. This scRNA-seq experiment was conducted over seven thousand two hundred cells from the Rh41 cell lines. For this study, we only collected the scRNA-seq UMI count datasets to analyze the tested methods. Here, we removed the cells whose library size <15000 and also removed the genes which have non-zero expressions in ≤5 cells. Through this process, we selected expression counts of 18406 genes over 6406 cells for further analysis. For DE analysis, the cell group information is necessary, which is unknown here. Therefore, we used the cluster analysis technique and methodology developed by Das et al. (2021) [6,7] to determine the optimum number of cell clusters for this data. We used the *OptimCluster* function implemented in SwarnSeq R package [6] to decide the number of optimum cell clusters.

The results are shown in following Figure. Here, we set the seed value at 208 and, found that the 6406 cells are clustered into 7 optimum cell clusters. Further, for DE analysis, we took cell clusters 6 and 7 (cells 2990) as group 2 and remaining cell clusters (3416 cells) as group 1, as shown in below Table. A brief description about this data is given in Table S1.

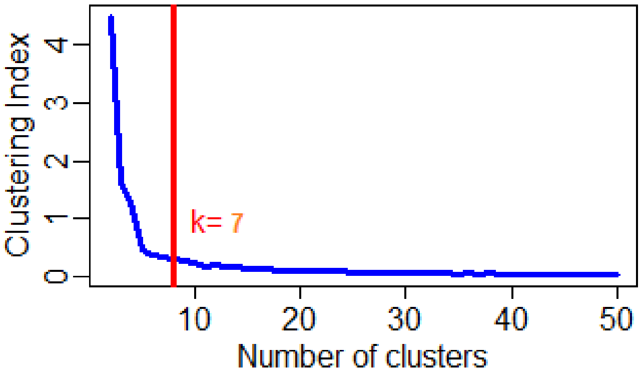

Determination of optimum number of cell clusters for Chen data. The number of cells in each cell clusters and group membership of the cell clusters are given in below Table.

Cellular group and cellular clusters information for Chen data.

Cell clusters	1	2	3	4	5	6	7
Number of cells	169	393	990	1766	98	2201	789
	Group 1 (3416)					Group 2 (2990)	

I. ***Savas Data*** (https://www.ncbi.nlm.nih.gov/geo/query/acc.cgi?acc= GSE110686): In this study, T cells were isolated from primary tumour tissues of two breast cancer patients. Single cells were captured from the sorted cells through the 10X Genomics Chromium System and libraries were sequenced using Illumina HiSeq 2500. Here, we collected the UMI and read count datasets from the single-cell and bulk RNA-seq to analyze the methods. At the preliminary stage, we removed the cells whose library size is less than 15000 and also removed the genes which have non-zero expressions in ≤ 5 cells. Through this, we selected expression counts of 12753 genes over

2903 cells for further analysis. As the cell group information is unknown, we used the cluster analysis technique (Supplementary Document S1) to determine the optimum number of cell clusters, which was implemented in *OptimCluster* function of SwarnSeq R package. Here, we found that the 2903 cells are clustered into 6 optimum cell clusters. Further, for DE analysis, we took cell cluster 6 (cells: 1290) as group 2 and remaining cell clusters (cells: 1613) as group 1. A brief description about this data is given in Table S1. The number of cells in each cell clusters are given in below mentioned Table.

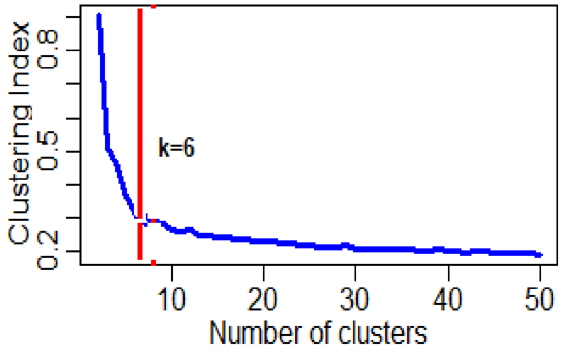

Determination of optimum number of cell clusters for Savas data.

Cell groups and cell clusters information for Savas data.

Cell Clusters	1	2	3	4	5	7	6
Number of cells	266	58	40	192	844	213	1290
	Group 1 (1613)						Group 2 (1290)

<u>Document S2</u>: Selection of reference genes for single-cell studies

To capture the real distributional of the single-cell data, we assessed the performance of the tested methods on 11 different publicly available real scRNA-seq UMI counts datasets having cells ranging from few hundreds to several thousands (Table S1). These datasets are briefly described in Document S1. Therefore, such an approach of performance evaluation was capable

of taking the biological ground truth of real DE genes for the benchmarking of performance the tested methods. However, it is more complicated to obtain the available list of real true DE genes for each of the considered dataset. To tackle this problem, we selected few known biological relevant datasets, such as Microarrays, bulk RNA-seq, to get the list of reference DE genes for which single-cell RNA-seq datasets are available for the same cell-lines. The detail procedure for obtaining the reference genes for each of the 11 considered real datasets to validate the performance of the tested methods is described as below.

***Islam Data (GSE29087)*:** For this dataset, we obtained the reference DE genes (the same cell lines for which single-cell dataset was generated) from the Microarray dataset. Further, we collected the Microarray datasets available for the mouse Embryonic Stem (ES) cell types (class label: 1), and Embryonic Fibroblasts (EF) cell types (class label: -1). This dataset is publicly available at http://carlosibanezlab.se//Data/Moliner_CELfiles.zip [8]. We used the Support Vector Machine-Recursive Feature Elimination (SVM-RFE) [9] to select the genes, which are differentially expressed between the ES and EF cell lines from the Microarray expression data and assumed these selected differentially expressed genes as reference genes for benchmarking the tested methods. The SVM-RFE is a machine learning algorithm, widely used for informative gene selection in Microarray data analysis and has superior performance compared to other gene selection methods, as established in the previous study [10]. Through the SVM-RFE, the top-ranked 3000 genes were selected and considered as the reference genes for performance analysis of the scRNA-seq DE methods.

***Tung data* (GSE77288)**: For this single-cell dataset, we obtained the reference DE genes (for the NA19101 and NA19239 cell lines) from the relevant bulk RNA-seq data. This bulk RNA-seq data, generated for the NA19101 and NA19239 cell lines, is publicly available at

https://github.com/jdblischak/singleCellSeq. Here, we used the SAMseq tool [11] to select the differentially expressed between the NA19101 and NA19239 cell lines from the bulk RNA-seq count data and assumed these selected differentially expressed genes as reference genes for benchmarking the tested methods. The rationale behind using SAMseq technique is that it is based on non-parametric statistical approach and does not depend on the distributional nature of the data [11]. Through this tool, we selected the top ranked 3000 genes as reference genes for benchmarking the performance of the 19 tested single-cell methods (Tables S2, S3).

Savas data **(GSE110686):** For Savas single-cell data, we collected the bulk RNA-seq count datasets for the corresponding cell lines from GEO database of NCBI with accession id GSE110938. Here, we used the SAMseq tool [11] to select the differentially expressed between the CD8+CD103+ and CD8+CD103- FACS sorted populations. Through this tool, we selected the top ranked 3000 genes as reference genes for benchmarking the performance of the 19 tested single-cell methods (Tables S2, S3).

Other scRNA-seq datasets: For the remaining eight single-cell datasets, we selected the reference genes from these data itself. Hence, to obtain the list of reference genes, we used Fold Change (FC) criterion (*i.e.,* ratios of mean expressions of genes over the two cellular groups, as given in Table S1). The detailed descriptions about the identification of cellular groups for each of the remaining eight datasets including Soumillon Datasets (Data 1, 2, and 3), Klein Data, Ziegenhain Data, Grun data, Gierahn Data, and Chen data is described in Supplementary Document S10. For each of the dataset, we selected the top 3000 genes based on the FC criterion as reference gene lists. It is important to note that the selection of reference genes from these datasets is completely independent of the detection of DE genes through the tested DE methods.

The selection of reference genes through FC criterion provides an equal platform for the comparative analysis of the 19 tested methods on each of the datasets.

Table S1. Filtered scRNA-seq datasets along with used in this study.

Sl. No.	Data	scRNA Accession	Ref. genes	Filtering	#Cells*	#Genes*	#Cells in G1	#Cells in G2
01	Tung	GSE77288	Bulk RNA-seq	(5, 1000)	576	15955	288	288
02	Islam	GSE29087	Microarrays	(5, 1000)	92	11436	48	44
03	Soumillon 1	GSE53638	scRNA-seq	(5, 1000)	1835	12289	1245	590
04	Soumillon 2	GSE53638	scRNA-seq	(5, 1000)	2268	15637	1245	1023
05	Soumillon 3	GSE53638	scRNA-seq	(5, 1000)	1613	15015	590	1023
06	Klein	GSE65525	scRNA-seq	(5, 1500)	1481	23971	683	798
07	Gierahn	GSE92495	scRNA-seq	(4, 1000)	1453	15996	916	537
08	Chen	GSE113660	Bulk RNA-seq	(5, 15000)	6406	18406	3416	2990
09	Savas	GSE110686	Bulk RNA-seq	(5, 15000)	2903	12753	1613	1290
10	Grun	GSE54695	scRNA-seq	(20, 1000)	274	11570	152	122
11	Ziegenhain	GSE75790	scRNA-seq	(30, 1000)	583	15579	296	287

#Cells*, #Genes* are the number of cells and genes in the scRNA-seq data after data pre-processing; #Cells in G1: number of cells in group 1; #Cells in G2: number of cells in group 2; Filtering: (x, y) Filtering parameters, where x represents the number of non-zero counts for each gene removed, y represents the cell size threshold; Ref. genes: GEO datasets from which reference genes are derived.

Document S3: Count data models

a. Negative Binomial Distribution

Most of the popular Differential Expression (DE) analysis tools, *e.g.* DESeq [12], DESeq2 [13], edgeR [14], *etc.*, for bulk RNA-sequencing (RNA-seq) study assume the RNA-seq read counts to follow a Negative Binomial (NB) distribution, and subsequently, DE analysis is performed under Generalized Linear Model (GLM) framework.

Let, Y_{ij}: random variable (*rv*) representing the RNA-seq read counts of ith (i = 1, 2, …, N) gene of jth (j = 1, 2, …, M) cell; μ_{ij}: mean of ith gene of jth cell in i^{th} cell; θ_{ij} $(= \varphi_{ij}^{-1})$ and φ_{ij}: size and dispersion parameters respectively of ith gene in j^{th} cell for NB distribution.

Further, the Probability Mass Function (PMF) of the NB distribution is expressed as:

$$f_{NB}(y) = P[Y_{ij} = y] = \frac{G(y+\theta_{ij})}{G(y+1)G(\theta_{ij})}\left(\frac{\theta_{ij}}{\theta_{ij}+\mu_{ij}}\right)^{\theta_{ij}}\left(\frac{\mu_{ij}}{\theta_{ij}+\mu_{ij}}\right)^{y} \quad \forall\, y = 0, 1, 2, \ldots \tag{1}$$

where, $\mu_{ij} \geq 0$; $\theta_{ij} > 0$ are the parameters of NB distribution, G(.): Gamma function. Then, the expected value and variance of Y_{ij} is shown as:

$$E(Y_{ij}) = \mu_{ij} \tag{2}$$

$$V(Y_{ij}) = \mu_{ij} + \frac{\mu_{ij}^2}{\theta_{ij}} = \mu_{ij} + \varphi_{ij} \tag{3}$$

If $\varphi_{ij} \to 0$ $(No\ dispersion)$ $\Rightarrow NB(\mu_{ij}, \theta_{ij}) \to Poisson(\mu_{ij})$

b. Zero Inflated Negative Binomial Model

The proportions of zeros in single cell RNA-sequencing (scRNA-seq) data are higher as compared to bulk RNA-seq data due to low efficiency of mRNA capture efficiency, lower abundance of transcriptomics in single cell, amplification bias, *etc.* Therefore, the application of NB based bulk RNA-seq DE tools leads to several technical problems including lower statistical power to detect

DE genes in scRNA-seq studies [15,16]. So, specialized scRNA-seq DE tools, *e.g.* ZINB-Wave [17], DEsingle [18], DECENT [19], etc. are developed based on the assumption that the observed scRNA-seq read counts follow a Zero Inflated Negative Binomial (ZINB) Distribution.

Let, Y_{ij}: *rv* representing the read (UMI) counts in scRNA-seq data of ith (i = 1, 2, …, N) gene of jth (j = 1, 2, …, M) cell; μ_{ij}: mean of ith gene of jth cell in i^{th} cell; θ_{ij} $(= \varphi_{ij}^{-1})$ and φ_{ij}: size and dispersion parameters respectively of ith gene in j^{th} cell for NB distribution; π_{ij}: zero inflation (*i.e.* the probability for a count to be an excess zero in a cell) parameter for i^{th} gene of jth cell. For any $\pi_{ij} \in [0,1]$, Y_{ij} is assumed to follow a ZINB distribution [15,18,19]. The PMF of the ZINB Distribution expressed as follows.

$$f_{ZINB}(y) = P\left[Y_{ij} = y\right] = \pi_{ij}\delta_0(y) + \left(1-\pi_{ij}\right)f_{NB}(y) \quad \forall\, y = 0,1,2,\ldots \tag{4}$$

where, $f_{NB}(.)$: PMF of NB distribution (Eq. 1); $\delta_0(.)$: Dirac's delta function. Here, $\delta_0(.)$ used to model the excess zeros in the data, and its PMF is expressed as:

$$\delta_0\left(Y_{ij} = y\right) := \begin{cases} 1; & y = 0 \\ 0; & y \neq 0 \end{cases} \tag{5}$$

Now, the PMF of the ZINB distribution to model the read counts from scRNA-seq data is given in Eq. 6.

$$P\left[Y_{ij} = y\right] = \begin{cases} \pi_{ij} + \left(1-\pi_{ij}\right)\left(\frac{\theta_{ij}}{\theta_{ij}+\mu_{ij}}\right)^{\theta_{ijk}} & when\, y = 0 \\ \left(1-\pi_{ijk}\right)\frac{G(y+\theta_{ij})}{G(y+1)G(\theta_{ij})}\left(\frac{\theta_{ij}}{\theta_{ij}+\mu_{ij}}\right)^{\theta_{ijk}}\left(\frac{\mu_{ij}}{\theta_{ij}+\mu_{ij}}\right)^{y}; & y > 0 \end{cases} \tag{6}$$

Now, $Y_{ij} \sim ZINB\left(\pi_{ij}, \mu_{ij}, \theta_{ij}\right)$, then the expected value and variance of Y_{ij} can be obtained as follows:

$$E\left(Y_{ij}\right) = \left(1-\pi_{ij}\right)\mu_{ij} \tag{7}$$

$$V\left(Y_{ij}\right) = \left(1-\pi_{ij}\right)\mu_{ij}\left(1 + \pi_{ij}\mu_{ij} + \frac{\mu_{ij}}{\theta_{ij}}\right) \tag{8}$$

If $\pi_{ij} = 0 \Rightarrow ZINB(\pi_{ij}, \mu_{ij}, \theta_{ij}) \rightarrow NB(\mu_{ij}, \theta_{ij})$

If $\varphi_{ij} \rightarrow 0$ $(No\ dispersion) \Rightarrow ZINB(\pi_{ij}, \mu_{ij}, \theta_{ij}) \rightarrow ZIP(\pi_{ij}, \mu_{ij})$

c. Poisson Distribution

Poisson Distributions (PD) are also extensively used for analysis of count data obtained from bulk RNA-seq or scRNA-seq experiments. The PMF of PD can be expressed as:

$$f_{PD}(y) = P[Y_{ij} = y] = \frac{e^{-\mu_{ij}}\mu_{ij}{}^{y}}{G(y+1)} \quad \forall\, y = 0, 1, 2, \ldots \tag{9}$$

$$E(Y_{ij}) = Var(Y_{ij}) = \mu_{ij} \tag{10}$$

d. ***Zero Inflated Poisson Distribution*** **(*ZIPD*)**

Poisson model has very strict assumptions, *i.e.,* mean equals the variance, which is often violated in scRNA-seq data analysis. When the variance is too large because there are many 0s as well as a few very high values for expression counts [20]. In this case, a better solution is often the ZIPD model.

The PMF of ZIPD distribution can be expressed as:

$$f_{ZIPD}(y) = P[Y_{ij} = y] = \pi_{ij} I(y = 0) + (1 - \pi_{ij}) f_{PD}(y) \qquad \forall\, y = 0, 1, 2, \ldots \tag{11}$$

$$= \begin{cases} \pi_{ij} + (1 - \pi_{ij}) e^{\mu_{ij}} & when\ y = 0 \\ (1 - \pi_{ij}) \frac{e^{-\mu_{ij}}\mu_{ij}{}^{y}}{G(y+1)}; & y > 0 \end{cases} \tag{12}$$

The mean and variance of ZIPD model is shown in Eq. 13 and 14, respectively.

$$E(Y) = (1 - \pi_{ij})\mu_{ij} \tag{13}$$

$$Var(Y) = (1 - \pi_{ij})\mu_{ij}(1 + \pi_{ij}\mu_{ij}) \tag{14}$$

e. Hermite Distribution

Hermite Distribution (HD) can be used to model the counts data [21]. Further, the PMF of HD is given in Eq. 15.

$$f_{HD}\left(Y_{ij}=y\middle|\alpha_{ij},\beta_{ij}\right)=e^{-(\alpha_{ij}+\beta_{ij})}\sum_{k=0}^{[\frac{y}{2}]}\frac{{\alpha_{ij}}^{y-2k}{\beta_{ij}}^{k}}{G(y-2k+1)G(k+1)}\ \forall\, y=0,1,2,\ldots \quad (15)$$

Further, the mean, variance, and dispersion index (*i.e.*, ratio between variance and mean) of rv Y_{ij}~HD (α,β) is given in Eq. 16 – 18.

$$E(Y_{ij})=f(\alpha_{ij},\beta_{ij})=(\alpha_{ij}+2\beta_{ij}) \quad (16)$$

$$Var(Y_{ij})=(\alpha_{ij}+4\beta_{ij}) \quad (17)$$

$$\varphi=g(\alpha_{ij},\beta_{ij})=1+2\beta_{ij}/(\alpha_{ij}+2\beta_{ij}) \quad (18)$$

The good-ness of fit of the above count data models, shown in Eq. 1, 6, 9, 12 and 15, were assessed through Akaike Information (AIC) and Bayesian Information (BIC) Criteria. The formula for AIC and BIC is expressed in Eq. 19 and 20.

$$AIC_m=-2logL_m+2P_m \quad (19)$$

$$BIC_m=-2logL_m+P_m log(M) \quad (20)$$

where, L_m: Likelihood function for m^{th} model; P_m: Number of parameters in m^{th} model; AIC_m and BIC_m: AIC and BIC values for m^{th} model; *M*: Total number of cells in the data.

Document S4. Normalization methods in scRNA-seq data analysis

a. Trimmed mean of M-values (TMM) Method

TMM normalization method originated from edgeR [22] to normalize the read (UMI) counts data. The details about the TMM can be found in Robinson and Oshlack, 2010 [23]. It deals with calculation of effective libraries sizes, which are then used as part of the per-sample normalization.

TMM normalization adjusts library sizes based on the assumption that most genes are not differentially expressed. Therefore, it is important not to make subsets of the count data before doing statistical analysis or visualization, as this can lead to differences being normalized away.

Algorithm

- 'log CPM' (Counts per Million) values are calculated for each gene. The CPM calculation uses the effective library sizes as calculated by the TMM normalization.
- After this first normalization, a second one is performed across samples for each gene: the counts for each gene are mean centered and scaled to unit variance.
- Genes or transcripts with zero expression across all samples or invalid values (NaN or +/- Infinity) are removed.

b. DESeq.norm Method

Modified median normalization method originated from DESeq to normalize the read (UMI) counts data. Let Y_{ij} denote the read counts of ith gene in j^{th} cell, then the size factor of jth cell is estimated by as follows:

$$\widehat{S_j} = \underset{i}{\text{median}} \frac{Y_{ij}}{\left(\prod_{v=1}^{M} Z_{iv}\right)^{1/M}} \quad (21)$$

where, $Y_{ij} \neq 0$ namely only the non-zero read counts of each gene are used. Finally, the normalized read counts of ith gene in j^{th} cell were computed through Eq. 40.

$$Y_{ij}^{norm} = \frac{Y_{ij}}{\widehat{S_j}} \quad (22)$$

<u>**Document S5:**</u> List of the methods and tools used in the comparative study.

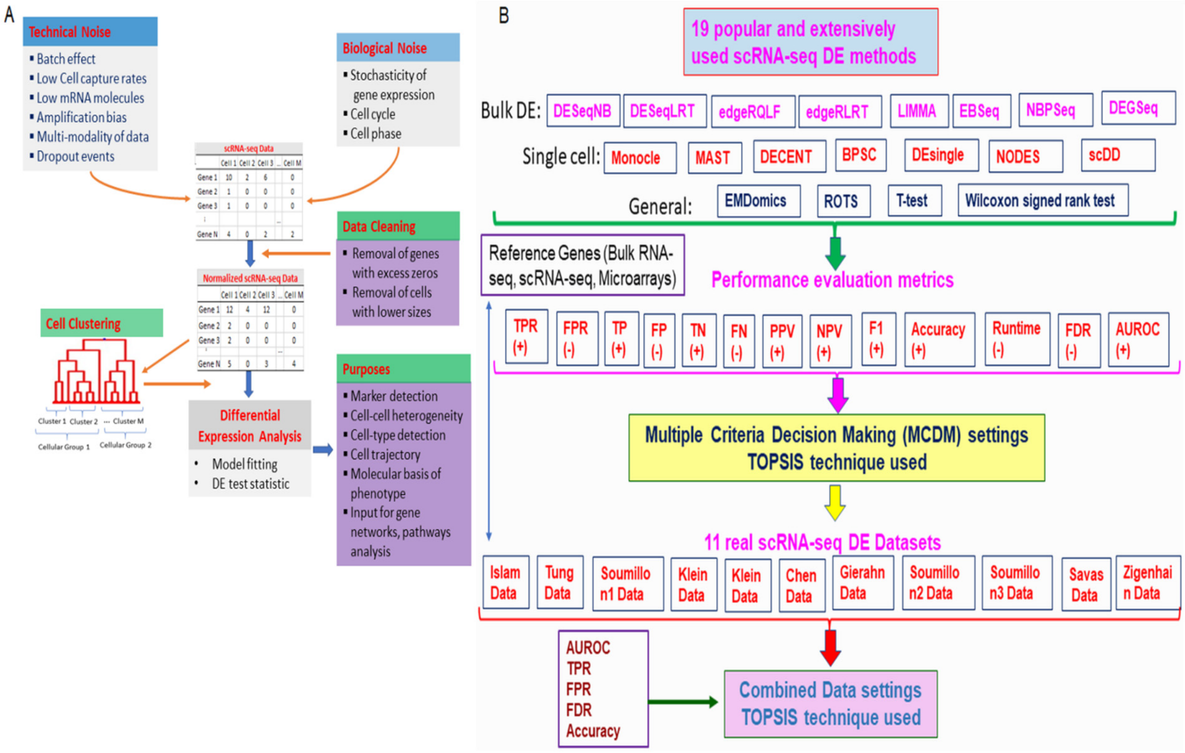

<u>Figure S1</u>. Schematic overview and layout of scRNA-seq DE analysis. (A) Schematic overview of the scRNA-seq DE analysis; (B) Layout of the comparative analysis of the DE tools used in this study. scRNA-seq data are inherently noisy with confounding factors, attributed as technical and biological source of variations. After sequencing, alignment and de-duplication are performed to quantify an initial gene expression profile matrix. Next, normalization is performed with raw expression data using various statistical methods to remove the amplification bias. Additional quality check can be performed when using spike-ins by inspecting the mapping ratio to discard low-quality cells. Finally, the normalized matrix is then subjected to main analysis through clustering of cells to identify subtypes. Cell trajectories can be inferred based on these data and by detecting differentially expressed genes between clusters. The identified genes can be further to unravel the biological processes of the underlying complex phenotypes through gene and cell-level analyses, as mentioned in Figure S1A.

Table S2. ScRNA-seq DE Tools details used in this study and their availability.

SN.	Methods	Year	R package	Version	Availability	Ref.
01	DESeqNB	2010	DESeq	1.39.0	https://bioconductor.org/packages/release/bioc/html/DESeq.html	[24]
02	DESeqLRT	2014	DESeq2	1.28.1	http://bioconductor.org/packages/release/bioc/html/DESeq2.html	[13]
03	DEGSeq	2009	DEGseq	1.42.0	https://bioconductor.org/packages/release/bioc/html/DEGseq.html	[25]
04	LIMMA	2002	limma	3.44.1	https://bioconductor.org/packages/release/bioc/html/limma.html	[26]

05	ROTS	2016	ROTS	1.16.0	https://www.bioconductor.org/packages/release/bioc/html/ROTS.html	[27]
06	edgeRLRT	2010	edgeR	3.30.3	https://bioconductor.org/packages/release/bioc/html/edgeR.html	[14]
07	edgeRQLF	2010	edgeR	3.30.3	https://bioconductor.org/packages/release/bioc/html/edgeR.html	[14]
08	NBSeq	2011	NBPSeq	0.3.0	https://cran.rstudio.com/web/packages/NBPSeq	[28]
09	EBSeq	2013	EBSeq	1.28.0	https://www.bioconductor.org/packages/release/bioc/html/EBSeq.html	[29]
10	T-test	1947	stats	4.0.2	https://cran.r-project.org/web/packages/STAT/index.html	[30]
11	Wilcox	1945	stats	4.0.2	https://cran.r-project.org/web/packages/STAT/index.html	[31]
12	Monocle	2017	monocle	2.16.0	www.bioconductor.org/packages/release/bioc/html/monocle.html	[32]
13	MAST	2015	MAST	1.14.0	https://github.com/RGLab/MAST	[33]
14	scDD	2016	scDD	1.12.0	https://www.bioconductor.org/packages/release/bioc/html/scDD.html	[34]
15	DEsingle	2018	DEsingle	1.8.2	https://www.bioconductor.org/packages/release/bioc/html/DEsingle.html	[18]
16	DECENT	2019	DECENT	1.1.0	https://github.com/cz-ye/DECENT	[19]
17	BPSC	2016	BPSC	0.99.2	https://github.com/nghiavtr/BPSC	[35]
18	NODES	2016	NODES	0.0.0.9010	https://goo.gl/Ndx07M	[36]
19	EMDomics	2016	EMDomics	2.18.0	https://www.bioconductor.org/packages/release/bioc/html/EMDomics.html	[37]

SN.: Serial Number; Parameters: Parameter settings used for analysis; Approach: Type of approach based on the underlying assumptions of the models; Year: Year of first release of the tool; Version: Latest version of the R package used in this study; Wilcox: Wilcox signed rank test

Table S3. List of the scRNA-seq DE methods along with the parameters used in this comparative study.

SN.	Methods	Parameters	Approach	DE Stat.	Run time	Utility	Ref.
01	DESeqNB	Defaults	Parametric	NB test	Minutes	Bulk	[24]
02	DESeqLRT	Defaults	Parametric	LRT	Minutes	Bulk	[13]
03	DEGSeq	Method=”LRT”	Parametric	Z-test	Minutes	Bulk	[25]
04	LIMMA	Linear model	Parametric	Wald test	Minutes	Bulk	[26]
05	ROTS	B = 1000, K = 6000	Non-Parametric	Z-test	Hours	Bulk	[27]
06	edgeRLRT	Defaults	Parametric	LRT	Minutes	Bulk	[14]
07	edgeRQLF		Parametric	QLF	Minutes	Bulk	[14]
08	NBSeq	model.disp = "NBQ"	Parametric	Exact test	Minutes	Bulk	[28]
09	EBSeq	sizeFactors = MedianNorm(.), maxround = 50	Parametric	Posterior prob.	Hours	Bulk	[29]
10	T-test	alternative = "two.sided",	Parametric	t-stat.	Seconds	Gen	[30]

		paired = FALSE, var.equal = FALSE					
11	Wilcox	alternative = "two.sided", paired = FALSE, var.equal = FALSE	Non-Parametric	M-W stat	Seconds	Gen	[31]
12	Monocle	UMI=T, cores = n.cores, verbose = T	Parametric	LRT	Minutes	Single cell	[32]
13	MAST	Defaults	Parametric	LRT	Minutes	Single cell	[33]
14	scDD	alpha=0.01, mu0=0, s0=0.01, a0=0.01, b0=0.01, UMI=T	Parametric	Bayesian stat.	Minutes	Single cell	[34]
15	DEsingle	parallel = FALSE, Defaults	Parametric	LRT	Hours	Single cell	[18]
16	DECENT	use.spikes = F, CE.range = c(0.02, 0.1), parallel = FALSE	Parametric	LRT	Days	Single cell	[19]
17	BPSC	coef=2, estIntPar=FALSE	Parametric	Z-test	Hours	Single cell	[35]
18	NODES	r = 20, smooth_points = 10000, zper = 0.5	Non-Parametric	Wilcoxon	Minutes	Single cell	[36]
19	EMDomics	nperm = 100, pairwise.p = TRUE, parallel=FALSE	Non-Parametric	Distance measure	Hours	Single cell	[37]

SN.: Serial Number; Parameters: Parameter settings used for analysis; Approach: Type of approach based on the underlying assumptions of the models; Year: Year of first release of the tool; Version: Latest version of the R package used in this study.

Table S4. List of the criteria used for comparative performance analysis of the scRNA-seq DE methods.

Sl. No.	Symbol	Criteria	Impact
01	C1	True Positive (TP)	+
02	C2	False Positive (FP)	-
03	C3	True Negative (TN)	+
04	C4	False Negative (FN)	-
05	C5	True Positive Rate (TPR)	+
06	C6	False Positive Rate (FPR)	-
07	C7	False Discovery Rate (FDR)	-
08	C8	Positive Prediction Rate (PPR)	+
09	C9	Negative Prediction Value (NPV)	+
10	C10	F1 Score	+
11	C11	Accuracy	+
12	C12	Area under Receiver Operating Curve (AUROC)	+
13	C13	Run Time	-
14	C14	Multiple Criteria Decision-Making setup	All C1-C13

C1-C13: symbols of the criteria

The expressions for these criteria are given in the main text of the manuscript (Eq. 30–37). The impact of the criteria on performance of the tested methods (Tables S2, S3) are shown as "+" and

"-". Here, the "+" indicates the criteria has positive impact, means higher the values of the criteria better are that method and *vice-versa*. Further, "-" indicates that higher value of the criteria has negative impact on the performance of the tested methods and *vice-versa*. In other words, "+" represents higher the value of the criterion, better is the method and *vice-versa*; "-" in impact represents lower the value of the criterion, better is the method and *vice-versa*. The positive impact criteria are {C1, C3, C5, C8, C9, C10, C11, C12} and the negative impact criteria are {C2, C4, C6, C7, C13}. Under the individual criterion-centric performance evaluation, the performance of the tested methods is evaluated based on the criterion (C1-C13) separately. Furthermore, under Multiple Criteria Decision-Making (MCDM) setting, all the criteria are considered simultaneously for performance analysis of the tested methods, given in Tables S2 and S3.

<u>Document S6.</u> Comparative performance analysis of the tested methods using TOPSIS technique under Multiple Criteria Decision-Making setup.

Multiple Criteria decision-making (MCDM) is a sub-discipline of operations research that explicitly evaluates multiple conflicting criteria in decision making. Conflicting criteria are typical in evaluating options: runtime (C13) or error rate (C7) or Accuracy (C11) are usually considered to evaluate the performance of the DE methods. In selecting a best method, Accuracy (C11), AUROC (C12), FDR (C7) may be some of the main criteria usually considered in single-cell studies. In DE analysis, researchers are more interested finding the DE methods with higher accuracy while simultaneously reducing error and computational time. In a typical analysis, researcher may be interested in a method with higher accuracy and lower computational time required, which are fundamentally conflicting criteria.

Therefore, in making the decision of selecting the best method for DE analysis of scRNA-seq data, it is pertinent to undertake the performance analysis of the tested methods under the

simultaneous consideration of all the conflicting criteria. Here, we emphasized to comparative performance analysis of the 19 methods under the simultaneous consideration of all the 13 criteria (Table S6). In operational research, such a performance evaluation setting is called as MCDM setup [38], where the main idea is to consider a set of criteria and choose the best performing method over a list of methods [39]. Under this MCDM set up, Technique for Order Performance by Similarity to Ideal Solution (TOPSIS) [40] has been extensively [41]. However, we used this approach for the first time in single-cell data analytics. Here, the basic idea is to choose the best method out of the 19 tested methods based on the simultaneous consideration of the 13 decision criteria (Table S6). Through TOPSIS, it is expected that the best identified method should have shortest geometric distance from the positive ideal solution (PIS) and the longest geometric distance from the negative ideal solution (NIS) [42]. The detailed method and major analytical steps for the MCDM-TOPSIS analysis are given as follows.

Let $\boldsymbol{U}$ be the resultant decision matrix used under MCDM setup, *i.e.,* $\boldsymbol{U} = ((u_{rs}))$, where u_{rs} represents the value of M_r (r^{th} method) (r = 1, 2, …, 19) under C_s (s^{th} decision criteria) (s = 1, 2, …, 13) and W_s 's are the criteria weights indicate the relative importance among them. Further, the W_s are calculated using the entropy technique through the following steps.

Step 1: Normalization of the decision matrix ($\boldsymbol{U}$)**:** The resulted values in $\boldsymbol{U}$ are first transformed to normalized values (P_{rs}) through using Eq. 23.

$$P_{rs} = {u_{rs}}/{\sum_{r=1}^{19} u_{rs}} \tag{23}$$

Step 2: Calculation of entropy measure (E_s) for s^{th} criterion is calculated using Eq. 24

$$E_s = -a \sum_{r=1}^{13} P_{rs} ln\,(P_{rs}) \tag{24}$$

where $a = 1/ln19$. Further, the degree of diversity (D_s) for s^{th} criterion is computed using Eq. 25.

$$D_s = 1 - E_s \tag{25}$$

Step 3: Calculation of weights (W_s) for each criterion: W_s are computed for s^{th} criterion through using Eq. 26.

$$W_s = D_s / \sum_{s=1}^{13} D_s \tag{26}$$

After obtaining criteria weights, they are incorporated in the usual TOPSIS technique to calculate the overall scores for each tested method. The major steps for the TOPSIS technique in this context are briefly given as:

[**1**] Construct the normalized decision matrix (**Z**) by vector normalization using Eq. 27.

$$z_{rs} = {}^{u_{rs}}\!/_{\sum_r u_{rs}^2} \tag{27}$$

[**2**] Calculate weighted normalized decision matrix using:

$$v_{rs} = W_s \times z_{rs} \tag{28}$$

[**3**] Determine the PIS, V^+, and NIS, V^-, by using Eq. 29 and 30.

$$V^+ = \{V_1^+, V_2^+, \ldots, V_S^+\} = \{\langle \max(v_{rs} | r = 1,2,\ldots,19) | s \in C_- \rangle, \langle \min(v_{rs} | r = 1,2,\ldots,19) | s \in C_+ \rangle\} \tag{29}$$

$$V^- = \{V_1^-, V_2^-, \ldots, V_S^-\} = \{\langle \max(v_{rs} | r = 1,2,\ldots,19) | s \in C_+ \rangle, \langle \min(v_{rs} | r = 1,2,\ldots,19) | s \in C_- \rangle\} \tag{30}$$

where $C_+ = \{s = 1,2,\ldots,8 | s$ associated with the criteria having a positive impact and $C_- = \{s = 1,2,\ldots,5 | s$ associated with the criteria having a negative impact.

[**4**] Calculate the L_2 distance for PIS (d_r^+) and NIS (d_r^-) using:

$$d_r^+ = \left(\sum_{s=1}^{8} (v_{rs} - V_s^+)^2\right)^{1/2} \tag{31}$$

$$d_r^- = \left(\sum_{s=1}^{5} (v_{rs} - V_s^-)^2\right)^{\frac{1}{2}} \tag{32}$$

[**5**] Determine the relative closeness of the tested method to the ideal solution using Eq. 33.

$$R_r = \frac{d_r^-}{d_r^- + d_r^+} \quad \forall\, r = 1,2,\ldots,19 \tag{33}$$

Through Eq. 33, the methods with higher R_r ($0 \leq R_r \leq 1$) are preferred and considered as better over the multiple criteria and *vice-versa*.

Document S7: Testing for zero-inflation parameters for genes in scRNA-seq data

Here, we assume that the UMI (read) counts of the genes from a scRNA-seq study are generated through a ZINB population model given in Eq. 4 and 6. In order to test the statistical significance of the zero inflation parameters of ith gene π_i of the ZINB model, we adopt the following Generalized Likelihood Ratio Test (GLRT) procedure. Here, for the testing purpose, we define the following null hypothesis.

$$H_0: \pi_i = 0 \ vs. \ H_1: \pi_i \neq 0$$

where, H_0: null hypothesis; H_1: alternate hypothesis. In other words, null hypothesis tells us that k^{th} gene is not zero inflated, and subsequently, the scRNA-seq data structure is same as RNA-seq data. Further, if we fail to reject H_0, then the RNA-seq DE tools can be used for DE analysis of scRNA-seq data with the expectation of satisfactory results.

The above-mentioned test, $H_0 \ vs. H_1$, can be tested through GLRT and the test statistic is given in Eq. 34.

$$-2ln\alpha = -2\{l(\boldsymbol{\Omega}_i = \widehat{\boldsymbol{\Omega}}_{i0}; Y_{ij}) - l(\boldsymbol{\Omega}_i = \widehat{\boldsymbol{\Omega}}_i; Y_{ij})\} \qquad (34)$$

where, $\widehat{\boldsymbol{\Omega}}_{i0}$: Maximum Likelihood Estimator (MLE) of $\boldsymbol{\Omega}_i$ for i^{th} gene under the constraint of H_0 and $\widehat{\boldsymbol{\Omega}}_i$: unconstrained MLE of $\boldsymbol{\Omega}_i$ for i^{th} gene, $\boldsymbol{\Omega}_i$: parametric space for i^{th} gene, *i.e.,* $\boldsymbol{\Omega}_i = \{\mu_i, \theta_i, \pi_i\}$. The test statistic in Eq. 34 is asymptotically distributed as Chi-square distribution with 1 degree of freedom under H_0.

We applied the above procedure to Tung et al.'s scRNA-seq data to test the statistical significance of the zero inflation parameters of genes. The results are shown in below Figure. It can be observed that most of the genes in scRNA-seq data are found to be zero inflated as their

corresponding *p-values* are less than the level of significance value. This finding motivates us to develop a statistical approach for testing of differential zero inflation of genes.

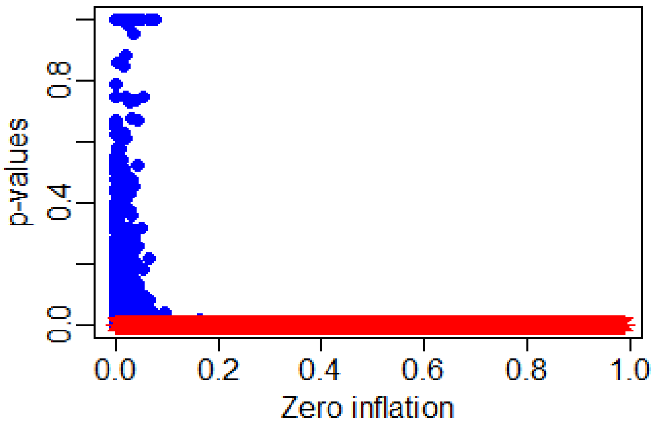

Plotting of estimated value of zero inflation parameter and their corresponding *p-values*. X-axis represents estimated values of zero inflation, (higher value of zero inflation parameter means a greater number of zeros found in the expression vector of that gene) and Y-axis represents the computed statistical significance value for the zero-inflation parameter, lesser the value represents the gene is more zero inflated.

<u>Document S8</u>: Statistical testing for overdispersion parameters in scRNA-seq data

For testing the statistical significance of the dispersion parameter of ith gene θ_i of the ZINB model, we adopt the following GLRT procedure. Here, for the testing purpose, we define the following null hypothesis.

$$H_0: \theta_i = 0 \ vs. \ H_1: \theta_i \neq 0$$

where, H_0: null hypothesis; H_1: alternate hypothesis. In other words, null hypothesis tells us that i^{th} gene is not dispersed, means the mean is same as the variance and subsequently, the scRNA-seq count data is obtained from a Poisson model. Further, if we fail to reject H_0, then we can say UMI counts scRNA-seq data is not overdispersed and simply fitting a Poisson model will give satisfactory results.

The above-mentioned test, $H_0 \ vs. H_1$, can be tested through GLRT and the test statistic is given in Eq. 35.

$$-2ln\alpha = -2\{l(\boldsymbol{\Omega}_i = \widehat{\boldsymbol{\Omega}}_{i0};\ Y_{ij}) - l(\boldsymbol{\Omega}_i = \widehat{\boldsymbol{\Omega}}_i;\ Y_{ij})\} \quad (35)$$

where, $\widehat{\boldsymbol{\Omega}}_{i0}$: MLE of $\boldsymbol{\Omega}_i$ for i^{th} gene under the constraint of H_0 and $\widehat{\boldsymbol{\Omega}}_i$: unconstrained MLE of $\boldsymbol{\Omega}_i$ for i^{th} gene, $\boldsymbol{\Omega}_i$: parametric space for i^{th} gene, *i.e.,* $\boldsymbol{\Omega}_i = \{\mu_i, \theta_i, \pi_i\}$. The test statistic in Eq. 35 is asymptotically distributed as Chi-square distribution with 1 degree of freedom under H_0.

We applied the above procedure to Tung et al.'s scRNA-seq data to test the statistical significance of the dispersion parameters of genes. The results are shown in below Figure. It can be observed that all the genes in Tung's scRNA-seq data is found to be zero inflated as their corresponding *p-values* are less than the level of significance value (at alpha = 0.0001) (below Figure). This finding is well reported in literature.

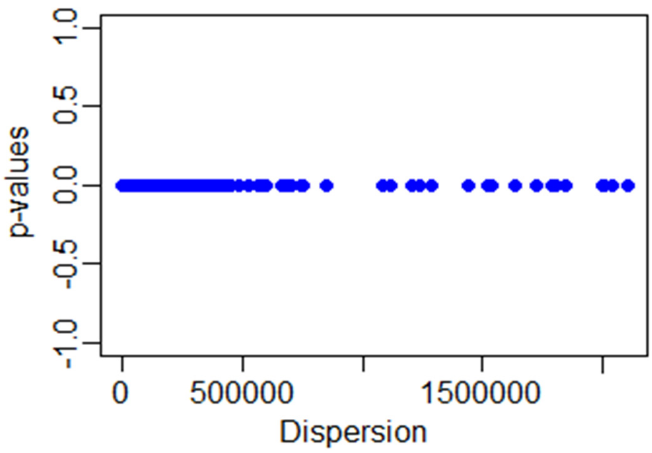

Testing for statistical significance of dispersion parameters. X-axis represents estimated values of overdispersion parameter through a ZINB model, and Y-axis represents the computed statistical significance value for the overdispersion parameter, lesser the value represents the gene is more overdispersed.

<u>Document S9</u>: Application of Count Data Models to Zero-Inflated and Overdispersed Real Datasets

In this section, we discuss about the fitting and suitability of different count data models such as NB, ZINB, PD, ZIPD and HD to the zero inflated and over dispersed datasets. These data set include, Embryonic Mouse Cysts count data and scRNA-seq UMI read counts data of a single gene, *i.e.* ENSG00000162585 from Tung's data [2].

A. *Embryonic Mouse Cysts Data*

Earlier experimental studies have shown that the scRNA-seq (UMI) read count data is zero inflated and over dispersed [1,43–48]. Hence, we consider a published zero-inflated and over-dispersed data on counts of cysts in embryonic mouse [49] to study the suitability of different discrete models. Here, we consider data on counts of cysts in embryonic mouse kidneys which had been subjected to steroids, taken from McElduff et al. [49]. This data reveals the details of the effect of a low protein diet in mice on kidney development in their offspring. Data on counts of cysts in embryonic mouse kidneys which had been subjected to steroid were featured in this study. Then, the count data models, such as NBD, ZINB, PD, ZIPD and HD are fitted on this data. Further, the parameters of these models are estimated through Maximum Likelihood Estimation (MLE) method. The observed frequencies and expected frequencies from different count models along with their estimated parameters are shown in Table S5. The goodness of fit of the above models to this experimental data is assessed through AIC and BIC.

Table S5. Fitting of well-known discrete models to over-dispersed and zero-inflated cyst count data.

Read	Obs. Freq.	Exp. Freq. NBD	Exp. Freq. ZINBD	Exp. Freq. PD	Exp. Freq. ZIPD	Exp. Freq. HD
0	65	63.29	64.99	25.1	65.03	45.36
1	14	17.56	14.01	37.32	5.1	13.75
2	10	8.98	9.11	27.74	8.87	28.92
3	6	5.72	6.27	13.74	10.28	8.35
4	4	3.91	4.44	5.11	8.93	9.19
5	2	2.79	3.2	1.52	6.21	2.53
6	2	2.04	2.33	0.38	3.6	1.94
7	2	1.52	1.71	0.08	1.79	0.51
8	1	1.15	1.26	0.01	0.78	0.31
9	1	0.88	0.93	0	0.3	0.08
10	1	0.68	0.69	0	0.1	0.04
11	2	0.52	0.52	0	0.03	0.01
12	1	0.41	0.38	0	0.01	0
Total	111	110.95	110.84	111	111.03	110.99

Parameters (MLE)	μ=1.49 θ=0.31	$\mu = 2.285$ $\theta = 0.698$ $\pi = 0.349$	$\mu = 1.486$	$\mu = 3.476$ $\pi = 0.572$	$\mu = 1.487$ $\varphi = 1.796$
#Parameters	2	3	1	2	2
Likelihood	-175.22	-172.8	-263.25	-191.9	-202.84
AIC	354.44	351.60	528.50	387.80	409.68
BIC	354.53	351.74	528.55	387.89	409.77

#Parameters: number of parameters; μ: Mean; θ: size; π: zero-inflation probability; φ: dispersion index (ratio of variance to mean); AIC: Akaike Information Criterion; BIC: Bayesian Information Criterion; Obs. Freq: Observed Frequency; Exp. Freq. NBD: computed expected frequency through NB model; Exp. Freq. ZINB: computed expected frequency through ZINB model; Exp. Freq. PD: computed expected frequency through Poisson model; Exp. Freq. ZIPD: computed expected frequency through ZIPD model; Exp. Freq. HD: computed expected frequency through HD model

It is observed that the expected frequencies computed from ZINB are closer to their observed values as compared to other models. Further, the AIC and BIC values (Eq. 19, 20) for ZINB is lowest followed by NB model for the given zero inflated and over dispersed cyst count data as compared to PD, ZIPD and HD (Table S5). This indicates, for fitting over-dispersed and zero inflated datasets like scRNA-seq data, ZINB model provides a better fit as compared to other count models, *i.e.,* NB, PD, ZIPD and HD (Table S5). Moreover, we validate the above claim by using another overdispersed and zero-inflated dataset from scRNA-seq study.

B. Application to scRNA-seq read counts data

Here, we fitted the considered count data models, such as NB, ZINB, PD, ZIPD and HD to the scRNA-seq read counts of ENSG00000162585 gene taken from Tung's data (available at https://github.com/jdblischak/singleCellSeq). The observed and expected frequencies computed through different count models for each read sequences along with estimated values of the parameters are shown in Table S6.

Table S6. Fitting of well-known discrete models to over-dispersed and zero-inflated scRNA-seq read count data.

**UMI Reads**	**Obs. Freq.**	**Pred. Freq. NB**	**Pred. Freq. PD**	**Pred. Freq. HD**	**Pred. Freq. ZINB**	**Pred. Freq. ZIP**

0	115	108.05	0.09	4.82	126.82	115
1	84	57.92	0.78	3.34	56.96	0.06
2	45	42.58	3.37	20.33	39.79	0.36
3	33	34.13	9.73	13.54	31.11	1.34
4	31	28.48	21.03	42.8	25.61	3.73
5	18	24.34	36.39	27.48	21.73	8.32
6	12	21.13	52.46	59.94	18.79	15.44
7	10	18.53	64.83	37.17	16.48	24.56
8	7	16.39	70.11	62.84	14.6	34.19
9	9	14.59	67.38	37.71	13.03	42.31
10	4	13.05	58.29	52.62	11.7	47.12
11	6	11.72	45.84	30.59	10.56	47.71
12	12	10.56	33.04	36.67	9.58	44.28
13	4	9.54	21.99	20.68	8.71	37.94
14	3	8.64	13.59	21.87	7.95	30.18
15	5	7.84	7.84	11.98	7.27	22.41
16	8	7.13	4.24	11.39	6.67	15.6
17	6	6.5	2.16	6.07	6.13	10.22
18	4	5.93	1.04	5.27	5.64	6.32
19	7	5.41	0.47	2.74	5.2	3.71
20	5	4.95	0.2	2.19	4.8	2.06
21	5	4.53	0.08	1.11	4.43	1.09
22	4	4.15	0.03	0.83	4.1	0.55
23	5	3.8	0.01	0.41	3.8	0.27
24	6	3.49	0	0.29	3.53	0.12
25	4	3.21	0	0.14	3.27	0.06
26	7	2.95	0	0.09	3.04	0.02
27	5	2.71	0	0.04	2.83	0.01
28	5	2.49	0	0.03	2.63	0
29	3	2.29	0	0.01	2.45	0
30	2	2.11	0	0.01	2.28	0
31	2	1.95	0	0	2.12	0
33	5	1.65	0	0	1.85	0
34	5	1.53	0	0	1.72	0
35	7	1.41	0	0	1.61	0
36	3	1.3	0	0	1.5	0
39	4	1.03	0	0	1.23	0
40	2	0.95	0	0	1.15	0
41	1	0.88	0	0	1.08	0
42	3	0.81	0	0	1.01	0
43	1	0.75	0	0	0.94	0
46	3	0.59	0	0	0.78	0
47	1	0.55	0	0	0.73	0

49	2	0.47	0	0	0.64	0
50	2	0.44	0	0	0.6	0
Parameters (MLE)		μ=8.14 θ=0.574	μ=8.65	μ=8.651 φ=1.92	μ=8.652 θ=0.47377 π=1.173e-05	μ=11.1373 π=0.224

#Parameters: parameters estimated through MLE; μ: Mean; θ: size; π: zero-inflation probability; φ: dispersion index (ratio of variance to mean); Obs. Freq: Observed Frequency; Pred. Freq. NBD: computed predicted frequency through NB model; Pred. Freq. ZINB: computed predicted frequency through ZINB model; Pred. Freq. PD: computed predicted frequency through Poisson model; Pred. Freq. ZIPD: computed predicted frequency through ZIPD model; Pred. Freq. HD: computed predicted frequency through HD model

It is observed that the expected frequencies computed from ZINB model are closer to their observed counter parts as compared to other models, such as NB, PD, ZIPD and HD (Table S6). Further, the fitting of the discrete models in terms of density plots are shown in Figure S2. This indicates, for fitting overdispersed and zero inflated scRNA-seq data, ZINB model provides a better fit to the observed scRNA-seq data as compared to other count models (Tables S6, Figure S2). Therefore, from the above applications of discrete models to zero-inflated and overdispersed scRNA-seq data, we can conclude that the ZINB model provides better fit to the data and better estimates of the parameters as compared to NB model. In other words, NB model is extensively used for modeling and fitting of bulk RNA-seq count data. But it performed poor when it is used for fitting the scRNA-seq data, which is simultaneously zero inflated and overdispersed (Figure S2). It can be observed that, for fitting overdispersed and zero inflated datasets like scRNA-seq data, ZINB model provides a better estimate of the mean and dispersion parameters as compared to other count data models (Table S6, Figure S2). More specifically, we test the ability of NB, and ZINB models to estimate the mean and dispersion parameters for scRNA-seq count data through simulation, which is described in the following section.

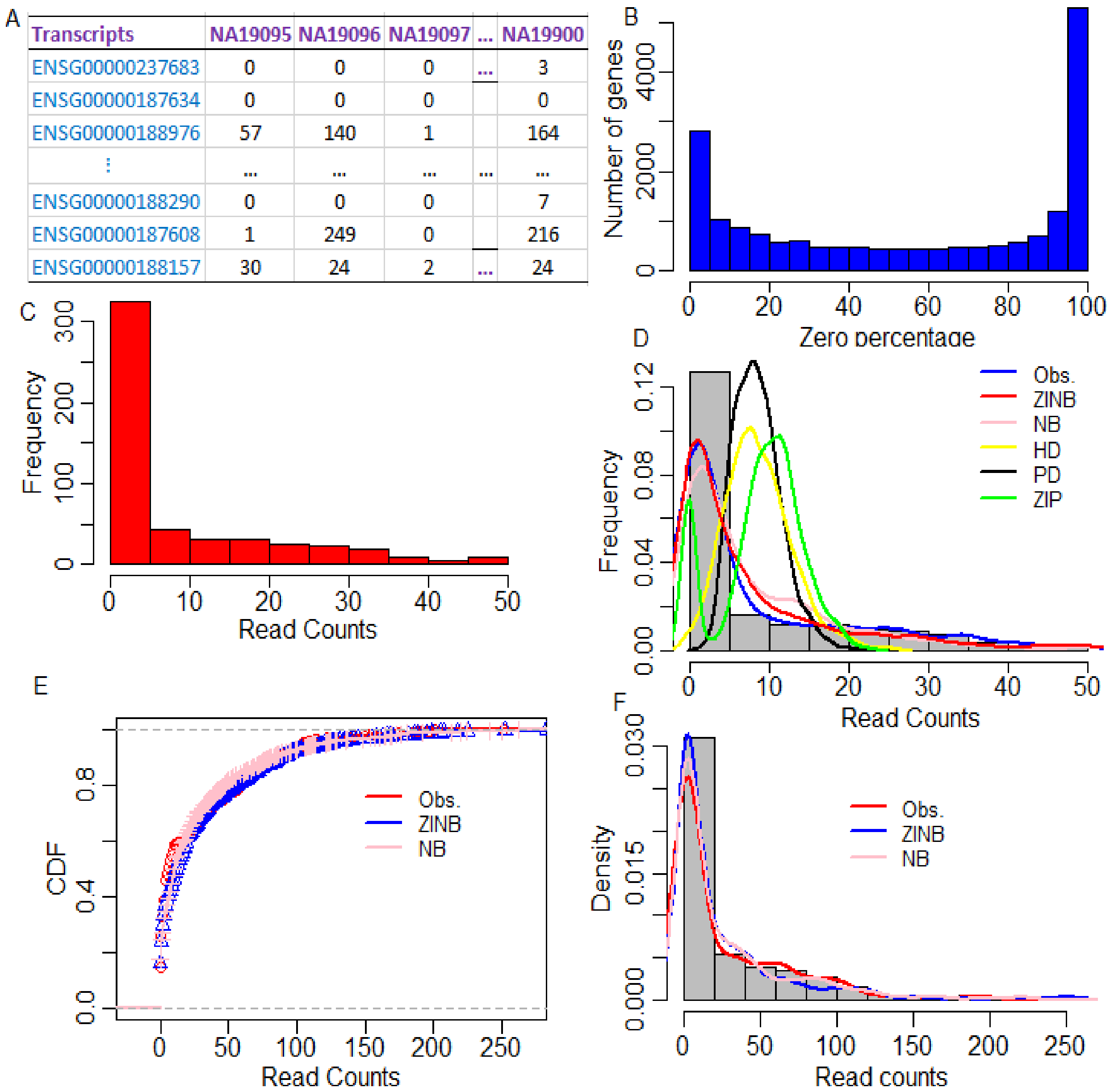

Figure S2. Data Characteristics, Distributions and Fitting of Various Discrete Data Models. (A) Glimpse of the scRNA-seq (UMI) read count data matrix. Here, rows represent the genes and columns represent the cell lines. The values represent the number of read of sequences mapped to each gene. (B) Distribution of zero percentages of genes in scRNA-seq data. X-axis represents the various zero percentages and Y-axis represents the number of genes. Here, the approx. 5000 genes have ~100 % zeros across all the cells, which indicates of zero-inflation in scRNA-seq data. (C) Distribution of scRNA-seq read counts of ENSG00000176022 gene (from Tung's data). X-axis represents the reads and Y-axis represents the frequency of the reads. (D) Fitting of Various Discrete Data Models to scRNA-seq read counts of ENSG00000176022 gene (from Tung's data). X-axis represents the read counts and Y-axis represents the density of read counts. The fitting of observed density and densities from the Negative Binomial (NB), Zero Inflated Negative Binomial (ZINB), Poisson Distribution (PD), Zero Inflated Poisson (ZIP) and Hermite Distribution (HD) to the observed data are shown in different colors. (E) Cumulative Distribution Function (CDF) plot for scRNA-seq data of ENSG00000176022 gene (from Tung's data). Here, X-axis represents the read counts and Y-axis represents the cumulative density of read counts. Observed CDF, and CDFs from NB and ZINB models are

shown in blue, pink, and red colors, respectively. (F) Density plots for scRNA-seq data of ENSG00000176022 gene (from Tung's data). Here, X-axis represents the read counts and Y-axis represents the density of read counts. Observed density plot, and density plots from NB and ZINB models are shown in blue, pink, and red colors, respectively.

Document S10. Comparative analysis of NB and ZINB models

NB model is implemented in popular RNA-seq DE tools such as DESeq2, edgeR, baySeq, cuffdiff, [13,14,50,51] *etc*. for analysis of RNA-seq count data. Such datasets are overdispersed but may not zero-inflated. It is pertinent to test its performance on scRNA-seq datasets, which is both zero inflated and overdispersed. So, we took scRNA-seq read counts of ENSG00000176022 gene over 576 cells from Tung's data available at www.github.com/jdblischak/singleCellSeq and NCBI's Gene Expression Omnibus database with GEO Series accession number GSE77288 (http://www.ncbi.nlm.nih.gov/geo/query/acc.cgi?acc=GSE77288) [2]. The distribution (*i.e.*, histogram) of scRNA-seq read counts of ENSG00000176022 gene is shown in Figure S2C. The fitting of NB and ZINB models to this observed scRNA-seq data is shown in Figures S2E and F. Then, we estimated the parameters of the ZINB model for this gene through executing the *zeroinfl* function implemented in pscl R package [52] and the results are shown in Table S7.

We applied NB and ZINB models to test their ability estimate the mean and dispersion parameters for scRNA-seq count data under an artificial setup. For this purpose, parameter estimates for ENSG00000176022 gene is used to simulate count expression data through ZINB model given in Eq. 5. Here, we simulated the zero inflated and overdispersed count data of the gene over 500 cells. Then both the models, such as NB and ZINB are applied on these simulated datasets to estimate the parameters through MLE. The simulation is repeated over 100 times to generate 100 scRNA-seq artificial datasets and both the NB and ZINB models are fitted to estimate their parameters. For this purpose, we executed *zeroinfl* and *glm.nb* functions implemented in pscl [52] and MASS statistical R packages respectively. The results are shown in Table S7.

Table S7. Comparative analysis of NB and ZINB models for estimation of parameters from scRNA-seq data.

		NB Model				ZINB Model			
**Parameters**	**True values**	**Estimate**	**Bias**	**MSE**	**95% CI**	**Estimate**	**Bias**	**MSE**	**95% CI**
**Mean (μ)**	115.21	65.981	-49.229	1.61	(56.24, 75.71)	115.252	0.042	1.261	(112.78, 117.70)
**Dispersion (θ^{-1})**	3.322	5.3236	1.865	0.38	(2.284, 2.364)	3.244	0.078	0.057	(2.658, 3.829)
**Zero inflation prob. (π)**	0.432	-	-	-	-	0.430	0.002	0.003	(0.422, 0.445)

Number of cells: 500; number of simulations: 100; MSE: Mean Standard Error; CI: Confidence Interval

Our analysis indicates that NB model underestimate the mean and over-estimate the dispersion parameters for scRNA-seq data (Table S7). The ZINB model provides better estimates of mean and dispersion, which are close to their true values for scRNA-seq data. Further, ZINB model has lower bias and MSE as compared to NB model (Table S7). It is interesting to note that 95% confidence interval of parameters for NB model is quite wider and do not contain the true values of the parameters. While this observation is quite satisfactory for ZINB model (Table S7). This indicates the better suitability of ZINB model for modeling the zero-inflated and over-dispersed scRNA-seq count data and provides better estimates of the parameters. The reason may be attributed as NB model thus accommodates excess zeros by underestimating the mean and overestimating the dispersion parameters. This phenomenon jeopardizes the statistical power of NB distribution-based tools to discover DE genes in the presence of zero-inflation, when applied to scRNA-seq data.

Document S11. Performance evaluation of DE methods based on runtime criterion.

We used the run time criterion (C13) (Table S4) to evaluate the performance of the tested DE methods given in Tables S2 and S3. Here, the run time refers to the amount of time required to get the informative Differentially Expressed Genes (DEG) of particular size through providing the

gene expression data as input to the R functions of the respective methods. Here, we performed all analyses on a standard 16 GB RAM Dell PC with Intel(R) Core (TM) i3-6100U CPU @ 2.30GHz, 2301 Mhz, 2 Core(s) Processor(s) and recorded the computational time required to analyze a gene expression data of 10,000 genes with 500 cells (cell group 1: 250 and cell group 2: 250). The scores of the tested methods based on the runtime criterion can be computed through Eq. 36 and the results are shown in following Table.

Let, p_r be the rank of rth DE method based on the computational time required to get the DE gene set of size from the data having 10000 genes with 200 cells. The rank score for r^{th} DE method can be calculated by using:

$$score_r = f(p_r) = \frac{1+M-p_r}{M} \tag{36}$$

where, $score_r$ is the rank score for r^{th} DE method, M is the total number of methods used in the analysis. The result from the runtime-based analysis of scRNA-seq DE methods (Tables S2, S3) is shown in following Table.

Runtime based analysis of the tested scRNA-seq DE methods.

Sl. No.	Methods	Run Time	Ranks (p_r)	Rank score ($score_r$)
1	BPSC	20 Min.	9	0.57894737
2	DECENT	20 hr. 15 Min.	19	0.05263158
3	DEGSeq	10 Min.	3	0.89473684
4	DESeqNB	16.5 Min.	6	0.73684211
5	DESeqLRT	16 Min.	5	0.78947368
6	DEsingle	12 hr. 20 Min	18	0.10526316
7	EBSeq	9 hr. 10 Min.	17	0.15789474
8	edgeRLRT	19 Min.	8	0.63157895
9	edgeRQLF	18 Min.	7	0.68421053
10	EMDomics	8 hr. 15 Min	16	0.21052632
11	LIMMA	14 Min.	4	0.84210526
12	MAST	22 Min.	11	0.47368421
13	Monocle	23 Min.	12	0.42105263
14	NBSeq	25 Min.	14	0.31578947
15	NODES	24 Min.	13	0.36842105

16	ROTS	27 Min.	15	0.26315789
17	scDD	20 Min.	10	0.52631579
18	T-test	8 Min.	1	1
19	Wilcox	9 Min.	2	0.94736842

hr. Hours requires to analyze the data; Min.: Minutes required

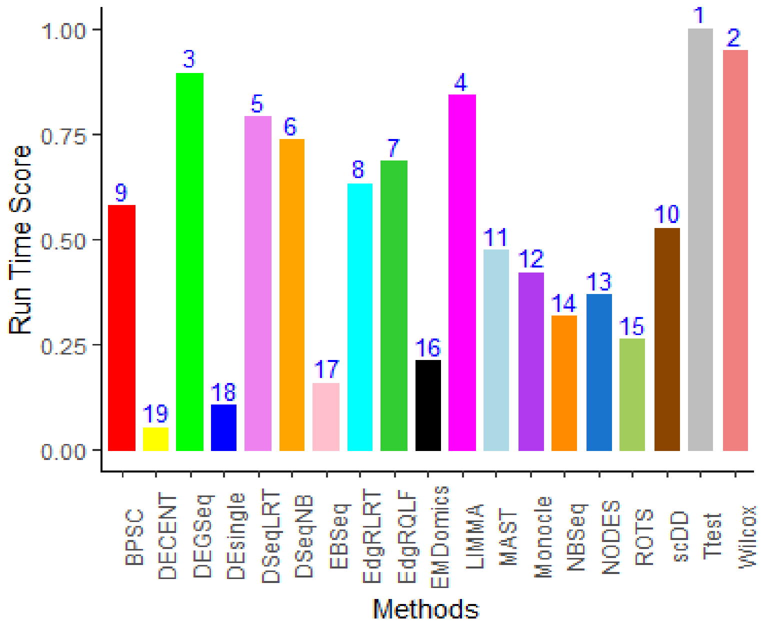

Runtime based performance evaluation of the scRNA-seq DE methods. X-axis represents the tested methods and Y-axis represents the runtime score.

<u>Document S12</u>. Similarity analysis of the scRNA-seq DE methods based on their ability to detect common DE genes

A. *Similarity analysis based on the detection of common genes*

Let, n_{rm} be the number of common DE genes detected by r^{th} and m^{th} DE methods; K: number of genes present in the DE gene sets detected though any DE method (*e.g.,* here 1000); p_{lm} be the proportions of the common genes detected by the rth and m^{th} DE methods. The values of n_{rm} are given in following Figures.

	DECENT	DEGSeq	DSeqNB	DSeqLRT	DEsingle	EBSeq	EdgRQLF	EdgRLRT	EMDomics	LIMMA	MAST	Monocle	NBSeq	NODES	ROTS	scDD	Ttest	Wilcox
BPSC	702	180	736	773	813	4	871	802	3	875	808	862	858	410	605	659	869	827
DECENT		184	807	621	631	49	682	755	3	736	587	605	663	367	463	391	670	604
DEGseq			197	160	177	277	180	186	6	180	178	176	181	431	257	153	177	174
DESeqNB				687	686	31	747	784	3	767	653	674	725	380	521	458	736	665
DESeqLRT					711	2	811	731	3	784	726	782	812	417	670	670	829	734
DEsingle						2	873	845	3	819	925	887	870	419	635	614	832	955
EBSeq							2	2	5	8	1	2	2	35	2	2	2	2
EdgRLRT								883	3	889	863	898	932	428	660	630	935	880
EdgRQLF									3	857	812	806	868	425	607	532	845	824
EMDomics										3	3	3	3	1	2	3	2	3
LIMMA											809	838	873	416	607	620	890	820
MAST												888	868	430	649	630	819	950
Monocle													917	421	659	701	870	908
NBSeq														433	666	641	903	882
NODES															637	360	426	419
ROTS																553	647	642
scDD																	649	639
T.test																		844

(A) Common genes detected between any pair of tested methods for Soumillon 2 data. The common genes between any pair of tested DE methods are shown in matrix form.

(B) Common genes detected between any pair of tested methods for real scRNA-seq datasets. The common genes between any pair of tested DE methods are shown in matrix form for (A) Islam data; (B) Tung data; (C) Soumillon 1 data; (D) Soumillon 3 data; (E) Savas data; (F) Grun; (G) Gienrahn data; (H) Ziegenhain data; (I) Chen data.

A / Methods	DECENT	DEGSeq	DESeqLRT	DESeqNB	DEsingle	EBSeq	edgRLRT	edgRQLF	LIMMA	MAST	Monocle	NBSeq	NODES	ROTS	scDD	Ttest	Wilcox
BPSC	191	184	173	39	872	2	727	664	2	166	289	208	593	782	31	719	706
DECENT		557	541	60	267	9	433	496	10	709	635	691	442	340	90	435	409
DEGSeq			576	140	213	6	298	323	11	561	554	572	383	288	181	302	275
DESeqLRT				96	195	2	284	290	1	699	727	749	358	331	113	283	250
DESeqNB					40	55	37	39	410	85	76	78	56	48	574	40	38
DEsingle						2	804	754	1	221	336	252	622	840	39	796	817
EBSeq							2	2	105	2	2	2	2	2	8	2	2
edgRLRT								910	5	368	468	387	707	785	47	968	900
edgRQLF									7	407	519	440	673	748	51	906	890
LIMMA										8	7	6	4	1	58	4	5
MAST											725	783	445	342	115	369	327
Monocle												871	514	450	103	471	425
NBSeq													468	381	107	391	343
NODES														643	76	699	665
ROTS															47	784	781
scDD																47	50
Ttest																	895

B	DECENT	DEGSeq	DSeqLRT	DSeqNB	DEsingle	EBSeq	EdgRQLF	EdgRLRT	EMDomics	LIMMA	MAST	Monocle	NBSeq	NODES	ROTS	scDD	Ttest	Wilcox
BPSC	426	158	625	466	471	38	546	155	532	621	514	567	693	569	633	583	587	38
DECENT		100	438	377	482	112	563	282	391	414	337	370	390	424	324	440	442	46
DEGSeq			135	357	92	142	112	91	104	133	203	130	135	129	146	122	123	391
DESeq2_LRT				396	549	25	586	138	555	601	485	570	580	589	540	595	628	30
DESeq_NB					372	91	427	196	376	470	294	320	388	361	299	382	436	409
Desingle						56	595	180	635	641	464	490	569	594	448	619	768	42
EBSeq							195	613	20	46	22	18	4	1	2	9	36	196
EdgeR								405	566	522	433	554	574	590	496	608	621	93
EdgeRQLF									130	168	90	95	96	89	64	104	150	221
LIMMA										649	438	517	664	569	493	594	739	39
MAST											398	448	576	486	437	520	790	40
Monocle												539	534	571	513	552	477	48
NBSeq													644	680	642	653	548	41
NODES														705	711	700	688	28
ROTS															673	938	625	29
scDD																650	539	26
T.test																	657	31
Wilcox																		38

C	DECENT	DEGSeq	DSeqLRT	DSeqNB	DEsingle	EBSeq	EdgRQLF	EdgRLRT	EMDomics	LIMMA	MAST	Monocle	NBSeq	NODES	ROTS	scDD	Ttest	Wilcox
BPSC	694	682	590	762	791	122	683	917	13	766	661	762	729	704	695	75	896	841
DECENT		579	362	767	689	358	750	756	21	640	714	660	721	541	489	89	735	701
DEGSEq			455	564	661	167	666	695	22	617	591	726	687	643	660	124	633	690
DSeqLRT				498	479	2	329	542	9	652	363	471	403	544	581	65	556	570
DSeqNB					693	244	625	782	14	727	647	619	654	557	520	85	813	739
DEsingle						155	730	843	15	723	697	776	762	668	673	88	749	868
EBSeq							262	164	30	181	214	168	219	70	27	82	181	151
EdgRQLF								744	31	576	745	771	842	602	541	95	658	710
EdgRLRT									15	767	712	784	780	698	680	87	892	874
EMDomics										13	25	21	28	13	9	97	13	16
LIMMA											603	670	634	649	663	77	791	805
MAST												700	732	605	534	90	657	713
Monocle													848	766	758	89	693	774
NBSeq														679	634	98	696	754
NODES															814	78	628	694
ROTS																83	615	683
scDD																	79	87
Ttest																		808

D	DECENT	DEGSeq	DSeqLRT	DSeqNB	DEsingle	EBSeq	EdgRLRT	EdgRQLF	EMDomics	LIMMA	MAST	Monocle	NBSeq	NODES	ROTS	scDD	Ttest	Wilcox
BPSC	500	94	908	765	762	2	799	710	10	2	167	448	436	388	16	40	776	718
DECENT		93	538	587	627	2	622	650	15	9	515	854	827	519	17	14	563	633
DEGSeq			100	104	107	253	109	113	59	2	90	106	101	82	253	244	108	110
DSeqLRT				815	783	2	845	746	10	6	181	482	471	379	20	39	800	748
DSeqNB					862	1	895	853	13	5	197	478	478	322	22	29	834	841
DEsingle						2	918	911	14	2	213	515	509	312	17	24	883	947
EBSeq							1	2	79	1	9	1	2	2	897	143	2	2
EdgRLRT								901	14	4	203	519	512	322	20	28	889	895
EdgRQLF									14	3	211	522	518	273	19	27	866	943
EMDomics										541	22	14	14	7	71	2	12	14
LIMMA											9	9	9	9	2	2	3	3
MAST												578	558	492	21	52	182	216
Monocle													892	616	17	24	474	516
NBSeq														593	18	23	469	514
NODES															11	47	277	275
ROTS																227	18	19
scDD																	33	26
Ttest																		881

E	DECENT	DEGSeq	DSeqLRT	DSeqNB	DEsingle	EBSeq	EdgRQLF	EdgRLRT	EMDomics	LIMMA	MAST	Monocle	NBSeq	NODES	ROTS	scDD	Ttest	Wilcox
BPSC	655	231	732	734	630	177	797	762	2	578	792	765	801	653	173	595	691	703
DECENT		243	544	737	471	278	621	710	1	534	579	579	653	508	185	359	475	559
DGSeq			235	237	243	566	243	249	14	230	240	247	248	339	727	217	234	243
DSEqLRT				702	523	130	625	599	2	430	645	724	711	628	171	695	525	544
DSeqNB					518	202	659	672	2	527	649	673	726	605	177	510	512	579
DEsingle						106	802	725	2	730	804	748	714	576	184	509	800	861
EBSeq							159	203	9	166	141	128	161	199	447	70	130	136
edgRLRT								873	2	724	919	834	840	631	185	533	831	875
edgRQLF									1	665	822	773	835	611	186	460	749	802
EMDomics										2	2	2	2	2	12	2	1	2
LIMMA											722	676	661	512	173	447	695	742
MAST												833	823	647	181	551	818	894
Monocle													882	643	186	652	727	760
NBSeq														646	186	588	698	754
NODES															257	508	561	624
ROTS																163	172	183
scDD																	512	484
Ttest																		838

F

	DECENT	DEGSEq	DESEqNB	DSeqLRT	DEsingle	EBSeq	edgRLRT	edgRQLF	LIMMA	MAST	Monocle	NBSeq	NODES	ROTS	scDD	Ttest	Wilcox
BPSC	372	349	423	790	889	11	843	727	2	382	457	343	515	880	178	817	686
DECENT		381	290	340	434	317	429	458	11	388	739	743	559	406	114	431	448
DEGSEq			291	355	380	123	325	335	24	392	380	394	423	410	126	316	295
DESEqNB				435	426	77	410	419	2	310	323	273	387	453	204	407	379
DSeqLRT					738	2	693	613	2	410	389	302	521	821	211	678	576
DEsingle						34	859	776	2	401	539	417	564	871	161	851	759
EBSeq							92	152	21	102	292	332	100	2	48	98	141
edgRLRT								871	2	367	539	412	517	774	162	931	828
edgRQLF									1	369	600	477	561	709	145	853	826
LIMMA										2	7	10	1	2	2	2	2
MAST											397	377	445	402	202	365	359
Monocle												805	613	498	106	559	573
NBSeq													557	395	93	428	446
NODES														580	164	525	509
ROTS															173	770	656
scDD																163	160
Ttest																	844

G	DECENT	DEGSeq	DSeqLRT	DSeqNB	DEsingle	EBSeq	EdgRQLF	EdgRLRT	EMDomics	LIMMA	MAST	Monocle	NBSeq	NODES	ROTS	scDD	Ttest	Wilcox
BPSC	780	627	710	777	693	734	770	791	1	736	726	610	317	553	589	483	594	660
DECENT		658	718	853	814	754	917	928	2	708	852	725	371	591	625	539	724	843
DEGSeq			636	669	623	643	624	676	2	605	607	634	336	593	795	566	599	636
DSeqLRT				756	703	886	665	758	2	821	661	738	435	772	550	708	651	640
DSeqNB					791	775	793	847	2	740	774	732	393	642	598	610	673	743
DEsingle						698	774	823	2	676	775	772	401	594	565	582	690	768
EBSeq							728	805	2	830	700	725	405	725	572	676	690	697
EdgRQLF								888	3	669	859	663	320	521	613	477	687	817
EdgRLRT									2	744	839	721	365	625	644	575	740	843
EMDomics										2	3	2	2	2	2	1	1	2
LIMMA											665	740	444	743	566	680	670	661
MAST												698	336	561	608	514	748	843
Monocle													476	669	546	653	712	709
NBSeq														450	296	440	383	354
NODES															559	820	689	604
ROTS																508	649	642
scDD																	632	548
Ttest																		800

H	DECENT	DEGSeq	DSeqLR	DSeqNB	DESingle	EBSeq	EdgRLRT	EdgRQLF	EMDomics	LIMMA	MAST	Monocle	NBSEq	NODES	ROTS	scDD	T.test	Wilcox
BPSC	31	217	238	51	247	2	900	877	29	2	202	123	39	333	480	55	884	825
DECENT		593	229	64	33	596	13	17	66	109	76	481	83	138	15	83	10	17
DEGSeq			306	62	176	422	208	223	53	31	107	575	77	230	141	88	210	218
DSeqLR				34	63	39	191	181	29	2	276	206	47	295	242	127	190	178
DSeqNB					58	63	57	55	967	93	63	65	924	59	69	305	56	55
DESingle						14	273	293	46	2	160	115	53	124	237	121	278	326
EBSeq							2	3	66	97	11	458	75	31	2	48	2	2
EdgRLRT								966	43	1	219	141	42	348	496	59	967	894
EdgRQLF									41	1	209	150	41	349	500	59	957	911
EMDomics										95	67	68	912	52	66	298	42	43
LIMMA											1	70	61	1	1	2	1	1
MAST												98	62	353	316	148	202	204
Monocle													76	184	97	71	142	148
NBSEq														56	65	327	41	40
NODES															248	106	349	354
ROTS																180	492	497
scDD																	60	59
T.test																		894

I	DECENT	DEGSeq	DSeqLRT	DSeqNB	DEsingle	EBSeq	EdgRLRT	EdgRQLF	EMDomics	LIMMA	MAST	Monocle	NBSeq	NODES	ROTS	scDD	Ttest	Wilcox
BPSC	63	278	311	234	438	14	154	92	852	30	95	368	234	408	265	381	476	478
DECENT		33	685	784	513	456	842	875	75	250	692	469	664	138	28	205	438	464
DEGSeq			112	71	151	40	57	34	277	7	50	218	112	368	748	305	194	163
DSeqLRT				772	764	361	829	685	297	180	724	607	729	314	98	382	667	705
DSeqNB					654	388	825	778	216	225	665	530	711	230	62	292	575	628
DEsingle						253	621	516	417	145	588	642	700	400	130	458	769	828
EBSeq							442	446	22	231	424	308	313	81	35	140	214	227
EdgRLRT								836	154	227	758	529	703	206	49	275	537	563
EdgRQLF									100	290	662	455	630	151	28	211	448	487
EMDomics										42	97	392	240	403	270	387	433	441
LIMMA											196	135	149	33	5	52	149	159
MAST												509	664	194	43	264	494	510
Monocle													667	510	195	603	605	637
NBSeq														309	101	388	601	653
NODES															299	707	452	434
ROTS																244	164	142
scDD																	502	476
Ttest																		782

J	DECENT	DEGSeq	DeSeqLRT	DeSeqNB	DEsingle	EBSeq	edgRLRT	edgRQLF	LIMMMA	MAST	Monocle	NBSeq	NODES	ROTS	scDD	Ttest	Wilcox
BPSC	191	184	173	39	872	0	727	664	0	166	289	208	593	782	31	719	706
DECENT		557	541	60	267	9	433	496	10	709	635	691	442	340	90	435	409
DEGSeq			576	140	213	6	298	323	11	561	554	572	383	288	181	302	275
DeSeqLRT				96	195	0	284	290	1	699	727	749	358	331	113	283	250
DeSeqNB					40	55	37	39	410	85	76	78	56	48	574	40	38
DEsingle						0	804	754	1	221	336	252	622	840	39	796	817
EBSeq							0	2	105	0	2	2	0	0	8	0	0
edgRLRT								910	5	368	468	387	707	785	47	968	900
edgRQLF									7	407	519	440	673	748	51	906	890
LIMMMA										8	7	6	4	1	58	4	5
MAST											725	783	445	342	115	369	327
Monocle												871	514	450	103	471	425
NBSeq													468	381	107	391	343
NODES														643	76	699	665
ROTS															47	784	781
scDD																47	50
Ttest																	895

The proportions of the common genes detected by any two scRNA-seq DE methods can be expressed in Eq. 36.

$$p_{rm} = \frac{n_{rm}}{K} \quad (36)$$

H_0: $n_{rm} = 0$ (no common genes detected between the rth and m^{th} DE methods)

H_1: $n_{rm} > 0$ (common genes detected between the rth and m^{th} DE methods)

To test H_0 vs. H_1 the Binomial test can be used. Here, $n_{rm} \sim Bion(K, P_{rm})$ and the PMF of n_{rm} can be expressed in Eq. 37.

$$P[n_{rm} = S] = \binom{K}{S} P_{rm}{}^{S} (1 - P_{rm})^{K-S} \quad (37)$$

where, P_{rm} is the parameter usually unknown and can be estimated through Eq. 41. Under the large sample condition, the test statistic in Eq. 37 follows standard normal distribution asymptotically. The *p-values* for the above statistical tests (repeated for each pair of methods on all the datasets) can be computed through *Binom.test* function implemented in stats R package.

B. Combined data analysis for detecting common genes

Let, C_d be the total number of cells present in the dth (d = 1, 2, …, D) scRNA-seq data. Now, the weight depicting the ability of the rth and m^{th} DE methods for detecting common genes over all the considered datasets can be expressed as in Eq. 38.

$$w_{rm} = \frac{\sum_{d=1}^{D} C_d P_{rmd}}{\sum_{d=1}^{D} C_d} \quad (38)$$

where, p_{rmd} be the proportions of the common genes detected by the rth and m^{th} DE methods in d^{th} scRNA-seq data.

Supplementary Figures (for Results and Discussion)

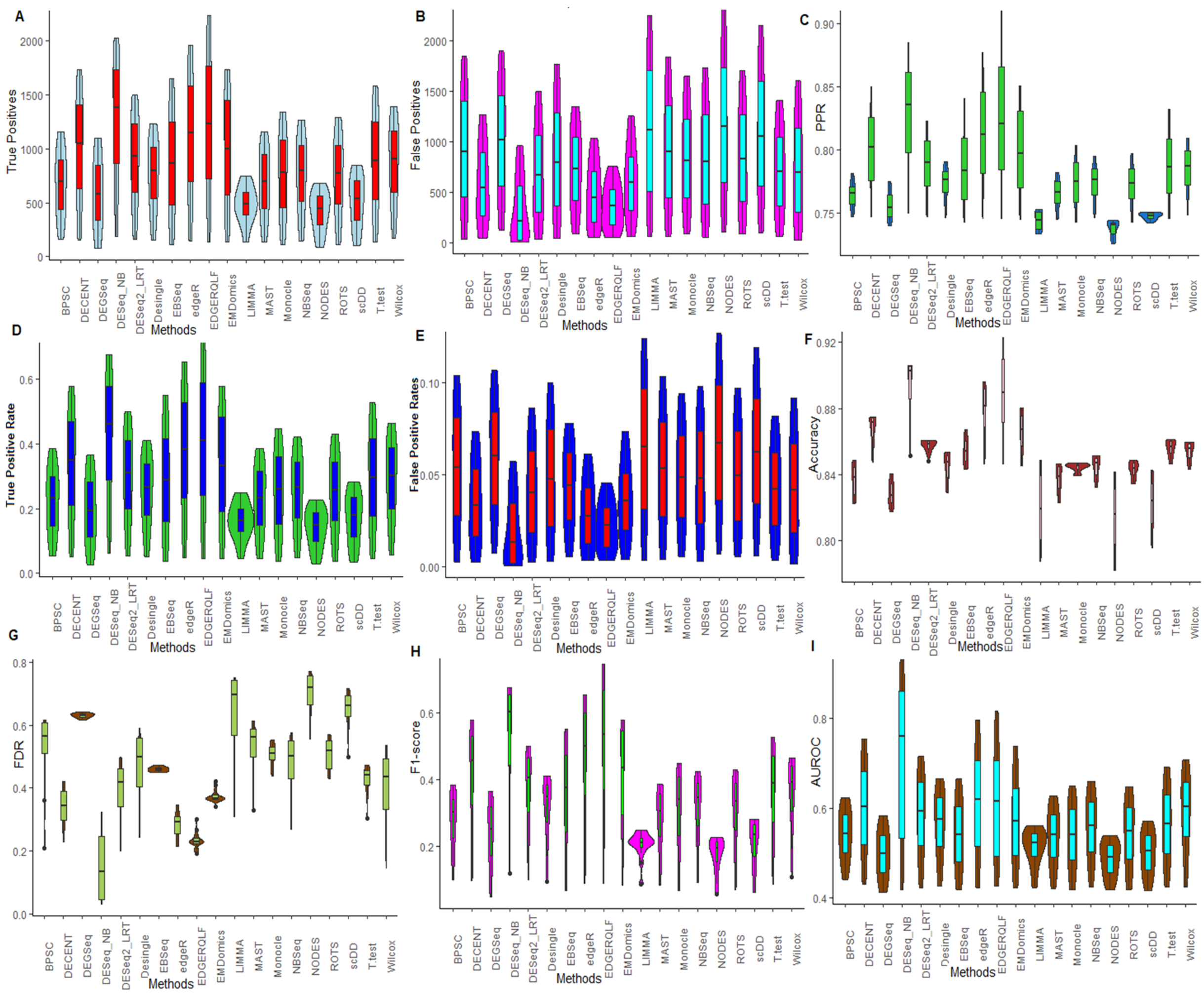

Figure S3. Comparative performance evaluation of the Differential Expression (DE) methods through the performance metrics for Islam data. The tested DE methods are evaluated on the Islam scRNA-seq data through the performance evaluation metrics, such as TP, FP, TPR, FPR, PPR, FDR, Accuracy, F1 score, and AUROC. The 19 tested methods are shown in the X-axis. The Violin plots are shown for comparative evaluation of tested methods through (A) TP; (B) FP; (C) PPR; (D) TPR; (E) FPR; (F) Accuracy; (G) FDR; (H) F1 score; and (I) AUROC. The violin plot shows the full distribution of the performance metrics computed through each tested method. The box represents inter-quartile range, the horizontal line represents median, the bars on the boxes shows the 1.5 x inter-quartile range.

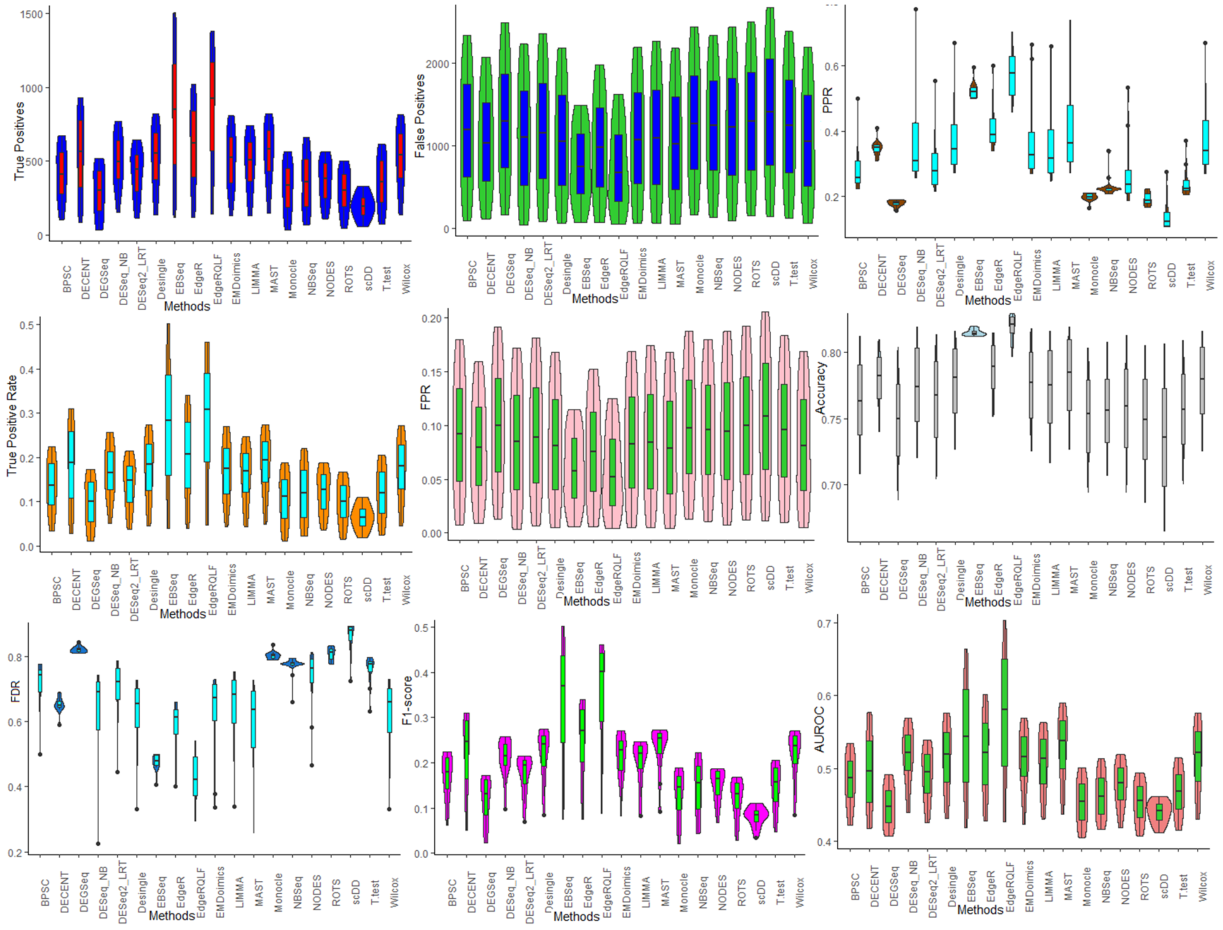

Figure S4. Comparative performance evaluation of the Differential Expression (DE) methods through the performance metrics for Tung data. The tested DE methods are evaluated on the Tung scRNA-seq data through the performance evaluation metrics, such as TP, FP, TPR, FPR, PPR, FDR, Accuracy, F1 score, and AUROC. The 19 tested methods are shown in the X-axis. The Violin plots are shown for comparative evaluation of tested methods through (A) TP; (B) FP; (C) PPR; (D) TPR; (E) FPR; (F) Accuracy; (G) FDR; (H) F1 score; and (I) AUROC. The violin plot shows the full distribution of the performance metrics computed through each tested method. The box represents inter-quartile range, the horizontal line represents median, the bars on the boxes shows the 1.5 x inter-quartile range.

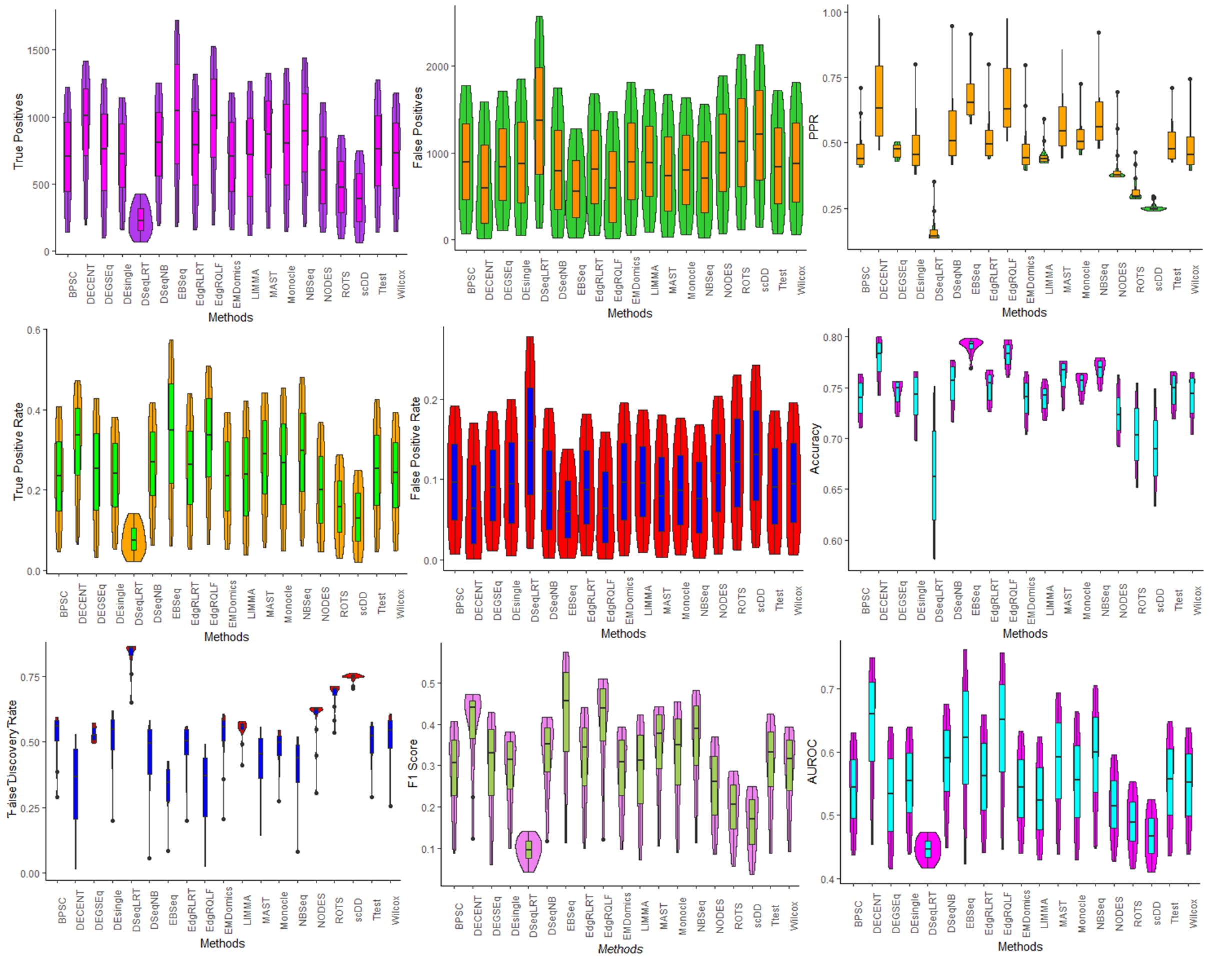

Figure S5. Comparative performance evaluation of the Differential Expression (DE) methods through the performance metrics for Soumillon1 data. The tested DE methods are evaluated on the Islam scRNA-seq data through the performance evaluation metrics, such as TP, FP, TPR, FPR, PPR, FDR, Accuracy, F1 score, and AUROC. The 19 tested methods are shown in the X-axis. The Violin plots are shown for comparative evaluation of tested methods through (A) TP; (B) FP; (C) PPR; (D) TPR; (E) FPR; (F) Accuracy; (G) FDR; (H) F1 score; and (I) AUROC. The violin plot shows the full distribution of the performance metrics computed through each tested method. The box represents inter-quartile range, the horizontal line represents median, the bars on the boxes shows the 1.5 x inter-quartile range.

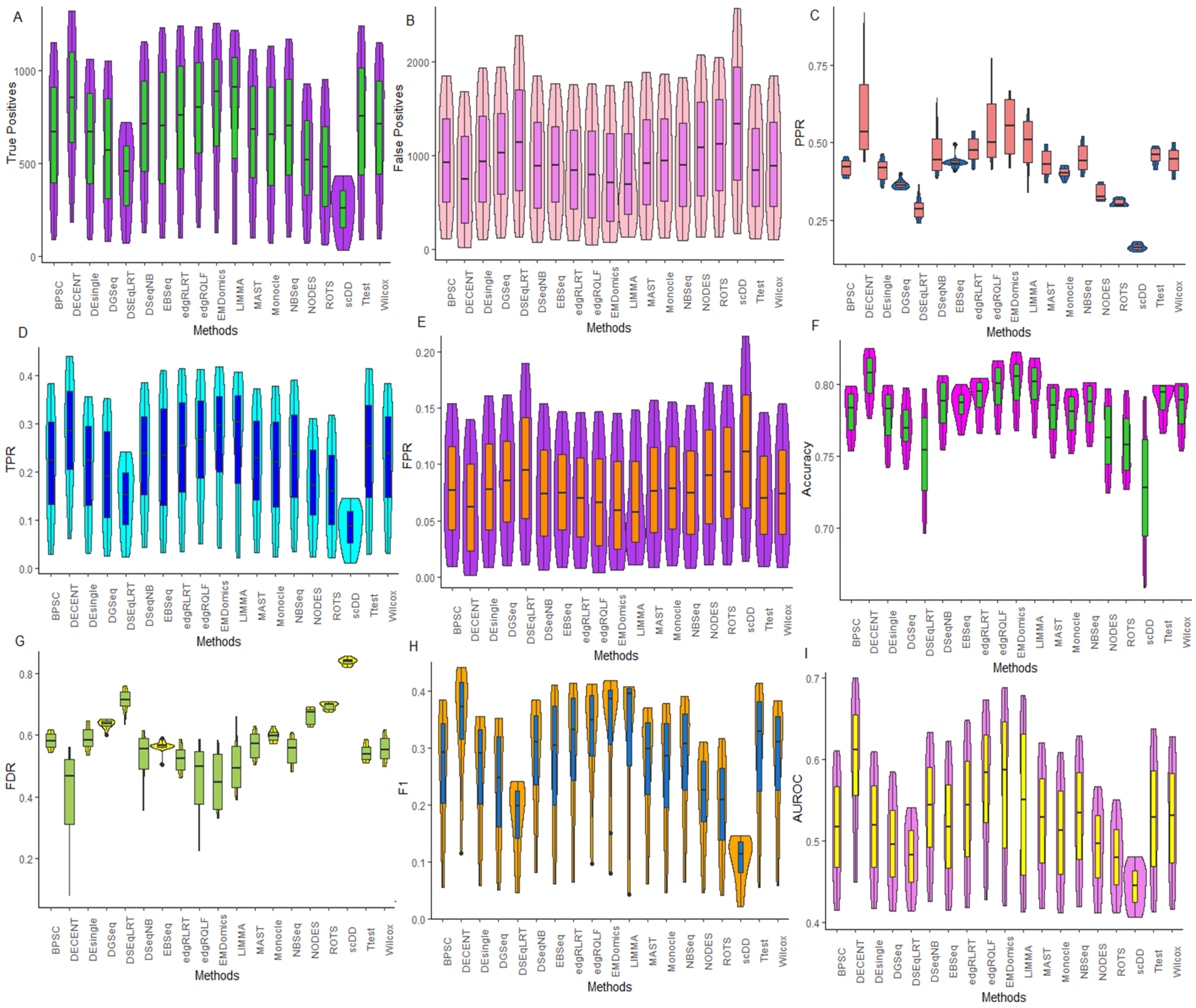

Figure S6. Comparative performance evaluation of the Differential Expression (DE) methods through the performance metrics for Soumillon 3 data. The tested DE methods are evaluated on the Islam scRNA-seq data through the performance evaluation metrics, such as TP, FP, TPR, FPR, PPR, FDR, Accuracy, F1 score, and AUROC. The 19 tested methods are shown in the X-axis. The Violin plots are shown for comparative evaluation of tested methods through (A) TP; (B) FP; (C) PPR; (D) TPR; (E) FPR; (F) Accuracy; (G) FDR; (H) F1 score; and (I) AUROC. The violin plot shows the full distribution of the performance metrics computed through each tested method. The box represents inter-quartile range, the horizontal line represents median, the bars on the boxes shows the 1.5 x inter-quartile range.

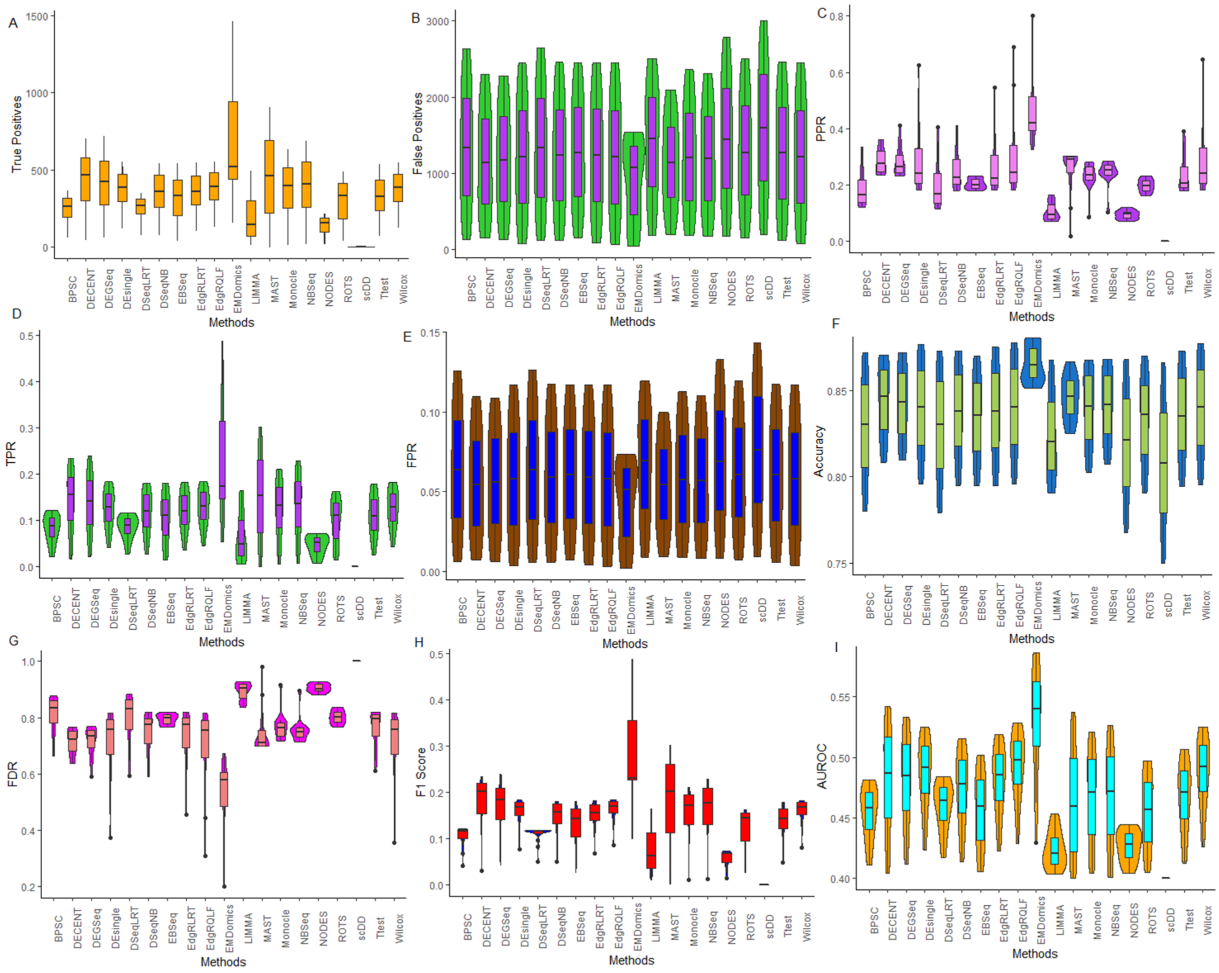

Figure S7. Comparative performance evaluation of the Differential Expression (DE) methods through the performance metrics for Klein data. The tested DE methods are evaluated on the Klein scRNA-seq data through the performance evaluation metrics, such as TP, FP, TPR, FPR, PPR, FDR, Accuracy, F1 score, and AUROC. The 19 tested methods are shown in the X-axis. The Violin plots are shown for comparative evaluation of tested methods through (A) TP; (B) FP; (C) PPR; (D) TPR; (E) FPR; (F) Accuracy; (G) FDR; (H) F1 score; and (I) AUROC. The violin plot shows the full distribution of the performance metrics computed through each tested method. The box represents inter-quartile range, the horizontal line represents median, the bars on the boxes shows the 1.5 x inter-quartile range.

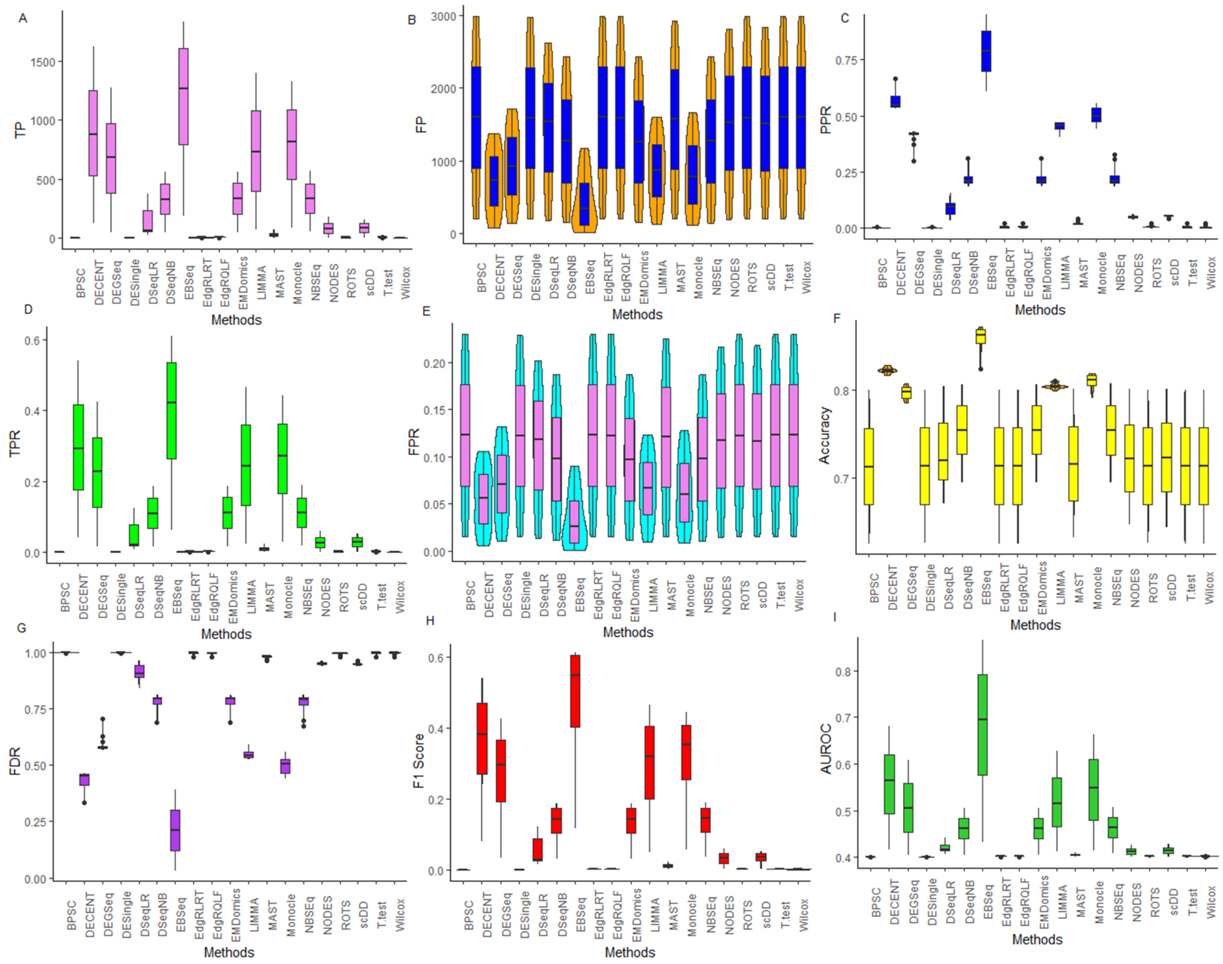

Figure S8. Comparative performance evaluation of the Differential Expression (DE) methods through the performance metrics for Gierahn data. The tested DE methods are evaluated on the Gierahn scRNA-seq data through the performance evaluation metrics, such as TP, FP, TPR, FPR, PPR, FDR, Accuracy, F1 score, and AUROC. The 19 tested methods are shown in the X-axis. The Violin plots are shown for comparative evaluation of tested methods through (A) TP; (B) FP; (C) PPR; (D) TPR; (E) FPR; (F) Accuracy; (G) FDR; (H) F1 score; and (I) AUROC. The violin plot shows the full distribution of the performance metrics computed through each tested method. The box represents inter-quartile range, the horizontal line represents median, the bars on the boxes shows the 1.5 x inter-quartile range.

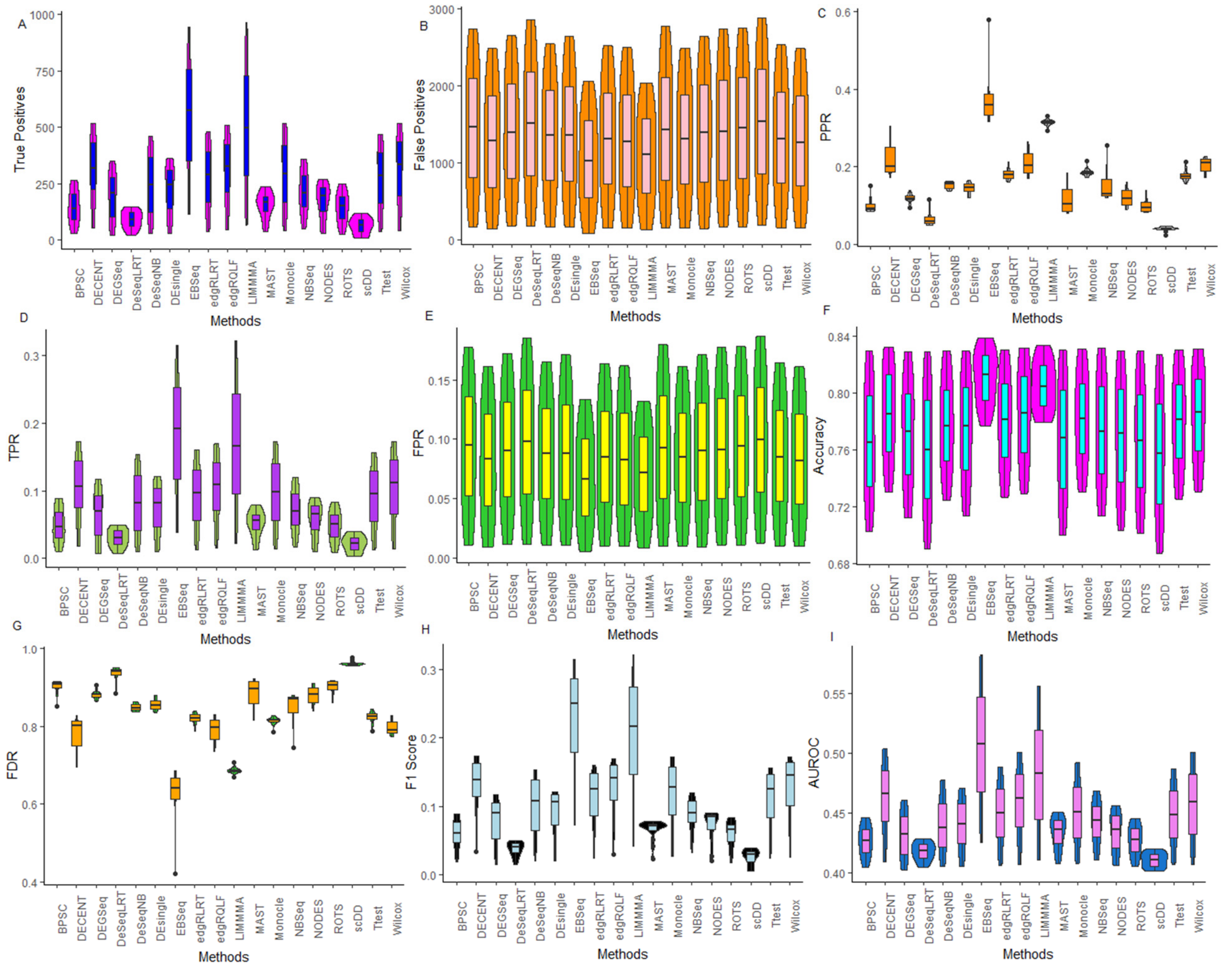

Figure S9. Comparative performance evaluation of the Differential Expression (DE) methods through the performance metrics for Chen data. The tested DE methods are evaluated on the Chen scRNA-seq data through the performance evaluation metrics, such as TP, FP, TPR, FPR, PPR, FDR, Accuracy, F1 score, and AUROC. The 19 tested methods are shown in the X-axis. The Violin plots are shown for comparative evaluation of tested methods through (A) TP; (B) FP; (C) PPR; (D) TPR; (E) FPR; (F) Accuracy; (G) FDR; (H) F1 score; and (I) AUROC. The violin plot shows the full distribution of the performance metrics computed through each tested method. The box represents inter-quartile range, the horizontal line represents median, the bars on the boxes shows the 1.5 x inter-quartile range.

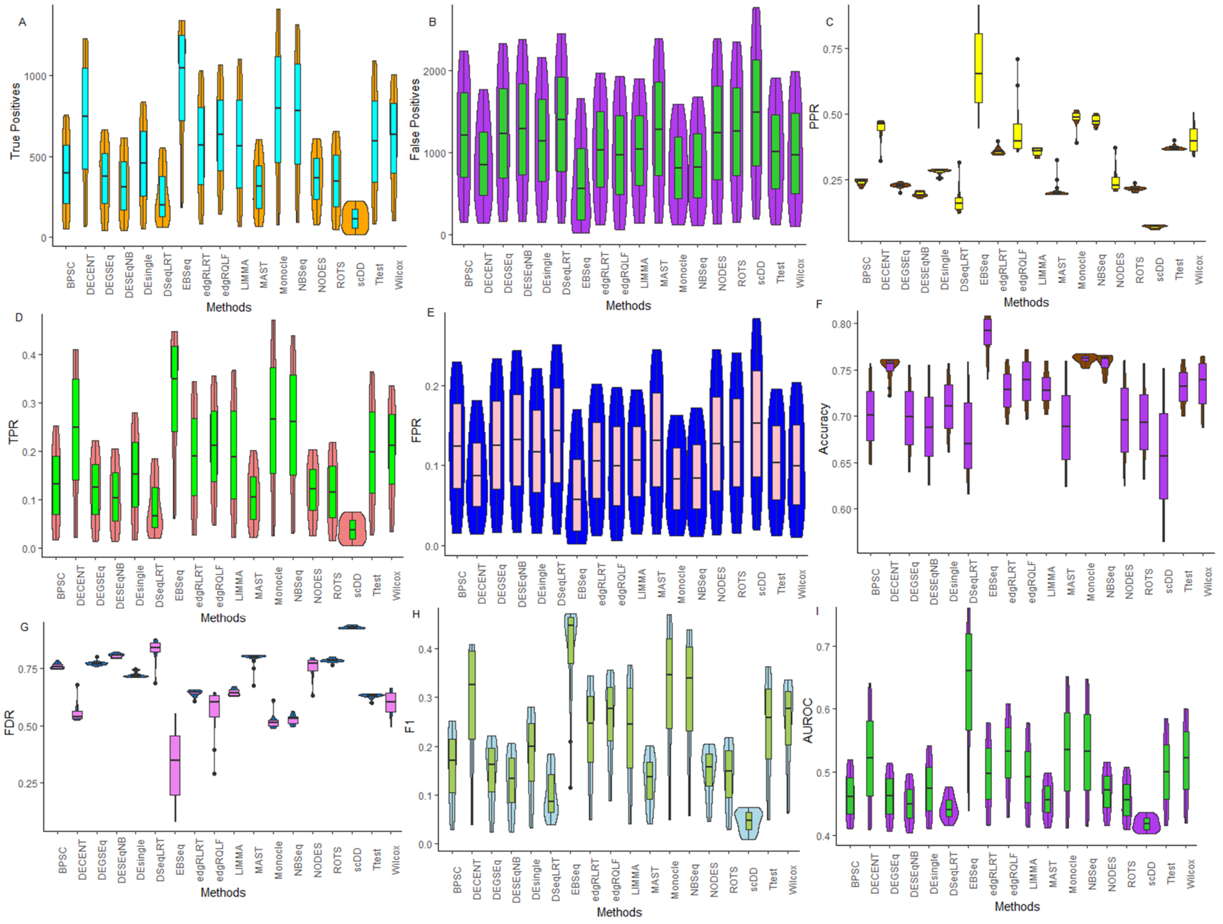

Figure S10. Comparative performance evaluation of the Differential Expression (DE) methods through the performance metrics for Savas data. The tested DE methods are evaluated on the Savas scRNA-seq data through the performance evaluation metrics, such as TP, FP, TPR, FPR, PPR, FDR, Accuracy, F1 score, and AUROC. The 19 tested methods are shown in the X-axis. The Violin plots are shown for comparative evaluation of tested methods through (A) TP; (B) FP; (C) PPR; (D) TPR; (E) FPR; (F) Accuracy; (G) FDR; (H) F1 score; and (I) AUROC. The violin plot shows the full distribution of the performance metrics computed through each tested method. The box represents inter-quartile range, the horizontal line represents median, the bars on the boxes shows the 1.5 x inter-quartile range.

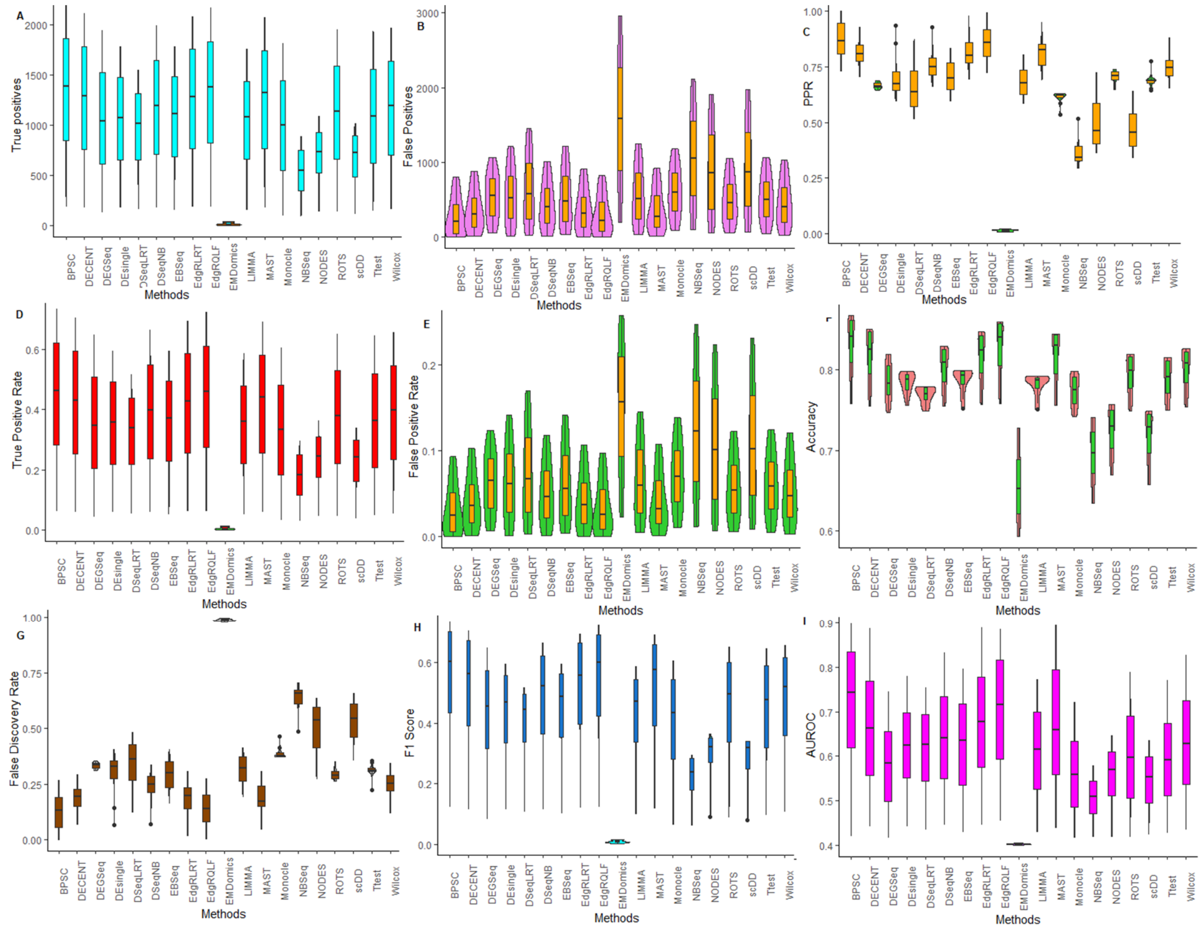

Figure S11. Comparative performance evaluation of the Differential Expression (DE) methods through the performance metrics for Grun data. The tested DE methods are evaluated on the Grun scRNA-seq data through the performance evaluation metrics, such as TP, FP, TPR, FPR, PPR, FDR, Accuracy, F1 score, and AUROC. The 19 tested methods are shown in the X-axis. The Violin plots are shown for comparative evaluation of tested methods through (A) TP; (B) FP; (C) PPR; (D) TPR; (E) FPR; (F) Accuracy; (G) FDR; (H) F1 score; and (I) AUROC. The violin plot shows the full distribution of the performance metrics computed through each tested method. The box represents inter-quartile range, the horizontal line represents median, the bars on the boxes shows the 1.5 x inter-quartile range.

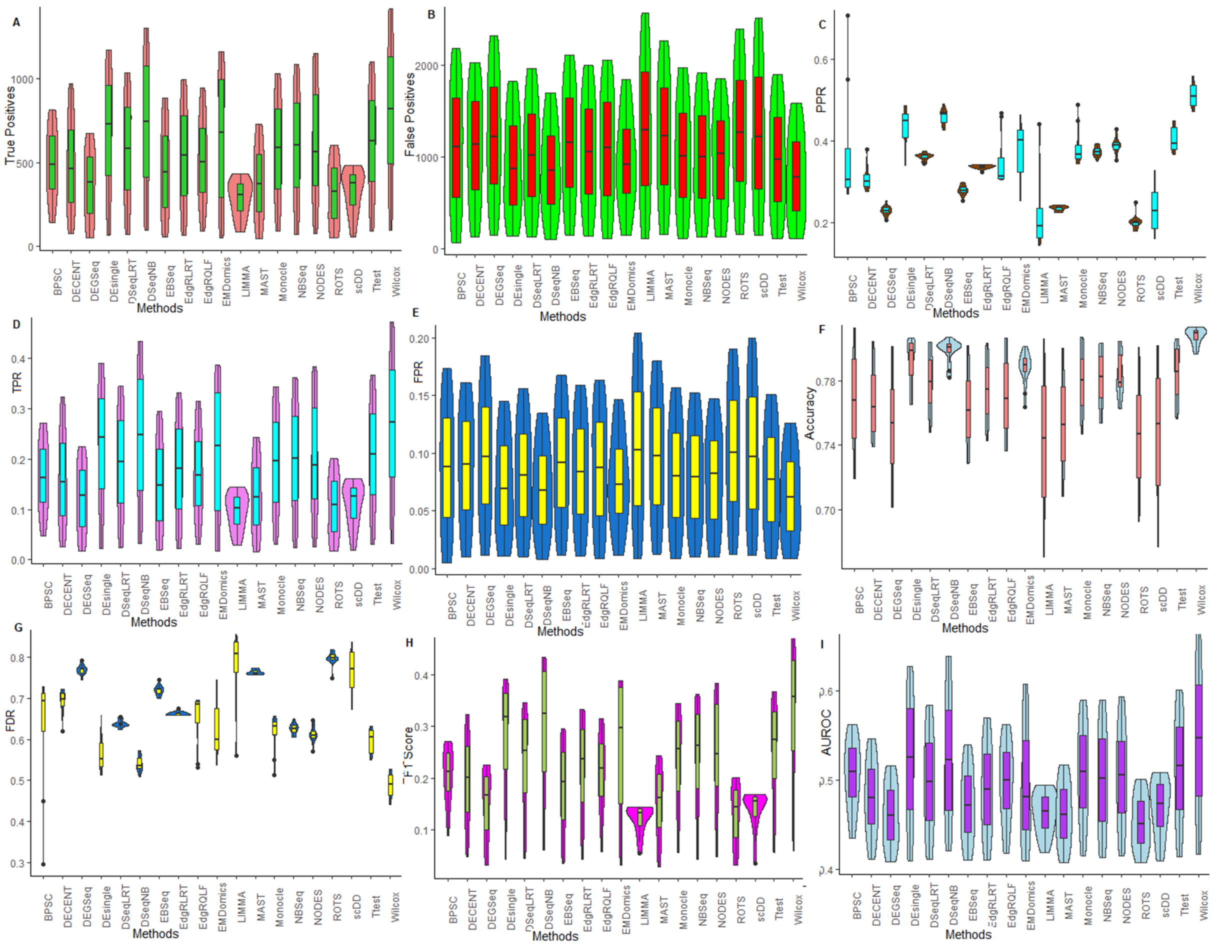

Figure S12. Comparative performance evaluation of the Differential Expression (DE) methods through the performance metrics for Ziegenhain data. The tested DE methods are evaluated on the Ziegenhain scRNA-seq data through the performance evaluation metrics, such as TP, FP, TPR, FPR, PPR, FDR, Accuracy, F1 score, and AUROC. The 19 tested methods are shown in the X-axis. The Violin plots are shown for comparative evaluation of tested methods through (A) TP; (B) FP; (C) PPR; (D) TPR; (E) FPR; (F) Accuracy; (G) FDR; (H) F1 score; and (I) AUROC. The violin plot shows the full distribution of the performance metrics computed through each tested method. The box represents inter quartile range, the horizontal line represents median, the bars on the boxes shows the 1.5x inter-quartile range.

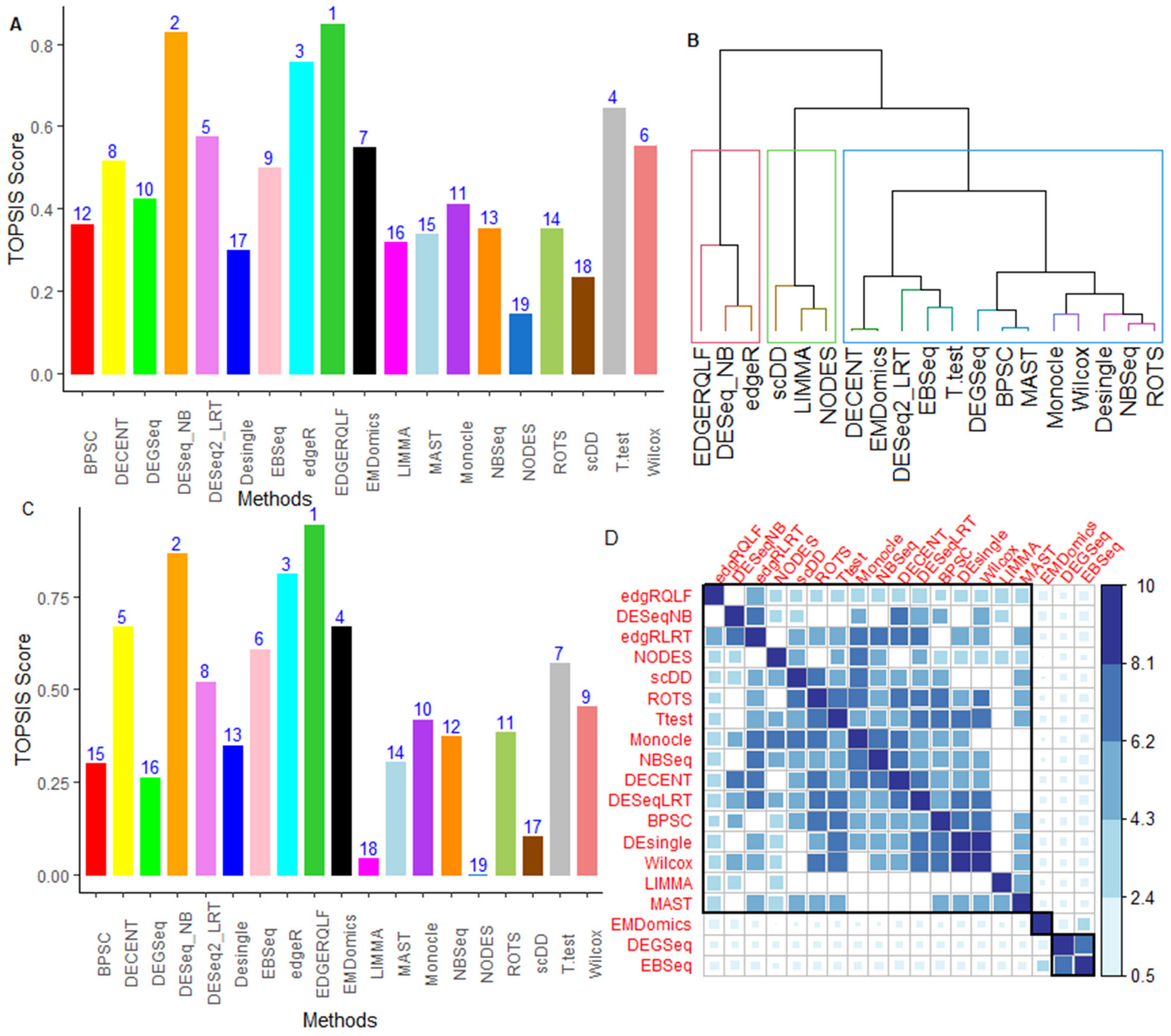

Figure S13. Performance evaluation of DE methods under Multiple Criteria Decision Making (MCDM) setup for Islam data. The comparative performance analysis of the 19 tested methods was carried out through TOPSIS approach under MCDM setup on Islam dataset. The MCDM-TOPSIS analysis was carried out under two settings, *i.e.*, (i) multi-criteria including runtime criterion; (ii) multi-criteria excluding runtime criterion. X-axis represents the tested methods and Y-axis represents the TOPSIS scores. The results from the (A) MCDM-TOPSIS analysis of the DE methods are shown for 12 performance metrics including runtime criterion; (B) Average similarities between the evaluated DE methods based on the 13-performance metrics. The dendrogram was obtained by average-linkage hierarchical clustering based on the matrix of average values of performance metrics over all gene sets; (C) MCDM-TOPSIS analysis of the DE methods based on 12 performance metrics excluding runtime criterion; (D) Similarity analysis of the tested methods based on their ability to detect common genes. The statistical

significance values are computed through Binomial test (Supp. Document S12). The strengths of similarity scores are represented in various colors and white empty boxes represents non-significant values.

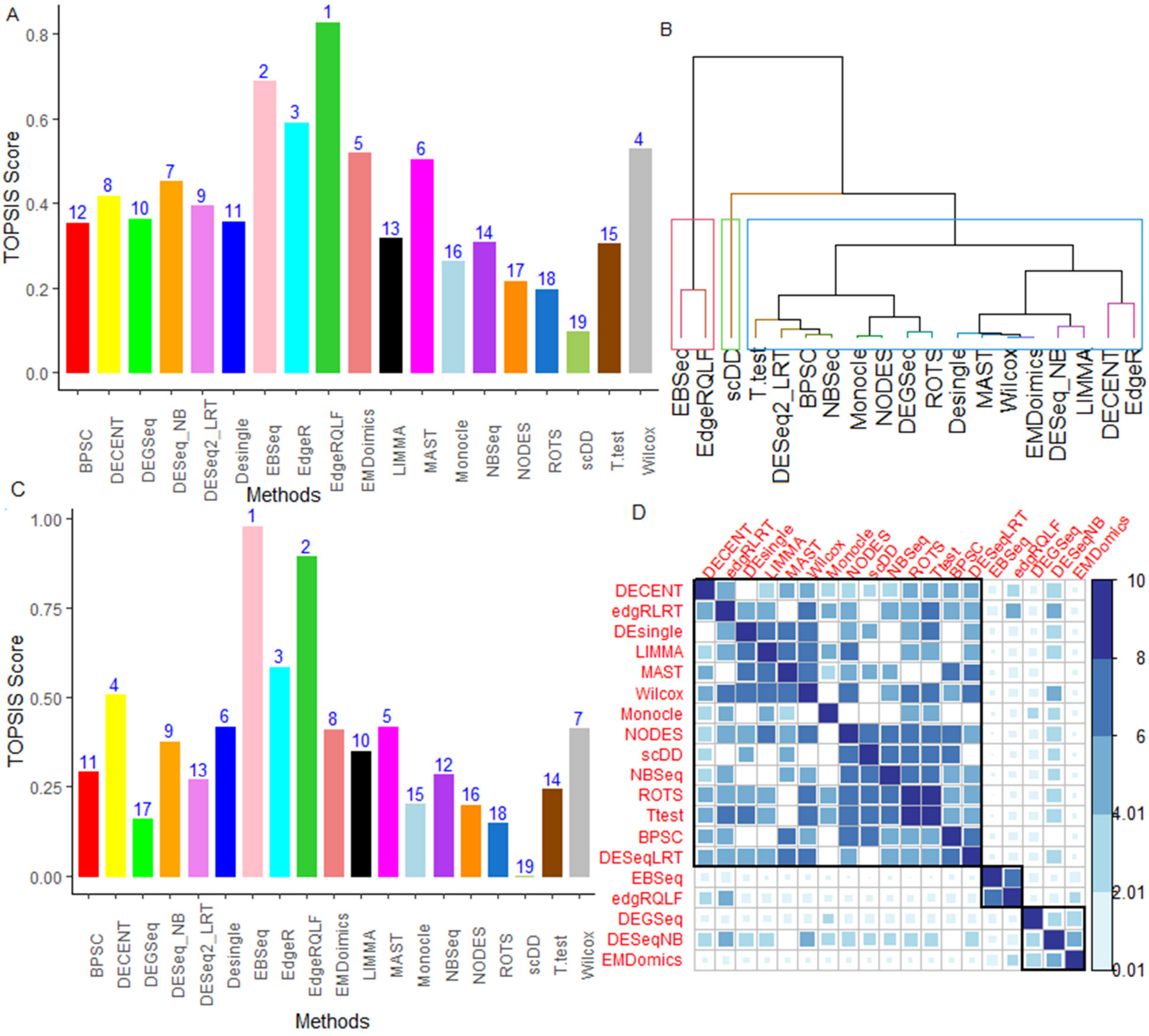

Figure S14. Performance evaluation of DE methods under the MCDM setup for Tung data. The comparative performance analysis of the 19 tested methods was carried out through TOPSIS approach under MCDM setup on Tung dataset. The MCDM-TOPSIS analysis was carried out under two settings, i.e. (i) multi-criteria including runtime criterion (total 13 criteria); (ii) multi-criteria excluding runtime criterion (12 criteria). X-axis represents the tested methods and Y-axis represents the TOPSIS scores. The results from the (A) MCDM-TOPSIS analysis of the DE methods are shown for 13 performance metrics including runtime criterion; (B) Average similarities between the evaluated DE methods based on the 13-performance metrics. The dendrogram was obtained by average-linkage hierarchical clustering based on the matrix of average values of performance metrics over all gene sets; (C) MCDM-TOPSIS analysis of the DE methods

based on 12 performance metrics excluding runtime criterion; (D) Similarity analysis of the tested methods based on their ability to detect common genes. The statistical significance values are computed through Binomial test (Supp. Document S12). The strengths of similarity scores are represented in various colors and white empty boxes represents non-significant values.

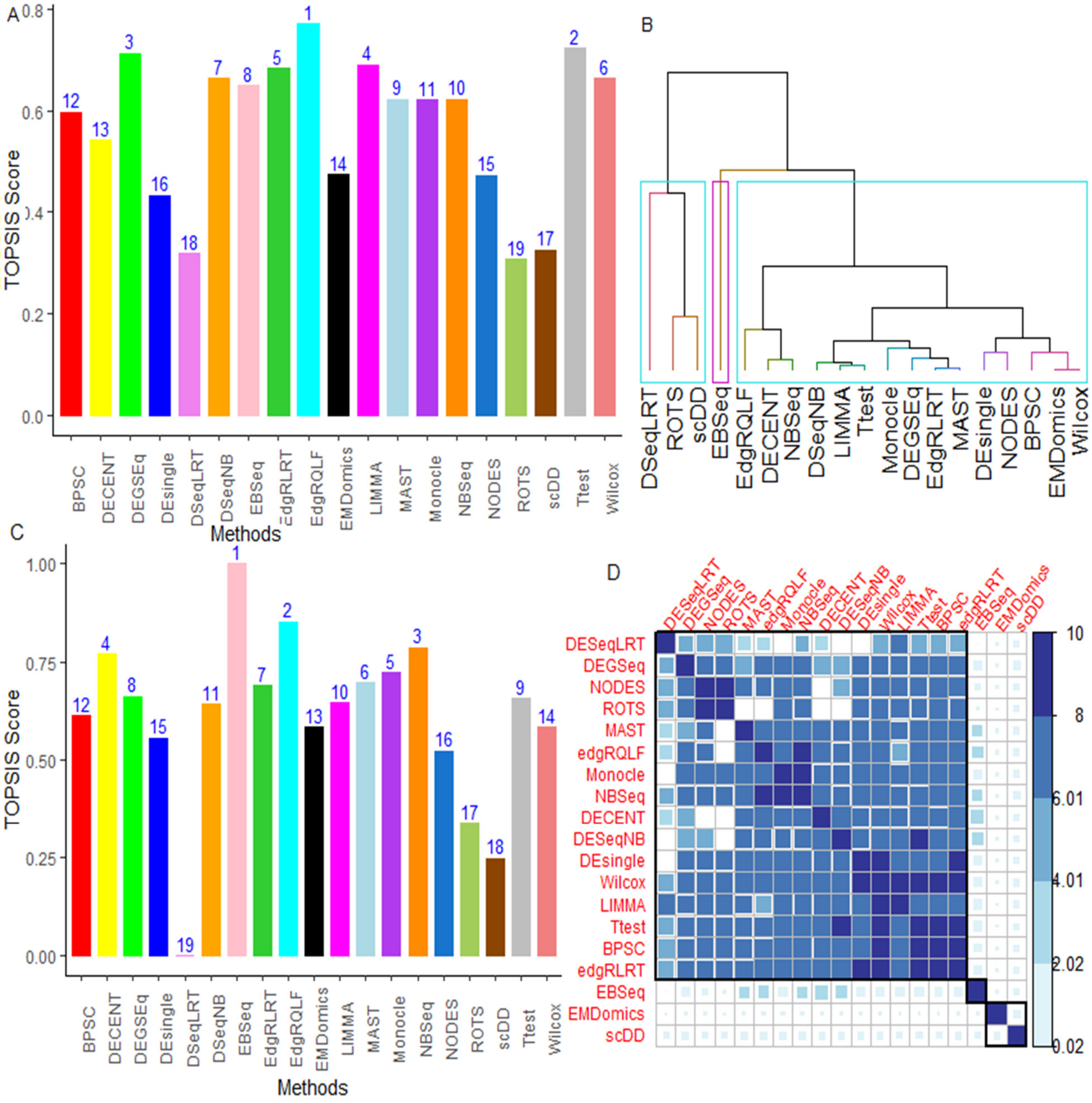

Figure S15. Performance evaluation of DE methods under the MCDM setup for Soumillion1 data. The comparative performance analysis of the 19 tested methods was carried out through TOPSIS approach under MCDM setup on Soumillion1 dataset. The MCDM-TOPSIS analysis was carried out under two settings, i.e. (i)

multi-criteria including runtime criterion; (ii) multi-criteria excluding runtime criterion. X-axis represents the tested methods and Y-axis represents the TOPSIS scores. The results from the (A) MCDM-TOPSIS analysis of the DE methods are shown for 12 performance metrics including runtime criterion; (B) Average similarities between the evaluated DE methods based on the 13-performance metrics. The dendrogram was obtained by average-linkage hierarchical clustering based on the matrix of average values of performance metrics over all gene sets; (C) MCDM-TOPSIS analysis of the DE methods based on 12 performance metrics excluding runtime criterion; (D) Similarity analysis of the tested methods based on their ability to detect common genes. The statistical significance values are computed through Binomial test (Supp. Document S12). The strengths of similarity scores are represented in various colors and white empty boxes represents non-significant values.

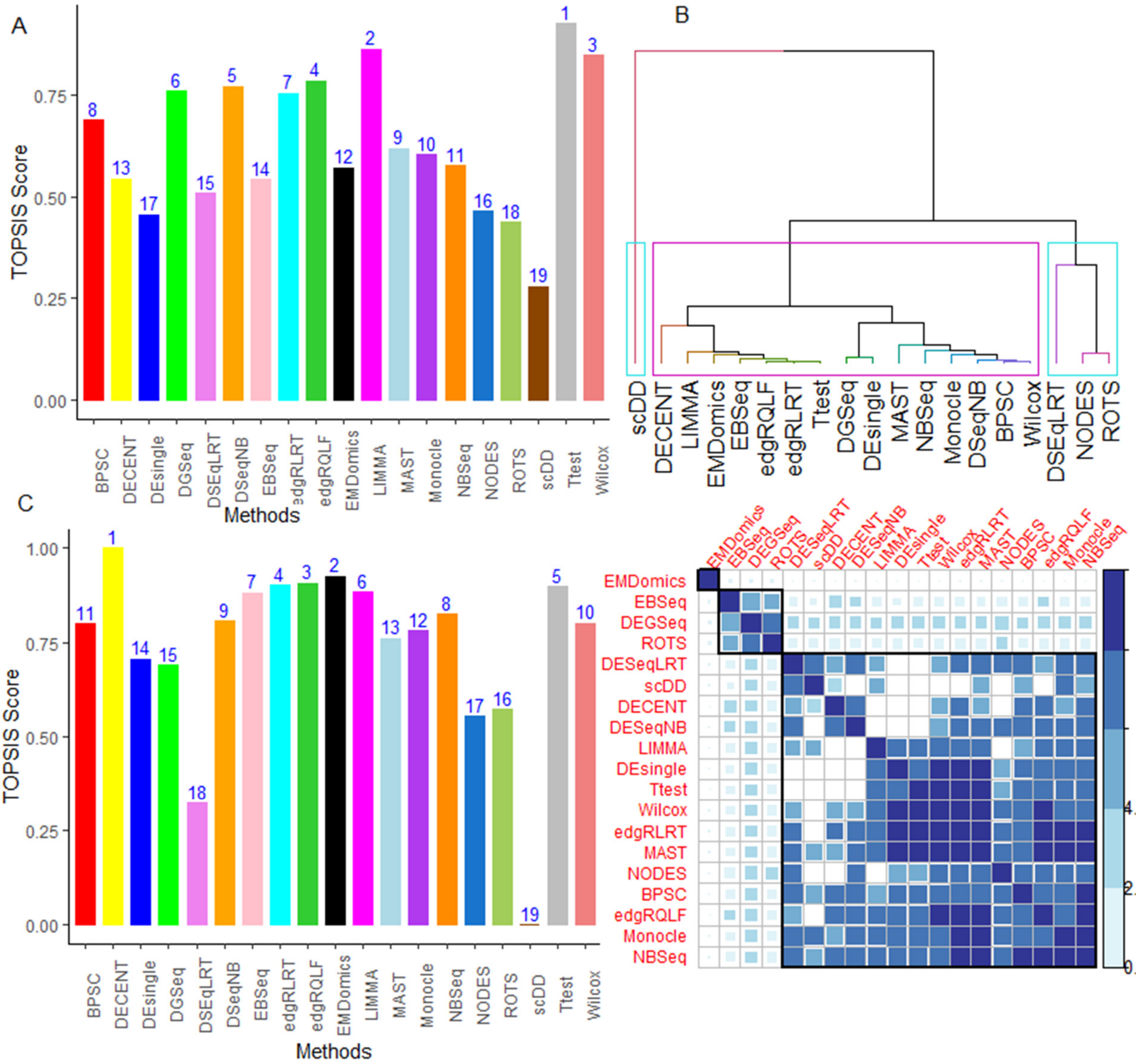

Figure S16. Performance evaluation of DE methods under the MCDM setup for Soumillion3 data. The comparative performance analysis of the 19 tested methods was carried out through TOPSIS approach under MCDM setup. X-axis represents the tested methods and Y-axis represents the TOPSIS scores. The results from

the (A) MCDM-TOPSIS analysis of the DE methods are shown for 12 performance metrics including runtime criterion; (B) Average similarities between the evaluated DE methods based on the 13-performance metrics. The dendrogram was obtained by average-linkage hierarchical clustering based on the matrix of average values of performance metrics over all gene sets; (C) MCDM-TOPSIS analysis of the DE methods based on 12 performance metrics excluding runtime criterion; (D) Similarity analysis of the tested methods based on their ability to detect common genes. The strengths of similarity scores are represented in various colors and white empty boxes represents non-significant values.

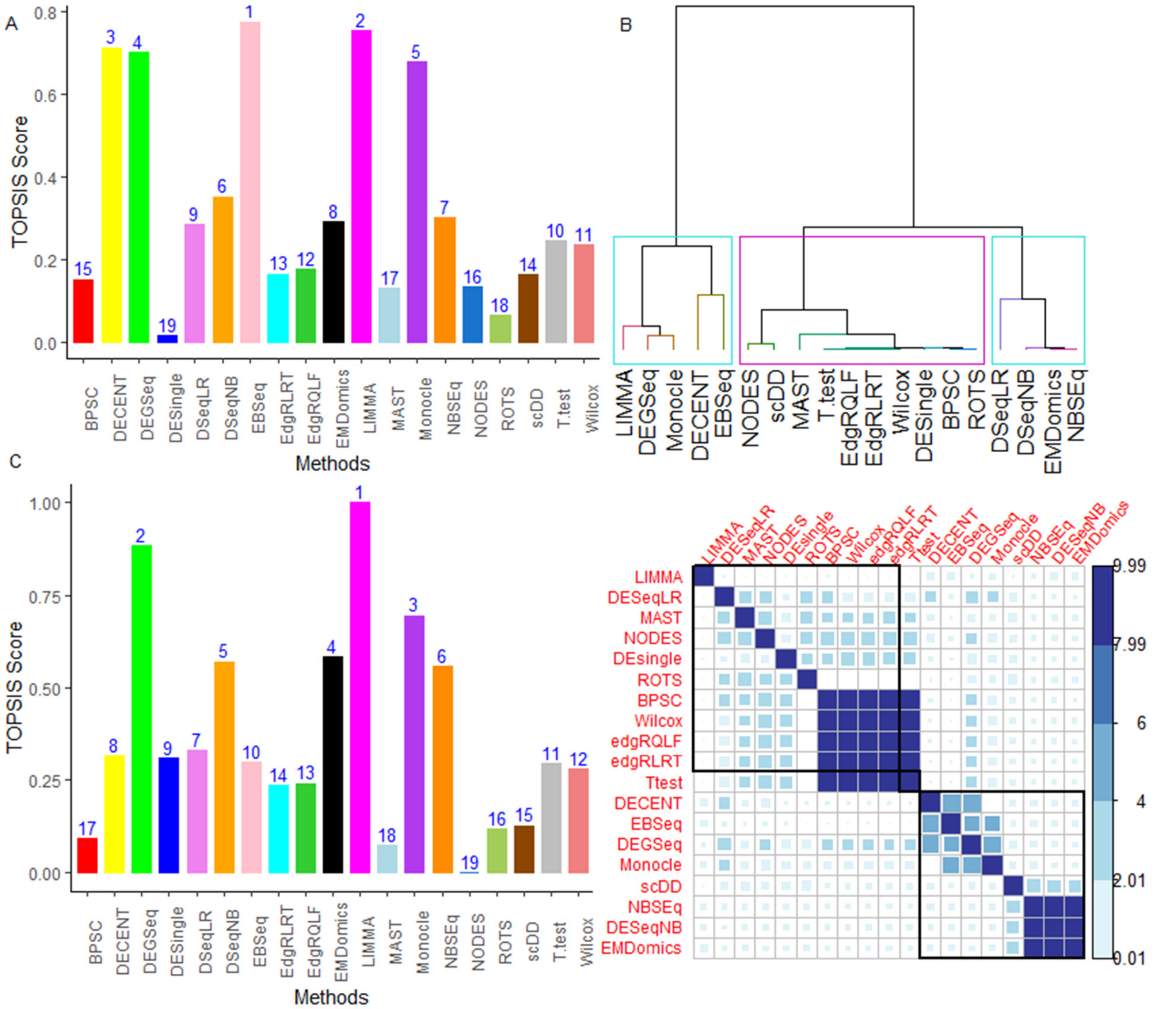

Figure S17. Performance evaluation of DE methods under the MCDM setup for Klein data.

The comparative performance analysis of the 19 tested methods was carried out through TOPSIS approach under MCDM setup on Klein dataset. The MCDM-TOPSIS analysis was carried out under two settings, i.e. (i) multi-criteria including runtime criterion; (ii) multi-criteria excluding runtime criterion. X-axis represents the tested methods and Y-axis represents the TOPSIS scores. The results from the (A) MCDM-TOPSIS analysis of the DE methods are shown for 12 performance metrics including runtime criterion; (B) Average similarities between the evaluated DE methods based on the 13-performance metrics. The dendrogram was obtained by average-linkage hierarchical clustering based on the matrix of average values of performance metrics over all gene sets; (C) MCDM-TOPSIS analysis of the DE

methods based on 12 performance metrics excluding runtime criterion; (D) Similarity analysis of the tested methods based on their ability to detect common genes. The strengths of similarity scores are represented in various colors and white empty boxes represent non-significant values at 1% level of significance.

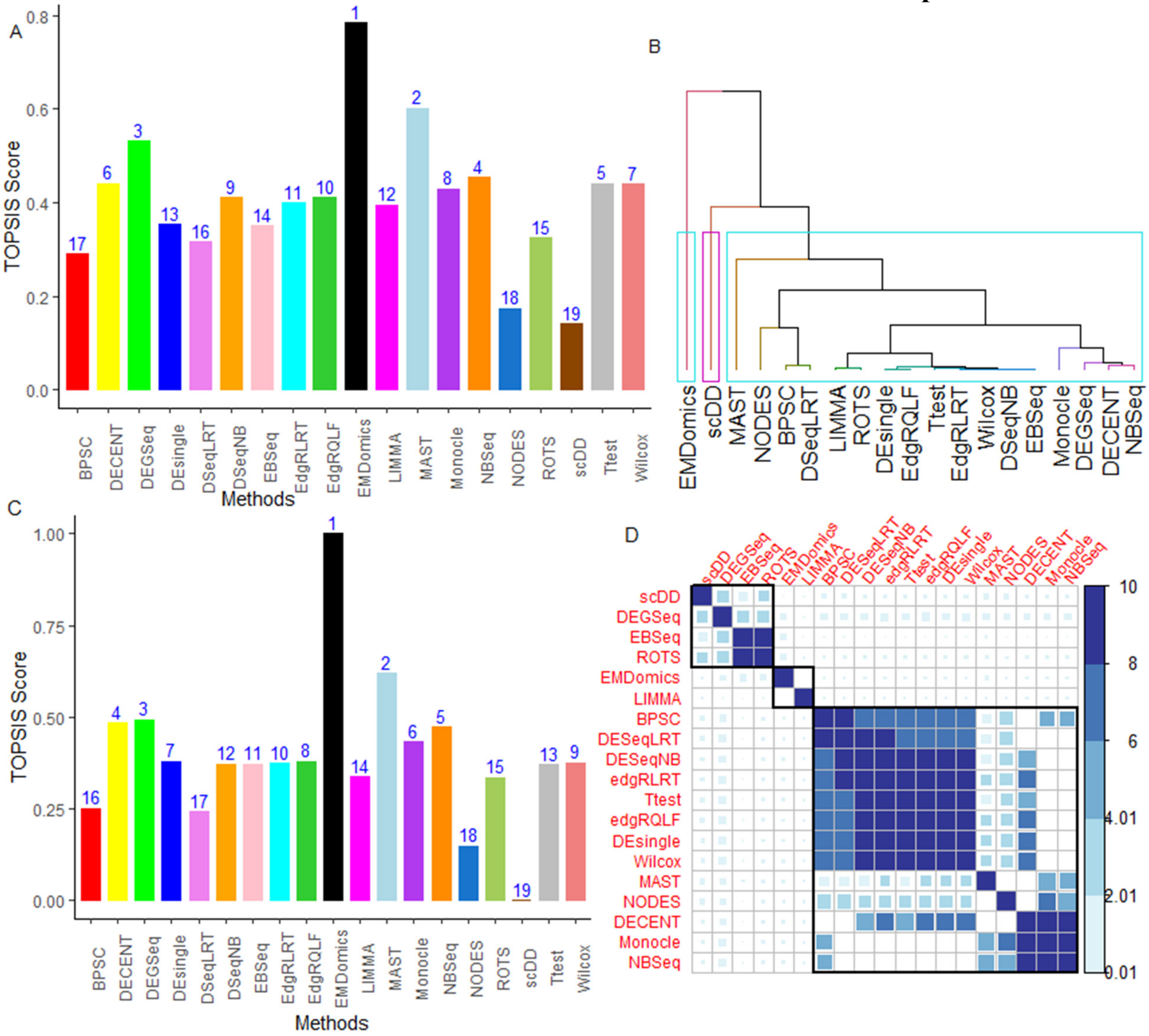

Figure S18. Performance evaluation of DE methods under the MCDM setup for Gierahn data. The comparative performance analysis of the 19 tested methods was carried out through TOPSIS approach under MCDM setup on Gierahn dataset. The MCDM-TOPSIS analysis was carried out under two settings, i.e. (i) multi-criteria including runtime; (ii) multi-criteria excluding runtime. X-axis represents the tested methods and Y-axis represents the TOPSIS scores. The results from the (A) MCDM-TOPSIS analysis of the DE methods are shown for 12 performance metrics including runtime criterion; (B) Average similarities between the evaluated DE methods based on the 13-performance metrics. The dendrogram was obtained by average-linkage hierarchical clustering based on the matrix of average values of performance metrics over all gene sets; (C) MCDM-TOPSIS analysis of the DE methods based on 12 performance metrics excluding runtime criterion; (D) Similarity analysis of the tested methods based on

their ability to detect common genes. The strengths of similarity scores are represented in various colors and white empty boxes represents non-significant values at 1% level of significance.

Figure S19. Performance evaluation of DE methods under the MCDM setup for Chen data.

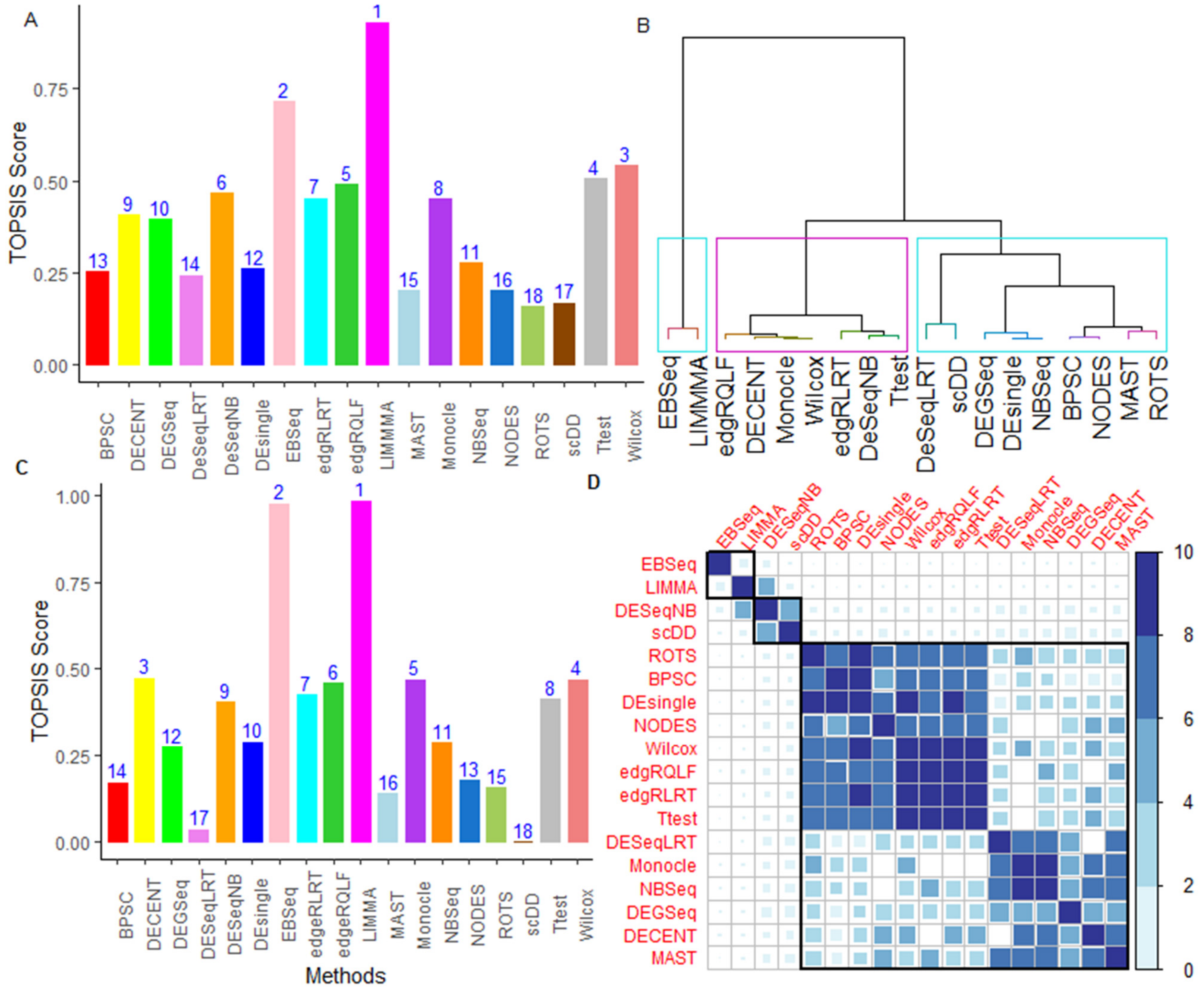

The comparative performance analysis of the 19 tested methods was carried out through TOPSIS approach under MCDM setup on Chen dataset. The MCDM-TOPSIS analysis was carried out under two settings, i.e. (i) multi-criteria including runtime criterion; (ii) multi-criteria excluding runtime criterion. X-axis represents the tested methods and Y-axis represents the TOPSIS scores. The results from the (A) MCDM-TOPSIS analysis of the DE methods are shown for 12 performance metrics including runtime criterion; (B) Average similarities between the evaluated DE methods based on the 13-performance metrics. The dendrogram was obtained by average-linkage hierarchical clustering based on the matrix of average values of performance metrics over all gene sets; (C) MCDM-TOPSIS analysis of the DE methods based on 12 performance metrics excluding runtime criterion; (D) Similarity analysis of the tested methods

based on their ability to detect common genes. The strengths of similarity scores are represented in various colors and white empty boxes represents non-significant values at 1% level of significance.

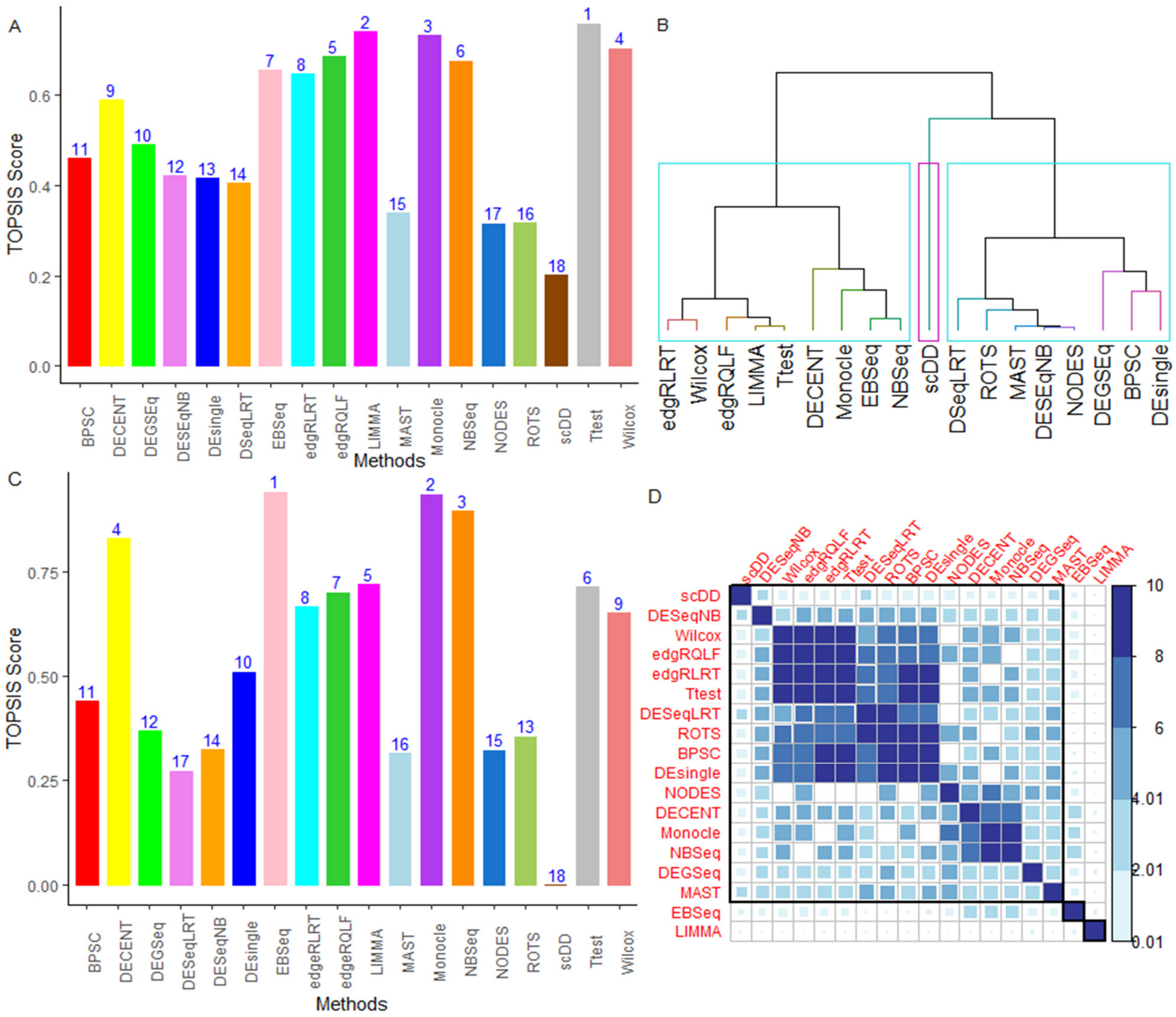

Figure S20. Performance evaluation of DE methods under the MCDM setup for Savas data. The comparative performance analysis of the 19 tested methods was carried out through TOPSIS approach under MCDM setup on Savas dataset. The MCDM-TOPSIS analysis was carried out under two settings, *i.e.*, (i) multi-criteria including runtime criterion; (ii) multi-criteria excluding runtime criterion. X-axis represents the tested methods and Y-axis represents the TOPSIS scores. The results from the (A) MCDM-TOPSIS analysis of the DE methods are shown for 12 performance metrics including runtime criterion; (B) Average similarities between the evaluated DE methods based on the 13-performance metrics. The dendrogram was obtained by average-linkage hierarchical clustering based on the matrix of average values of performance metrics over all gene sets; (C) MCDM-TOPSIS analysis of the DE methods based on 12 performance metrics excluding runtime criterion; (D) Similarity analysis of the tested methods based on their ability to detect common genes. The strengths of similarity scores are represented in various colors and

white empty boxes represents non-significant values similarity scores are represented in various colors and white empty boxes represent non-significant values at 1% level of significance.

Figure S21. Performance evaluation of DE methods under the MCDM setup for Grun data.

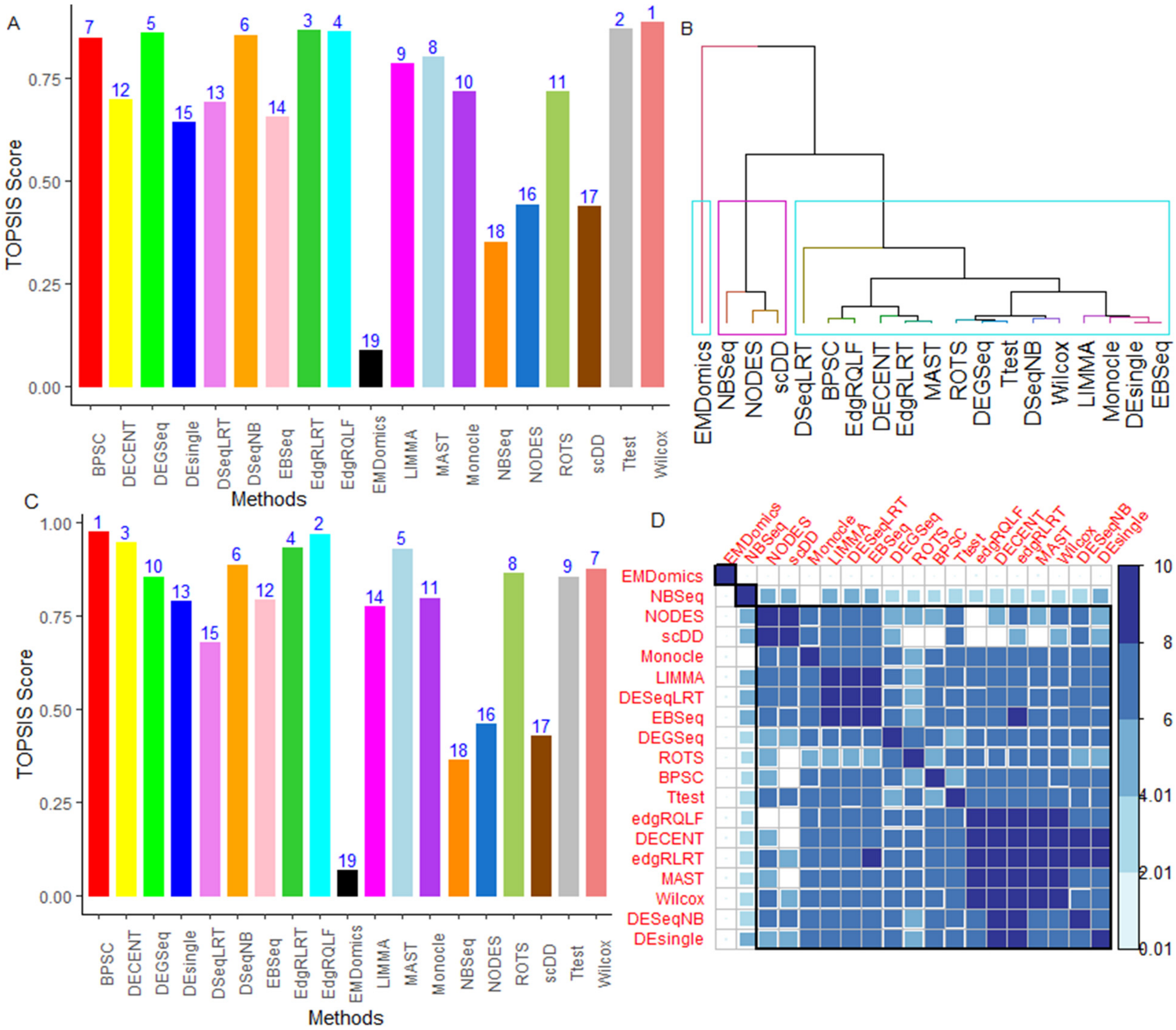

The comparative performance analysis of the 19 tested methods was carried out through TOPSIS approach under MCDM setup on Grun dataset. The MCDM-TOPSIS analysis was carried out under two settings, i.e. (i) multi-criteria including runtime criterion; (ii) multi-criteria excluding runtime criterion. X-axis represents the tested methods and Y-axis represents the TOPSIS scores. The results from the (A) MCDM-TOPSIS analysis of the DE methods are shown for 12 performance metrics including runtime criterion; (B) Average similarities between the evaluated DE methods based on the 13-performance metrics. The dendrogram was obtained by average-linkage hierarchical clustering based on the matrix of average values of performance metrics over all gene sets; (C) MCDM-TOPSIS analysis of the DE methods based on 12 performance metrics excluding runtime criterion; (D) Similarity analysis of the tested methods based on their ability to detect common genes. The strengths of similarity scores are represented in various colors and white empty boxes represent non-significant values at 1% level of significance.

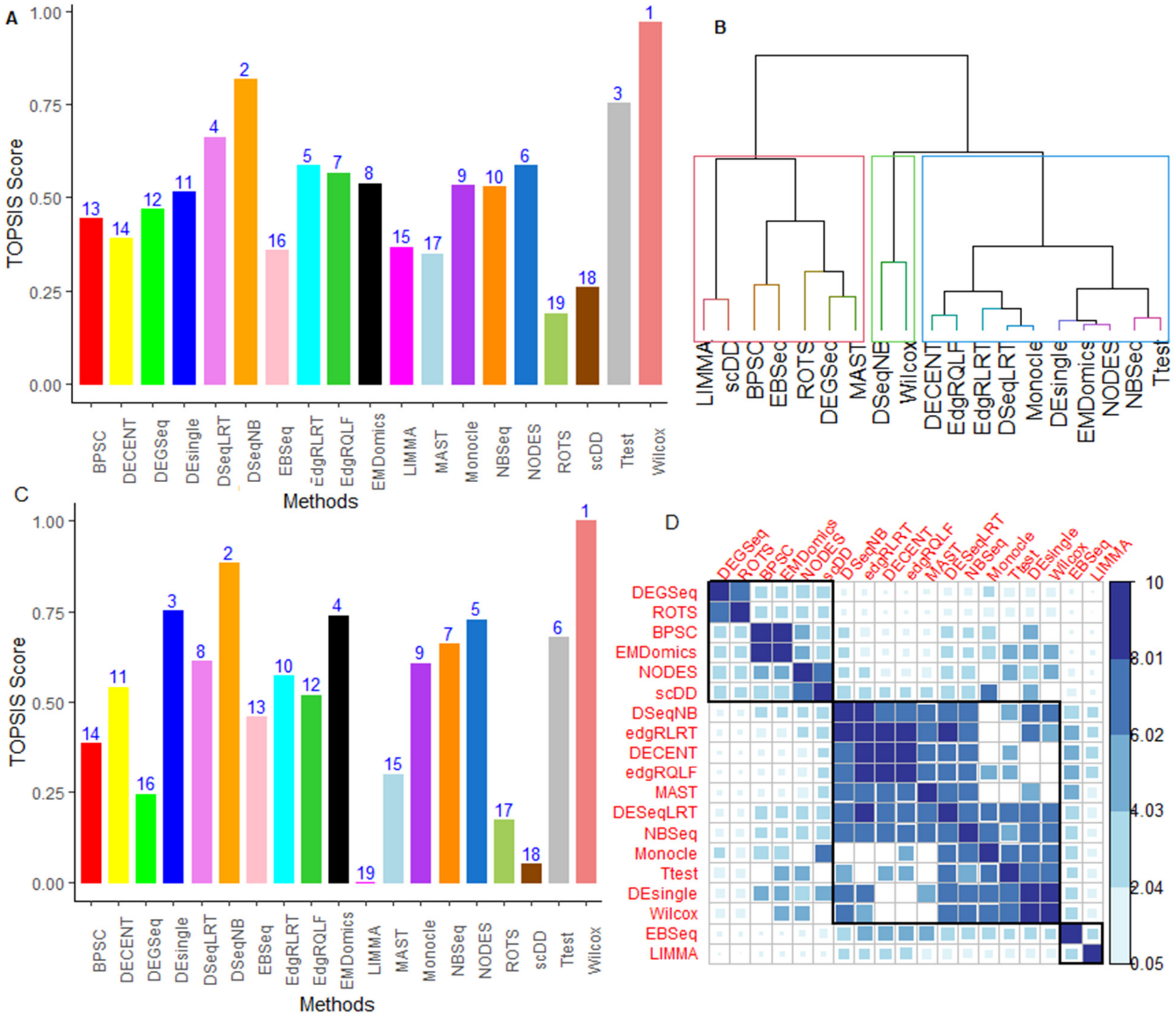

Figure S22. Performance evaluation of DE methods under the MCDM for Ziegenhain data. The comparative performance analysis of the 19 tested methods was carried out through TOPSIS approach under MCDM setup on Ziegenhain dataset. The MCDM-TOPSIS analysis was carried out under two settings, i.e. (i) multi-criteria including runtime criterion; (ii) multi-criteria excluding runtime criterion. X-axis represents the tested methods and Y-axis represents the TOPSIS scores. The results from the (A) MCDM-TOPSIS analysis of the DE methods are shown for 12 performance metrics including runtime criterion; (B) Average similarities between the evaluated DE methods based on the 13-performance metrics. The dendrogram was obtained by average-linkage hierarchical clustering based on the matrix of average values of performance metrics over all gene sets; (C) MCDM-TOPSIS analysis of the DE methods based on 12 performance metrics excluding runtime criterion; (D) Similarity analysis of the tested methods based on their ability to detect common genes. The strengths of similarity scores are represented in various colors and white empty boxes represents non-significant values at 1% level of significance.

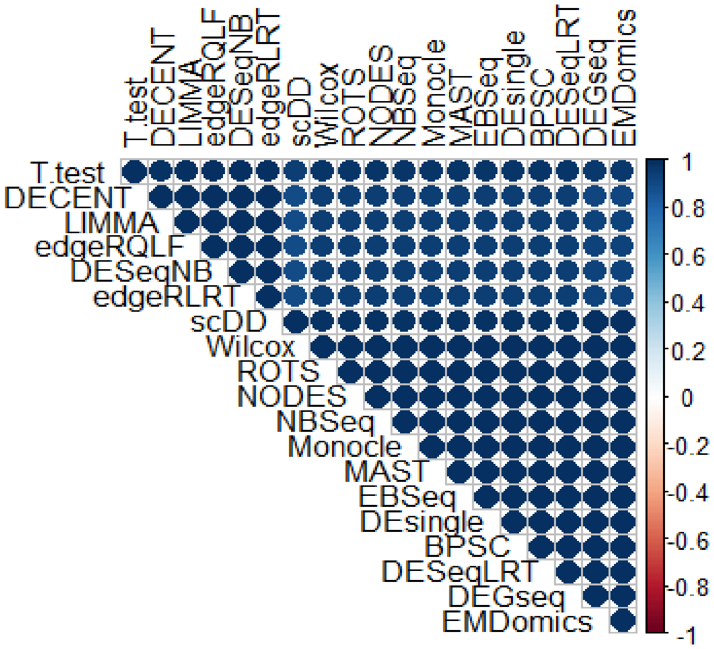

Figure S23. Correlation plot for the tested methods over the computed performance metrics for Soumillon2 data. The correlation measures are computed through Spearman's rank correlation and the correlation measures are shown for *p-value* < 0.01.

Figure S24. Correlation plot for the tested methods over the computed performance metrics for the real scRNA-seq data. The correlation plot is shown for (A) Islam (B) Tung (C) Ziegenhain (D) Soumillon1 (E) Soumillon3 (F) Klein (G) Grun (H) Gierahn. The correlation measures are computed through Spearman's rank correlation and the correlation measures are shown for *p-value* < 0.01.

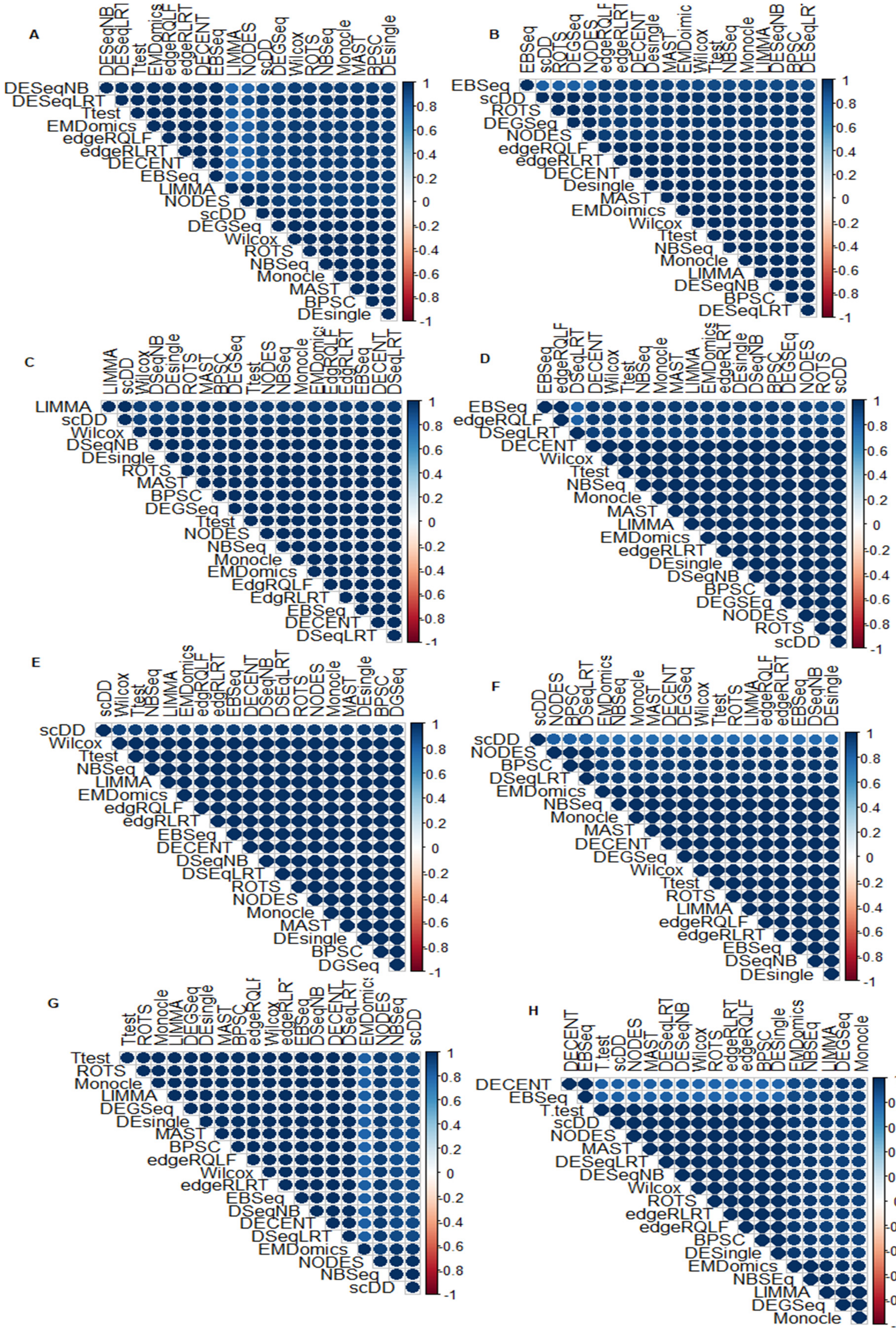
A
DESeqNB DESeqLRT Ttest EMDomics edgeRQLF edgeRLRT DECENT EBSeq LIMMA NODES scDD DEGSeq Wilcox ROTS NBSeq Monocle MAST BPSC DEsingle
B
EBSeq scDD ROTS DEGSeq NODES edgeRQLF edgeRLRT DECENT Desingle MAST EMDoimics Wilcox Ttest NBSeq Monocle LIMMA DESeqNB BPSC DESeqLRT
C
LIMMA scDD Wilcox DSeqNB DEsingle ROTS MAST BPSC DEGSeq Ttest NODES NBSeq Monocle EMDomics EdgRQLF EdgRLRT EBSeq DECENT DSeqLRT
D
EBSeq edgeRQLF DSeqLRT DECENT Wilcox Ttest NBSeq Monocle MAST LIMMA EMDomics edgeRLRT DEsingle DSeqNB BPSC DEGSEq NODES ROTS scDD
E
scDD Wilcox Ttest NBSeq LIMMA EMDomics edgRQLF edgRLRT EBSeq DECENT DSeqNB DSEqLRT ROTS NODES Monocle MAST DEsingle BPSC DGSeq
F
scDD NODES BPSC DSeqLRT EMDomics NBSeq Monocle MAST DECENT DEGSeq Wilcox Ttest ROTS LIMMA edgeRQLF edgeRLRT EBSeq DSeqNB DEsingle
G
Ttest ROTS Monocle LIMMA DEGSeq DEsingle MAST BPSC edgeRQLF Wilcox edgeRLRT EBSeq DSeqNB DECENT DSeqLRT EMDomics NODES NBSeq scDD
H
DECENT EBSeq T.test scDD NODES MAST DESeqLRT DESeqNB Wilcox ROTS edgeRLRT edgeRQLF BPSC DESingle EMDomics NBSEq LIMMA DEGSeq Monocle
1 0.8 0.6 0.4 0.2 0 -0.2 -0.4 -0.6 -0.8 -1

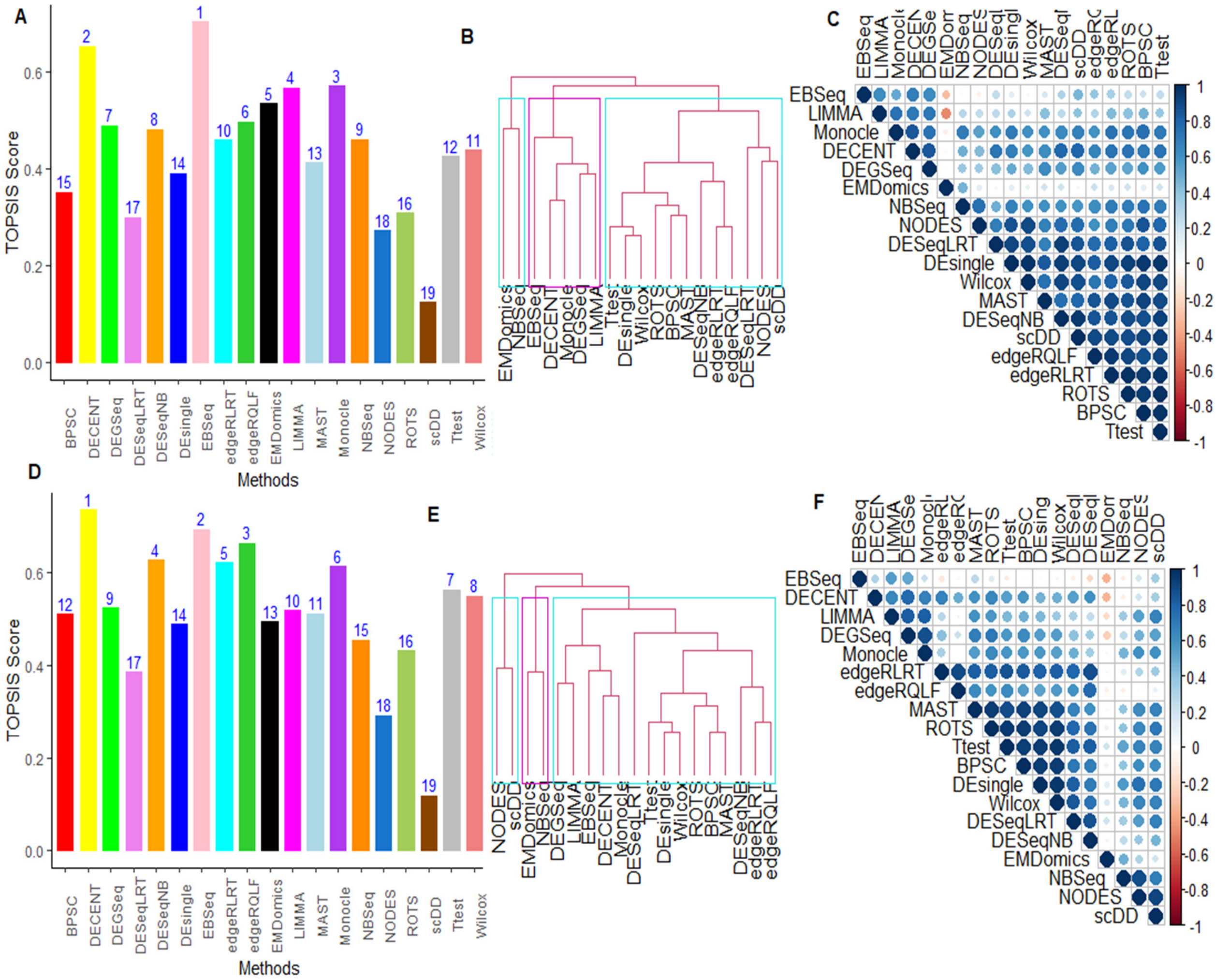

Figure S25. Combined data analysis of the DE methods based on TPR and FPR metrics through TOPSIS Approach. The comparative performance evaluation of the DE methods was performed based on TPR and FPR metrics through TOPSIS approach under multi-data setup. This analysis was performed on data matrix having TPR and FPR scores of the tested methods across the 11 considered datasets. (A) Shows results from TOPSIS analysis of the tested DE methods based on TPR; (B) Similarity analysis of the evaluated DE methods based on TPR through clustering. The dendrogram was obtained by average-linkage hierarchical clustering based on the matrix of average (over DE gene sets) TPR scores across all data sets. (C) Similarity analysis of the evaluated DE methods based on TPR through correlation. The correlation plot was obtained by Spearman's rank correlation method based on the matrix of average (over DE gene sets) TPR scores across all data sets; (D) Shows results from TOPSIS analysis of the tested DE methods based on FPR; (E) Similarity analysis of the evaluated DE methods based on FPR through clustering. The dendrogram was obtained by average-linkage hierarchical clustering based on the matrix of

average (over DE gene sets) FPR across all data sets. (F) Similarity analysis of the evaluated DE methods based on FPR metrics through correlation. The correlation plot was obtained by Spearman's rank correlation method based on the matrix of average (over DE gene sets) FPR scores across all data sets.

Figure S26. Combined data analysis of the DE methods based on AUROC metrics through

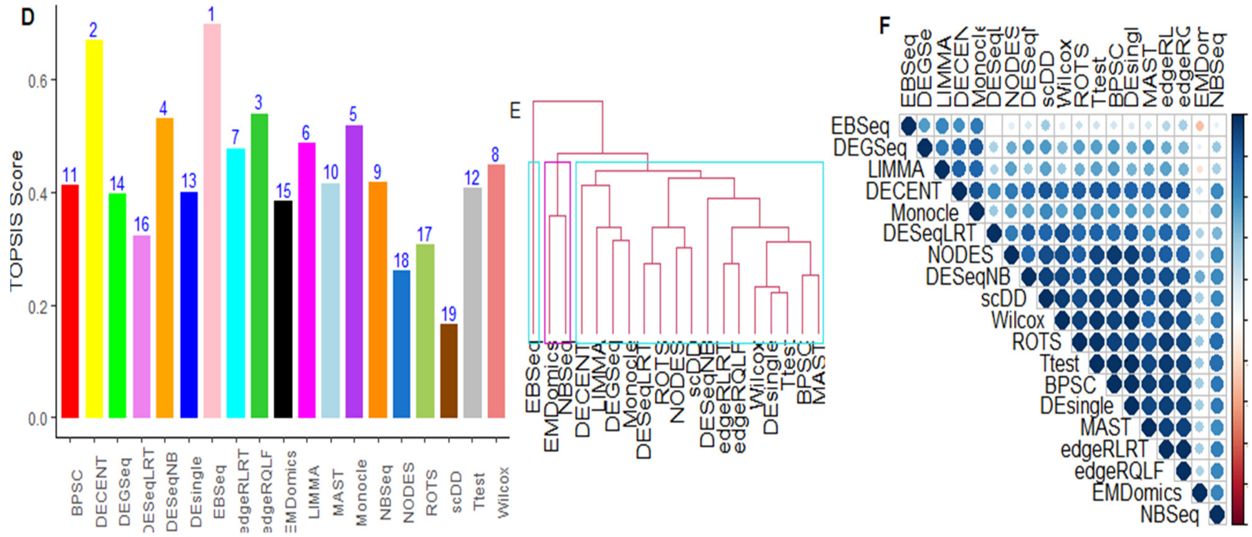

TOPSIS Approach. The comparative performance evaluation of the DE methods was performed based on AUROC metrics through TOPSIS approach under multi-data setup. This analysis was performed on data matrix having AUROC scores of the tested methods across the 11 considered datasets. (A) Shows results from TOPSIS analysis of the tested DE methods based on AUROC; (B) Similarity analysis of the evaluated DE methods based on AUROC through clustering. The dendrogram was obtained by average-linkage hierarchical clustering based on the matrix of average (over DE gene sets) AUROC scores across all data sets. (C) Similarity analysis of the evaluated DE methods based on AUROC through correlation. The correlation plot was obtained by Spearman's rank correlation method based on the matrix of average (over DE gene sets) AUROC scores across all data sets.

Table S8. Evaluation of DE methods based on performance evaluation metrics for Soumillon2 scRNA-seq data.

Methods	TP	FP	TN	FN	TPR	FPR	FDR	PPR	NPV	ACC	F1	AUROC

DEG = 500												
BPSC	351	149	12486	2649	0.117	0.012	0.298	0.702	0.825	0.821	0.201	0.472
DECENT	490	10	12625	2510	0.163	0.001	0.020	0.980	0.834	0.839	0.280	0.541
DEGseq	173	327	12308	2827	0.058	0.026	0.654	0.346	0.813	0.798	0.099	0.430
DESeqNB	443	57	12578	2557	0.148	0.005	0.114	0.886	0.831	0.833	0.253	0.513
DESeqLRT	308	192	12443	2692	0.103	0.015	0.384	0.616	0.822	0.816	0.176	0.461
DEsingle	324	176	12459	2676	0.108	0.014	0.352	0.648	0.823	0.818	0.185	0.457
EBSeq	263	237	12398	2737	0.088	0.019	0.474	0.526	0.819	0.810	0.150	0.441
edgeRLRT	339	161	12474	2661	0.113	0.013	0.322	0.678	0.824	0.820	0.194	0.467
edgeRQLF	414	86	12549	2586	0.138	0.007	0.172	0.828	0.829	0.829	0.237	0.479
EMDomics	331	169	12466	2669	0.110	0.013	0.338	0.662	0.824	0.818	0.189	0.484
LIMMA	347	153	12482	2653	0.116	0.012	0.306	0.694	0.825	0.821	0.198	0.458
MAST	309	191	12444	2691	0.103	0.015	0.382	0.618	0.822	0.816	0.177	0.461
Monocle	283	217	12418	2717	0.094	0.017	0.434	0.566	0.820	0.812	0.162	0.448
NBSeq	335	165	12470	2665	0.112	0.013	0.330	0.670	0.824	0.819	0.191	0.463
NODES	221	279	12356	2779	0.074	0.022	0.558	0.442	0.816	0.804	0.126	0.439
ROTS	231	269	12366	2769	0.077	0.021	0.538	0.462	0.817	0.806	0.132	0.441
scDD	175	325	12310	2825	0.058	0.026	0.650	0.350	0.813	0.799	0.100	0.433
T-test	322	178	12457	2678	0.107	0.014	0.356	0.644	0.823	0.817	0.184	0.461
Wilcox	309	191	12444	2691	0.103	0.015	0.382	0.618	0.822	0.816	0.177	0.459
DEG = 1000												
BPSC	639	361	12274	2361	0.213	0.029	0.361	0.639	0.839	0.826	0.320	0.529
DECENT	914	86	12549	2086	0.305	0.007	0.086	0.914	0.857	0.861	0.457	0.641
DEGseq	368	632	12003	2632	0.123	0.050	0.632	0.368	0.820	0.791	0.184	0.461
DESeqNB	813	187	12448	2187	0.271	0.015	0.187	0.813	0.851	0.848	0.407	0.586
DESeqLRT	555	445	12190	2445	0.185	0.035	0.445	0.555	0.833	0.815	0.278	0.509
DEsingle	620	380	12255	2380	0.207	0.030	0.380	0.620	0.837	0.823	0.310	0.513
EBSeq	553	447	12188	2447	0.184	0.035	0.447	0.553	0.833	0.815	0.277	0.487
edgeRLRT	650	350	12285	2350	0.217	0.028	0.350	0.650	0.839	0.827	0.325	0.525
edgeRQLF	761	239	12396	2239	0.254	0.019	0.239	0.761	0.847	0.842	0.381	0.557
EMDomics	461	539	12096	2539	0.154	0.043	0.539	0.461	0.827	0.803	0.231	0.521
LIMMA	683	317	12318	2317	0.228	0.025	0.317	0.683	0.842	0.832	0.342	0.516
MAST	591	409	12226	2409	0.197	0.032	0.409	0.591	0.835	0.820	0.296	0.511
Monocle	573	427	12208	2427	0.191	0.034	0.427	0.573	0.834	0.817	0.287	0.497
NBSeq	638	362	12273	2362	0.213	0.029	0.362	0.638	0.839	0.826	0.319	0.519
NODES	433	567	12068	2567	0.144	0.045	0.567	0.433	0.825	0.800	0.217	0.474
ROTS	472	528	12107	2528	0.157	0.042	0.528	0.472	0.827	0.805	0.236	0.479
scDD	325	675	11960	2675	0.108	0.053	0.675	0.325	0.817	0.786	0.163	0.459
T-test	627	373	12262	2373	0.209	0.030	0.373	0.627	0.838	0.824	0.314	0.514
Wilcox	594	406	12229	2406	0.198	0.032	0.406	0.594	0.836	0.820	0.297	0.509
DEG = 1500												
BPSC	906	594	12041	2094	0.302	0.047	0.396	0.604	0.852	0.828	0.403	0.580

DECENT	1211	289	12346	1789	0.404	0.023	0.193	0.807	0.873	0.867	0.538	0.728
DEGseq	556	944	11691	2444	0.185	0.075	0.629	0.371	0.827	0.783	0.247	0.492
DESeqNB	1096	404	12231	1904	0.365	0.032	0.269	0.731	0.865	0.852	0.487	0.659
DESeqLRT	773	727	11908	2227	0.258	0.058	0.485	0.515	0.842	0.811	0.344	0.554
DEsingle	882	618	12017	2118	0.294	0.049	0.412	0.588	0.850	0.825	0.392	0.566
EBSeq	785	715	11920	2215	0.262	0.057	0.477	0.523	0.843	0.813	0.349	0.538
edgeRLRT	934	566	12069	2066	0.311	0.045	0.377	0.623	0.854	0.832	0.415	0.578
edgeRQLF	980	520	12115	2020	0.327	0.041	0.347	0.653	0.857	0.838	0.436	0.631
EMDomics	538	962	11673	2462	0.179	0.076	0.641	0.359	0.826	0.781	0.239	0.540
LIMMA	1011	489	12146	1989	0.337	0.039	0.326	0.674	0.859	0.842	0.449	0.573
MAST	828	672	11963	2172	0.276	0.053	0.448	0.552	0.846	0.818	0.368	0.560
Monocle	843	657	11978	2157	0.281	0.052	0.438	0.562	0.847	0.820	0.375	0.546
NBSeq	903	597	12038	2097	0.301	0.047	0.398	0.602	0.852	0.828	0.401	0.574
NODES	681	819	11816	2319	0.227	0.065	0.546	0.454	0.836	0.799	0.303	0.512
ROTS	623	877	11758	2377	0.208	0.069	0.585	0.415	0.832	0.792	0.277	0.519
scDD	453	1047	11588	2547	0.151	0.083	0.698	0.302	0.820	0.770	0.201	0.484
T-test	912	588	12047	2088	0.304	0.047	0.392	0.608	0.852	0.829	0.405	0.567
Wilcox	837	663	11972	2163	0.279	0.052	0.442	0.558	0.847	0.819	0.372	0.561
							DEG = 2000					
BPSC	1131	869	11766	1869	0.377	0.069	0.435	0.566	0.863	0.825	0.452	0.631
DECENT	1400	600	12035	1600	0.467	0.047	0.300	0.700	0.883	0.859	0.560	0.784
DEGseq	746	1254	11381	2254	0.249	0.099	0.627	0.373	0.835	0.776	0.298	0.523
DESeqNB	1341	659	11976	1659	0.447	0.052	0.330	0.671	0.878	0.852	0.536	0.716
DESeqLRT	948	1052	11583	2052	0.316	0.083	0.526	0.474	0.850	0.801	0.379	0.595
DEsingle	1107	893	11742	1893	0.369	0.071	0.447	0.554	0.861	0.822	0.443	0.618
EBSeq	928	1072	11563	2072	0.309	0.085	0.536	0.464	0.848	0.799	0.371	0.588
edgeRLRT	1167	833	11802	1833	0.389	0.066	0.417	0.584	0.866	0.829	0.467	0.634
edgeRQLF	1189	811	11824	1811	0.396	0.064	0.406	0.595	0.867	0.832	0.476	0.679
EMDomics	661	1339	11296	2339	0.220	0.106	0.670	0.331	0.828	0.765	0.264	0.557
LIMMA	1321	679	11956	1679	0.440	0.054	0.340	0.661	0.877	0.849	0.528	0.633
MAST	1028	972	11663	1972	0.343	0.077	0.486	0.514	0.855	0.812	0.411	0.606
Monocle	1080	920	11715	1920	0.360	0.073	0.460	0.540	0.859	0.818	0.432	0.597
NBSeq	1138	862	11773	1862	0.379	0.068	0.431	0.569	0.863	0.826	0.455	0.625
NODES	836	1164	11471	2164	0.279	0.092	0.582	0.418	0.841	0.787	0.334	0.554
ROTS	841	1159	11476	2159	0.280	0.092	0.580	0.421	0.842	0.788	0.336	0.551
scDD	561	1439	11196	2439	0.187	0.114	0.720	0.281	0.821	0.752	0.224	0.508
T-test	1144	856	11779	1856	0.381	0.068	0.428	0.572	0.864	0.827	0.458	0.623
Wilcox	1073	927	11708	1927	0.358	0.073	0.464	0.537	0.859	0.817	0.429	0.607
							DEG = 2500					
BPSC	1338	1162	11473	1662	0.446	0.092	0.465	0.535	0.873	0.819	0.487	0.677
DECENT	1553	947	11688	1447	0.518	0.075	0.379	0.621	0.890	0.847	0.565	0.823
DEGseq	998	1502	11133	2002	0.333	0.119	0.601	0.399	0.848	0.776	0.363	0.549
DESeqNB	1509	991	11644	1491	0.503	0.078	0.396	0.604	0.886	0.841	0.549	0.769

DESeqLRT	1117	1383	11252	1883	0.372	0.109	0.553	0.447	0.857	0.791	0.406	0.631
DEsingle	1281	1219	11416	1719	0.427	0.096	0.488	0.512	0.869	0.812	0.466	0.667
EBSeq	1020	1480	11155	1980	0.340	0.117	0.592	0.408	0.849	0.779	0.371	0.626
edgeRLRT	1364	1136	11499	1636	0.455	0.090	0.454	0.546	0.875	0.823	0.496	0.685
edgeRQLF	1362	1138	11497	1638	0.454	0.090	0.455	0.545	0.875	0.822	0.495	0.721
EMDomics	767	1733	10902	2233	0.256	0.137	0.693	0.307	0.830	0.746	0.279	0.576
LIMMA	1492	1008	11627	1508	0.497	0.080	0.403	0.597	0.885	0.839	0.543	0.711
MAST	1191	1309	11326	1809	0.397	0.104	0.524	0.476	0.862	0.801	0.433	0.648
Monocle	1287	1213	11422	1713	0.429	0.096	0.485	0.515	0.870	0.813	0.468	0.645
NBSeq	1328	1172	11463	1672	0.443	0.093	0.469	0.531	0.873	0.818	0.483	0.675
NODES	1018	1482	11153	1982	0.339	0.117	0.593	0.407	0.849	0.778	0.370	0.586
ROTS	999	1501	11134	2001	0.333	0.119	0.600	0.400	0.848	0.776	0.363	0.587
scDD	640	1860	10775	2360	0.213	0.147	0.744	0.256	0.820	0.730	0.233	0.529
T-test	1338	1162	11473	1662	0.446	0.092	0.465	0.535	0.873	0.819	0.487	0.674
Wilcox	1258	1242	11393	1742	0.419	0.098	0.497	0.503	0.867	0.809	0.457	0.654
						DEG = 3000						
BPSC	1478	1522	11113	1522	0.493	0.120	0.507	0.493	0.880	0.805	0.493	0.722
DECENT	1674	1326	11309	1326	0.558	0.105	0.442	0.558	0.895	0.830	0.558	0.857
DEGseq	1228	1772	10863	1772	0.409	0.140	0.591	0.409	0.860	0.773	0.409	0.585
DESeqNB	1653	1347	11288	1347	0.551	0.107	0.449	0.551	0.893	0.828	0.551	0.811
DESeqLRT	1247	1753	10882	1753	0.416	0.139	0.584	0.416	0.861	0.776	0.416	0.666
DEsingle	1428	1572	11063	1572	0.476	0.124	0.524	0.476	0.876	0.799	0.476	0.709
EBSeq	1110	1890	10745	1890	0.370	0.150	0.630	0.370	0.850	0.758	0.370	0.654
edgeRLRT	1537	1463	11172	1463	0.512	0.116	0.488	0.512	0.884	0.813	0.512	0.729
edgeRQLF	1506	1494	11141	1494	0.502	0.118	0.498	0.502	0.882	0.809	0.502	0.758
EMDomics	844	2156	10479	2156	0.281	0.171	0.719	0.281	0.829	0.724	0.281	0.594
LIMMA	1612	1388	11247	1388	0.537	0.110	0.463	0.537	0.890	0.822	0.537	0.768
MAST	1337	1663	10972	1663	0.446	0.132	0.554	0.446	0.868	0.787	0.446	0.685
Monocle	1454	1546	11089	1546	0.485	0.122	0.515	0.485	0.878	0.802	0.485	0.691
NBSeq	1497	1503	11132	1503	0.499	0.119	0.501	0.499	0.881	0.808	0.499	0.718
NODES	1173	1827	10808	1827	0.391	0.145	0.609	0.391	0.855	0.766	0.391	0.620
ROTS	1170	1830	10805	1830	0.390	0.145	0.610	0.390	0.855	0.766	0.390	0.618
scDD	697	2303	10332	2303	0.232	0.182	0.768	0.232	0.818	0.705	0.232	0.547
T-test	1501	1499	11136	1499	0.500	0.119	0.500	0.500	0.881	0.808	0.500	0.719
Wilcox	1413	1587	11048	1587	0.471	0.126	0.529	0.471	0.874	0.797	0.471	0.695

TP: True Positives; FP: False Positives; TN: True Negatives; FN: False Negatives; TPR: True Positive Rate; FPR: False Positive Rate; FDR: False Discovery Rate; PPR: Positive Prediction Rate; NPV: Negative Prediction Value; ACC: Accuracy; F1: F1 score; AUROC: Area Under Receiver Operating Curve

Table S9. Evaluation of DE methods based on performance evaluation metrics for Islam scRNA-seq data.

	TP	FP	TN	FN	TPR	FPR	FDR	PPR	NPV	ACC	F1	AUROC

Methods				DEG = 500								
BPSC	300	200	8236	2700	0.100	0.024	0.400	0.600	0.753	0.746	0.171	0.450
DECENT	370	130	8306	2630	0.123	0.015	0.260	0.740	0.760	0.759	0.211	0.466
DEGSeq	190	310	8126	2810	0.063	0.037	0.620	0.380	0.743	0.727	0.109	0.431
DESeqLRT	353	147	8289	2647	0.118	0.017	0.294	0.706	0.758	0.756	0.202	0.470
DESeqNB	484	16	8420	2516	0.161	0.002	0.032	0.968	0.770	0.779	0.277	0.442
DEsingle	346	154	8282	2654	0.115	0.018	0.308	0.692	0.757	0.754	0.198	0.469
EBSeq	269	231	8205	2731	0.090	0.027	0.462	0.538	0.750	0.741	0.154	0.446
edgeRLRT	378	113	8323	2613	0.129	0.013	0.226	0.774	0.761	0.762	0.221	0.456
edgeRQLF	387	122	8314	2622	0.126	0.014	0.244	0.756	0.760	0.760	0.216	0.452
EMDomics	313	187	8249	2687	0.104	0.022	0.374	0.626	0.754	0.749	0.179	0.456
LIMMA	291	209	8227	2709	0.097	0.025	0.418	0.582	0.752	0.745	0.166	0.462
MAST	285	215	8221	2715	0.095	0.025	0.430	0.570	0.752	0.744	0.163	0.456
Monocle	278	222	8214	2722	0.093	0.026	0.444	0.556	0.751	0.743	0.159	0.446
NBSeq	317	183	8253	2683	0.106	0.022	0.366	0.634	0.755	0.749	0.181	0.464
NODES	181	319	8117	2819	0.060	0.038	0.638	0.362	0.742	0.726	0.103	0.436
ROTS	282	218	8218	2718	0.094	0.026	0.436	0.564	0.751	0.743	0.161	0.445
scDD	205	295	8141	2795	0.068	0.035	0.590	0.410	0.744	0.730	0.117	0.439
T-test	310	190	8246	2690	0.103	0.023	0.380	0.620	0.754	0.748	0.177	0.459
Wilcox	398	102	8334	2602	0.133	0.012	0.204	0.796	0.762	0.764	0.227	0.479
						DEG = 1000						
BPSC	470	530	7906	2530	0.157	0.063	0.530	0.470	0.758	0.732	0.235	0.509
DECENT	691	309	8127	2309	0.230	0.037	0.309	0.691	0.779	0.771	0.346	0.533
DEGSeq	374	626	7810	2626	0.125	0.074	0.626	0.374	0.748	0.716	0.187	0.463
DESeqLRT	652	348	8088	2348	0.217	0.041	0.348	0.652	0.775	0.764	0.326	0.528
DESeqNB	961	39	8397	2039	0.320	0.005	0.039	0.961	0.805	0.818	0.481	0.569
DEsingle	576	424	8012	2424	0.192	0.050	0.424	0.576	0.768	0.751	0.288	0.525
EBSeq	533	467	7969	2467	0.178	0.055	0.467	0.533	0.764	0.743	0.267	0.489
edgeRLRT	758	242	8194	2242	0.253	0.029	0.242	0.758	0.785	0.783	0.379	0.534
edgeRQLF	808	192	8244	2192	0.269	0.023	0.192	0.808	0.790	0.792	0.404	0.505
EMDomics	639	361	8075	2361	0.213	0.043	0.361	0.639	0.774	0.762	0.320	0.504
LIMMA	407	593	7843	2593	0.136	0.070	0.593	0.407	0.752	0.721	0.204	0.499
MAST	486	514	7922	2514	0.162	0.061	0.514	0.486	0.759	0.735	0.243	0.499
Monocle	495	505	7931	2505	0.165	0.060	0.505	0.495	0.760	0.737	0.248	0.492
NBSeq	554	446	7990	2446	0.185	0.053	0.446	0.554	0.766	0.747	0.277	0.513
NODES	322	678	7758	2678	0.107	0.080	0.678	0.322	0.743	0.707	0.161	0.463
ROTS	533	467	7969	2467	0.178	0.055	0.467	0.533	0.764	0.743	0.267	0.496
scDD	368	632	7804	2632	0.123	0.075	0.632	0.368	0.748	0.715	0.184	0.469
T-test	585	415	8021	2415	0.195	0.049	0.415	0.585	0.769	0.753	0.293	0.509
Wilcox	644	356	8080	2356	0.215	0.042	0.356	0.644	0.774	0.763	0.322	0.548
						DEG = 1500						
BPSC	660	840	7596	2340	0.220	0.100	0.560	0.440	0.764	0.722	0.293	0.536

DECENT	998	502	7934	2002	0.333	0.060	0.335	0.665	0.799	0.781	0.444	0.590
DEGSeq	546	954	7482	2454	0.182	0.113	0.636	0.364	0.753	0.702	0.243	0.493
DESeqLRT	888	612	7824	2112	0.296	0.073	0.408	0.592	0.787	0.762	0.395	0.584
DESeqNB	1322	178	8258	1678	0.441	0.021	0.119	0.881	0.831	0.838	0.588	0.743
DEsingle	771	729	7707	2229	0.257	0.086	0.486	0.514	0.776	0.741	0.343	0.566
EBSeq	802	698	7738	2198	0.267	0.083	0.465	0.535	0.779	0.747	0.356	0.533
edgeRLRT	1088	412	8024	1912	0.363	0.049	0.275	0.725	0.808	0.797	0.484	0.604
edgeRQLF	1153	347	8089	1847	0.384	0.041	0.231	0.769	0.814	0.808	0.512	0.604
EMDomics	942	558	7878	2058	0.314	0.066	0.372	0.628	0.793	0.771	0.419	0.561
LIMMA	469	1031	7405	2531	0.156	0.122	0.687	0.313	0.745	0.689	0.208	0.519
MAST	669	831	7605	2331	0.223	0.099	0.554	0.446	0.765	0.724	0.297	0.534
Monocle	742	758	7678	2258	0.247	0.090	0.505	0.495	0.773	0.736	0.330	0.531
NBSeq	756	744	7692	2244	0.252	0.088	0.496	0.504	0.774	0.739	0.336	0.554
NODES	427	1073	7363	2573	0.142	0.127	0.715	0.285	0.741	0.681	0.190	0.486
ROTS	729	771	7665	2271	0.243	0.091	0.514	0.486	0.771	0.734	0.324	0.540
scDD	515	985	7451	2485	0.172	0.117	0.657	0.343	0.750	0.697	0.229	0.498
T-test	844	656	7780	2156	0.281	0.078	0.437	0.563	0.783	0.754	0.375	0.555
Wilcox	861	639	7797	2139	0.287	0.076	0.426	0.574	0.785	0.757	0.383	0.594
DEG = 2000												
BPSC	812	1188	7248	2188	0.271	0.141	0.594	0.406	0.768	0.705	0.325	0.568
DECENT	1255	745	7691	1745	0.418	0.088	0.373	0.628	0.815	0.782	0.502	0.650
DEGSeq	730	1270	7166	2270	0.243	0.151	0.635	0.365	0.759	0.690	0.292	0.522
DESeqLRT	1108	892	7544	1892	0.369	0.106	0.446	0.554	0.799	0.757	0.443	0.631
DESeqNB	1591	409	8027	1409	0.530	0.048	0.205	0.796	0.851	0.841	0.636	0.827
DEsingle	933	1067	7369	2067	0.311	0.126	0.534	0.467	0.781	0.726	0.373	0.604
EBSeq	1074	926	7510	1926	0.358	0.110	0.463	0.537	0.796	0.751	0.430	0.578
edgeRLRT	1399	601	7835	1601	0.466	0.071	0.301	0.700	0.830	0.807	0.560	0.670
edgeRQLF	1552	448	7988	1448	0.517	0.053	0.224	0.776	0.847	0.834	0.621	0.660
EMDomics	1264	736	7701	1736	0.421	0.087	0.368	0.632	0.816	0.784	0.506	0.611
LIMMA	549	1451	6985	2451	0.183	0.172	0.726	0.275	0.740	0.659	0.220	0.534
MAST	842	1158	7278	2158	0.281	0.137	0.579	0.421	0.771	0.710	0.337	0.567
Monocle	949	1051	7385	2051	0.316	0.125	0.526	0.475	0.783	0.729	0.380	0.574
NBSeq	924	1076	7360	2076	0.308	0.128	0.538	0.462	0.780	0.724	0.370	0.594
NODES	512	1488	6948	2488	0.171	0.176	0.744	0.256	0.736	0.652	0.205	0.506
ROTS	928	1072	7364	2072	0.309	0.127	0.536	0.464	0.780	0.725	0.371	0.578
scDD	639	1361	7075	2361	0.213	0.161	0.681	0.320	0.750	0.675	0.256	0.524
T-test	1107	893	7543	1893	0.369	0.106	0.447	0.554	0.799	0.756	0.443	0.601
Wilcox	1060	940	7496	1940	0.353	0.111	0.470	0.530	0.794	0.748	0.424	0.635
DEG = 2500												
BPSC	972	1528	6908	2028	0.324	0.181	0.611	0.389	0.773	0.689	0.353	0.596
DECENT	1500	1000	7436	1500	0.500	0.119	0.400	0.600	0.832	0.781	0.545	0.704
DEGSeq	924	1576	6860	2076	0.308	0.187	0.630	0.370	0.768	0.681	0.336	0.552
DESeqLRT	1320	1180	7256	1680	0.440	0.140	0.472	0.528	0.812	0.750	0.480	0.673

DESeqNB	1830	670	7766	1170	0.610	0.079	0.268	0.732	0.869	0.839	0.665	0.881
DEsingle	1077	1423	7013	1923	0.359	0.169	0.569	0.431	0.785	0.707	0.392	0.636
EBSeq	1369	1131	7305	1631	0.456	0.134	0.452	0.548	0.817	0.758	0.498	0.620
edgeRLRT	1704	796	7640	1296	0.568	0.094	0.318	0.682	0.855	0.817	0.620	0.732
edgeRQLF	1917	583	7853	1083	0.639	0.069	0.233	0.767	0.879	0.854	0.697	0.733
EMDomics	1548	952	7486	1452	0.516	0.113	0.381	0.619	0.838	0.790	0.563	0.672
LIMMA	633	1867	6569	2367	0.211	0.221	0.747	0.253	0.735	0.630	0.230	0.548
MAST	1000	1500	6936	2000	0.333	0.178	0.600	0.400	0.776	0.694	0.364	0.599
Monocle	1148	1352	7084	1852	0.383	0.160	0.541	0.459	0.793	0.720	0.417	0.613
NBSeq	1100	1400	7036	1900	0.367	0.166	0.560	0.440	0.787	0.711	0.400	0.627
NODES	596	1904	6532	2404	0.199	0.226	0.762	0.238	0.731	0.623	0.217	0.523
ROTS	1097	1403	7033	1903	0.366	0.166	0.561	0.439	0.787	0.711	0.399	0.616
scDD	744	1756	6680	2256	0.248	0.208	0.702	0.298	0.748	0.649	0.271	0.548
T-test	1348	1152	7284	1652	0.449	0.137	0.461	0.539	0.815	0.755	0.490	0.647
Wilcox	1231	1269	7167	1769	0.410	0.150	0.508	0.492	0.802	0.734	0.448	0.673
DEG = 3000												
BPSC	1154	1846	6590	1846	0.385	0.219	0.615	0.385	0.781	0.677	0.385	0.624
DECENT	1737	1267	7169	1267	0.580	0.150	0.417	0.578	0.850	0.778	0.578	0.774
DEGSeq	1098	1902	6534	1902	0.366	0.225	0.634	0.366	0.775	0.667	0.366	0.584
DESeqLRT	1501	1499	6937	1499	0.500	0.178	0.500	0.500	0.822	0.738	0.500	0.716
DESeqNB	2030	970	7466	970	0.677	0.115	0.323	0.677	0.885	0.830	0.677	0.932
DEsingle	1230	1770	6666	1770	0.410	0.210	0.590	0.410	0.790	0.690	0.410	0.665
EBSeq	1652	1348	7088	1348	0.551	0.160	0.449	0.551	0.840	0.764	0.551	0.666
edgeRLRT	1960	1040	7396	1040	0.653	0.123	0.347	0.653	0.877	0.818	0.653	0.797
edgeRQLF	2241	759	7677	759	0.747	0.090	0.253	0.747	0.910	0.867	0.747	0.817
EMDomics	1735	1264	7174	1264	0.577	0.150	0.421	0.579	0.850	0.779	0.579	0.739
LIMMA	749	2251	6185	2251	0.250	0.267	0.750	0.250	0.733	0.606	0.250	0.562
MAST	1162	1838	6598	1838	0.387	0.218	0.613	0.387	0.782	0.679	0.387	0.629
Monocle	1344	1656	6780	1656	0.448	0.196	0.552	0.448	0.804	0.710	0.448	0.650
NBSeq	1269	1731	6705	1731	0.423	0.205	0.577	0.423	0.795	0.697	0.423	0.659
NODES	684	2316	6120	2316	0.228	0.275	0.772	0.228	0.725	0.595	0.228	0.539
ROTS	1289	1711	6725	1711	0.430	0.203	0.570	0.430	0.797	0.701	0.430	0.649
scDD	846	2154	6282	2154	0.282	0.255	0.718	0.282	0.745	0.623	0.282	0.570
T-test	1584	1416	7020	1416	0.528	0.168	0.472	0.528	0.832	0.752	0.528	0.692
Wilcox	1391	1609	6827	1609	0.464	0.191	0.536	0.464	0.809	0.719	0.464	0.708

TP: True Positives; FP: False Positives; TN: True Negatives; FN: False Negatives; TPR: True Positive Rate; FPR: False Positive Rate; FDR: False Discovery Rate; PPR: Positive Prediction Rate; NPV: Negative Prediction Value; ACC: Accuracy; F1: F1 score; AUROC: Area Under Receiver Operating Curve

Table S10. Evaluation of DE methods based on performance evaluation metrics for Tung scRNA-seq data.

	TP	FP	TN	FN	TPR	FPR	FDR	PPR	NPV	ACC	F1	AUROC
					DEG = 500							
BPSC	190	310	12645	2810	0.063	0.024	0.620	0.380	0.818	0.804	0.109	0.440
DECENT	170	330	12625	2830	0.057	0.025	0.660	0.340	0.817	0.802	0.097	0.432
DEGSeq	81	419	12536	2919	0.027	0.032	0.838	0.162	0.811	0.791	0.046	0.414
DESeqLRT	204	296	12659	2796	0.068	0.023	0.592	0.408	0.819	0.806	0.117	0.446
DESeqNB	296	204	12751	2704	0.099	0.016	0.408	0.592	0.825	0.818	0.169	0.468
DEsingle	242	258	12697	2758	0.081	0.020	0.516	0.484	0.822	0.811	0.138	0.457
EBSeq	264	236	12719	2736	0.088	0.018	0.472	0.528	0.823	0.814	0.151	0.448
EdgeR	237	263	12692	2763	0.079	0.020	0.526	0.474	0.821	0.810	0.135	0.450
EdgeRQLF	330	170	12785	2670	0.110	0.013	0.340	0.660	0.827	0.822	0.189	0.461
LIMMA	244	256	12699	2756	0.081	0.020	0.512	0.488	0.822	0.811	0.139	0.455
MAST	297	203	12752	2703	0.099	0.016	0.406	0.594	0.825	0.818	0.170	0.469
Monocle	92	408	12547	2908	0.031	0.031	0.816	0.184	0.812	0.792	0.053	0.415
NBSeq	114	386	12569	2886	0.038	0.030	0.772	0.228	0.813	0.795	0.065	0.427
NODES	183	317	12638	2817	0.061	0.024	0.634	0.366	0.818	0.804	0.105	0.442
ROTS	111	389	12566	2889	0.037	0.030	0.778	0.222	0.813	0.795	0.063	0.418
scDD	96	404	12551	2904	0.032	0.031	0.808	0.192	0.812	0.793	0.055	0.421
T.test	149	351	12604	2851	0.050	0.027	0.702	0.298	0.816	0.799	0.085	0.429
Wilcox	253	247	12708	2747	0.084	0.019	0.494	0.506	0.822	0.812	0.145	0.455
EMDomics	287	213	12742	2713	0.096	0.016	0.426	0.574	0.824	0.817	0.164	0.460
					DEG = 1000							
BPSC	298	702	12253	2702	0.099	0.054	0.702	0.298	0.819	0.787	0.149	0.458
DECENT	365	635	12320	2635	0.122	0.049	0.635	0.365	0.824	0.795	0.183	0.458
DEGSeq	188	812	12143	2812	0.063	0.063	0.812	0.188	0.812	0.773	0.094	0.428
DESeqLRT	317	683	12272	2683	0.106	0.053	0.683	0.317	0.821	0.789	0.159	0.470
DESeqNB	394	606	12349	2606	0.131	0.047	0.606	0.394	0.826	0.799	0.197	0.502
DEsingle	403	597	12358	2597	0.134	0.046	0.597	0.403	0.826	0.800	0.202	0.487
EBSeq	528	472	12483	2472	0.176	0.036	0.472	0.528	0.835	0.815	0.264	0.490
EdgeR	428	572	12383	2572	0.143	0.044	0.572	0.428	0.828	0.803	0.214	0.484
EdgeRQLF	627	373	12582	2373	0.209	0.029	0.373	0.627	0.841	0.828	0.314	0.514
LIMMA	392	608	12347	2608	0.131	0.047	0.608	0.392	0.826	0.798	0.196	0.484
MAST	457	543	12412	2543	0.152	0.042	0.543	0.457	0.830	0.807	0.229	0.507
Monocle	212	788	12167	2788	0.071	0.061	0.788	0.212	0.814	0.776	0.106	0.433
NBSeq	215	785	12170	2785	0.072	0.061	0.785	0.215	0.814	0.776	0.108	0.441
NODES	271	729	12226	2729	0.090	0.056	0.729	0.271	0.818	0.783	0.136	0.460
ROTS	209	791	12164	2791	0.070	0.061	0.791	0.209	0.813	0.775	0.105	0.437
scDD	148	852	12103	2852	0.049	0.066	0.852	0.148	0.809	0.768	0.074	0.431
T.test	239	761	12194	2761	0.080	0.059	0.761	0.239	0.815	0.779	0.120	0.448
Wilcox	414	586	12369	2586	0.138	0.045	0.586	0.414	0.827	0.801	0.207	0.489
EMDomics	372	628	12327	2628	0.124	0.048	0.628	0.372	0.824	0.796	0.186	0.494
					DEG = 1500							

BPSC	398	1102	11853	2602	0.133	0.085	0.735	0.265	0.820	0.768	0.177	0.490
DECENT	535	965	11990	2465	0.178	0.074	0.643	0.357	0.829	0.785	0.238	0.490
DEGSeq	283	1217	11738	2717	0.094	0.094	0.811	0.189	0.812	0.753	0.126	0.444
DESeqLRT	428	1072	11883	2572	0.143	0.083	0.715	0.285	0.822	0.772	0.190	0.491
DESeqNB	472	1028	11927	2528	0.157	0.079	0.685	0.315	0.825	0.777	0.210	0.519
DEsingle	531	969	11986	2469	0.177	0.075	0.646	0.354	0.829	0.785	0.236	0.514
EBSeq	800	700	12255	2200	0.267	0.054	0.467	0.533	0.848	0.818	0.356	0.535
EdgeR	592	908	12047	2408	0.197	0.070	0.605	0.395	0.833	0.792	0.263	0.515
EdgeRQLF	881	619	12336	2119	0.294	0.048	0.413	0.587	0.853	0.828	0.392	0.570
LIMMA	487	1013	11942	2513	0.162	0.078	0.675	0.325	0.826	0.779	0.216	0.509
MAST	557	943	12012	2443	0.186	0.073	0.629	0.371	0.831	0.788	0.248	0.533
Monocle	316	1184	11771	2684	0.105	0.091	0.789	0.211	0.814	0.758	0.140	0.451
NBSeq	342	1158	11797	2658	0.114	0.089	0.772	0.228	0.816	0.761	0.152	0.457
NODES	364	1136	11819	2636	0.121	0.088	0.757	0.243	0.818	0.764	0.162	0.476
ROTS	285	1215	11740	2715	0.095	0.094	0.810	0.190	0.812	0.754	0.127	0.453
scDD	190	1310	11645	2810	0.063	0.101	0.873	0.127	0.806	0.742	0.084	0.440
T.test	340	1160	11795	2660	0.113	0.090	0.773	0.227	0.816	0.761	0.151	0.465
Wilcox	522	978	11977	2478	0.174	0.075	0.652	0.348	0.829	0.783	0.232	0.517
EMDomics	493	1007	11948	2507	0.164	0.078	0.671	0.329	0.827	0.780	0.219	0.512
DEG = 2000												
BPSC	498	1502	11453	2502	0.166	0.116	0.751	0.249	0.821	0.749	0.199	0.520
DECENT	701	1299	11656	2299	0.234	0.100	0.650	0.351	0.835	0.774	0.280	0.520
DEGSeq	373	1627	11328	2627	0.124	0.126	0.814	0.187	0.812	0.733	0.149	0.460
DESeqLRT	505	1495	11460	2495	0.168	0.115	0.748	0.253	0.821	0.750	0.202	0.509
DESeqNB	599	1401	11554	2401	0.200	0.108	0.701	0.300	0.828	0.762	0.240	0.535
DEsingle	633	1367	11588	2367	0.211	0.106	0.684	0.317	0.830	0.766	0.253	0.537
EBSeq	1021	979	11976	1979	0.340	0.076	0.490	0.511	0.858	0.815	0.408	0.584
EdgeR	748	1252	11703	2252	0.249	0.097	0.626	0.374	0.839	0.780	0.299	0.545
EdgeRQLF	1071	929	12026	1929	0.357	0.072	0.465	0.536	0.862	0.821	0.428	0.623
LIMMA	579	1421	11534	2421	0.193	0.110	0.711	0.290	0.827	0.759	0.232	0.529
MAST	651	1349	11606	2349	0.217	0.104	0.675	0.326	0.832	0.768	0.260	0.554
Monocle	409	1591	11364	2591	0.136	0.123	0.796	0.205	0.814	0.738	0.164	0.469
NBSeq	454	1546	11409	2546	0.151	0.119	0.773	0.227	0.818	0.744	0.182	0.475
NODES	440	1560	11395	2560	0.147	0.120	0.780	0.220	0.817	0.742	0.176	0.492
ROTS	367	1633	11322	2633	0.122	0.126	0.817	0.184	0.811	0.733	0.147	0.467
scDD	222	1778	11177	2778	0.074	0.137	0.889	0.111	0.801	0.714	0.089	0.447
T.test	438	1562	11393	2562	0.146	0.121	0.781	0.219	0.816	0.742	0.175	0.482
Wilcox	627	1373	11582	2373	0.209	0.106	0.687	0.314	0.830	0.765	0.251	0.538
EMDomics	598	1402	11553	2402	0.199	0.108	0.701	0.299	0.828	0.762	0.239	0.533
DEG = 2500												
BPSC	587	1913	11042	2413	0.196	0.148	0.765	0.235	0.821	0.729	0.213	0.550
DECENT	828	1672	11283	2172	0.276	0.129	0.669	0.331	0.839	0.759	0.301	0.550

DEGSeq	453	2047	10908	2547	0.151	0.158	0.819	0.181	0.811	0.712	0.165	0.477
DESeqLRT	563	1937	11018	2437	0.188	0.150	0.775	0.225	0.819	0.726	0.205	0.525
DESeqNB	675	1825	11130	2325	0.225	0.141	0.730	0.270	0.827	0.740	0.245	0.552
DEsingle	726	1774	11181	2274	0.242	0.137	0.710	0.290	0.831	0.746	0.264	0.558
EBSeq	1262	1238	11717	1738	0.421	0.096	0.495	0.505	0.871	0.813	0.459	0.624
EdgeR	894	1606	11349	2106	0.298	0.124	0.642	0.358	0.843	0.767	0.325	0.574
EdgeRQLF	1235	1265	11690	1765	0.412	0.098	0.506	0.494	0.869	0.810	0.449	0.666
LIMMA	653	1847	11108	2347	0.218	0.143	0.739	0.261	0.826	0.737	0.237	0.547
MAST	740	1760	11195	2260	0.247	0.136	0.704	0.296	0.832	0.748	0.269	0.573
Monocle	486	2014	10941	2514	0.162	0.155	0.806	0.194	0.813	0.716	0.177	0.486
NBSeq	558	1942	11013	2442	0.186	0.150	0.777	0.223	0.819	0.725	0.203	0.494
NODES	501	1999	10956	2499	0.167	0.154	0.800	0.200	0.814	0.718	0.182	0.506
ROTS	440	2060	10895	2560	0.147	0.159	0.824	0.176	0.810	0.710	0.160	0.481
scDD	271	2229	10726	2729	0.090	0.172	0.892	0.108	0.797	0.689	0.099	0.454
T.test	532	1968	10987	2468	0.177	0.152	0.787	0.213	0.817	0.722	0.193	0.499
Wilcox	727	1773	11182	2273	0.242	0.137	0.709	0.291	0.831	0.746	0.264	0.558
EMDomics	710	1790	11165	2290	0.237	0.138	0.716	0.284	0.830	0.744	0.258	0.551
DEG = 3000												
BPSC	673	2327	10628	2327	0.224	0.180	0.776	0.224	0.820	0.708	0.224	0.578
DECENT	929	2071	10884	2071	0.310	0.160	0.690	0.310	0.840	0.740	0.310	0.578
DEGSeq	517	2483	10472	2483	0.172	0.192	0.828	0.172	0.808	0.689	0.172	0.492
DESeqLRT	645	2355	10600	2355	0.215	0.182	0.785	0.215	0.818	0.705	0.215	0.539
DESeqNB	771	2229	10726	2229	0.257	0.172	0.743	0.257	0.828	0.721	0.257	0.569
DEsingle	821	2179	10776	2179	0.274	0.168	0.726	0.274	0.832	0.727	0.274	0.576
EBSeq	1508	1492	11463	1492	0.503	0.115	0.497	0.503	0.885	0.813	0.503	0.664
EdgeR	1019	1981	10974	1981	0.340	0.153	0.660	0.340	0.847	0.752	0.340	0.601
EdgeRQLF	1381	1619	11336	1619	0.460	0.125	0.540	0.460	0.875	0.797	0.460	0.703
LIMMA	740	2260	10695	2260	0.247	0.174	0.753	0.247	0.826	0.717	0.247	0.563
MAST	820	2180	10775	2180	0.273	0.168	0.727	0.273	0.832	0.727	0.273	0.590
Monocle	566	2434	10521	2434	0.189	0.188	0.811	0.189	0.812	0.695	0.189	0.501
NBSeq	664	2336	10619	2336	0.221	0.180	0.779	0.221	0.820	0.707	0.221	0.513
NODES	562	2438	10517	2438	0.187	0.188	0.813	0.187	0.812	0.694	0.187	0.519
ROTS	503	2497	10458	2497	0.168	0.193	0.832	0.168	0.807	0.687	0.168	0.494
scDD	330	2670	10285	2670	0.110	0.206	0.890	0.110	0.794	0.665	0.110	0.462
T.test	614	2386	10569	2386	0.205	0.184	0.795	0.205	0.816	0.701	0.205	0.515
Wilcox	814	2186	10769	2186	0.271	0.169	0.729	0.271	0.831	0.726	0.271	0.576
EMDomics	812	2188	10767	2188	0.271	0.169	0.729	0.271	0.831	0.726	0.271	0.569

TP: True Positives; FP: False Positives; TN: True Negatives; FN: False Negatives; TPR: True Positive Rate; FPR: False Positive Rate; FDR: False Discovery Rate; PPR: Positive Prediction Rate; NPV: Negative Prediction Value; ACC: Accuracy; F1: F1 score; AUROC: Area Under Receiver Operating Curve

Table S11. Evaluation of DE methods based on performance evaluation metrics for Ziegenhain scRNA-seq data.

	TP	FP	TN	FN	TPR	FPR	FDR	PPR	NPV	ACC	F1	AUROC
**Methods**							DEG = 500					
BPSC	252	248	12331	2748	0.084	0.020	0.496	0.504	0.818	0.808	0.144	0.459
DECENT	168	332	12247	2832	0.056	0.026	0.664	0.336	0.812	0.797	0.096	0.430
DEGSeq	105	395	12184	2895	0.035	0.031	0.790	0.210	0.808	0.789	0.060	0.420
DESeqLRT	181	319	12260	2819	0.060	0.025	0.638	0.362	0.813	0.799	0.103	0.430
DESeqNB	214	286	12293	2786	0.071	0.023	0.572	0.428	0.815	0.803	0.122	0.439
DEsingle	249	251	12328	2751	0.083	0.020	0.502	0.498	0.818	0.807	0.142	0.427
EBSeq	135	365	12214	2865	0.045	0.029	0.730	0.270	0.810	0.793	0.077	0.424
edgeRLRT	164	336	12243	2836	0.055	0.027	0.672	0.328	0.812	0.796	0.094	0.429
edgeRQLF	231	269	12310	2769	0.077	0.021	0.538	0.462	0.816	0.805	0.132	0.440
EMDomics	155	345	12234	2845	0.052	0.027	0.690	0.310	0.811	0.795	0.089	0.423
LIMMA	142	358	12221	2858	0.047	0.028	0.716	0.284	0.810	0.794	0.081	0.433
MAST	117	383	12196	2883	0.039	0.030	0.766	0.234	0.809	0.790	0.067	0.419
Monocle	228	272	12307	2772	0.076	0.022	0.544	0.456	0.816	0.805	0.130	0.441
NBSeq	175	325	12254	2825	0.058	0.026	0.650	0.350	0.813	0.798	0.100	0.430
NODES	211	289	12290	2789	0.070	0.023	0.578	0.422	0.815	0.802	0.121	0.433
ROTS	96	404	12175	2904	0.032	0.032	0.808	0.192	0.807	0.788	0.055	0.418
scDD	168	332	12247	2832	0.056	0.026	0.664	0.336	0.812	0.797	0.096	0.425
T-test	217	283	12296	2783	0.072	0.022	0.566	0.434	0.815	0.803	0.124	0.438
Wilcox	284	216	12363	2716	0.095	0.017	0.432	0.568	0.820	0.812	0.162	0.441
							NDEG = 1000					
BPSC	364	636	11943	2636	0.121	0.051	0.636	0.364	0.819	0.790	0.182	0.485
DECENT	286	714	11865	2714	0.095	0.057	0.714	0.286	0.814	0.780	0.143	0.455
DEGSeq	219	781	11798	2781	0.073	0.062	0.781	0.219	0.809	0.771	0.110	0.436
DESeqLRT	373	627	11952	2627	0.124	0.050	0.627	0.373	0.820	0.791	0.187	0.461
DESeqNB	465	535	12044	2535	0.155	0.043	0.535	0.465	0.826	0.803	0.233	0.473
DEsingle	467	533	12046	2533	0.156	0.042	0.533	0.467	0.826	0.803	0.234	0.476
EBSeq	254	746	11833	2746	0.085	0.059	0.746	0.254	0.812	0.776	0.127	0.445
edgeRLRT	336	664	11915	2664	0.112	0.053	0.664	0.336	0.817	0.786	0.168	0.456
edgeRQLF	346	654	11925	2654	0.115	0.052	0.654	0.346	0.818	0.788	0.173	0.473
EMDomics	336	664	11915	2664	0.112	0.053	0.664	0.336	0.817	0.786	0.168	0.449
LIMMA	229	771	11808	2771	0.076	0.061	0.771	0.229	0.810	0.773	0.115	0.449
MAST	231	769	11810	2769	0.077	0.061	0.769	0.231	0.810	0.773	0.116	0.438
Monocle	368	632	11947	2632	0.123	0.050	0.632	0.368	0.819	0.790	0.184	0.475
NBSeq	393	607	11972	2607	0.131	0.048	0.607	0.393	0.821	0.794	0.197	0.460
NODES	391	609	11970	2609	0.130	0.048	0.609	0.391	0.821	0.793	0.196	0.470
ROTS	189	811	11768	2811	0.063	0.064	0.811	0.189	0.807	0.768	0.095	0.432
scDD	268	732	11847	2732	0.089	0.058	0.732	0.268	0.813	0.778	0.134	0.452
T-test	430	570	12009	2570	0.143	0.045	0.570	0.430	0.824	0.798	0.215	0.474
Wilcox	543	457	12122	2457	0.181	0.036	0.457	0.543	0.831	0.813	0.272	0.492
							NDEG = 1500					

BPSC	466	1034	11545	2534	0.155	0.082	0.689	0.311	0.820	0.771	0.207	0.505
DECENT	431	1069	11510	2569	0.144	0.085	0.713	0.287	0.818	0.766	0.192	0.476
DEGSeq	361	1139	11440	2639	0.120	0.091	0.759	0.241	0.813	0.757	0.160	0.455
DESeqLRT	552	948	11631	2448	0.184	0.075	0.632	0.368	0.826	0.782	0.245	0.491
DESeqNB	702	798	11781	2298	0.234	0.063	0.532	0.468	0.837	0.801	0.312	0.514
DEsingle	687	813	11766	2313	0.229	0.065	0.542	0.458	0.836	0.799	0.305	0.517
EBSeq	412	1088	11491	2588	0.137	0.086	0.725	0.275	0.816	0.764	0.183	0.467
edgeRLRT	500	1000	11579	2500	0.167	0.079	0.667	0.333	0.822	0.775	0.222	0.484
edgeRQLF	473	1027	11552	2527	0.158	0.082	0.685	0.315	0.821	0.772	0.210	0.495
EMDomics	620	880	11699	2380	0.207	0.070	0.587	0.413	0.831	0.791	0.276	0.475
LIMMA	294	1206	11373	2706	0.098	0.096	0.804	0.196	0.808	0.749	0.131	0.463
MAST	343	1157	11422	2657	0.114	0.092	0.771	0.229	0.811	0.755	0.152	0.458
Monocle	559	941	11638	2441	0.186	0.075	0.627	0.373	0.827	0.783	0.248	0.503
NBSeq	567	933	11646	2433	0.189	0.074	0.622	0.378	0.827	0.784	0.252	0.496
NODES	519	981	11598	2481	0.173	0.078	0.654	0.346	0.824	0.778	0.231	0.502
ROTS	307	1193	11386	2693	0.102	0.095	0.795	0.205	0.809	0.751	0.136	0.448
scDD	354	1146	11433	2646	0.118	0.091	0.764	0.236	0.812	0.757	0.157	0.470
T-test	595	905	11674	2405	0.198	0.072	0.603	0.397	0.829	0.788	0.264	0.510
Wilcox	767	733	11846	2233	0.256	0.058	0.489	0.511	0.841	0.810	0.341	0.540
NDEG = 2000												
BPSC	583	1417	11162	2417	0.194	0.113	0.709	0.292	0.822	0.754	0.233	0.524
DECENT	591	1409	11170	2409	0.197	0.112	0.705	0.296	0.823	0.755	0.236	0.498
DEGSeq	470	1530	11049	2530	0.157	0.122	0.765	0.235	0.814	0.739	0.188	0.477
DESeqLRT	730	1270	11309	2270	0.243	0.101	0.635	0.365	0.833	0.773	0.292	0.522
DESeqNB	947	1053	11526	2053	0.316	0.084	0.527	0.474	0.849	0.801	0.379	0.553
DEsingle	870	1130	11449	2130	0.290	0.090	0.565	0.435	0.843	0.791	0.348	0.558
EBSeq	567	1433	11146	2433	0.189	0.114	0.717	0.284	0.821	0.752	0.227	0.490
edgeRLRT	683	1317	11262	2317	0.228	0.105	0.659	0.342	0.829	0.767	0.273	0.512
edgeRQLF	609	1391	11188	2391	0.203	0.111	0.696	0.305	0.824	0.757	0.244	0.518
EMDomics	926	1074	11505	2074	0.309	0.085	0.537	0.463	0.847	0.798	0.370	0.508
LIMMA	351	1649	10930	2649	0.117	0.131	0.825	0.176	0.805	0.724	0.140	0.475
MAST	478	1522	11057	2522	0.159	0.121	0.761	0.239	0.814	0.740	0.191	0.477
Monocle	735	1265	11314	2265	0.245	0.101	0.633	0.368	0.833	0.773	0.294	0.532
NBSeq	748	1252	11327	2252	0.249	0.100	0.626	0.374	0.834	0.775	0.299	0.527
NODES	774	1226	11353	2226	0.258	0.097	0.613	0.387	0.836	0.778	0.310	0.525
ROTS	413	1587	10992	2587	0.138	0.126	0.794	0.207	0.809	0.732	0.165	0.466
scDD	417	1583	10996	2583	0.139	0.126	0.792	0.209	0.810	0.733	0.167	0.487
T-test	771	1229	11350	2229	0.257	0.098	0.615	0.386	0.836	0.778	0.308	0.541
Wilcox	1010	990	11589	1990	0.337	0.079	0.495	0.505	0.853	0.809	0.404	0.581
NDEG = 2500												
BPSC	701	1799	10780	2299	0.234	0.143	0.720	0.280	0.824	0.737	0.255	0.544
DECENT	762	1738	10841	2238	0.254	0.138	0.695	0.305	0.829	0.745	0.277	0.522
DEGSeq	570	1930	10649	2430	0.190	0.153	0.772	0.228	0.814	0.720	0.207	0.497

DESeqLRT	890	1610	10969	2110	0.297	0.128	0.644	0.356	0.839	0.761	0.324	0.554
DESeqNB	1141	1359	11220	1859	0.380	0.108	0.544	0.456	0.858	0.793	0.415	0.598
DEsingle	1019	1481	11098	1981	0.340	0.118	0.592	0.408	0.849	0.778	0.371	0.595
EBSeq	729	1771	10808	2271	0.243	0.141	0.708	0.292	0.826	0.741	0.265	0.514
edgeRLRT	844	1656	10923	2156	0.281	0.132	0.662	0.338	0.835	0.755	0.307	0.541
edgeRQLF	765	1735	10844	2235	0.255	0.138	0.694	0.306	0.829	0.745	0.278	0.540
EMDomics	1053	1447	11132	1947	0.351	0.115	0.579	0.421	0.851	0.782	0.383	0.564
LIMMA	385	2115	10464	2615	0.128	0.168	0.846	0.154	0.800	0.696	0.140	0.486
MAST	600	1900	10679	2400	0.200	0.151	0.760	0.240	0.816	0.724	0.218	0.498
Monocle	889	1611	10968	2111	0.296	0.128	0.644	0.356	0.839	0.761	0.323	0.561
NBSeq	923	1577	11002	2077	0.308	0.125	0.631	0.369	0.841	0.765	0.336	0.559
NODES	988	1512	11067	2012	0.329	0.120	0.605	0.395	0.846	0.774	0.359	0.557
ROTS	510	1990	10589	2490	0.170	0.158	0.796	0.204	0.810	0.712	0.185	0.483
scDD	434	2066	10513	2566	0.145	0.164	0.826	0.174	0.804	0.703	0.158	0.500
T-test	934	1566	11013	2066	0.311	0.124	0.626	0.374	0.842	0.767	0.340	0.572
Wilcox	1219	1281	11298	1781	0.406	0.102	0.512	0.488	0.864	0.803	0.443	0.624
NDEG = 3000												
BPSC	813	2187	10392	2187	0.271	0.174	0.729	0.271	0.826	0.719	0.271	0.563
DECENT	969	2031	10548	2031	0.323	0.161	0.677	0.323	0.839	0.739	0.323	0.546
DEGSeq	675	2325	10254	2325	0.225	0.185	0.775	0.225	0.815	0.702	0.225	0.516
DESeqLRT	1036	1964	10615	1964	0.345	0.156	0.655	0.345	0.844	0.748	0.345	0.584
DESeqNB	1302	1698	10881	1698	0.434	0.135	0.566	0.434	0.865	0.782	0.434	0.639
DEsingle	1172	1828	10751	1828	0.391	0.145	0.609	0.391	0.855	0.765	0.391	0.628
EBSeq	887	2113	10466	2113	0.296	0.168	0.704	0.296	0.832	0.729	0.296	0.540
edgeRLRT	998	2002	10577	2002	0.333	0.159	0.667	0.333	0.841	0.743	0.333	0.570
edgeRQLF	946	2054	10525	2054	0.315	0.163	0.685	0.315	0.837	0.736	0.315	0.563
EMDomics	1160	1840	10739	1840	0.387	0.146	0.613	0.387	0.854	0.764	0.387	0.608
LIMMA	434	2566	10013	2566	0.145	0.204	0.855	0.145	0.796	0.671	0.145	0.494
MAST	730	2270	10309	2270	0.243	0.180	0.757	0.243	0.820	0.709	0.243	0.517
Monocle	1031	1969	10610	1969	0.344	0.157	0.656	0.344	0.843	0.747	0.344	0.590
NBSeq	1084	1916	10663	1916	0.361	0.152	0.639	0.361	0.848	0.754	0.361	0.590
NODES	1151	1849	10730	1849	0.384	0.147	0.616	0.384	0.853	0.763	0.384	0.593
ROTS	605	2395	10184	2395	0.202	0.190	0.798	0.202	0.810	0.693	0.202	0.501
scDD	485	2515	10064	2515	0.162	0.200	0.838	0.162	0.800	0.677	0.162	0.509
T-test	1102	1898	10681	1898	0.367	0.151	0.633	0.367	0.849	0.756	0.367	0.601
Wilcox	1415	1585	10994	1585	0.472	0.126	0.528	0.472	0.874	0.797	0.472	0.665

TP: True Positives; FP: False Positives; TN: True Negatives; FN: False Negatives; TPR: True Positive Rate; FPR: False Positive Rate; FDR: False Discovery Rate; PPR: Positive Prediction Rate; NPV: Negative Prediction Value; ACC: Accuracy; F1: F1 score; AUROC: Area Under Receiver Operating Curve

Table S12. Evaluation of DE methods based on performance evaluation metrics for Grun scRNA-seq data.

	TP	FP	TN	FN	TPR	FPR	FDR	PPR	NPV	ACC	F1	AUROC
**Methods**						DEG = 500						
BPSC	491	9	8561	2509	0.164	0.001	0.018	0.982	0.773	0.782	0.281	0.541
DECENT	445	55	8515	2555	0.148	0.006	0.110	0.890	0.769	0.774	0.254	0.496
DEGSeq	343	157	8419	2657	0.114	0.018	0.314	0.686	0.760	0.757	0.196	0.455
DESeqLRT	404	96	8474	2596	0.135	0.011	0.192	0.808	0.765	0.767	0.231	0.489
DESeqNB	424	76	8494	2576	0.141	0.009	0.152	0.848	0.767	0.771	0.242	0.495
DEsingle	412	88	8482	2588	0.137	0.010	0.176	0.824	0.766	0.769	0.235	0.499
EBSeq	412	88	8482	2588	0.137	0.010	0.176	0.824	0.766	0.769	0.235	0.471
edgeRQLF	476	24	8546	2524	0.159	0.003	0.048	0.952	0.772	0.780	0.272	0.528
edgeRLRT	457	43	8527	2543	0.152	0.005	0.086	0.914	0.770	0.776	0.261	0.508
EMDomics	9	491	8566	2991	0.003	0.054	0.982	0.018	0.741	0.711	0.005	0.401
LIMMA	381	119	8451	2619	0.127	0.014	0.238	0.762	0.763	0.763	0.218	0.471
MAST	445	55	8515	2555	0.148	0.006	0.110	0.890	0.769	0.774	0.254	0.499
Monocle	298	202	8368	2702	0.099	0.024	0.404	0.596	0.756	0.749	0.170	0.444
NBSeq	228	272	8298	2772	0.076	0.032	0.544	0.456	0.750	0.737	0.130	0.444
NODES	340	160	8410	2660	0.113	0.019	0.320	0.680	0.760	0.756	0.194	0.464
ROTS	366	134	8436	2634	0.122	0.016	0.268	0.732	0.762	0.761	0.209	0.460
scDD	299	201	8369	2701	0.100	0.023	0.402	0.598	0.756	0.749	0.171	0.456
T-test	351	149	8421	2649	0.117	0.017	0.298	0.702	0.761	0.758	0.201	0.464
Wilcox	410	90	8480	2590	0.137	0.011	0.180	0.820	0.766	0.768	0.234	0.486
						DEG = 1000						
BPSC	934	66	8504	2066	0.311	0.008	0.066	0.934	0.805	0.816	0.467	0.639
DECENT	843	157	8413	2157	0.281	0.018	0.157	0.843	0.796	0.800	0.422	0.572
DEGSeq	685	315	8261	2315	0.228	0.037	0.315	0.685	0.781	0.773	0.343	0.511
DESeqLRT	713	287	8283	2287	0.238	0.033	0.287	0.713	0.784	0.778	0.357	0.558
DESeqNB	781	219	8351	2219	0.260	0.026	0.219	0.781	0.790	0.789	0.391	0.563
DEsingle	711	289	8281	2289	0.237	0.034	0.289	0.711	0.783	0.777	0.356	0.561
EBSeq	749	251	8319	2251	0.250	0.029	0.251	0.749	0.787	0.784	0.375	0.554
edgeRQLF	908	92	8478	2092	0.303	0.011	0.092	0.908	0.802	0.811	0.454	0.613
edgeRLRT	846	154	8416	2154	0.282	0.018	0.154	0.846	0.796	0.801	0.423	0.592
EMDomics	13	987	8564	2987	0.004	0.103	0.987	0.013	0.741	0.683	0.007	0.403
LIMMA	724	276	8294	2276	0.241	0.032	0.276	0.724	0.785	0.779	0.362	0.540
MAST	847	153	8417	2153	0.282	0.018	0.153	0.847	0.796	0.801	0.424	0.573
Monocle	616	384	8186	2384	0.205	0.045	0.384	0.616	0.774	0.761	0.308	0.496
NBSeq	370	630	7940	2630	0.123	0.074	0.630	0.370	0.751	0.718	0.185	0.477
NODES	568	432	8138	2432	0.189	0.050	0.432	0.568	0.770	0.752	0.284	0.521
ROTS	731	269	8301	2269	0.244	0.031	0.269	0.731	0.785	0.781	0.366	0.520
scDD	525	475	8095	2475	0.175	0.055	0.475	0.525	0.766	0.745	0.263	0.506
T-test	693	307	8263	2307	0.231	0.036	0.307	0.693	0.782	0.774	0.347	0.522
Wilcox	781	219	8351	2219	0.260	0.026	0.219	0.781	0.790	0.789	0.391	0.546
						DEG = 1500						

BPSC	1311	189	8381	1689	0.437	0.022	0.126	0.874	0.832	0.838	0.583	0.729
DECENT	1215	285	8285	1785	0.405	0.033	0.190	0.810	0.823	0.821	0.540	0.650
DEGSeq	978	522	8054	2022	0.326	0.061	0.348	0.652	0.799	0.780	0.435	0.575
DESeqLRT	972	528	8042	2028	0.324	0.062	0.352	0.648	0.799	0.779	0.432	0.615
DESeqNB	1139	361	8209	1861	0.380	0.042	0.241	0.759	0.815	0.808	0.506	0.625
DEsingle	1023	477	8093	1977	0.341	0.056	0.318	0.682	0.804	0.788	0.455	0.612
EBSeq	1071	429	8141	1929	0.357	0.050	0.286	0.714	0.808	0.796	0.476	0.619
edgeRQLF	1305	195	8375	1695	0.435	0.023	0.130	0.870	0.832	0.837	0.580	0.696
edgeRLRT	1215	285	8285	1785	0.405	0.033	0.190	0.810	0.823	0.821	0.540	0.663
EMDomics	13	1487	8564	2987	0.004	0.148	0.991	0.009	0.741	0.657	0.006	0.403
LIMMA	1019	481	8089	1981	0.340	0.056	0.321	0.679	0.803	0.787	0.453	0.606
MAST	1241	259	8311	1759	0.414	0.030	0.173	0.827	0.825	0.826	0.552	0.647
Monocle	936	564	8006	2064	0.312	0.066	0.376	0.624	0.795	0.773	0.416	0.548
NBSeq	529	971	7599	2471	0.176	0.113	0.647	0.353	0.755	0.703	0.235	0.504
NODES	705	795	7775	2295	0.235	0.093	0.530	0.470	0.772	0.733	0.313	0.564
ROTS	1066	434	8136	1934	0.355	0.051	0.289	0.711	0.808	0.795	0.474	0.587
scDD	696	804	7766	2304	0.232	0.094	0.536	0.464	0.771	0.731	0.309	0.546
T-test	1037	463	8107	1963	0.346	0.054	0.309	0.691	0.805	0.790	0.461	0.578
Wilcox	1124	376	8194	1876	0.375	0.044	0.251	0.749	0.814	0.805	0.500	0.616
DEG = 2000												
BPSC	1677	323	8247	1323	0.559	0.038	0.162	0.839	0.862	0.858	0.671	0.802
DECENT	1585	415	8155	1415	0.528	0.048	0.208	0.793	0.852	0.842	0.634	0.718
DEGSeq	1319	681	7896	1681	0.440	0.079	0.341	0.660	0.824	0.796	0.528	0.624
DESeqLRT	1195	805	7765	1805	0.398	0.094	0.403	0.598	0.811	0.774	0.478	0.666
DESeqNB	1464	536	8034	1536	0.488	0.063	0.268	0.732	0.839	0.821	0.586	0.693
DEsingle	1304	696	7874	1696	0.435	0.081	0.348	0.652	0.823	0.793	0.522	0.667
EBSeq	1338	662	7908	1662	0.446	0.077	0.331	0.669	0.826	0.799	0.535	0.683
edgeRQLF	1661	339	8231	1339	0.554	0.040	0.170	0.831	0.860	0.855	0.664	0.781
edgeRLRT	1567	433	8137	1433	0.522	0.051	0.217	0.784	0.850	0.839	0.627	0.733
EMDomics	13	1987	8564	2987	0.004	0.188	0.994	0.007	0.741	0.633	0.005	0.403
LIMMA	1300	700	7870	1700	0.433	0.082	0.350	0.650	0.822	0.793	0.520	0.663
MAST	1596	404	8166	1404	0.532	0.047	0.202	0.798	0.853	0.844	0.638	0.733
Monocle	1255	745	7825	1745	0.418	0.087	0.373	0.628	0.818	0.785	0.502	0.600
NBSeq	675	1325	7245	2325	0.225	0.155	0.663	0.338	0.757	0.685	0.270	0.529
NODES	854	1146	7424	2146	0.285	0.134	0.573	0.427	0.776	0.715	0.342	0.593
ROTS	1403	597	7973	1597	0.468	0.070	0.299	0.702	0.833	0.810	0.561	0.650
scDD	843	1157	7413	2157	0.281	0.135	0.579	0.422	0.775	0.714	0.337	0.580
T-test	1366	634	7936	1634	0.455	0.074	0.317	0.683	0.829	0.804	0.546	0.638
Wilcox	1455	545	8025	1545	0.485	0.064	0.273	0.728	0.839	0.819	0.582	0.683
DEG = 2500												
BPSC	1979	521	8049	1021	0.660	0.061	0.208	0.792	0.887	0.867	0.720	0.881
DECENT	1884	616	7954	1116	0.628	0.072	0.246	0.754	0.877	0.850	0.685	0.806
DEGSeq	1657	843	7734	1343	0.552	0.098	0.337	0.663	0.852	0.811	0.603	0.677

DESeqLRT	1390	1110	7460	1610	0.463	0.130	0.444	0.556	0.822	0.765	0.505	0.712
DESeqNB	1762	738	7832	1238	0.587	0.086	0.295	0.705	0.864	0.829	0.641	0.761
DEsingle	1581	919	7651	1419	0.527	0.107	0.368	0.632	0.844	0.798	0.575	0.720
EBSeq	1588	912	7658	1412	0.529	0.106	0.365	0.635	0.844	0.799	0.577	0.739
edgeRQLF	1936	564	8006	1064	0.645	0.066	0.226	0.774	0.883	0.859	0.704	0.870
edgeRLRT	1861	639	7931	1139	0.620	0.075	0.256	0.744	0.874	0.846	0.677	0.814
EMDomics	38	2462	8560	2962	0.013	0.223	0.985	0.015	0.743	0.613	0.014	0.405
LIMMA	1537	963	7607	1463	0.512	0.112	0.385	0.615	0.839	0.790	0.559	0.720
MAST	1852	648	7922	1148	0.617	0.076	0.259	0.741	0.873	0.845	0.673	0.823
Monocle	1561	939	7631	1439	0.520	0.110	0.376	0.624	0.841	0.794	0.568	0.657
NBSeq	797	1703	6867	2203	0.266	0.199	0.681	0.319	0.757	0.662	0.290	0.555
NODES	975	1525	7045	2025	0.325	0.178	0.610	0.390	0.777	0.693	0.355	0.621
ROTS	1700	800	7770	1300	0.567	0.093	0.320	0.680	0.857	0.818	0.618	0.718
scDD	936	1564	7006	2064	0.312	0.182	0.626	0.374	0.772	0.686	0.340	0.610
T-test	1670	830	7740	1330	0.557	0.097	0.332	0.668	0.853	0.813	0.607	0.703
Wilcox	1741	759	7811	1259	0.580	0.089	0.304	0.696	0.861	0.826	0.633	0.755
DEG = 3000												
BPSC	2199	801	7769	801	0.733	0.093	0.267	0.733	0.907	0.862	0.733	0.958
DECENT	2118	882	7688	882	0.706	0.103	0.294	0.706	0.897	0.848	0.706	0.886
DEGSeq	1941	1059	7518	1059	0.647	0.123	0.353	0.647	0.877	0.817	0.647	0.745
DESeqLRT	1549	1451	7119	1451	0.516	0.169	0.484	0.516	0.831	0.749	0.516	0.753
DESeqNB	1991	1009	7561	1009	0.664	0.118	0.336	0.664	0.882	0.826	0.664	0.832
DEsingle	1787	1213	7357	1213	0.596	0.142	0.404	0.596	0.858	0.790	0.596	0.779
EBSeq	1787	1213	7357	1213	0.596	0.142	0.404	0.596	0.858	0.790	0.596	0.795
edgeRQLF	2171	829	7741	829	0.724	0.097	0.276	0.724	0.903	0.857	0.724	0.940
edgeRLRT	2083	917	7653	917	0.694	0.107	0.306	0.694	0.893	0.841	0.694	0.888
EMDomics	47	2953	8557	2953	0.016	0.257	0.984	0.016	0.743	0.593	0.016	0.406
LIMMA	1757	1243	7327	1243	0.586	0.145	0.414	0.586	0.855	0.785	0.586	0.772
MAST	2076	924	7646	924	0.692	0.108	0.308	0.692	0.892	0.840	0.692	0.893
Monocle	1816	1184	7386	1184	0.605	0.138	0.395	0.605	0.862	0.795	0.605	0.721
NBSeq	886	2114	6456	2114	0.295	0.247	0.705	0.295	0.753	0.635	0.295	0.579
NODES	1091	1909	6661	1909	0.364	0.223	0.636	0.364	0.777	0.670	0.364	0.646
ROTS	1949	1051	7519	1051	0.650	0.123	0.350	0.650	0.877	0.818	0.650	0.788
scDD	1022	1978	6592	1978	0.341	0.231	0.659	0.341	0.769	0.658	0.341	0.635
T-test	1935	1065	7505	1065	0.645	0.124	0.355	0.645	0.876	0.816	0.645	0.769
Wilcox	1967	1033	7537	1033	0.656	0.121	0.344	0.656	0.879	0.821	0.656	0.826

TP: True Positives; FP: False Positives; TN: True Negatives; FN: False Negatives; TPR: True Positive Rate; FPR: False Positive Rate; FDR: False Discovery Rate; PPR: Positive Prediction Rate; NPV: Negative Prediction Value; ACC: Accuracy; F1: F1 score; AUROC: Area Under Receiver Operating Curve

Table S13. Evaluation of DE methods based on performance evaluation metrics for Savas scRNA-seq data.

**Methods**	TP	FP	TN	FN	TPR	FPR	FDR	PPR	NPV	ACC	F1	AUROC

						DEG = 500						
BPSC	108	392	9361	2892	0.036	0.040	0.784	0.216	0.764	0.742	0.062	0.420
DECENT	224	276	9477	2776	0.074667	0.028	0.552	0.448	0.773	0.761	0.128	0.430
DEGSeq	115	385	9369	2885	0.038333	0.039	0.770	0.230	0.765	0.744	0.066	0.418
DESeqNB	90	410	9343	2910	0.03	0.042	0.820	0.180	0.763	0.740	0.051	0.415
DESeqLRT	97	403	9350	2903	0.032333	0.041	0.806	0.194	0.763	0.741	0.055	0.424
DEsingle	132	368	9385	2868	0.044	0.038	0.736	0.264	0.766	0.746	0.075	0.422
EBSeq	444	56	9697	2556	0.148	0.006	0.112	0.888	0.791	0.795	0.254	0.483
edgeRLRT	180	320	9433	2820	0.06	0.033	0.640	0.360	0.770	0.754	0.103	0.433
edgeRQLF	279	221	9532	2721	0.093	0.023	0.442	0.558	0.778	0.769	0.159	0.462
LIMMA	175	325	9428	2825	0.058333	0.033	0.650	0.350	0.769	0.753	0.100	0.430
MAST	117	383	9370	2883	0.039	0.039	0.766	0.234	0.765	0.744	0.067	0.423
Monocle	250	250	9503	2750	0.083333	0.026	0.500	0.500	0.776	0.765	0.143	0.433
NBSeq	243	257	9496	2757	0.081	0.026	0.514	0.486	0.775	0.764	0.139	0.437
NODES	140	360	9393	2860	0.046667	0.037	0.720	0.280	0.767	0.748	0.080	0.429
ROTS	104	396	9357	2896	0.034667	0.041	0.792	0.208	0.764	0.742	0.059	0.418
scDD	30	470	9283	2970	0.01	0.048	0.940	0.060	0.758	0.730	0.017	0.405
T-test	191	309	9444	2809	0.063667	0.032	0.618	0.382	0.771	0.756	0.109	0.432
Wilcox	233	267	9487	2767	0.077667	0.027	0.534	0.466	0.774	0.762	0.133	0.441
							DEG = 1000					
BPSC	232	768	8985	2768	0.077333	0.079	0.768	0.232	0.764	0.723	0.116	0.437
DECENT	469	531	9222	2531	0.156333	0.054	0.531	0.469	0.785	0.760	0.235	0.471
DEGSeq	234	766	8995	2766	0.078	0.078	0.766	0.234	0.765	0.723	0.117	0.437
DESeqNB	188	812	8941	2812	0.062667	0.083	0.812	0.188	0.761	0.716	0.094	0.430
DESeqLRT	137	863	8890	2863	0.045667	0.088	0.863	0.137	0.756	0.708	0.069	0.432
DEsingle	286	714	9039	2714	0.095333	0.073	0.714	0.286	0.769	0.731	0.143	0.444
EBSeq	779	221	9532	2221	0.259667	0.023	0.221	0.779	0.811	0.809	0.390	0.585
edgeRLRT	358	642	9111	2642	0.119333	0.066	0.642	0.358	0.775	0.742	0.179	0.463
edgeRQLF	445	555	9198	2555	0.148333	0.057	0.555	0.445	0.783	0.756	0.223	0.498
LIMMA	340	660	9093	2660	0.113333	0.068	0.660	0.340	0.774	0.740	0.170	0.457
MAST	196	804	8949	2804	0.065333	0.082	0.804	0.196	0.761	0.717	0.098	0.438
Monocle	513	487	9266	2487	0.171	0.050	0.487	0.513	0.788	0.767	0.257	0.479
NBSeq	504	496	9257	2496	0.168	0.051	0.496	0.504	0.788	0.765	0.252	0.480
NODES	252	748	9005	2748	0.084	0.077	0.748	0.252	0.766	0.726	0.126	0.448
ROTS	211	789	8964	2789	0.070333	0.081	0.789	0.211	0.763	0.719	0.106	0.434
scDD	64	936	8817	2936	0.021333	0.096	0.936	0.064	0.750	0.696	0.032	0.410
T-test	372	628	9125	2628	0.124	0.064	0.628	0.372	0.776	0.745	0.186	0.464
Wilcox	435	565	9189	2565	0.145	0.058	0.565	0.435	0.782	0.755	0.218	0.480
							DEG = 1500					
BPSC	363	1137	8616	2637	0.121	0.117	0.758	0.242	0.766	0.704	0.161	0.457
DECENT	713	787	8966	2287	0.237667	0.081	0.525	0.475	0.797	0.759	0.317	0.512
DEGSeq	353	1147	8649	2647	0.117667	0.117	0.765	0.235	0.766	0.704	0.157	0.458

DESeqNB	291	1209	8544	2709	0.097	0.124	0.806	0.194	0.759	0.693	0.129	0.446
DESeqLRT	183	1317	8436	2817	0.061	0.135	0.878	0.122	0.750	0.676	0.081	0.439
DEsingle	431	1069	8684	2569	0.143667	0.110	0.713	0.287	0.772	0.715	0.192	0.469
EBSeq	1010	490	9263	1990	0.336667	0.050	0.327	0.673	0.823	0.806	0.449	0.649
edgeRLRT	531	969	8784	2469	0.177	0.099	0.646	0.354	0.781	0.730	0.236	0.492
edgeRQLF	600	900	8853	2400	0.2	0.092	0.600	0.400	0.787	0.741	0.267	0.527
LIMMA	529	971	8782	2471	0.176333	0.100	0.647	0.353	0.780	0.730	0.235	0.486
MAST	294	1206	8547	2706	0.098	0.124	0.804	0.196	0.760	0.693	0.131	0.453
Monocle	752	748	9005	2248	0.250667	0.077	0.499	0.501	0.800	0.765	0.334	0.526
NBSeq	737	763	8990	2263	0.245667	0.078	0.509	0.491	0.799	0.763	0.328	0.524
NODES	348	1152	8601	2652	0.116	0.118	0.768	0.232	0.764	0.702	0.155	0.467
ROTS	324	1176	8577	2676	0.108	0.121	0.784	0.216	0.762	0.698	0.144	0.452
scDD	107	1393	8360	2893	0.035667	0.143	0.929	0.071	0.743	0.664	0.048	0.416
T-test	562	938	8815	2438	0.187333	0.096	0.625	0.375	0.783	0.735	0.250	0.494
Wilcox	605	895	8859	2395	0.201667	0.092	0.597	0.403	0.787	0.742	0.269	0.515
								DEG = 2000				
BPSC	488	1512	8241	2512	0.162667	0.155	0.756	0.244	0.766	0.684	0.195	0.478
DECENT	945	1055	8698	2055	0.315	0.108	0.528	0.473	0.809	0.756	0.378	0.553
DEGSeq	463	1537	8324	2537	0.154333	0.156	0.769	0.232	0.766	0.683	0.185	0.478
DESeqNB	396	1604	8149	2604	0.132	0.164	0.802	0.198	0.758	0.670	0.158	0.462
DESeqLRT	303	1697	8056	2697	0.101	0.174	0.849	0.152	0.749	0.655	0.121	0.448
DEsingle	574	1426	8327	2426	0.191333	0.146	0.713	0.287	0.774	0.698	0.230	0.493
EBSeq	1177	823	8930	1823	0.392333	0.084	0.412	0.589	0.830	0.793	0.471	0.696
edgeRLRT	698	1302	8451	2302	0.232667	0.133	0.651	0.349	0.786	0.717	0.279	0.521
edgeRQLF	763	1237	8516	2237	0.254333	0.127	0.619	0.382	0.792	0.728	0.305	0.554
LIMMA	731	1269	8484	2269	0.243667	0.130	0.635	0.366	0.789	0.723	0.292	0.514
MAST	390	1610	8143	2610	0.13	0.165	0.805	0.195	0.757	0.669	0.156	0.468
Monocle	972	1028	8725	2028	0.324	0.105	0.514	0.486	0.811	0.760	0.389	0.570
NBSeq	941	1059	8694	2059	0.313667	0.109	0.530	0.471	0.809	0.756	0.376	0.568
NODES	440	1560	8193	2560	0.146667	0.160	0.780	0.220	0.762	0.677	0.176	0.483
ROTS	432	1568	8185	2568	0.144	0.161	0.784	0.216	0.761	0.676	0.173	0.470
scDD	145	1855	7898	2855	0.048333	0.190	0.928	0.073	0.734	0.631	0.058	0.423
T-test	727	1273	8480	2273	0.242333	0.131	0.637	0.364	0.789	0.722	0.291	0.526
Wilcox	743	1257	8497	2257	0.247667	0.129	0.629	0.372	0.790	0.724	0.297	0.547
								DEG = 2500				
BPSC	613	1887	7866	2387	0.204333	0.193	0.755	0.245	0.767	0.665	0.223	0.500
DECENT	1113	1387	8366	1887	0.371	0.142	0.555	0.445	0.816	0.743	0.405	0.599
DEGSeq	553	1947	7973	2447	0.184333	0.196	0.779	0.221	0.765	0.660	0.201	0.497
DESeqNB	504	1996	7757	2496	0.168	0.205	0.798	0.202	0.757	0.648	0.183	0.480
DESeqLRT	433	2067	7686	2567	0.144333	0.212	0.827	0.173	0.750	0.637	0.157	0.461
DEsingle	710	1790	7963	2290	0.236667	0.184	0.716	0.284	0.777	0.680	0.258	0.518
EBSeq	1289	1211	8542	1711	0.429667	0.124	0.484	0.516	0.833	0.771	0.469	0.734
edgeRLRT	875	1625	8128	2125	0.291667	0.167	0.650	0.350	0.793	0.706	0.318	0.549

edgeRQLF	914	1586	8167	2086	0.304667	0.163	0.634	0.366	0.797	0.712	0.332	0.581
LIMMA	912	1588	8165	2088	0.304	0.163	0.635	0.365	0.796	0.712	0.332	0.547
MAST	482	2018	7735	2518	0.160667	0.207	0.807	0.193	0.754	0.644	0.175	0.484
Monocle	1199	1301	8452	1801	0.399667	0.133	0.520	0.480	0.824	0.757	0.436	0.611
NBSeq	1150	1350	8403	1850	0.383333	0.138	0.540	0.460	0.820	0.749	0.418	0.607
NODES	538	1962	7791	2462	0.179333	0.201	0.785	0.215	0.760	0.653	0.196	0.499
ROTS	550	1950	7803	2450	0.183333	0.200	0.780	0.220	0.761	0.655	0.200	0.489
scDD	190	2310	7443	2810	0.063333	0.237	0.924	0.076	0.726	0.599	0.069	0.429
T-test	910	1590	8163	2090	0.303333	0.163	0.636	0.364	0.796	0.711	0.331	0.555
Wilcox	873	1627	8127	2127	0.291	0.167	0.651	0.349	0.793	0.706	0.317	0.575
							DEG = 3000					
BPSC	756	2244	7509	2244	0.252	0.230	0.748	0.252	0.770	0.648	0.252	0.520
DECENT	1227	1773	7980	1773	0.409	0.182	0.591	0.409	0.818	0.722	0.409	0.641
DEGSeq	665	2335	7656	2335	0.221667	0.234	0.778	0.222	0.766	0.641	0.222	0.515
DESeqNB	616	2384	7369	2384	0.205333	0.244	0.795	0.205	0.756	0.626	0.205	0.498
DESeqLRT	554	2446	7307	2446	0.184667	0.251	0.815	0.185	0.749	0.616	0.185	0.477
DEsingle	840	2160	7593	2160	0.28	0.221	0.720	0.280	0.779	0.661	0.280	0.542
EBSeq	1342	1658	8095	1658	0.447333	0.170	0.553	0.447	0.830	0.740	0.447	0.762
edgeRLRT	1031	1969	7784	1969	0.343667	0.202	0.656	0.344	0.798	0.691	0.344	0.578
edgeRQLF	1067	1933	7820	1933	0.355667	0.198	0.644	0.356	0.802	0.697	0.356	0.608
LIMMA	1100	1900	7853	1900	0.366667	0.195	0.633	0.367	0.805	0.702	0.367	0.578
MAST	605	2395	7358	2395	0.201667	0.246	0.798	0.202	0.754	0.624	0.202	0.499
Monocle	1414	1586	8167	1586	0.471333	0.163	0.529	0.471	0.837	0.751	0.471	0.652
NBSeq	1316	1684	8069	1684	0.438667	0.173	0.561	0.439	0.827	0.736	0.439	0.648
NODES	611	2389	7364	2389	0.203667	0.245	0.796	0.204	0.755	0.625	0.204	0.516
ROTS	653	2347	7406	2347	0.217667	0.241	0.782	0.218	0.759	0.632	0.218	0.507
scDD	225	2775	6978	2775	0.075	0.285	0.925	0.075	0.715	0.565	0.075	0.436
T-test	1090	1910	7843	1910	0.363333	0.196	0.637	0.363	0.804	0.700	0.363	0.585
Wilcox	1007	1993	7761	1993	0.335667	0.204	0.664	0.336	0.796	0.687	0.336	0.600

TP: True Positives; FP: False Positives; TN: True Negatives; FN: False Negatives; TPR: True Positive Rate; FPR: False Positive Rate; FDR: False Discovery Rate; PPR: Positive Prediction Rate; NPV: Negative Prediction Value; ACC: Accuracy; F1: F1 score; AUROC: Area Under Receiver Operating Curve

Table S14. Evaluation of DE methods based on performance evaluation metrics for Soumillon1 scRNA-seq data.

Methods	TP	FP	TN	FN	TPR	FPR	FDR	PPR	NPV	ACC	F1	AUROC
							DEG = 500					

BPSC	285	215	9074	2715	0.095	0.023	0.430	0.570	0.770	0.762	0.163	0.465
DECENT	460	40	9249	2540	0.153	0.004	0.080	0.920	0.785	0.790	0.263	0.521
DEGSeq	244	256	9033	2756	0.081	0.028	0.512	0.488	0.766	0.755	0.139	0.441
DESeqLRT	106	394	8895	2894	0.035	0.042	0.788	0.212	0.755	0.732	0.061	0.426
DESeqNB	380	120	9169	2620	0.127	0.013	0.240	0.760	0.778	0.777	0.217	0.499
DEsingle	312	188	9101	2688	0.104	0.020	0.376	0.624	0.772	0.766	0.178	0.470
EBSeq	419	81	9208	2581	0.140	0.009	0.162	0.838	0.781	0.783	0.239	0.492
edgeRQLF	439	61	9228	2561	0.146	0.007	0.122	0.878	0.783	0.787	0.251	0.504
edgeRLRT	319	181	9108	2681	0.106	0.019	0.362	0.638	0.773	0.767	0.182	0.472
EMDomics	281	219	9070	2719	0.094	0.024	0.438	0.562	0.769	0.761	0.161	0.471
LIMMA	238	262	9027	2762	0.079	0.028	0.524	0.476	0.766	0.754	0.136	0.451
MAST	368	132	9157	2632	0.123	0.014	0.264	0.736	0.777	0.775	0.210	0.479
Monocle	294	206	9083	2706	0.098	0.022	0.412	0.588	0.770	0.763	0.168	0.462
NBSeq	380	120	9169	2620	0.127	0.013	0.240	0.760	0.778	0.777	0.217	0.492
NODES	249	251	9038	2751	0.083	0.027	0.502	0.498	0.767	0.756	0.142	0.459
ROTS	194	306	8983	2806	0.065	0.033	0.612	0.388	0.762	0.747	0.111	0.438
scDD	133	367	8922	2867	0.044	0.040	0.734	0.266	0.757	0.737	0.076	0.424
T-test	310	190	9099	2690	0.103	0.020	0.380	0.620	0.772	0.766	0.177	0.465
Wilcox	300	200	9089	2700	0.100	0.022	0.400	0.600	0.771	0.764	0.171	0.467
DEG = 1000												
BPSC	483	517	8772	2517	0.161	0.056	0.517	0.483	0.777	0.753	0.242	0.502
DECENT	771	229	9060	2229	0.257	0.025	0.229	0.771	0.803	0.800	0.386	0.598
DEGSeq	492	508	8782	2508	0.164	0.055	0.508	0.492	0.778	0.755	0.246	0.483
DESeqLRT	159	841	8448	2841	0.053	0.091	0.841	0.159	0.748	0.700	0.080	0.436
DESeqNB	595	405	8884	2405	0.198	0.044	0.405	0.595	0.787	0.771	0.298	0.546
DEsingle	517	483	8806	2483	0.172	0.052	0.483	0.517	0.780	0.759	0.259	0.512
EBSeq	707	293	8996	2293	0.236	0.032	0.293	0.707	0.797	0.790	0.354	0.561
edgeRQLF	755	245	9044	2245	0.252	0.026	0.245	0.755	0.801	0.797	0.378	0.585
edgeRLRT	538	462	8827	2462	0.179	0.050	0.462	0.538	0.782	0.762	0.269	0.516
EMDomics	486	514	8775	2514	0.162	0.055	0.514	0.486	0.777	0.754	0.243	0.503
LIMMA	448	552	8737	2552	0.149	0.059	0.552	0.448	0.774	0.747	0.224	0.484
MAST	618	382	8907	2382	0.206	0.041	0.382	0.618	0.789	0.775	0.309	0.537
Monocle	537	463	8826	2463	0.179	0.050	0.463	0.537	0.782	0.762	0.269	0.506
NBSeq	636	364	8925	2364	0.212	0.039	0.364	0.636	0.791	0.778	0.318	0.546
NODES	383	617	8672	2617	0.128	0.066	0.617	0.383	0.768	0.737	0.192	0.485
ROTS	308	692	8597	2692	0.103	0.074	0.692	0.308	0.762	0.725	0.154	0.464
scDD	238	762	8527	2762	0.079	0.082	0.762	0.238	0.755	0.713	0.119	0.444
T-test	532	468	8821	2468	0.177	0.050	0.468	0.532	0.781	0.761	0.266	0.509
Wilcox	514	486	8803	2486	0.171	0.052	0.486	0.514	0.780	0.758	0.257	0.507
DEG = 1500												
BPSC	672	828	8461	2328	0.224	0.089	0.552	0.448	0.784	0.743	0.299	0.537
DECENT	969	531	8762	2031	0.323	0.057	0.354	0.646	0.812	0.792	0.431	0.653
DEGSeq	721	779	8512	2279	0.240	0.084	0.519	0.481	0.789	0.751	0.320	0.525

DESeqLRT	214	1286	8003	2786	0.071	0.138	0.857	0.143	0.742	0.669	0.095	0.445
DESeqNB	780	720	8569	2220	0.260	0.078	0.480	0.520	0.794	0.761	0.347	0.583
DEsingle	694	806	8483	2306	0.231	0.087	0.537	0.463	0.786	0.747	0.308	0.548
EBSeq	990	510	8779	2010	0.330	0.055	0.340	0.660	0.814	0.795	0.440	0.614
edgeRQLF	971	529	8760	2029	0.324	0.057	0.353	0.647	0.812	0.792	0.432	0.642
edgeRLRT	757	743	8546	2243	0.252	0.080	0.495	0.505	0.792	0.757	0.336	0.555
EMDomics	673	827	8462	2327	0.224	0.089	0.551	0.449	0.784	0.743	0.299	0.537
LIMMA	673	827	8462	2327	0.224	0.089	0.551	0.449	0.784	0.743	0.299	0.517
MAST	836	664	8625	2164	0.279	0.071	0.443	0.557	0.799	0.770	0.372	0.583
Monocle	764	736	8553	2236	0.255	0.079	0.491	0.509	0.793	0.758	0.340	0.547
NBSeq	857	643	8646	2143	0.286	0.069	0.429	0.571	0.801	0.773	0.381	0.591
NODES	559	941	8348	2441	0.186	0.101	0.627	0.373	0.774	0.725	0.248	0.510
ROTS	449	1051	8238	2551	0.150	0.113	0.701	0.299	0.764	0.707	0.200	0.485
scDD	365	1135	8154	2635	0.122	0.122	0.757	0.243	0.756	0.693	0.162	0.463
T-test	734	766	8523	2266	0.245	0.082	0.511	0.489	0.790	0.753	0.326	0.549
Wilcox	698	802	8487	2302	0.233	0.086	0.535	0.465	0.787	0.747	0.310	0.544
							DEG = 2000					
BPSC	847	1153	8136	2153	0.282	0.124	0.577	0.424	0.791	0.731	0.339	0.570
DECENT	1127	873	8423	1873	0.376	0.094	0.437	0.564	0.818	0.777	0.451	0.692
DEGSeq	907	1093	8202	2093	0.302	0.118	0.547	0.454	0.797	0.741	0.363	0.567
DESeqLRT	276	1724	7565	2724	0.092	0.186	0.862	0.138	0.735	0.638	0.110	0.454
DESeqNB	940	1060	8229	2060	0.313	0.114	0.530	0.470	0.800	0.746	0.376	0.617
DEsingle	853	1147	8142	2147	0.284	0.123	0.574	0.427	0.791	0.732	0.341	0.580
EBSeq	1261	739	8550	1739	0.420	0.080	0.370	0.631	0.831	0.798	0.504	0.664
edgeRQLF	1171	829	8460	1829	0.390	0.089	0.415	0.586	0.822	0.784	0.468	0.684
edgeRLRT	939	1061	8228	2061	0.313	0.114	0.531	0.470	0.800	0.746	0.376	0.593
EMDomics	847	1153	8136	2153	0.282	0.124	0.577	0.424	0.791	0.731	0.339	0.569
LIMMA	868	1132	8157	2132	0.289	0.122	0.566	0.434	0.793	0.734	0.347	0.555
MAST	1018	982	8307	1982	0.339	0.106	0.491	0.509	0.807	0.759	0.407	0.624
Monocle	970	1030	8259	2030	0.323	0.111	0.515	0.485	0.803	0.751	0.388	0.588
NBSeq	1058	942	8347	1942	0.353	0.101	0.471	0.529	0.811	0.765	0.423	0.632
NODES	743	1257	8032	2257	0.248	0.135	0.629	0.372	0.781	0.714	0.297	0.538
ROTS	590	1410	7879	2410	0.197	0.152	0.705	0.295	0.766	0.689	0.236	0.507
scDD	506	1494	7795	2494	0.169	0.161	0.747	0.253	0.758	0.675	0.202	0.482
T-test	889	1111	8178	2111	0.296	0.120	0.556	0.445	0.795	0.738	0.356	0.586
Wilcox	852	1148	8141	2148	0.284	0.124	0.574	0.426	0.791	0.732	0.341	0.579
							DEG = 2500					
BPSC	1035	1465	7824	1965	0.345	0.158	0.586	0.414	0.799	0.721	0.376	0.600
DECENT	1257	1243	8068	1743	0.419	0.133	0.497	0.503	0.822	0.757	0.457	0.723
DEGSeq	1093	1407	7903	1907	0.364	0.151	0.563	0.437	0.806	0.731	0.397	0.605
DESeqLRT	342	2158	7131	2658	0.114	0.232	0.863	0.137	0.728	0.608	0.124	0.464
DESeqNB	1093	1407	7882	1907	0.364	0.151	0.563	0.437	0.805	0.730	0.397	0.647
DEsingle	1006	1494	7795	1994	0.335	0.161	0.598	0.402	0.796	0.716	0.366	0.611

EBSeq	1489	1011	8278	1511	0.496	0.109	0.404	0.596	0.846	0.795	0.541	0.715
edgeRQLF	1356	1144	8145	1644	0.452	0.123	0.458	0.542	0.832	0.773	0.493	0.722
edgeRLRT	1117	1383	7906	1883	0.372	0.149	0.553	0.447	0.808	0.734	0.406	0.627
EMDomics	1034	1466	7823	1966	0.345	0.158	0.586	0.414	0.799	0.721	0.376	0.600
LIMMA	1065	1435	7854	1935	0.355	0.154	0.574	0.426	0.802	0.726	0.387	0.590
MAST	1183	1317	7972	1817	0.394	0.142	0.527	0.473	0.814	0.745	0.430	0.661
Monocle	1179	1321	7968	1821	0.393	0.142	0.528	0.472	0.814	0.744	0.429	0.625
NBSeq	1266	1234	8055	1734	0.422	0.133	0.494	0.506	0.823	0.758	0.460	0.668
NODES	927	1573	7716	2073	0.309	0.169	0.629	0.371	0.788	0.703	0.337	0.566
ROTS	729	1771	7518	2271	0.243	0.191	0.708	0.292	0.768	0.671	0.265	0.530
scDD	631	1869	7420	2369	0.210	0.201	0.748	0.252	0.758	0.655	0.229	0.503
T-test	1083	1417	7872	1917	0.361	0.153	0.567	0.433	0.804	0.729	0.394	0.618
Wilcox	1024	1476	7813	1976	0.341	0.159	0.590	0.410	0.798	0.719	0.372	0.609
DEG = 3000												
BPSC	1220	1780	7509	1780	0.407	0.192	0.593	0.407	0.808	0.710	0.407	0.631
DECENT	1415	1585	7744	1585	0.472	0.170	0.528	0.472	0.830	0.743	0.472	0.749
DEGSeq	1284	1716	7606	1716	0.428	0.184	0.572	0.428	0.816	0.721	0.428	0.639
DESeqLRT	425	2575	6714	2575	0.142	0.277	0.858	0.142	0.723	0.581	0.142	0.474
DESeqNB	1253	1747	7542	1747	0.418	0.188	0.582	0.418	0.812	0.716	0.418	0.676
DEsingle	1143	1857	7432	1857	0.381	0.200	0.619	0.381	0.800	0.698	0.381	0.640
EBSeq	1719	1281	8008	1281	0.573	0.138	0.427	0.573	0.862	0.792	0.573	0.762
edgeRQLF	1525	1475	7814	1475	0.508	0.159	0.492	0.508	0.841	0.760	0.508	0.758
edgeRLRT	1320	1680	7609	1680	0.440	0.181	0.560	0.440	0.819	0.727	0.440	0.659
EMDomics	1182	1818	7471	1818	0.394	0.196	0.606	0.394	0.804	0.704	0.394	0.633
LIMMA	1265	1735	7554	1735	0.422	0.187	0.578	0.422	0.813	0.718	0.422	0.624
MAST	1324	1676	7613	1676	0.441	0.180	0.559	0.441	0.820	0.727	0.441	0.695
Monocle	1362	1638	7651	1638	0.454	0.176	0.546	0.454	0.824	0.733	0.454	0.664
NBSeq	1442	1558	7731	1558	0.481	0.168	0.519	0.481	0.832	0.746	0.481	0.706
NODES	1106	1894	7395	1894	0.369	0.204	0.631	0.369	0.796	0.692	0.369	0.596
ROTS	863	2137	7152	2137	0.288	0.230	0.712	0.288	0.770	0.652	0.288	0.553
scDD	748	2252	7037	2252	0.249	0.242	0.751	0.249	0.758	0.633	0.249	0.525
T-test	1276	1724	7565	1724	0.425	0.186	0.575	0.425	0.814	0.719	0.425	0.649
Wilcox	1181	1819	7470	1819	0.394	0.196	0.606	0.394	0.804	0.704	0.394	0.639

TP: True Positives; FP: False Positives; TN: True Negatives; FN: False Negatives; TPR: True Positive Rate; FPR: False Positive Rate; FDR: False Discovery Rate; PPR: Positive Prediction Rate; NPV: Negative Prediction Value; ACC: Accuracy; F1: F1 score; AUROC: Area Under Receiver Operating Curve

Table S15. Evaluation of DE methods based on performance evaluation metrics for Soumillon3 scRNA-seq data.

	TP	FP	TN	FN	TPR	FPR	FDR	PPR	NPV	ACC	F1	AUROC
DEG = 500												

BPSC	226	274	11740	2773	0.075	0.023	0.548	0.452	0.809	0.797	0.129	0.436
DECENT	422	78	11936	2577	0.141	0.006	0.156	0.844	0.822	0.823	0.241	0.495
DEGSeq	184	316	11698	2815	0.061	0.026	0.632	0.368	0.806	0.791	0.105	0.434
DESeqLRT	160	340	11674	2839	0.053	0.028	0.680	0.320	0.804	0.788	0.091	0.430
DESeqNB	290	210	11804	2709	0.097	0.017	0.420	0.580	0.813	0.806	0.166	0.456
DEsingle	221	279	11735	2778	0.074	0.023	0.558	0.442	0.809	0.796	0.126	0.440
EBSeq	215	285	11729	2784	0.072	0.024	0.570	0.430	0.808	0.796	0.123	0.439
edgeRLRT	268	232	11782	2731	0.089	0.019	0.464	0.536	0.812	0.803	0.153	0.444
edgeRQLF	363	137	11877	2636	0.121	0.011	0.274	0.726	0.818	0.815	0.207	0.471
EMDomics	314	186	11828	2685	0.105	0.015	0.372	0.628	0.815	0.809	0.179	0.454
LIMMA	238	262	11752	2761	0.079	0.022	0.524	0.476	0.810	0.799	0.136	0.429
MAST	241	259	11755	2758	0.080	0.022	0.518	0.482	0.810	0.799	0.138	0.441
Monocle	202	298	11716	2797	0.067	0.025	0.596	0.404	0.807	0.794	0.115	0.431
NBSeq	254	246	11768	2745	0.085	0.020	0.492	0.508	0.811	0.801	0.145	0.445
NODES	184	316	11698	2815	0.061	0.026	0.632	0.368	0.806	0.791	0.105	0.430
ROTS	150	350	11664	2849	0.050	0.029	0.700	0.300	0.804	0.787	0.086	0.427
scDD	84	416	11598	2915	0.028	0.035	0.832	0.168	0.799	0.778	0.048	0.413
T-test	240	260	11754	2759	0.080	0.022	0.520	0.480	0.810	0.799	0.137	0.436
Wilcox	240	260	11754	2759	0.080	0.022	0.520	0.480	0.810	0.799	0.137	0.440
						DEG = 1000						
BPSC	436	564	11450	2563	0.145	0.047	0.564	0.436	0.817	0.792	0.218	0.475
DECENT	651	349	11665	2348	0.217	0.029	0.349	0.651	0.832	0.820	0.326	0.567
DEGSeq	352	648	11366	2647	0.117	0.054	0.648	0.352	0.811	0.781	0.176	0.462
DESeqLRT	306	694	11320	2693	0.102	0.058	0.694	0.306	0.808	0.774	0.153	0.455
DESeqNB	498	502	11512	2501	0.166	0.042	0.502	0.498	0.822	0.800	0.249	0.501
DEsingle	438	562	11452	2561	0.146	0.047	0.562	0.438	0.817	0.792	0.219	0.475
EBSeq	434	566	11448	2565	0.145	0.047	0.566	0.434	0.817	0.791	0.217	0.474
edgeRLRT	518	482	11532	2481	0.173	0.040	0.482	0.518	0.823	0.803	0.259	0.491
edgeRQLF	602	398	11616	2397	0.201	0.033	0.398	0.602	0.829	0.814	0.301	0.534
EMDomics	669	331	11683	2330	0.223	0.028	0.331	0.669	0.834	0.823	0.335	0.502
LIMMA	597	403	11611	2402	0.199	0.034	0.403	0.597	0.829	0.813	0.299	0.469
MAST	471	529	11485	2528	0.157	0.044	0.529	0.471	0.820	0.796	0.236	0.482
Monocle	428	572	11442	2571	0.143	0.048	0.572	0.428	0.817	0.791	0.214	0.466
NBSeq	488	512	11502	2511	0.163	0.043	0.512	0.488	0.821	0.799	0.244	0.486
NODES	366	634	11380	2633	0.122	0.053	0.634	0.366	0.812	0.782	0.183	0.462
ROTS	295	705	11309	2704	0.098	0.059	0.705	0.295	0.807	0.773	0.148	0.452
scDD	176	824	11190	2823	0.059	0.069	0.824	0.176	0.799	0.757	0.088	0.429
T-test	489	511	11503	2510	0.163	0.043	0.511	0.489	0.821	0.799	0.245	0.478
Wilcox	483	517	11497	2516	0.161	0.043	0.517	0.483	0.820	0.798	0.242	0.483
						DEG = 1500						
BPSC	634	866	11148	2365	0.211	0.072	0.577	0.423	0.825	0.785	0.282	0.511
DECENT	823	677	11337	2176	0.274	0.056	0.451	0.549	0.839	0.810	0.366	0.604
DEGSeq	527	973	11041	2472	0.176	0.081	0.649	0.351	0.817	0.771	0.234	0.491

DESeqLRT	433	1067	10947	2566	0.144	0.089	0.711	0.289	0.810	0.758	0.192	0.479
DESeqNB	683	817	11197	2316	0.228	0.068	0.545	0.455	0.829	0.791	0.304	0.537
DEsingle	631	869	11145	2368	0.210	0.072	0.579	0.421	0.825	0.784	0.281	0.512
EBSeq	658	842	11172	2341	0.219	0.070	0.561	0.439	0.827	0.788	0.293	0.511
edgeRLRT	726	774	11240	2273	0.242	0.064	0.516	0.484	0.832	0.797	0.323	0.536
edgeRQLF	768	732	11282	2231	0.256	0.061	0.488	0.512	0.835	0.803	0.341	0.577
EMDomics	864	636	11378	2135	0.288	0.053	0.424	0.576	0.842	0.815	0.384	0.575
LIMMA	876	624	11390	2123	0.292	0.052	0.416	0.584	0.843	0.817	0.389	0.536
MAST	655	845	11169	2344	0.218	0.070	0.563	0.437	0.827	0.788	0.291	0.521
Monocle	626	874	11140	2373	0.209	0.073	0.583	0.417	0.824	0.784	0.278	0.505
NBSeq	667	833	11181	2332	0.222	0.069	0.555	0.445	0.827	0.789	0.297	0.528
NODES	503	997	11017	2496	0.168	0.083	0.665	0.335	0.815	0.767	0.224	0.491
ROTS	445	1055	10959	2554	0.148	0.088	0.703	0.297	0.811	0.760	0.198	0.476
scDD	248	1252	10762	2751	0.083	0.104	0.835	0.165	0.796	0.733	0.110	0.443
T-test	712	788	11226	2287	0.237	0.066	0.525	0.475	0.831	0.795	0.317	0.521
Wilcox	679	821	11193	2320	0.226	0.068	0.547	0.453	0.828	0.791	0.302	0.524
						DEG = 2000						
BPSC	807	1193	10821	2192	0.269	0.099	0.597	0.404	0.832	0.775	0.323	0.547
DECENT	996	1004	11010	2003	0.332	0.084	0.502	0.498	0.846	0.800	0.398	0.636
DEGSeq	750	1250	10764	2249	0.250	0.104	0.625	0.375	0.827	0.767	0.300	0.517
DESeqLRT	542	1458	10556	2457	0.181	0.121	0.729	0.271	0.811	0.739	0.217	0.501
DESeqNB	846	1154	10860	2153	0.282	0.096	0.577	0.423	0.835	0.780	0.338	0.572
DEsingle	793	1207	10807	2206	0.264	0.100	0.604	0.397	0.830	0.773	0.317	0.548
EBSeq	866	1134	10880	2133	0.289	0.094	0.567	0.433	0.836	0.782	0.346	0.548
edgeRLRT	925	1075	10939	2074	0.308	0.089	0.538	0.463	0.841	0.790	0.370	0.574
edgeRQLF	946	1054	10960	2053	0.315	0.088	0.527	0.473	0.842	0.793	0.378	0.610
EMDomics	991	1009	11005	2008	0.330	0.084	0.505	0.496	0.846	0.799	0.396	0.624
LIMMA	1018	982	11032	1981	0.339	0.082	0.491	0.509	0.848	0.803	0.407	0.602
MAST	825	1175	10839	2174	0.275	0.098	0.588	0.413	0.833	0.777	0.330	0.556
Monocle	813	1187	10827	2186	0.271	0.099	0.594	0.407	0.832	0.775	0.325	0.540
NBSeq	850	1150	10864	2149	0.283	0.096	0.575	0.425	0.835	0.780	0.340	0.563
NODES	636	1364	10650	2363	0.212	0.114	0.682	0.318	0.818	0.752	0.254	0.517
ROTS	591	1409	10605	2408	0.197	0.117	0.705	0.296	0.815	0.746	0.236	0.501
scDD	318	1682	10332	2681	0.106	0.140	0.841	0.159	0.794	0.709	0.127	0.456
T-test	897	1103	10911	2102	0.299	0.092	0.552	0.449	0.838	0.787	0.359	0.564
Wilcox	849	1151	10863	2150	0.283	0.096	0.576	0.425	0.835	0.780	0.340	0.562
						DEG = 2500						
BPSC	986	1514	10500	2013	0.329	0.126	0.606	0.394	0.839	0.765	0.359	0.579
DECENT	1176	1324	10690	1823	0.392	0.110	0.530	0.470	0.854	0.790	0.428	0.666
DEGSeq	916	1584	10430	2083	0.305	0.132	0.634	0.366	0.834	0.756	0.333	0.551
DESeqLRT	640	1860	10154	2359	0.213	0.155	0.744	0.256	0.811	0.719	0.233	0.521
DESeqNB	1005	1495	10519	1994	0.335	0.124	0.598	0.402	0.841	0.768	0.366	0.603
DEsingle	936	1564	10450	2063	0.312	0.130	0.626	0.374	0.835	0.758	0.340	0.580

EBSeq	1061	1439	10575	1938	0.354	0.120	0.576	0.424	0.845	0.775	0.386	0.585
edgeRLRT	1089	1411	10603	1910	0.363	0.117	0.564	0.436	0.847	0.779	0.396	0.613
edgeRQLF	1095	1405	10609	1904	0.365	0.117	0.562	0.438	0.848	0.780	0.398	0.643
EMDomics	1120	1380	10634	1879	0.373	0.115	0.552	0.448	0.850	0.783	0.407	0.658
LIMMA	1117	1383	10631	1882	0.372	0.115	0.553	0.447	0.850	0.783	0.406	0.646
MAST	974	1526	10488	2025	0.325	0.127	0.610	0.390	0.838	0.763	0.354	0.590
Monocle	980	1520	10494	2019	0.327	0.127	0.608	0.392	0.839	0.764	0.356	0.575
NBSeq	1019	1481	10533	1980	0.340	0.123	0.592	0.408	0.842	0.769	0.371	0.597
NODES	796	1704	10310	2203	0.265	0.142	0.682	0.318	0.824	0.740	0.290	0.541
ROTS	783	1717	10297	2216	0.261	0.143	0.687	0.313	0.823	0.738	0.285	0.523
scDD	383	2117	9897	2616	0.128	0.176	0.847	0.153	0.791	0.685	0.139	0.469
T-test	1073	1427	10587	1926	0.358	0.119	0.571	0.429	0.846	0.777	0.390	0.602
Wilcox	1009	1491	10523	1990	0.336	0.124	0.596	0.404	0.841	0.768	0.367	0.596
						DEG = 3000						
BPSC	1152	1848	10166	1847	0.384	0.154	0.616	0.384	0.846	0.754	0.384	0.611
DECENT	1320	1680	10334	1679	0.440	0.140	0.560	0.440	0.860	0.776	0.440	0.699
DEGSeq	1055	1945	10069	1944	0.352	0.162	0.648	0.352	0.838	0.741	0.352	0.585
DESeqLRT	723	2277	9737	2276	0.241	0.190	0.759	0.241	0.811	0.697	0.241	0.541
DESeqNB	1155	1845	10169	1844	0.385	0.154	0.615	0.385	0.846	0.754	0.385	0.633
DEsingle	1065	1935	10079	1934	0.355	0.161	0.645	0.355	0.839	0.742	0.355	0.609
EBSeq	1231	1769	10245	1768	0.410	0.147	0.590	0.410	0.853	0.764	0.410	0.623
edgeRLRT	1241	1759	10255	1758	0.414	0.146	0.586	0.414	0.854	0.766	0.414	0.648
edgeRQLF	1239	1761	10253	1760	0.413	0.147	0.587	0.413	0.853	0.765	0.413	0.673
EMDomics	1254	1746	10268	1745	0.418	0.145	0.582	0.418	0.855	0.767	0.418	0.687
LIMMA	1218	1782	10232	1781	0.406	0.148	0.594	0.406	0.852	0.763	0.406	0.678
MAST	1114	1886	10128	1885	0.371	0.157	0.629	0.371	0.843	0.749	0.371	0.620
Monocle	1135	1865	10149	1864	0.378	0.155	0.622	0.378	0.845	0.752	0.378	0.608
NBSeq	1173	1827	10187	1826	0.391	0.152	0.609	0.391	0.848	0.757	0.391	0.629
NODES	931	2069	9945	2068	0.310	0.172	0.690	0.310	0.828	0.724	0.310	0.567
ROTS	952	2048	9966	2047	0.317	0.170	0.683	0.317	0.830	0.727	0.317	0.551
scDD	437	2563	9451	2562	0.146	0.213	0.854	0.146	0.787	0.659	0.146	0.481
T-test	1241	1759	10255	1758	0.414	0.146	0.586	0.414	0.854	0.766	0.414	0.637
Wilcox	1150	1850	10164	1849	0.383	0.154	0.617	0.383	0.846	0.754	0.383	0.628

TP: True Positives; FP: False Positives; TN: True Negatives; FN: False Negatives; TPR: True Positive Rate; FPR: False Positive Rate; FDR: False Discovery Rate; PPR: Positive Prediction Rate; NPV: Negative Prediction Value; ACC: Accuracy; F1: F1 score; AUROC: Area Under Receiver Operating Curve

Table S16. Evaluation of DE methods based on performance evaluation metrics for Klein scRNA-seq data.

**Methods**	**TP**	**FP**	**TN**	**FN**	**TPR**	**FPR**	**FDR**	**PPR**	**NPV**	**ACC**	**F1**	**AUROC**
						DEG = 500						

BPSC	136	364	20605	2864	0.045	0.017	0.728	0.272	0.878	0.865	0.078	0.426
DECENT	178	322	20647	2822	0.059	0.015	0.644	0.356	0.880	0.869	0.102	0.423
DEGSeq	191	309	20660	2809	0.064	0.015	0.618	0.382	0.880	0.870	0.109	0.433
DESeqLRT	155	345	20624	2845	0.052	0.016	0.690	0.310	0.879	0.867	0.089	0.434
DESeqNB	177	323	20646	2823	0.059	0.015	0.646	0.354	0.880	0.869	0.101	0.435
DEsingle	221	279	20690	2779	0.074	0.013	0.558	0.442	0.882	0.872	0.126	0.451
EBSeq	110	390	20579	2890	0.037	0.019	0.780	0.220	0.877	0.863	0.063	0.418
edgeRLRT	206	294	20675	2794	0.069	0.014	0.588	0.412	0.881	0.871	0.118	0.445
edgeRQLF	243	257	20712	2757	0.081	0.012	0.514	0.486	0.883	0.874	0.139	0.459
EMDomics	302	198	20771	2698	0.101	0.009	0.396	0.604	0.885	0.879	0.173	0.473
LIMMA	38	462	20509	2962	0.013	0.022	0.924	0.076	0.874	0.857	0.022	0.408
MAST	77	423	20546	2923	0.026	0.020	0.846	0.154	0.875	0.860	0.044	0.406
Monocle	134	366	20603	2866	0.045	0.017	0.732	0.268	0.878	0.865	0.077	0.413
NBSeq	133	367	20602	2867	0.044	0.018	0.734	0.266	0.878	0.865	0.076	0.414
NODES	59	441	20528	2941	0.020	0.021	0.882	0.118	0.875	0.859	0.034	0.410
ROTS	109	391	20578	2891	0.036	0.019	0.782	0.218	0.877	0.863	0.062	0.418
scDD	0	500	20469	3000	0.000	0.024	1.000	0.000	0.872	0.854	0.000	0.400
T-test	163	337	20632	2837	0.054	0.016	0.674	0.326	0.879	0.868	0.093	0.431
Wilcox	226	274	20695	2774	0.075	0.013	0.548	0.452	0.882	0.873	0.129	0.453
DEG = 1000												
BPSC	205	795	20174	2795	0.068	0.038	0.795	0.205	0.878	0.850	0.103	0.444
DECENT	326	674	20295	2674	0.109	0.032	0.674	0.326	0.884	0.860	0.163	0.456
DEGSeq	294	706	20263	2706	0.098	0.034	0.706	0.294	0.882	0.858	0.147	0.461
DESeqLRT	226	774	20195	2774	0.075	0.037	0.774	0.226	0.879	0.852	0.113	0.451
DESeqNB	276	724	20245	2724	0.092	0.035	0.724	0.276	0.881	0.856	0.138	0.458
DEsingle	306	694	20275	2694	0.102	0.033	0.694	0.306	0.883	0.859	0.153	0.474
EBSeq	234	766	20203	2766	0.078	0.037	0.766	0.234	0.880	0.853	0.117	0.435
edgeRLRT	287	713	20256	2713	0.096	0.034	0.713	0.287	0.882	0.857	0.144	0.468
edgeRQLF	321	679	20290	2679	0.107	0.032	0.679	0.321	0.883	0.860	0.161	0.481
EMDomics	453	547	20422	2547	0.151	0.026	0.547	0.453	0.889	0.871	0.227	0.517
LIMMA	85	915	20057	2915	0.028	0.044	0.915	0.085	0.873	0.840	0.043	0.413
MAST	255	745	20224	2745	0.085	0.036	0.745	0.255	0.880	0.854	0.128	0.427
Monocle	283	717	20252	2717	0.094	0.034	0.717	0.283	0.882	0.857	0.142	0.442
NBSeq	283	717	20252	2717	0.094	0.034	0.717	0.283	0.882	0.857	0.142	0.442
NODES	105	895	20074	2895	0.035	0.043	0.895	0.105	0.874	0.842	0.053	0.419
ROTS	214	786	20183	2786	0.071	0.037	0.786	0.214	0.879	0.851	0.107	0.433
scDD	0	1000	19969	3000	0.000	0.048	1.000	0.000	0.869	0.833	0.000	0.400
T-test	254	746	20223	2746	0.085	0.036	0.746	0.254	0.880	0.854	0.127	0.453
Wilcox	309	691	20278	2691	0.103	0.033	0.691	0.309	0.883	0.859	0.155	0.475
DEG = 1500												
BPSC	260	1240	19729	2740	0.087	0.059	0.827	0.173	0.878	0.834	0.116	0.456
DECENT	438	1062	19907	2562	0.146	0.051	0.708	0.292	0.886	0.849	0.195	0.483
DEGSeq	402	1098	19871	2598	0.134	0.052	0.732	0.268	0.884	0.846	0.179	0.481

DESeqLRT	263	1237	19732	2737	0.088	0.059	0.825	0.175	0.878	0.834	0.117	0.463
DESeqNB	344	1156	19813	2656	0.115	0.055	0.771	0.229	0.882	0.841	0.153	0.475
DEsingle	371	1129	19840	2629	0.124	0.054	0.753	0.247	0.883	0.843	0.165	0.489
EBSeq	317	1183	19786	2683	0.106	0.056	0.789	0.211	0.881	0.839	0.141	0.456
edgeRLRT	347	1153	19816	2653	0.116	0.055	0.769	0.231	0.882	0.841	0.154	0.483
edgeRQLF	379	1121	19848	2621	0.126	0.053	0.747	0.253	0.883	0.844	0.168	0.495
EMDomics	511	989	19980	2489	0.170	0.047	0.659	0.341	0.889	0.855	0.227	0.537
LIMMA	123	1377	19597	2877	0.041	0.066	0.918	0.082	0.872	0.823	0.055	0.419
MAST	431	1069	19900	2569	0.144	0.051	0.713	0.287	0.886	0.848	0.192	0.454
Monocle	381	1119	19850	2619	0.127	0.053	0.746	0.254	0.883	0.844	0.169	0.467
NBSeq	386	1114	19855	2614	0.129	0.053	0.743	0.257	0.884	0.844	0.172	0.467
NODES	146	1354	19615	2854	0.049	0.065	0.903	0.097	0.873	0.824	0.065	0.426
ROTS	320	1180	19789	2680	0.107	0.056	0.787	0.213	0.881	0.839	0.142	0.453
scDD	0	1500	19469	3000	0.000	0.072	1.000	0.000	0.866	0.812	0.000	0.400
T-test	312	1188	19781	2688	0.104	0.057	0.792	0.208	0.880	0.838	0.139	0.469
Wilcox	370	1130	19839	2630	0.123	0.054	0.753	0.247	0.883	0.843	0.164	0.490
						DEG = 2000						
BPSC	299	1701	19268	2701	0.100	0.081	0.851	0.150	0.877	0.816	0.120	0.466
DECENT	532	1468	19501	2468	0.177	0.070	0.734	0.266	0.888	0.836	0.213	0.505
DEGSeq	503	1497	19472	2497	0.168	0.071	0.749	0.252	0.886	0.833	0.201	0.500
DESeqLRT	294	1706	19263	2706	0.098	0.081	0.853	0.147	0.877	0.816	0.118	0.471
DESeqNB	424	1576	19393	2576	0.141	0.075	0.788	0.212	0.883	0.827	0.170	0.489
DEsingle	440	1560	19409	2560	0.147	0.074	0.780	0.220	0.883	0.828	0.176	0.502
EBSeq	390	1610	19359	2610	0.130	0.077	0.805	0.195	0.881	0.824	0.156	0.472
edgeRLRT	423	1577	19392	2577	0.141	0.075	0.789	0.212	0.883	0.827	0.169	0.495
edgeRQLF	443	1557	19412	2557	0.148	0.074	0.779	0.222	0.884	0.828	0.177	0.507
EMDomics	770	1230	19739	2230	0.257	0.059	0.615	0.385	0.898	0.856	0.308	0.549
LIMMA	200	1800	19174	2800	0.067	0.086	0.900	0.100	0.873	0.808	0.080	0.428
MAST	591	1409	19560	2409	0.197	0.067	0.705	0.296	0.890	0.841	0.236	0.482
Monocle	473	1527	19442	2527	0.158	0.073	0.764	0.237	0.885	0.831	0.189	0.487
NBSeq	493	1507	19462	2507	0.164	0.072	0.754	0.247	0.886	0.833	0.197	0.488
NODES	173	1827	19142	2827	0.058	0.087	0.914	0.087	0.871	0.806	0.069	0.434
ROTS	387	1613	19356	2613	0.129	0.077	0.807	0.194	0.881	0.824	0.155	0.471
scDD	0	2000	18969	3000	0.000	0.095	1.000	0.000	0.863	0.791	0.000	0.400
T-test	390	1610	19359	2610	0.130	0.077	0.805	0.195	0.881	0.824	0.156	0.481
Wilcox	435	1565	19404	2565	0.145	0.075	0.783	0.218	0.883	0.828	0.174	0.503
						DEG = 2500						
BPSC	327	2173	18796	2673	0.109	0.104	0.869	0.131	0.875	0.798	0.119	0.474
DECENT	617	1883	19086	2383	0.206	0.090	0.753	0.247	0.889	0.822	0.224	0.524
DEGSeq	589	1911	19058	2411	0.196	0.091	0.764	0.236	0.888	0.820	0.214	0.518
DESeqLRT	323	2177	18792	2677	0.108	0.104	0.871	0.129	0.875	0.797	0.117	0.478
DESeqNB	488	2012	18957	2512	0.163	0.096	0.805	0.195	0.883	0.811	0.177	0.503
DEsingle	496	2004	18965	2504	0.165	0.096	0.802	0.198	0.883	0.812	0.180	0.514

EBSeq	474	2026	18943	2526	0.158	0.097	0.810	0.190	0.882	0.810	0.172	0.487
edgeRLRT	488	2012	18957	2512	0.163	0.096	0.805	0.195	0.883	0.811	0.177	0.507
edgeRQLF	502	1998	18971	2498	0.167	0.095	0.799	0.201	0.884	0.812	0.183	0.518
EMDomics	1039	1461	19508	1961	0.346	0.070	0.584	0.416	0.909	0.857	0.378	0.574
LIMMA	354	2146	18828	2646	0.118	0.102	0.858	0.142	0.877	0.800	0.129	0.439
MAST	750	1750	19219	2250	0.250	0.083	0.700	0.300	0.895	0.833	0.273	0.510
Monocle	553	1947	19022	2447	0.184	0.093	0.779	0.221	0.886	0.817	0.201	0.505
NBSeq	589	1911	19058	2411	0.196	0.091	0.764	0.236	0.888	0.820	0.214	0.508
NODES	199	2301	18668	2801	0.066	0.110	0.920	0.080	0.870	0.787	0.072	0.439
ROTS	438	2062	18907	2562	0.146	0.098	0.825	0.175	0.881	0.807	0.159	0.485
scDD	0	2500	18469	3000	0.000	0.119	1.000	0.000	0.860	0.771	0.000	0.400
T-test	469	2031	18938	2531	0.156	0.097	0.812	0.188	0.882	0.810	0.171	0.494
Wilcox	499	2001	18968	2501	0.166	0.095	0.800	0.200	0.884	0.812	0.181	0.514
							DEG = 3000					
BPSC	365	2635	18334	2635	0.122	0.126	0.878	0.122	0.874	0.780	0.122	0.482
DECENT	704	2296	18673	2296	0.235	0.109	0.765	0.235	0.891	0.808	0.235	0.542
DEGSeq	719	2281	18688	2281	0.240	0.109	0.760	0.240	0.891	0.810	0.240	0.533
DESeqLRT	350	2650	18319	2650	0.117	0.126	0.883	0.117	0.874	0.779	0.117	0.484
DESeqNB	542	2458	18511	2458	0.181	0.117	0.819	0.181	0.883	0.795	0.181	0.516
DEsingle	552	2448	18521	2448	0.184	0.117	0.816	0.184	0.883	0.796	0.184	0.525
EBSeq	543	2457	18512	2457	0.181	0.117	0.819	0.181	0.883	0.795	0.181	0.501
edgeRLRT	545	2455	18514	2455	0.182	0.117	0.818	0.182	0.883	0.795	0.182	0.519
edgeRQLF	551	2449	18520	2449	0.184	0.117	0.816	0.184	0.883	0.796	0.184	0.529
EMDomics	1460	1540	19429	1540	0.487	0.073	0.513	0.487	0.927	0.872	0.487	0.586
LIMMA	492	2508	18466	2508	0.164	0.120	0.836	0.164	0.880	0.791	0.164	0.453
MAST	905	2095	18874	2095	0.302	0.100	0.698	0.302	0.900	0.825	0.302	0.537
Monocle	632	2368	18601	2368	0.211	0.113	0.789	0.211	0.887	0.802	0.211	0.521
NBSeq	689	2311	18658	2311	0.230	0.110	0.770	0.230	0.890	0.807	0.230	0.527
NODES	217	2783	18186	2783	0.072	0.133	0.928	0.072	0.867	0.768	0.072	0.445
ROTS	489	2511	18458	2511	0.163	0.120	0.837	0.163	0.880	0.790	0.163	0.497
scDD	0	3000	17969	3000	0.000	0.143	1.000	0.000	0.857	0.750	0.000	0.400
T-test	539	2461	18508	2461	0.180	0.117	0.820	0.180	0.883	0.795	0.180	0.507
Wilcox	546	2454	18515	2454	0.182	0.117	0.818	0.182	0.883	0.795	0.182	0.525

TP: True Positives; FP: False Positives; TN: True Negatives; FN: False Negatives; TPR: True Positive Rate; FPR: False Positive Rate; FDR: False Discovery Rate; PPR: Positive Prediction Rate; NPV: Negative Prediction Value; ACC: Accuracy; F1: F1 score; AUROC: Area Under Receiver Operating Curve

Table S17. Evaluation of DE methods based on performance evaluation metrics for Gierahn scRNA-seq data.

	TP	FP	TN	FN	TPR	FPR	FDR	PPR	NPV	ACC	F1	AUROC
							DEG = 500					

BPSC	1	498	12498	2999	0.001	0.038	0.998	0.002	0.806	0.781	0.001	0.400
DECENT	294	206	12790	2706	0.098	0.016	0.412	0.588	0.825	0.818	0.168	0.455
DEGSeq	191	308	12688	2809	0.064	0.024	0.617	0.383	0.819	0.805	0.109	0.426
DESeqLRT	45	455	12541	2955	0.015	0.035	0.910	0.090	0.809	0.787	0.026	0.411
DESeqNB	139	361	12635	2861	0.046	0.028	0.722	0.278	0.815	0.799	0.079	0.424
DESingle	2	492	12504	3000	0.000	0.038	1.000	0.000	0.807	0.782	0.000	0.400
EBSeq	468	32	12964	2532	0.156	0.002	0.064	0.936	0.837	0.840	0.267	0.494
edgeRLRT	5	495	12501	2995	0.002	0.038	0.990	0.010	0.807	0.782	0.003	0.401
edgeRQLF	5	495	12501	2995	0.002	0.038	0.990	0.010	0.807	0.782	0.003	0.401
EMDomics	140	356	12640	2860	0.047	0.027	0.718	0.282	0.815	0.799	0.080	0.424
LIMMA	226	274	12722	2774	0.075	0.021	0.548	0.452	0.821	0.809	0.129	0.434
MAST	13	487	12509	2987	0.004	0.037	0.974	0.026	0.807	0.783	0.007	0.403
Monocle	274	225	12771	2726	0.091	0.017	0.451	0.549	0.824	0.816	0.157	0.439
NBSeq	148	352	12644	2852	0.049	0.027	0.704	0.296	0.816	0.800	0.085	0.427
NODES	22	478	12518	2978	0.007	0.037	0.956	0.044	0.808	0.784	0.013	0.404
ROTS	4	496	12500	2996	0.001	0.038	0.992	0.008	0.807	0.782	0.002	0.401
scDD	29	471	12525	2971	0.010	0.036	0.942	0.058	0.808	0.785	0.017	0.404
T-test	4	496	12500	2996	0.001	0.038	0.992	0.008	0.807	0.782	0.002	0.401
Wilcox	4	496	12500	2996	0.001	0.038	0.992	0.008	0.807	0.782	0.002	0.401
DEG = 1000												
BPSC	1	998	11998	2999	0.000	0.077	0.999	0.001	0.800	0.750	0.001	0.400
DECENT	579	421	12575	2421	0.193	0.032	0.421	0.579	0.839	0.822	0.290	0.504
DEGSeq	421	578	12418	2579	0.140	0.044	0.579	0.421	0.828	0.803	0.211	0.461
DESeqLRT	55	945	12051	2945	0.018	0.073	0.945	0.055	0.804	0.757	0.028	0.414
DESeqNB	224	776	12220	2776	0.075	0.060	0.776	0.224	0.815	0.778	0.112	0.443
DESingle	3	992	12004	3000	0.000	0.076	1.000	0.000	0.800	0.750	0.000	0.400
EBSeq	864	136	12860	2136	0.288	0.010	0.136	0.864	0.858	0.858	0.432	0.595
edgeRLRT	5	995	12001	2995	0.002	0.077	0.995	0.005	0.800	0.751	0.003	0.401
edgeRQLF	6	994	12002	2994	0.002	0.076	0.994	0.006	0.800	0.751	0.003	0.402
EMDomics	224	771	12225	2776	0.075	0.059	0.775	0.225	0.815	0.778	0.112	0.443
LIMMA	435	565	12431	2565	0.145	0.043	0.565	0.435	0.829	0.804	0.218	0.473
MAST	16	984	12012	2984	0.005	0.076	0.984	0.016	0.801	0.752	0.008	0.404
Monocle	551	445	12551	2449	0.184	0.034	0.447	0.553	0.837	0.819	0.276	0.489
NBSeq	228	772	12224	2772	0.076	0.059	0.772	0.228	0.815	0.778	0.114	0.445
NODES	41	959	12037	2959	0.014	0.074	0.959	0.041	0.803	0.755	0.021	0.407
ROTS	4	996	12000	2996	0.001	0.077	0.996	0.004	0.800	0.750	0.002	0.401
scDD	52	948	12048	2948	0.017	0.073	0.948	0.052	0.803	0.756	0.026	0.409
T-test	5	995	12001	2995	0.002	0.077	0.995	0.005	0.800	0.751	0.003	0.401
Wilcox	4	996	12000	2996	0.001	0.077	0.996	0.004	0.800	0.750	0.002	0.401
DEG = 1500												
BPSC	1	1497	11499	2999	0.000	0.115	0.999	0.001	0.793	0.719	0.000	0.400
DECENT	846	654	12342	2154	0.282	0.050	0.436	0.564	0.851	0.824	0.376	0.550
DEGSeq	629	868	12128	2371	0.210	0.067	0.580	0.420	0.836	0.798	0.280	0.499

DESeqLRT	61	1439	11557	2939	0.020	0.111	0.959	0.041	0.797	0.726	0.027	0.416
DESeqNB	312	1188	11808	2688	0.104	0.091	0.792	0.208	0.815	0.758	0.139	0.459
DESingle	0	1492	11504	3000	0.000	0.115	1.000	0.000	0.793	0.719	0.000	0.400
EBSeq	1205	295	12701	1795	0.402	0.023	0.197	0.803	0.876	0.869	0.536	0.678
edgeRLRT	5	1495	11501	2995	0.002	0.115	0.997	0.003	0.793	0.719	0.002	0.402
edgeRQLF	6	1494	11502	2994	0.002	0.115	0.996	0.004	0.793	0.719	0.003	0.402
EMDomics	312	1183	11813	2688	0.104	0.091	0.791	0.209	0.815	0.758	0.139	0.459
LIMMA	678	822	12174	2322	0.226	0.063	0.548	0.452	0.840	0.803	0.301	0.508
MAST	25	1475	11521	2975	0.008	0.113	0.983	0.017	0.795	0.722	0.011	0.405
Monocle	776	716	12280	2224	0.259	0.055	0.480	0.520	0.847	0.816	0.346	0.539
NBSeq	312	1188	11808	2688	0.104	0.091	0.792	0.208	0.815	0.758	0.139	0.461
NODES	72	1428	11568	2928	0.024	0.110	0.952	0.048	0.798	0.728	0.032	0.411
ROTS	7	1493	11503	2993	0.002	0.115	0.995	0.005	0.794	0.720	0.003	0.402
scDD	82	1418	11578	2918	0.027	0.109	0.945	0.055	0.799	0.729	0.036	0.413
T-test	5	1495	11501	2995	0.002	0.115	0.997	0.003	0.793	0.719	0.002	0.402
Wilcox	4	1496	11500	2996	0.001	0.115	0.997	0.003	0.793	0.719	0.002	0.401
DEG = 2000												
BPSC	3	1993	11003	2997	0.001	0.153	0.998	0.002	0.786	0.688	0.001	0.400
DECENT	1069	931	12065	1931	0.356	0.072	0.466	0.535	0.862	0.821	0.428	0.598
DEGSeq	844	1152	11844	2156	0.281	0.089	0.577	0.423	0.846	0.793	0.338	0.536
DESeqLRT	189	1811	11185	2811	0.063	0.139	0.906	0.095	0.799	0.711	0.076	0.420
DESeqNB	406	1594	11402	2594	0.135	0.123	0.797	0.203	0.815	0.738	0.162	0.475
DESingle	0	1992	11004	3000	0.000	0.153	1.000	0.000	0.786	0.688	0.000	0.400
EBSeq	1469	531	12465	1531	0.490	0.041	0.266	0.735	0.891	0.871	0.588	0.755
edgeRLRT	5	1995	11001	2995	0.002	0.154	0.998	0.003	0.786	0.688	0.002	0.402
edgeRQLF	6	1994	11002	2994	0.002	0.153	0.997	0.003	0.786	0.688	0.002	0.402
EMDomics	406	1589	11407	2594	0.135	0.122	0.796	0.204	0.815	0.738	0.163	0.475
LIMMA	936	1064	11932	2064	0.312	0.082	0.532	0.468	0.853	0.804	0.374	0.545
MAST	34	1966	11030	2966	0.011	0.151	0.983	0.017	0.788	0.692	0.014	0.406
Monocle	989	1002	11994	2011	0.330	0.077	0.503	0.497	0.856	0.812	0.396	0.582
NBSeq	408	1592	11404	2592	0.136	0.122	0.796	0.204	0.815	0.738	0.163	0.476
NODES	106	1894	11102	2894	0.035	0.146	0.947	0.053	0.793	0.701	0.042	0.416
ROTS	7	1993	11003	2993	0.002	0.153	0.997	0.004	0.786	0.688	0.003	0.402
scDD	108	1892	11104	2892	0.036	0.146	0.946	0.054	0.793	0.701	0.043	0.418
T-test	5	1995	11001	2995	0.002	0.154	0.998	0.003	0.786	0.688	0.002	0.402
Wilcox	4	1996	11000	2996	0.001	0.154	0.998	0.002	0.786	0.688	0.002	0.401
DEG = 2500												
BPSC	5	2488	10508	2995	0.002	0.191	0.998	0.002	0.778	0.657	0.002	0.401
DECENT	1351	1149	11847	1649	0.450	0.088	0.460	0.540	0.878	0.825	0.491	0.638
DEGSeq	1064	1430	11566	1936	0.355	0.110	0.573	0.427	0.857	0.790	0.387	0.572
DESeqLRT	275	2225	10771	2725	0.092	0.171	0.890	0.110	0.798	0.691	0.100	0.430
DESeqNB	495	2005	10991	2505	0.165	0.154	0.802	0.198	0.814	0.718	0.180	0.490
DESingle	10	2482	10514	2990	0.003	0.191	0.996	0.004	0.779	0.658	0.004	0.400

EBSeq	1683	817	12179	1317	0.561	0.063	0.327	0.673	0.902	0.867	0.612	0.816
edgeRLRT	5	2495	10501	2995	0.002	0.192	0.998	0.002	0.778	0.657	0.002	0.402
edgeRQLF	6	2494	10502	2994	0.002	0.192	0.998	0.002	0.778	0.657	0.002	0.402
EMDomics	495	2000	10996	2505	0.165	0.154	0.802	0.198	0.814	0.718	0.180	0.491
LIMMA	1174	1326	11670	1826	0.391	0.102	0.530	0.470	0.865	0.803	0.427	0.586
MAST	47	2453	10543	2953	0.016	0.189	0.981	0.019	0.781	0.662	0.017	0.407
Monocle	1171	1319	11677	1829	0.390	0.101	0.530	0.470	0.865	0.803	0.427	0.623
NBSeq	487	2013	10983	2513	0.162	0.155	0.805	0.195	0.814	0.717	0.177	0.492
NODES	137	2363	10633	2863	0.046	0.182	0.945	0.055	0.788	0.673	0.050	0.421
ROTS	9	2491	10505	2991	0.003	0.192	0.996	0.004	0.778	0.657	0.003	0.402
scDD	134	2366	10630	2866	0.045	0.182	0.946	0.054	0.788	0.673	0.049	0.422
T-test	5	2495	10501	2995	0.002	0.192	0.998	0.002	0.778	0.657	0.002	0.402
Wilcox	5	2495	10501	2995	0.002	0.192	0.998	0.002	0.778	0.657	0.002	0.401
						DEG = 3000						
BPSC	5	2987	10009	2995	0.002	0.230	0.998	0.002	0.770	0.626	0.002	0.401
DECENT	1625	1375	11621	1375	0.542	0.106	0.458	0.542	0.894	0.828	0.542	0.680
DEGSeq	1275	1717	11279	1725	0.425	0.132	0.574	0.426	0.867	0.785	0.426	0.608
DESeqLRT	371	2629	10367	2629	0.124	0.202	0.876	0.124	0.798	0.671	0.124	0.442
DESeqNB	562	2438	10558	2438	0.187	0.188	0.813	0.187	0.812	0.695	0.187	0.506
DESingle	10	2982	10014	2990	0.003	0.229	0.997	0.003	0.770	0.627	0.003	0.401
EBSeq	1834	1166	11830	1166	0.611	0.090	0.389	0.611	0.910	0.854	0.611	0.867
edgeRLRT	5	2995	10001	2995	0.002	0.230	0.998	0.002	0.770	0.626	0.002	0.402
edgeRQLF	6	2994	10002	2994	0.002	0.230	0.998	0.002	0.770	0.626	0.002	0.402
EMDomics	563	2432	10564	2437	0.188	0.187	0.812	0.188	0.813	0.696	0.188	0.506
LIMMA	1395	1605	11391	1605	0.465	0.123	0.535	0.465	0.877	0.799	0.465	0.628
MAST	68	2932	10064	2932	0.023	0.226	0.977	0.023	0.774	0.633	0.023	0.409
Monocle	1328	1662	11334	1672	0.443	0.128	0.556	0.444	0.871	0.792	0.443	0.664
NBSeq	568	2432	10564	2432	0.189	0.187	0.811	0.189	0.813	0.696	0.189	0.506
NODES	182	2818	10178	2818	0.061	0.217	0.939	0.061	0.783	0.648	0.061	0.426
ROTS	11	2989	10007	2989	0.004	0.230	0.996	0.004	0.770	0.626	0.004	0.402
scDD	159	2841	10155	2841	0.053	0.219	0.947	0.053	0.781	0.645	0.053	0.427
T-test	7	2993	10003	2993	0.002	0.230	0.998	0.002	0.770	0.626	0.002	0.402
Wilcox	5	2995	10001	2995	0.002	0.230	0.998	0.002	0.770	0.626	0.002	0.401

TP: True Positives; FP: False Positives; TN: True Negatives; FN: False Negatives; TPR: True Positive Rate; FPR: False Positive Rate; FDR: False Discovery Rate; PPR: Positive Prediction Rate; NPV: Negative Prediction Value; ACC: Accuracy; F1: F1 score; AUROC: Area Under Receiver Operating Curve

Table S18. Evaluation of DE methods based on performance evaluation metrics for Chen data.

Methods	TP	FP	TN	FN	TPR	FPR	FDR	PPR	NPV	ACC	F1	AUROC
						DEG = 500						

BPSC	59	441	14965	2941	0.020	0.029	0.882	0.118	0.836	0.816	0.034	0.411
DECENT	146	354	15052	2854	0.049	0.023	0.708	0.292	0.841	0.826	0.083	0.424
DEGSeq	46	454	14952	2954	0.015	0.029	0.908	0.092	0.835	0.815	0.026	0.408
DESeqLRT	40	460	14946	2960	0.013	0.030	0.920	0.080	0.835	0.814	0.023	0.409
DESeqNB	75	425	14981	2925	0.025	0.028	0.850	0.150	0.837	0.818	0.043	0.413
DEsingle	78	422	14984	2922	0.026	0.027	0.844	0.156	0.837	0.818	0.045	0.413
EBSeq	207	293	15113	2793	0.069	0.019	0.586	0.414	0.844	0.832	0.118	0.446
edgeRLRT	103	397	15009	2897	0.034	0.026	0.794	0.206	0.838	0.821	0.059	0.417
edgeRQLF	133	367	15039	2867	0.044	0.024	0.734	0.266	0.840	0.824	0.076	0.421
LIMMMA	144	356	15050	2856	0.048	0.023	0.712	0.288	0.841	0.825	0.082	0.426
MAST	83	417	14989	2917	0.028	0.027	0.834	0.166	0.837	0.819	0.047	0.416
Monocle	94	406	15000	2906	0.031	0.026	0.812	0.188	0.838	0.820	0.054	0.418
NBSeq	96	404	15002	2904	0.032	0.026	0.808	0.192	0.838	0.820	0.055	0.420
NODES	68	432	14974	2932	0.023	0.028	0.864	0.136	0.836	0.817	0.039	0.412
ROTS	57	443	14963	2943	0.019	0.029	0.886	0.114	0.836	0.816	0.033	0.411
scDD	16	484	14922	2984	0.005	0.031	0.968	0.032	0.833	0.812	0.009	0.403
T-test	99	401	15005	2901	0.033	0.026	0.802	0.198	0.838	0.821	0.057	0.417
Wilcox	110	390	15016	2890	0.037	0.025	0.780	0.220	0.839	0.822	0.063	0.418
DEG = 1000												
BPSC	101	899	14507	2899	0.034	0.058	0.899	0.101	0.833	0.794	0.051	0.419
DECENT	242	758	14648	2758	0.081	0.049	0.758	0.242	0.842	0.809	0.121	0.447
DEGSeq	118	882	14524	2882	0.039	0.057	0.882	0.118	0.834	0.796	0.059	0.417
DESeqLRT	66	934	14472	2934	0.022	0.061	0.934	0.066	0.831	0.790	0.033	0.414
DESeqNB	142	858	14548	2858	0.047	0.056	0.858	0.142	0.836	0.798	0.071	0.424
DEsingle	152	848	14558	2848	0.051	0.055	0.848	0.152	0.836	0.799	0.076	0.427
EBSeq	382	618	14788	2618	0.127	0.040	0.618	0.382	0.850	0.824	0.191	0.473
edgeRLRT	185	815	14591	2815	0.062	0.053	0.815	0.185	0.838	0.803	0.093	0.433
edgeRQLF	229	771	14635	2771	0.076	0.050	0.771	0.229	0.841	0.808	0.115	0.442
LIMMMA	318	682	14724	2682	0.106	0.044	0.682	0.318	0.846	0.817	0.159	0.451
MAST	133	867	14539	2867	0.044	0.056	0.867	0.133	0.835	0.797	0.067	0.427
Monocle	186	814	14592	2814	0.062	0.053	0.814	0.186	0.838	0.803	0.093	0.433
NBSeq	158	842	14564	2842	0.053	0.055	0.842	0.158	0.837	0.800	0.079	0.433
NODES	143	857	14549	2857	0.048	0.056	0.857	0.143	0.836	0.798	0.072	0.423
ROTS	104	896	14510	2896	0.035	0.058	0.896	0.104	0.834	0.794	0.052	0.419
scDD	44	956	14450	2956	0.015	0.062	0.956	0.044	0.830	0.787	0.022	0.406
T-test	178	822	14584	2822	0.059	0.053	0.822	0.178	0.838	0.802	0.089	0.432
Wilcox	226	774	14632	2774	0.075	0.050	0.774	0.226	0.841	0.807	0.113	0.436
DEG = 1500												
BPSC	137	1363	14043	2863	0.046	0.088	0.909	0.091	0.831	0.770	0.061	0.426
DECENT	314	1186	14220	2686	0.105	0.077	0.791	0.209	0.841	0.790	0.140	0.463
DEGSeq	200	1300	14106	2800	0.067	0.084	0.867	0.133	0.834	0.777	0.089	0.430
DESeqLRT	86	1414	13992	2914	0.029	0.092	0.943	0.057	0.828	0.765	0.038	0.418
DESeqNB	222	1278	14128	2778	0.074	0.083	0.852	0.148	0.836	0.780	0.099	0.435

DEsingle	222	1278	14128	2778	0.074	0.083	0.852	0.148	0.836	0.780	0.099	0.439
EBSeq	562	938	14468	2438	0.187	0.061	0.625	0.375	0.856	0.817	0.250	0.500
edgeRLRT	273	1227	14179	2727	0.091	0.080	0.818	0.182	0.839	0.785	0.121	0.447
edgeRQLF	306	1194	14212	2694	0.102	0.078	0.796	0.204	0.841	0.789	0.136	0.459
LIMMMA	470	1030	14376	2530	0.157	0.067	0.687	0.313	0.850	0.807	0.209	0.478
MAST	162	1338	14068	2838	0.054	0.087	0.892	0.108	0.832	0.773	0.072	0.435
Monocle	278	1222	14184	2722	0.093	0.079	0.815	0.185	0.839	0.786	0.124	0.448
NBSeq	199	1301	14105	2801	0.066	0.084	0.867	0.133	0.834	0.777	0.088	0.442
NODES	188	1312	14094	2812	0.063	0.085	0.875	0.125	0.834	0.776	0.084	0.435
ROTS	145	1355	14051	2855	0.048	0.088	0.903	0.097	0.831	0.771	0.064	0.426
scDD	65	1435	13971	2935	0.022	0.093	0.957	0.043	0.826	0.763	0.029	0.410
T-test	271	1229	14177	2729	0.090	0.080	0.819	0.181	0.839	0.785	0.120	0.446
Wilcox	321	1179	14227	2679	0.107	0.077	0.786	0.214	0.842	0.790	0.143	0.455
DEG = 2000												
BPSC	174	1826	13580	2826	0.058	0.119	0.913	0.087	0.828	0.747	0.070	0.432
DECENT	392	1608	13798	2608	0.131	0.104	0.804	0.196	0.841	0.771	0.157	0.477
DEGSeq	250	1750	13656	2750	0.083	0.114	0.875	0.125	0.832	0.756	0.100	0.441
DESeqLRT	109	1891	13515	2891	0.036	0.123	0.946	0.055	0.824	0.740	0.044	0.421
DESeqNB	325	1675	13731	2675	0.108	0.109	0.838	0.163	0.837	0.764	0.130	0.449
DEsingle	289	1711	13695	2711	0.096	0.111	0.856	0.145	0.835	0.760	0.116	0.451
EBSeq	699	1301	14105	2301	0.233	0.084	0.651	0.350	0.860	0.804	0.280	0.529
edgeRLRT	347	1653	13753	2653	0.116	0.107	0.827	0.174	0.838	0.766	0.139	0.462
edgeRQLF	380	1620	13786	2620	0.127	0.105	0.810	0.190	0.840	0.770	0.152	0.474
LIMMMA	627	1373	14033	2373	0.209	0.089	0.687	0.314	0.855	0.796	0.251	0.504
MAST	179	1821	13585	2821	0.060	0.118	0.911	0.090	0.828	0.748	0.072	0.441
Monocle	365	1635	13771	2635	0.122	0.106	0.818	0.183	0.839	0.768	0.146	0.463
NBSeq	252	1748	13658	2748	0.084	0.113	0.874	0.126	0.833	0.756	0.101	0.451
NODES	212	1788	13618	2788	0.071	0.116	0.894	0.106	0.830	0.751	0.085	0.443
ROTS	174	1826	13580	2826	0.058	0.119	0.913	0.087	0.828	0.747	0.070	0.433
scDD	80	1920	13486	2920	0.027	0.125	0.960	0.040	0.822	0.737	0.032	0.414
T-test	340	1660	13746	2660	0.113	0.108	0.830	0.170	0.838	0.765	0.136	0.460
Wilcox	397	1603	13803	2603	0.132	0.104	0.802	0.199	0.841	0.771	0.159	0.473
DEG = 2500												
BPSC	225	2275	13131	2775	0.075	0.148	0.910	0.090	0.826	0.726	0.082	0.439
DECENT	454	2046	13360	2546	0.151	0.133	0.818	0.182	0.840	0.751	0.165	0.491
DEGSeq	299	2201	13205	2701	0.100	0.143	0.880	0.120	0.830	0.734	0.109	0.451
DESeqLRT	128	2372	13034	2872	0.043	0.154	0.949	0.051	0.819	0.715	0.047	0.425
DESeqNB	397	2103	13303	2603	0.132	0.137	0.841	0.159	0.836	0.744	0.144	0.463
DEsingle	322	2178	13228	2678	0.107	0.141	0.871	0.129	0.832	0.736	0.117	0.462
EBSeq	822	1678	13728	2178	0.274	0.109	0.671	0.329	0.863	0.791	0.299	0.556
edgeRLRT	422	2078	13328	2578	0.141	0.135	0.831	0.169	0.838	0.747	0.153	0.475
edgeRQLF	450	2050	13356	2550	0.150	0.133	0.820	0.180	0.840	0.750	0.164	0.488
LIMMMA	794	1706	13700	2206	0.265	0.111	0.682	0.318	0.861	0.787	0.289	0.530

MAST	207	2293	13113	2793	0.069	0.149	0.917	0.083	0.824	0.724	0.075	0.446
Monocle	453	2047	13359	2547	0.151	0.133	0.819	0.181	0.840	0.750	0.165	0.478
NBSeq	310	2190	13216	2690	0.103	0.142	0.876	0.124	0.831	0.735	0.113	0.459
NODES	243	2257	13149	2757	0.081	0.147	0.903	0.097	0.827	0.728	0.088	0.450
ROTS	212	2288	13118	2788	0.071	0.149	0.915	0.085	0.825	0.724	0.077	0.440
scDD	100	2400	13006	2900	0.033	0.156	0.960	0.040	0.818	0.712	0.036	0.417
T-test	416	2084	13322	2584	0.139	0.135	0.834	0.166	0.838	0.746	0.151	0.474
Wilcox	454	2046	13360	2546	0.151	0.133	0.818	0.182	0.840	0.751	0.165	0.488
						DEG = 3000						
BPSC	265	2735	12671	2735	0.088	0.178	0.912	0.088	0.822	0.703	0.088	0.446
DECENT	519	2481	12925	2481	0.173	0.161	0.827	0.173	0.839	0.730	0.173	0.503
DEGSeq	351	2649	12757	2649	0.117	0.172	0.883	0.117	0.828	0.712	0.117	0.461
DESeqLRT	149	2851	12555	2851	0.050	0.185	0.950	0.050	0.815	0.690	0.050	0.429
DESeqNB	462	2538	12868	2538	0.154	0.165	0.846	0.154	0.835	0.724	0.154	0.477
DEsingle	363	2637	12769	2637	0.121	0.171	0.879	0.121	0.829	0.713	0.121	0.471
EBSeq	946	2054	13352	2054	0.315	0.133	0.685	0.315	0.867	0.777	0.315	0.582
edgeRLRT	479	2521	12885	2521	0.160	0.164	0.840	0.160	0.836	0.726	0.160	0.489
edgeRQLF	508	2492	12914	2492	0.169	0.162	0.831	0.169	0.838	0.729	0.169	0.501
LIMMMA	966	2034	13372	2034	0.322	0.132	0.678	0.322	0.868	0.779	0.322	0.556
MAST	236	2764	12642	2764	0.079	0.179	0.921	0.079	0.821	0.700	0.079	0.451
Monocle	516	2484	12922	2484	0.172	0.161	0.828	0.172	0.839	0.730	0.172	0.492
NBSeq	361	2639	12767	2639	0.120	0.171	0.880	0.120	0.829	0.713	0.120	0.468
NODES	270	2730	12676	2730	0.090	0.177	0.910	0.090	0.823	0.703	0.090	0.456
ROTS	251	2749	12657	2749	0.084	0.178	0.916	0.084	0.822	0.701	0.084	0.446
scDD	119	2881	12525	2881	0.040	0.187	0.960	0.040	0.813	0.687	0.040	0.420
T-test	468	2532	12874	2532	0.156	0.164	0.844	0.156	0.836	0.725	0.156	0.487
Wilcox	517	2483	12923	2483	0.172	0.161	0.828	0.172	0.839	0.730	0.172	0.501

TP: True Positives; FP: False Positives; TN: True Negatives; FN: False Negatives; TPR: True Positive Rate; FPR: False Positive Rate; FDR: False Discovery Rate; PPR: Positive Prediction Rate; NPV: Negative Prediction Value; ACC: Accuracy; F1: F1 score; AUROC: Area Under Receiver Operating Curve

Table S19. Ranking of DE methods across all datasets based on the performance metrics.

						ACC						
**Methods**	D1	D2	D3	D4	D5	D6	D7	D8	D9	D10	D11	**Rank Score**
BPSC	15	11	14	1	12	16	12	9.5	8	13.5	16	4.84
DECENT	5	4	11	3	4	4	4	1	1	4	2	9.32

DEGSeq	16	17	16	9	8	3	13	15	14	12	5	4.84
DESeqLRT	8	13	8	15	19	17	18	18	2	17	9	4
DESeqNB	2	9	2	6	11	11.5	15	9.5	13	9	8	6.53
DEsingle	13	5.5	3	12.5	15	7.5	10	14	10	10.5	13	5.58
EBSeq	6	1	13	12.5	1	11.5	2	6	17	2	1	7.74
edgeRLRT	3	3	10	4	2	11.5	8	3.5	4	7	16	7.79
edgeRQLF	1	2	12	2	6.5	7.5	7	5	5	6	16	7.89
EMDomics	4	7.5	4	19	13	1	11	2	18	19	6.5	6.05
LIMMA	18	10	19	14	10	14	5	7	3	1	3	6.11
MAST	14	5.5	15	5	6.5	2	17	13	12	16	12	5.37
Monocle	10	15	9	11	5	6	1	12	9	4	4	7.05
NBSeq	12	12	7	18	3	5	3	8	6.5	10.5	6.5	6.76
NODES	19	16	5	16	16	18	16	17	15.5	13.5	10	3.05
ROTS	11	18	17	8	17	15	14	16	15.5	15	16	3.03
scDD	17	19	18	17	18	19	19	19	19	18	11	1.37
T-test	7	14	6	10	9	11.5	6	3.5	6.5	8	16	6.45
Wilcox	9	7.5	1	7	14	9	9	11	11	4	19	6.24

TPR

**Methods**	D1	D2	D3	D4	D5	D6	D7	D8	D9	D10	D11	Rank Score
BPSC	15	11	14	1	12	16	12	10	8	15	16.5	4.71
DECENT	5	4	11	3	4	4	4	1	1	3	2	9.37
DEGSeq	16	17	16	9	8	3	13	15	14	13	5	4.79
DESeqLRT	8	13	8	15	19	17	18	18	13	18	9	3.37
DESeqNB	2	9	2	6	11	11.5	15	9	2	9	8	7.13
DEsingle	13	5	3	12.5	15	7.5	11	14	10	11	14	5.47
EBSeq	6	1	13	12.5	1	11.5	2	6	17	2	1	7.74
edgeRLRT	3	3	10	4	7	9.5	8	3.5	4	7	16.5	7.61
edgeRQLF	1	2	12	2	2	7.5	7	5	5	6	16.5	8.11
EMDomics	4	7.5	4	19	13	1	10	2	18	10	7	6.55
LIMMA	18	10	19	14	10	14	5	7	3	1	3	6.11
MAST	14	6	15	5	6	2	17	13	12	17	12	5.32
Monocle	10	15	9	11	5	6	1	12	9	4.5	4	7.03
NBSeq	12	12	7	18	3	5	3	8	7	12	6	6.68
NODES	19	16	5	16	16	18	16	17	15	14	10	3.05
ROTS	11	18	17	8	17	15	14	16	16	16	13	3.11
scDD	17	19	18	17	18	19	19	19	19	19	11	1.32
T-test	7	14	6	10	9	13	6	3.5	6	8	16.5	6.37
Wilcox	9	7.5	1	7	14	9.5	9	11	11	4.5	19	6.18

FPR

**Methods**	D1	D2	D3	D4	D5	D6	D7	D8	D9	D10	D11	Rank Score
BPSC	15	11.5	14	1	12	16.5	12	10.5	8	15.5	16	4.63
DECENT	4.5	4	11	3	4	3.5	5	1	1	5	2	9.26
DEGSeq	16	17	16	8.5	8	3.5	13	15	14	13	5	4.79

DESeqLRT	8	13	8	15	19	16.5	18	18	13	18	9	3.39
DESeqNB	2	9	2	6	11	9.5	15	10.5	2	10	8	7.11
DEsingle	13	5.5	3	12.5	15	9.5	11	14	10	11.5	13	5.37
EBSeq	6	1	13	12.5	1	9.5	3	5.5	17	2	1	7.82
edgeRLRT	3	3	10	4	7	9.5	9	3.5	4	8.5	16	7.5
edgeRQLF	1	2	12	2	2	9.5	8	5.5	5	7	16	7.89
EMDomics	4.5	7.5	4	19	14	1	1	2	18	3	6.5	7.34
LIMMA	18	10	19	14	10	14.5	6	7	3	1	3	6.03
MAST	14	5.5	15	5	6	2	17	13	12	17	12	5.34
Monocle	10	15.5	9	11	5	6	2	12	9	5	4	6.92
NBSeq	12	11.5	7	18	3	5	4	8	6.5	11.5	6.5	6.68
NODES	19	15.5	5	16	16	18	16	17	15.5	14	10	3.05
ROTS	11	18	17	8.5	17	14.5	14	16	15.5	15.5	16	3
scDD	17	19	18	17	18	19	19	19	19	19	11	1.32
T-test	7	14	6	10	9	9.5	7	3.5	6.5	8.5	16	6.47
Wilcox	9	7.5	1	7	13	13	10	9	11	5	19	6.08
						F1						
**Methods**	D1	D2	D3	D4	D5	D6	D7	D8	D9	D10	D11	Rank Score
BPSC	15	11	14	1	12	16	12	10	8	15	17	4.68
DECENT	5	4	11	3	4	4	4	1	1	4	2	9.32
DEGSeq	16	17	16	9	8	3	13	15	14	13	5	4.79
DESeqLRT	8	13	8	15	19	17	18	18	13	18	9	3.37
DESeqNB	2	9	2	6	11	11.5	15	9	2	10	8	7.08
DEsingle	13	5	3	12.5	15	7.5	10	14	10	11	14	5.53
EBSeq	6	1	13	12.5	1	11.5	2	6	17	2	1	7.74
edgeRLRT	3	3	10	4	7	9.5	8	3.5	4	8	17	7.53
edgeRQLF	1	2	12	2	2	7.5	7	5	5	7	17	8.03
EMDomics	4	7.5	4	19	13.5	1	11	2	18	3	7	6.84
LIMMA	18	10	19	14	10	14	5	7	3	1	3	6.11
MAST	14	6	15	5	6	2	17	13	12	17	12	5.32
Monocle	10	15	9	11	5	6	1	12	9	5.5	4	6.97
NBSeq	12	12	7	18	3	5	3	8	7	12	6	6.68
NODES	19	16	5	16	16	18	16	17	15	14	10	3.05
ROTS	11	18	17	8	17	15	14	16	16	16	13	3.11
scDD	17	19	18	17	18	19	19	19	19	19	11	1.32
T-test	7	14	6	10	9	13	6	3.5	6	9	17	6.29
Wilcox	9	7.5	1	7	13.5	9.5	9	11	11	5.5	17	6.26

D1: Islam; D2: Tung; D3: Ziegenhain; D4: Grun; D5: Soumillion1; D6: Klein; D7: Savas; D8: Soumillion3; D9: Soumillion2; D10: Chen; D11: Geinhein; TPR: True Positive Rate; FPR: False Positive Rate; F1: F1 Score; FDR: False Discovery Rate; AUROC: Area Under Receiver Operating Curve; ACC: Accuracy

Table S20. Ranking of DE methods across all datasets through AUROC metric.

**Methods**	D1	D2	D3	D4	D5	D6	D7	D8	D9	D10	D11	Rank Score
BPSC	15	11	14	1	12	16	12	10	8	15	16.5	4.71

DECENT	5	4	11	3	4	4	4	1	1	4	2	9.32
DEGSeq	16	17	16	9	8	3	13	15	14	13	5	4.79
DESeqLRT	8	13	8	15	19	17	18	18	13	18	9	3.37
DESeqNB	2	9	2	6	11	11.5	15	9	2	10	8	7.08
DEsingle	13	5	3	12.5	15	7.5	11	14	10	11	14	5.47
EBSeq	6	1	13	12.5	1	11.5	2	6	17	2	1	7.74
edgeRLRT	3	3	10	4	7	9.5	8	3.5	4	8	16.5	7.55
edgeRQLF	1	2	12	2	2	7.5	7	5	5	7	16.5	8.05
EMDomics	4	7.5	4	19	13	1	10	2	18	3	7	6.92
LIMMA	18	10	19	14	10	14	5	7	3	1	3	6.11
MAST	14	6	15	5	6	2	17	13	12	17	12	5.32
Monocle	10	15	9	11	5	6	1	12	9	5.5	4	6.97
NBSeq	12	12	7	18	3	5	3	8	7	12	6	6.68
NODES	19	16	5	16	16	18	16	17	15	14	10	3.05
ROTS	11	18	17	8	17	15	14	16	16	16	13	3.11
scDD	17	19	18	17	18	19	19	19	19	19	11	1.32
T-test	7	14	6	10	9	13	6	3.5	6	9	16.5	6.32
Wilcox	9	7.5	1	7	14	9.5	9	11	11	5.5	19	6.13

D1: Islam; D2: Tung; D3: Ziegenhain; D4: Grun; D5: Soumillon1; D6: Klein; D7: Savas; D8: Soumillon3; D9: Soumillon2; D10: Chen; D11: Geinhein.

Table S21. Ranking of DE methods across all datasets based on the FDR metric.

**Methods**	D1	D2	D3	D4	D5	D6	D7	D8	D9	D10	D11	Rank Score
BPSC	15	12	11.5	1	14	16	12	12	6	16.5	17.5	4.55
DECENT	4	5	13	5	3	2	4	1	1	3	2	9.32
DEGSeq	16	18	16	14	11	4	14	15	18	13	5	4
DESeqLRT	6	11	9	13	19	15	18	18	13	18	9	3.74
DESeqNB	1	8.5	2	6	6	11	17	7	2	10	7	7.5
DEsingle	10	6.5	3	10	10	8	11	13	9	11	17.5	5.84
EBSeq	9	2	14	8	1	13	1	10	14	1	1	7.68
edgeRLRT	3	3	10	4	8	10	8.5	5	5	7	14.5	7.47
edgeRQLF	2	1	11.5	2	2	5	5	4	4	4.5	14.5	8.66
EMDomics	5	8.5	4	19	13	1	10	2	17	9	7	6.55
LIMMA	18	10	19	11	15	17	8.5	3	3	2	4	5.76
MAST	14	4	15	3	5	3	16	11	12	15	12	5.79
Monocle	12	16	7.5	15	7	9	2	14	11	6	3	6.18
NBSeq	11	15	7.5	18	4	6	3	8	8	12	7	6.34
NODES	19	13	6	16	16	18	13	16	15	14	11	3.32
ROTS	13	17	18	9	17	14	15	17	16	16.5	14.5	2.79
scDD	17	19	17	17	18	19	19	19	19	19	10	1.42
T-test	8	14	5	12	9	12	7	6	7	8	14.5	6.18
Wilcox	7	6.5	1	7	12	7	6	9	10	4.5	19	6.89

D1: Islam; D2: Tung; D3: Ziegenhain; D4: Grun; D5: Soumillion1; D6: Klein; D7: Savas; D8: Soumillion3; D9: Soumillion2; D10: Chen; D11: Geinhein;

Table S22. Conflicts among the criteria for ranking of the DE methods for Geinhein data.

Methods	C1	C2	C3	C4	C5	C6	C7	C8	C9	C10	C11	C12	C13
BPSC	17.5	14	16.5	16.5	15.5	16	16.5	17.5	14	17.5	16	16.5	9
DECENT	2	2	2	2	2	2	2	2	2	2	2	2	19
DEGSeq	5	5	5	5	5	5	5	5	5	5	5	5	3
DESeqLRT	9	9	9	9	9	9	9	9	9	9	9	9	5
DESeqNB	8	8	8	8	8	8	8	7	8	8	8	8	6
DEsingle	14	13	14	14	15.5	13	14	17.5	13	14	13	14	18
EBSeq	1	1	1	1	1	1	1	1	1	1	1	1	17
edgeRLRT	17.5	18	16.5	16.5	15.5	16	16.5	14.5	18	17.5	16	16.5	8
edgeRQLF	16	17	16.5	16.5	15.5	16	16.5	14.5	17	16	16	16.5	7
EMDomics	7	6.5	7	7	6.5	6.5	7	7	6.5	7	6.5	7	16
LIMMA	3	3	3	3	3	3	3	4	3	3	3	3	4
MAST	12	12	12	12	12	12	12	12	12	12	12	12	11
Monocle	4	4	4	4	4	4	4	3	4	4	4	4	12
NBSeq	6	6.5	6	6	6.5	6.5	6	7	6.5	6	6.5	6	14
NODES	10	10	10	10	10	10	10	11	10	10	10	10	13
ROTS	13	15	13	13	15.5	16	13	14.5	15	13	16	13	15
scDD	11	11	11	11	11	11	11	10	11	11	11	11	10
T-test	15	16	16.5	16.5	15.5	16	16.5	14.5	16	15	16	16.5	1
Wilcox	19	19	19	19	19	19	19	19	19	19	19	19	2

C1:True Positive; C2: True negative; C3: True Positive Rate; C4: Positive Prediction Rate; C5: Negative Prediction Value; C6: Accuracy; C7: F1 score; C8: AUROC; C9: False Positive; C10: False Negative; C11: False Positive Rate; C12: False Discovery Rate; C13: Runtime

Table S23. Conflicts among the criteria for ranking of the DE methods for Islam data.

Methods	C1	C2	C3	C4	C5	C6	C7	C8	C9	C10	C11	C12	C13
BPSC	15	15	15	15	15	15	15	15	15	15	15	15	9
DECENT	5	5	5	5	4.5	5	5	4	5	5	4.5	5	19
DEGSeq	16	16	16	16	16	16	16	16	16	16	16	16	3
DESeqLRT	8	8	8	8	8	8	8	6	8	8	8	8	5
DESeqNB	2	2	2	2	2	2	2	1	2	2	2	2	6
DEsingle	13	13	13	13	13	13	13	10	13	13	13	13	18
EBSeq	6	6	6	6	6	6	6	9	6	6	6	6	17
edgeRLRT	3	3	3	3	3	3	3	3	3	3	3	3	8
edgeRQLF	1	1	1	1	1	1	1	2	1	1	1	1	7
EMDomics	4	4	4	4	4.5	4	4	5	4	4	4.5	4	16
LIMMA	18	18	18	18	18	18	18	18	18	18	18	18	4
MAST	14	14	14	14	14	14	14	14	14	14	14	14	11
Monocle	10	10	10	10	10	10	10	12	10	10	10	10	12
NBSeq	12	12	12	12	12	12	12	11	12	12	12	12	14
NODES	19	19	19	19	19	19	19	19	19	19	19	19	13
ROTS	11	11	11	11	11	11	11	13	11	11	11	11	15
scDD	17	17	17	17	17	17	17	17	17	17	17	17	10
T-test	7	7	7	7	7	7	7	8	7	7	7	7	1
Wilcox	9	9	9	9	9	9	9	7	9	9	9	9	2

C1:True Positive; C2: True negative; C3: True Positive Rate; C4: Positive Prediction Rate; C5: Negative Prediction Value; C6: Accuracy; C7: F1 score; C8: AUROC; C9: False Positive; C10: False Negative; C11: False Positive Rate; C12: False Discovery Rate; C13: Runtime

Table S24. Conflicts among the criteria for ranking of the DE methods for Tung data.

Methods	C1	C2	C3	C4	C5	C6	C7	C8	C9	C10	C11	C12	C13
BPSC	11	11	11	11	11.5	11	11	12	11	11	11.5	11	9
DECENT	4	4	4	4	4	4	4	5	4	4	4	4	19
DEGSeq	17	17	17	17	17	17	17	18	17	17	17	17	3
DESeqLRT	13	13	13	13	13	13	13	11	13	13	13	13	5
DESeqNB	9	9	9	9	9	9	9	8.5	9	9	9	9	6
DEsingle	5	5	5	5	5.5	5.5	5	6.5	5	5	5.5	5	18
EBSeq	1	1	1	1	1	1	1	2	1	1	1	1	17
EdgeR	3	3	3	3	3	3	3	3	3	3	3	3	8
EdgeRQLF	2	2	2	2	2	2	2	1	2	2	2	2	7
LIMMA	10	10	10	10	10	10	10	10	10	10	10	10	16
MAST	6	6	6	6	5.5	5.5	6	4	6	6	5.5	6	4
Monocle	15	15	15	15	15.5	15	15	16	15	15	15.5	15	11
NBSeq	12	12	12	12	11.5	12	12	15	12	12	11.5	12	12
NODES	16	16	16	16	15.5	16	16	13	16	16	15.5	16	14
ROTS	18	18	18	18	18	18	18	17	18	18	18	18	13
scDD	19	19	19	19	19	19	19	19	19	19	19	19	15
T-test	14	14	14	14	14	14	14	14	14	14	14	14	10
Wilcox	7	7	7.5	7.5	7.5	7.5	7.5	6.5	7	7	7.5	7.5	1
EMDomics	8	8	7.5	7.5	7.5	7.5	7.5	8.5	8	8	7.5	7.5	2

C1:True Positive; C2: True negative; C3: True Positive Rate; C4: Positive Prediction Rate; C5: Negative Prediction Value; C6: Accuracy; C7: F1 score; C8: AUROC; C9: False Positive; C10: False Negative; C11: False Positive Rate; C12: False Discovery Rate; C13: Runtime

Table S25. Conflicts among the criteria for ranking of the DE methods for Islam Ziegenhain data.

Methods	C1	C2	C3	C4	C5	C6	C7	C8	C9	C10	C11	C12	C13
BPSC	14	14	14	14	14	14	14	11.5	14	14	14	14	9
DECENT	11	11	11	11	11	11	11	13	11	11	11	11	19
DEGSeq	16	16	16	16	16	16	16	16	16	16	16	16	3
DESeqLRT	8	8	8	8	8	8	8	9	8	8	8	8	5
DESeqNB	2	2	2	2	2	2	2	2	2	2	2	2	6
DEsingle	3	3	3	3	3	3	3	3	3	3	3	3	18
EBSeq	13	13	13	13	13	13	13	14	13	13	13	13	17
edgeRLRT	10	10	10	10	10	10	10	10	10	10	10	10	8
edgeRQLF	12	12	12	12	12	12	12	11.5	12	12	12	12	7
EMDomics	4	4	4	4	4	4	4	4	4	4	4	4	16
LIMMA	19	19	19	19	19	19	19	19	19	19	19	19	4
MAST	15	15	15	15	15	15	15	15	15	15	15	15	11
Monocle	9	9	9	9	9	9	9	7.5	9	9	9	9	12
NBSeq	7	7	7	7	7	7	7	7.5	7	7	7	7	14
NODES	5	5	5	5	5	5	5	6	5	5	5	5	13
ROTS	17	17	17	17	17	17	17	18	17	17	17	17	15
scDD	18	18	18	18	18	18	18	17	18	18	18	18	10

Ttest	6	6	6	6	6	6	6	5	6	6	6	6	1
Wilcox	1	1	1	1	1	1	1	1	1	1	1	1	2

C1:True Positive; C2: True negative; C3: True Positive Rate; C4: Positive Prediction Rate; C5: Negative Prediction Value; C6: Accuracy; C7: F1 score; C8: AUROC; C9: False Positive; C10: False Negative; C11: False Positive Rate; C12: False Discovery Rate; C13: Runtime

Table S26. Conflicts among the criteria for ranking of the DE methods for Grun data.

Methods	C1	C2	C3	C4	C5	C6	C7	C8	C9	C10	C11	C12	C13
BPSC	1	2	1	1	1	1	1	1	1	1	1	1	9
DECENT	3	4	3	3	3	3	3	5	3	3	3	3	19
DEGSeq	9	10	9	9	8.5	9	9	14	9	9	8.5	9	3
DESeqLRT	15	16	15	15	15	15	15	13	15	15	15	15	5
DESeqNB	6	7	6	6	6	6	6	6	6	6	6	6	6
DEsingle	12.5	13.5	12.5	12.5	12.5	12.5	12.5	10	12.5	12.5	12.5	12.5	18
EBSeq	12.5	13.5	12.5	12.5	12.5	12.5	12.5	8	12.5	12.5	12.5	12.5	17
edgeRLRT	4	5	4	4	4	4	4	4	4	4	4	4	8
edgeRQLF	2	3	2	2	2	2	2	2	2	2	2	2	7
EMDomics	19	1	19	19	19	19	19	19	19	19	19	19	16
LIMMA	14	15	14	14	14	14	14	11	14	14	14	14	4
MAST	5	6	5	5	5	5	5	3	5	5	5	5	11
Monocle	11	12	11	11	11	11	11	15	11	11	11	11	12
NBSeq	18	19	18	18	18	18	18	18	18	18	18	18	14
NODES	16	17	16	16	16	16	16	16	16	16	16	16	13
ROTS	8	9	8	8	8.5	8	8	9	8	8	8.5	8	15
scDD	17	18	17	17	17	17	17	17	17	17	17	17	10
T-test	10	11	10	10	10	10	10	12	10	10	10	10	1
Wilcox	7	8	7	7	7	7	7	7	7	7	7	7	2

C1:True Positive; C2: True negative; C3: True Positive Rate; C4: Positive Prediction Rate; C5: Negative Prediction Value; C6: Accuracy; C7: F1 score; C8: AUROC; C9: False Positive; C10: False Negative; C11: False Positive Rate; C12: False Discovery Rate; C13: Runtime

Table S27. Conflicts among the criteria for ranking of the DE methods for Soumillon1 data.

Methods	C1	C2	C3	C4	C5	C6	C7	C8	C9	C10	C11	C12	C13
BPSC	12	12	12	12	12	12	12	14	12	12	12	12	9
DECENT	4	3	4	4	4	4	4	3	4	4	4	4	19
DEGSeq	8	8	8	8	8	8	8	11	8	8	8	8	3
DESeqLRT	19	19	19	19	19	19	19	19	19	19	19	19	5
DESeqNB	11	11	11	11	11	11	11	6	11	11	11	11	6
DEsingle	15	15	15	15	15	15	15	10	15	15	15	15	18
EBSeq	1	1	1	1	1	1	1	1	1	1	1	1	17
edgeRLRT	7	7	7	7	7	6.5	7	8	7	7	7	7	8
edgeRQLF	2	2	2	2	2	2	2	2	2	2	2	2	7
EMDomics	13	13	13	13	14	13	13	13	13	13	14	13	16
LIMMA	10	10	10	10	10	10	10	15	10	10	10	10	4
MAST	6	6	6	6	6	6.5	6	5	6	6	6	6	11
Monocle	5	5	5	5	5	5	5	7	5	5	5	5	12
NBSeq	3	4	3	3	3	3	3	4	3	3	3	3	14
NODES	16	16	16	16	16	16	16	16	16	16	16	16	13
ROTS	17	17	17	17	17	17	17	17	17	17	17	17	15

scDD	18	18	18	18	18	18	18	18	18	18	18	18	10
Ttest	9	9	9	9	9	9	9	9	9	9	9	9	1
Wilcox	14	14	14	14	13	14	14	12	14	14	13	14	2

C1:True Positive; C2: True negative; C3: True Positive Rate; C4: Positive Prediction Rate; C5: Negative Prediction Value; C6: Accuracy; C7: F1 score; C8: AUROC; C9: False Positive; C10: False Negative; C11: False Positive Rate; C12: False Discovery Rate; C13: Runtime

Table S28. Conflicts among the criteria for ranking of the DE methods for Klein data.

Methods	C1	C2	C3	C4	C5	C6	C7	C8	C9	C10	C11	C12	C13
BPSC	16	16	16	16	16.5	16	16	16	16	16	16.5	16	9
DECENT	4	4	4	4	3.5	4	4	2	4	4	3.5	4	19
DEGSeq	3	3	3	3	3.5	3	3	4	3	3	3.5	3	3
DESeqLRT	17	17	17	17	16.5	17	17	15	17	17	16.5	17	5
DESeqNB	12	12	11.5	11.5	9.5	11.5	11.5	11	12	12	9.5	11.5	6
DEsingle	7	7	7.5	7.5	9.5	7.5	7.5	8	7	7	9.5	7.5	18
EBSeq	11	11	11.5	11.5	9.5	11.5	11.5	13	11	11	9.5	11.5	17
edgeRLRT	10	10	9.5	9.5	9.5	11.5	9.5	10	10	10	9.5	9.5	8
edgeRQLF	8	8	7.5	7.5	9.5	7.5	7.5	5	8	8	9.5	7.5	7
EMDomics	1	1	1	1	1	1	1	1	1	1	1	1	16
LIMMA	14	14	14	14	14.5	14	14	17	14	14	14.5	14	4
MAST	2	2	2	2	2	2	2	3	2	2	2	2	11
Monocle	6	6	6	6	6	6	6	9	6	6	6	6	12
NBSeq	5	5	5	5	5	5	5	6	5	5	5	5	14
NODES	18	18	18	18	18	18	18	18	18	18	18	18	13
ROTS	15	15	15	15	14.5	15	15	14	15	15	14.5	15	15
scDD	19	19	19	19	19	19	19	19	19	19	19	19	10
T-test	13	13	13	13	9.5	11.5	13	12	13	13	9.5	13	1
Wilcox	9	9	9.5	9.5	13	9	9.5	7	9	9	13	9.5	2

C1:True Positive; C2: True negative; C3: True Positive Rate; C4: Positive Prediction Rate; C5: Negative Prediction Value; C6: Accuracy; C7: F1 score; C8: AUROC; C9: False Positive; C10: False Negative; C11: False Positive Rate; C12: False Discovery Rate; C13: Runtime

Table S29. Conflicts among the criteria for ranking of the DE methods for Soumillon3 data.

Methods	C1	C2	C3	C4	C5	C6	C7	C8	C9	C10	C11	C12	C13
BPSC	10	10	10	10	10.5	9.5	10	12	10	10	10.5	10	9
DECENT	1	1	1	1	1	1	1	1	1	1	1	1	19
DEGSeq	15	15	15	15	15	15	15	15	15	15	15	15	3
DESeqLRT	18	18	18	18	18	18	18	18	18	18	18	18	5
DESeqNB	9	9	9	9	10.5	9.5	9	7	9	9	10.5	9	6
DEsingle	14	14	14	14	14	14	14	13	14	14	14	14	18
EBSeq	6	6	6	6	5.5	6	6	10	6	6	5.5	6	17
edgeRLRT	3.5	3.5	3.5	3.5	3.5	3.5	3.5	5	3.5	3.5	3.5	3.5	8
edgeRQLF	5	5	5	5	5.5	5	5	4	5	5	5.5	5	7
EMDomics	2	2	2	2	2	2	2	2	2	2	2	2	16
LIMMA	7	7	7	7	7	7	7	3	7	7	7	7	4
MAST	13	13	13	13	13	13	13	11	13	13	13	13	11
Monocle	12	12	12	12	12	12	12	14	12	12	12	12	12

NBSeq	8	8	8	8	8	8	8	8	8	8	8	8	14
NODES	17	17	17	17	17	17	17	16	17	17	17	17	13
ROTS	16	16	16	16	16	16	16	17	16	16	16	16	15
scDD	19	19	19	19	19	19	19	19	19	19	19	19	10
T-test	3.5	3.5	3.5	3.5	3.5	3.5	3.5	6	3.5	3.5	3.5	3.5	1
Wilcox	11	11	11	11	9	11	11	9	11	11	9	11	2

C1:True Positive; C2: True negative; C3: True Positive Rate; C4: Positive Prediction Rate; C5: Negative Prediction Value; C6: Accuracy; C7: F1 score; C8: AUROC; C9: False Positive; C10: False Negative; C11: False Positive Rate; C12: False Discovery Rate; C13: Runtime

Table S30. Conflicts among the criteria for ranking of the DE methods for Soumillon2 data.

Methods	C1	C2	C3	C4	C5	C6	C7	C8	C9	C10	C11	C12	C13
BPSC	8	8	8	8	8	8	8	6	8	8	8	8	9
DECENT	1	1	1	1	1	1	1	1	1	1	1	1	19
DEGSeq	14	14	14	14	14	14	14	18	14	14	14	14	3
DESeqLRT	13	13	13	13	13	13	13	13	13	13	13	13	5
DESeqNB	2	2	2	2	2	2	2	2	2	2	2	2	6
DEsingle	10	10	10	10	10	10	10	9	10	10	10	10	18
EBSeq	17	17	17	17	17	17	17	14	17	17	17	17	17
edgeRLRT	4	4	4	4	4	4	4	5	4	4	4	4	8
edgeRQLF	5	5	5	5	5	5	5	4	5	5	5	5	7
EMDomics	18	18	18	18	18	18	18	17	18	18	18	18	16
LIMMA	3	3	3	3	3	3	3	3	3	3	3	3	4
MAST	12	12	12	12	12	12	12	12	12	12	12	12	11
Monocle	9	9	9	9	9	9	9	11	9	9	9	9	12
NBSeq	7	7	7	7	6.5	6.5	7	8	7	7	6.5	7	14
NODES	15	15	15	15	15.5	15.5	15	15	15	15	15.5	15	13
ROTS	16	16	16	16	15.5	15.5	16	16	16	16	15.5	16	15
scDD	19	19	19	19	19	19	19	19	19	19	19	19	10
T-test	6	6	6	6	6.5	6.5	6	7	6	6	6.5	6	1
Wilcox	8	8	7.5	7.5	7.5	7.5	7.5	8.5	8	8	7.5	7.5	2

C1:True Positive; C2: True negative; C3: True Positive Rate; C4: Positive Prediction Rate; C5: Negative Prediction Value; C6: Accuracy; C7: F1 score; C8: AUROC; C9: False Positive; C10: False Negative; C11: False Positive Rate; C12: False Discovery Rate; C13: Runtime

Table S31. Effect of the number of cells on the performance (ranking) of the DE methods assessed through AUROC.

Methods	Small				Score	Medium				Score	Large			Score
BPSC	15	12	11.5	1	2.13	14	16	12	17.5	1.08	12	6	16.5	1.34
DECENT	4	5	13	5	2.79	3	2	4	2	3.63	1	1	3	2.89
DEGSeq	16	18	16	14	0.84	11	4	14	5	2.42	15	18	13	0.74
DESeqLRT	6	11	9	13	2.16	19	15	18	9	1.00	18	13	18	0.58

DESeqNB	1	8.5	2	6	3.29	6	11	17	7	2.05	7	2	10	2.16
DEsingle	10	6.5	3	10	2.66	10	8	11	17.5	1.76	13	9	11	1.42
EBSeq	9	2	14	8	2.47	1	13	1	1	3.37	10	14	1	1.84
edgeRLRT	3	3	10	4	3.16	8	10	8.5	14.5	2.05	5	5	7	2.26
edgeRQLF	2	1	11.5	2	3.34	2	5	5	14.5	2.82	4	4	4.5	2.50
EMDomics	5	8.5	4	19	2.29	13	1	10	7	2.58	2	17	9	1.68
LIMMA	18	10	19	11	1.16	15	17	8.5	4	1.87	3	3	2	2.74
MAST	14	4	15	3	2.32	5	3	16	12	2.32	11	12	15	1.16
Monocle	12	16	7.5	15	1.55	7	9	2	3	3.11	14	11	6	1.53
NBSeq	11	15	7.5	18	1.50	4	6	3	7	3.16	8	8	12	1.68
NODES	19	13	6	16	1.37	16	18	13	11	1.16	16	15	14	0.79
ROTS	13	17	18	9	1.21	17	14	15	14.5	1.03	17	16	16.5	0.55
scDD	17	19	17	17	0.53	18	19	19	10	0.74	19	19	19	0.16
Ttest	8	14	5	12	2.16	9	12	7	14.5	1.97	6	7	8	2.05
Wilcox	7	6.5	1	7	3.08	12	7	6	19	1.89	9	10	4.5	1.92

Small number of cells (< 600) in scRNA-seq; Medium number of cells (600 – 2000) scRNA-seq; Large number of cells (> 2000); score: Average of the rank scores computed through Eq. 32 based on the AUROC measure

Table S32. ANOVA table for studying the effects of number of cells and cells/group on perform ance of DE methods.

Source of variation	df	SS	MS	F-value	Pr (> F)	Sig.
Methods	18	0.9223	0.05124	4.4213	4.49E-08	***
Data	10	2.7733	0.27733	23.930	2.00E-16	***
Number of cells	2	0.6117	0.30585	26.391	5.58E-11	***
Cells/group	2	0.6348	0.3174	27.388	5.64E-11	***
Residuals	176	2.0397	0.0116			
Total	208	6.9818	0.0336			

df: degrees of freedom; SS: Sum of Squares; MS: Mean Squares; Sig. Significance status;
***: values significant at 0.1% level of significance

References

1. Islam S, Kjällquist U, Moliner A, Zajac P, Fan JB, Lönnerberg P, et al. Characterization of the single-cell transcriptional landscape by highly multiplex RNA-seq. Genome Res. 2011. doi:10.1101/gr.110882.110
2. Tung P-Y, Blischak JD, Hsiao CJ, Knowles DA, Burnett JE, Pritchard JK, et al. Batch effects and the effective design of single-cell gene expression studies. Sci Rep. 2017;7: 39921. doi:10.1038/srep39921

3. Soumillon M, Cacchiarelli D, Semrau S, van Oudenaarden A, Mikkelsen TS. Characterization of directed differentiation by high-throughput single-cell RNA-Seq. bioRxiv. 2014. doi:10.1101/003236
4. Klein AM, Mazutis L, Akartuna I, Tallapragada N, Veres A, Li V, et al. Droplet barcoding for single-cell transcriptomics applied to embryonic stem cells. Cell. 2015. doi:10.1016/j.cell.2015.04.044
5. Gierahn TM, Wadsworth MH, Hughes TK, Bryson BD, Butler A, Satija R, et al. Seq-Well: Portable, low-cost rna sequencing of single cells at high throughput. Nat Methods. 2017. doi:10.1038/nmeth.4179
6. Das S, Rai SN. SwarnSeq: An improved statistical approach for differential expression analysis of single-cell RNA-seq data. Genomics. 2021;113: 1308–1324. doi:10.1016/j.ygeno.2021.02.014
7. Das S, Rai SN. Statistical methods for analysis of single-cell RNA-sequencing data. MethodsX. 2021;8: 101580. doi:https://doi.org/10.1016/j.mex.2021.101580
8. Moliner A, Enfors P, Ibáñez CF, Andäng M. Mouse embryonic stem cell-derived spheres with distinct neurogenic potentials. Stem Cells Dev. 2008. doi:10.1089/scd.2007.0211
9. Liang Y, Zhang F, Wang J, Joshi T, Wang Y, Xu D. Prediction of Drought-Resistant Genes in Arabidopsis thaliana Using SVM-RFE. Zhu D, editor. PLoS One. 2011;6: e21750. doi:10.1371/journal.pone.0021750
10. Das S, Meher PK, Rai A, Bhar LM, Mandal BN. Statistical Approaches for Gene Selection, Hub Gene Identification and Module Interaction in Gene Co-Expression Network Analysis: An Application to Aluminum Stress in Soybean (Glycine max L.). Tian Z, editor. PLoS One. 2017;12: e0169605. doi:10.1371/journal.pone.0169605
11. Li J, Tibshirani R. Finding consistent patterns: A nonparametric approach for identifying differential expression in RNA-Seq data. Stat Methods Med Res. 2013. doi:10.1177/0962280211428386
12. Love MI, Huber W, Anders S. Moderated estimation of fold change and dispersion for RNA-seq data with DESeq2. Genome Biol. 2014. doi:10.1186/s13059-014-0550-8
13. Love MI, Anders S, Huber W. Differential analysis of count data - the DESeq2 package. Genome Biology. 2014. doi:110.1186/s13059-014-0550-8
14. Robinson MD, McCarthy DJ, Smyth GK. edgeR: a Bioconductor package for differential expression analysis of digital gene expression data. Bioinformatics. 2010;26: 139–140. doi:10.1093/bioinformatics/btp616
15. Van den Berge K, Perraudeau F, Soneson C, Love MI, Risso D, Vert J-P, et al. Observation weights unlock bulk RNA-seq tools for zero inflation and single-cell applications. Genome Biol. 2018;19: 24. doi:10.1186/s13059-018-1406-4
16. Risso D, Perraudeau F, Gribkova S, Dudoit S, Vert J-P. A general and flexible method for signal extraction from single-cell RNA-seq data. Nat Commun. 2018;9: 284. doi:10.1038/s41467-017-02554-5
17. Van den Berge K, Soneson C, Love MI, Robinson MD, Clement L. zingeR: unlocking RNA-seq tools for zero-inflation and single cell applications. doi.org. 2017. doi:10.1101/157982
18. Miao Z, Deng K, Wang X, Zhang X. DEsingle for detecting three types of differential expression in single-cell RNA-seq data. Berger B, editor. Bioinformatics. 2018;34: 3223–3224. doi:10.1093/bioinformatics/bty332
19. Ye C, Speed TP, Salim A. DECENT: differential expression with capture efficiency adjustmeNT for single-cell RNA-seq data. Berger B, editor. Bioinformatics. 2019;35: 5155–

5162. doi:10.1093/bioinformatics/btz453
20. Zeileis A, Kleiber C, Jackman S. Regression models for count data in R. J Stat Softw. 2008. doi:10.18637/jss.v027.i08
21. KEMP CD, KEMP AW. Some properties of the "Hermite" distribution. Biometrika. 1965;52: 381–394. doi:10.1093/biomet/52.3-4.381
22. Robinson MD, McCarthy DJ, Smyth GK. edgeR: a Bioconductor package for differential expression analysis of digital gene expression data. Bioinformatics. 2010;26: 139–140. doi:10.1093/bioinformatics/btp616
23. Robinson MD, Oshlack A. A scaling normalization method for differential expression analysis of RNA-seq data. Genome Biol. 2010. doi:10.1186/gb-2010-11-3-r25
24. Anders S, Huber W. Differential expression analysis for sequence count data. Genome Biol. 2010;11: R106. doi:10.1186/gb-2010-11-10-r106
25. Wang L, Feng Z, Wang X, Wang X, Zhang X. DEGseq: An R package for identifying differentially expressed genes from RNA-seq data. Bioinformatics. 2009. doi:10.1093/bioinformatics/btp612
26. Smyth GK. limma: Linear Models for Microarray Data. Bioinformatics and Computational Biology Solutions Using R and Bioconductor. doi:10.1007/0-387-29362-0_23
27. Seyednasrollah F, Rantanen K, Jaakkola P, Elo LL. ROTS: Reproducible RNA-seq biomarker detector - Prognostic markers for clear cell renal cell cancer. Nucleic Acids Res. 2016. doi:10.1093/nar/gkv806
28. Di Y, Schafer DW, Cumbie JS, Chang JH. The NBP negative binomial model for assessing differential gene expression from RNA-Seq. Stat Appl Genet Mol Biol. 2011. doi:10.2202/1544-6115.1637
29. Leng N, Dawson JA, Thomson JA, Ruotti V, Rissman AI, Smits BMG, et al. EBSeq: an empirical Bayes hierarchical model for inference in RNA-seq experiments. Bioinformatics. 2013;29: 1035–1043. doi:10.1093/bioinformatics/btt087
30. Welch BL. The Generalization of `Student's' Problem when Several Different Population Variances are Involved. Biometrika. 1947. doi:10.2307/2332510
31. Wilcoxon F. Individual Comparisons by Ranking Methods. Biometrics Bull. 1945;1: 80. doi:10.2307/3001968
32. Qiu X, Hill A, Packer J, Lin D, Ma Y-A, Trapnell C. Single-cell mRNA quantification and differential analysis with Census. Nat Methods. 2017;14: 309–315. doi:10.1038/nmeth.4150
33. Finak G, McDavid A, Yajima M, Deng J, Gersuk V, Shalek AK, et al. MAST: a flexible statistical framework for assessing transcriptional changes and characterizing heterogeneity in single-cell RNA sequencing data. Genome Biol. 2015;16: 278. doi:10.1186/s13059-015-0844-5
34. Korthauer KD, Chu LF, Newton MA, Li Y, Thomson J, Stewart R, et al. A statistical approach for identifying differential distributions in single-cell RNA-seq experiments. Genome Biol. 2016. doi:10.1186/s13059-016-1077-y
35. Vu TN, Wills QF, Kalari KR, Niu N, Wang L, Rantalainen M, et al. Beta-Poisson model for single-cell RNA-seq data analyses. Bioinformatics. 2016. doi:10.1093/bioinformatics/btw202
36. Sengupta D, Rayan NA, Lim M, Lim B, Prabhakar S. Fast, scalable and accurate differential expression analysis for single cells. bioRxiv. 2016. doi:10.1101/049734
37. Nabavi S, Schmolze D, Maitituoheti M, Malladi S, Beck AH. EMDomics: A robust and powerful method for the identification of genes differentially expressed between heterogeneous classes. Bioinformatics. 2016. doi:10.1093/bioinformatics/btv634

38. Yoon K, Hwang C-L. Multiple Attribute Decision Making. Multiple Attribute Decision Making. 2455 Teller Road, Thousand Oaks California 91320 United States of America: SAGE Publications, Inc.; 1995. doi:10.4135/9781412985161
39. Khezrian M, Jahan A, Kadir WMNW, Ibrahim S. An approach for web service selection based on confidence level of decision maker. PLoS One. 2014. doi:10.1371/journal.pone.0097831
40. Ahn BS. Compatible weighting method with rank order centroid: Maximum entropy ordered weighted averaging approach. Eur J Oper Res. 2011. doi:10.1016/j.ejor.2011.02.017
41. Das S, Rai A, Mishra DC, Rai SN. Statistical approach for selection of biologically informative genes. Gene. 2018;655. doi:10.1016/j.gene.2018.02.044
42. Assari A. Role of public participation in sustainability of historical city: usage of TOPSIS method. Indian J Sci Technol. 2012. doi:10.17485/ijst/2012/v5i3.2
43. Boon WC, Petkovic-Duran K, Zhu Y, Manasseh R, Horne MK, Aumann TD. Increasing cDNA yields from single-cell quantities of mRNA in standard laboratory reverse transcriptase reactions using acoustic microstreaming. J Vis Exp. 2011. doi:10.3791/3144
44. Macaulay IC, Voet T. Single Cell Genomics: Advances and Future Perspectives. PLoS Genetics. 2014. doi:10.1371/journal.pgen.1004126
45. Marinov GK, Williams BA, McCue K, Schroth GP, Gertz J, Myers RM, et al. From single-cell to cell-pool transcriptomes: Stochasticity in gene expression and RNA splicing. Genome Res. 2014. doi:10.1101/gr.161034.113
46. Kharchenko P V., Silberstein L, Scadden DT. Bayesian approach to single-cell differential expression analysis. Nat Methods. 2014;11: 740–742. doi:10.1038/nmeth.2967
47. Pierson E, Yau C. ZIFA: Dimensionality reduction for zero-inflated single-cell gene expression analysis. Genome Biol. 2015. doi:10.1186/s13059-015-0805-z
48. Wang Y, Navin NE. Advances and Applications of Single-Cell Sequencing Technologies. Molecular Cell. 2015. doi:10.1016/j.molcel.2015.05.005
49. McElduff F, Cortina-Borja M, Chan SK, Wade A. When t-tests or Wilcoxon-Mann-Whitney tests won't do. Am J Physiol - Adv Physiol Educ. 2010. doi:10.1152/advan.00017.2010
50. Hardcastle TJ, Kelly KA. BaySeq: Empirical Bayesian methods for identifying differential expression in sequence count data. BMC Bioinformatics. 2010. doi:10.1186/1471-2105-11-422
51. Trapnell C, Hendrickson DG, Sauvageau M, Goff L, Rinn JL, Pachter L. Differential analysis of gene regulation at transcript resolution with RNA-seq. Nat Biotechnol. 2013. doi:10.1038/nbt.2450
52. Jackman S, Tahk A, Zeileis A, Maimone C, Fearon J, Maintainer ZM. Package "pscl": Political Science Computational Laboratory. R Packag Vignettes Collect. 2017.
